# Visible Light-Mediated Monofluoromethylation/Acylation of Olefins by Dual Organo-Catalysis

**DOI:** 10.3390/molecules29040790

**Published:** 2024-02-08

**Authors:** Jiuli Xia, Yunliang Guo, Zhiguang Lv, Jiaqiong Sun, Guangfan Zheng, Qian Zhang

**Affiliations:** 1 Key Laboratory of Functional Organic Molecule Design & Synthesis of Jilin Province, Department of Chemistry, Northeast Normal University, Changchun 130024, China; xiajl699@nenu.edu.cn (J.X.); lvzhiguang@nenu.edu.cn (Z.L.); zhangq651@nenu.edu.cn (Q.Z.); 2 School of Environment, Northeast Normal University, Changchun 130117, China; guoyl367@nenu.edu.cn; 3 State Key Laboratory of Organometallic Chemistry, Shanghai Institute of Organic Chemistry, Chinese Academy of Sciences, 345 Lingling Road, Shanghai 200032, China

**Keywords:** metal-free, NHCs, organic photocatalyst, monofluoromethylation/acylation, alkenes

## Abstract

Monofluoromethyl (CH_2_F) motifs exhibit unique bioactivities and are considered privileged units in drug discovery. The radical monofluoromethylative difunctionalization of alkenes stands out as an appealing approach to access CH_2_F-containing compounds. However, this strategy remains largely underdeveloped, particularly under metal-free conditions. In this study, we report on visible light-mediated three-component monofluoromethylation/acylation of styrene derivatives employing NHC and organic photocatalyst dual catalysis. A diverse array of α-aryl-*β*-monofluoromethyl ketones was successfully synthesized with excellent functional group tolerance and selectivity. The mild and metal-free CH_2_F radical generation strategy from NaSO_2_CFH_2_ holds potential for further applications in fluoroalkyl radical chemistry.

## 1. Introduction

**Scheme 1:**
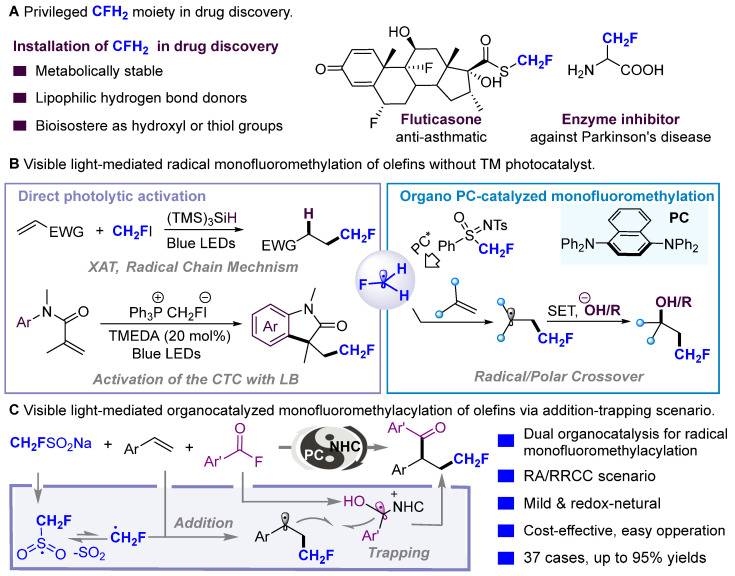
Motivation for visible light-mediated organocatalyzed monofluoromethylation/acylation of alkenes.

The incorporation of fluoroalkyl groups into readily available molecules has garnered increasing attention due to the prevalence of fluoroalkyl-containing compounds in bioactive molecules and drug discovery [1,2,3,4,5]. Among various methodologies [6], the radical approach [7,8,9,10,11,12,13,14] has emerged as particularly noteworthy due to its mild conditions, broad substrate scope, and high selectivity, offering compelling alternatives for synthesizing fluoroalkyl-containing compounds. Notably, CH_2_F, as a distinct fluoroalkyl group, has been recognized for its potential as a metabolically stable and lipophilic bioisostere for NH_2_, OH, SH, etc. and is thus considered a privileged unit in drug discovery (Scheme 1A, Left) [15,16]. For example, fluticasone is a widely used corticosteroid drug, and β-fluorinated amino acid could act as an enzyme inhibitor and be suitable against Parkinson’s disease (Scheme 1A, Right) [17]. However, compared with the well-established well-developed radical installation of CF_3_ or CF_2_H and CF_2_R groups, radical monofluoromethylation [18,19,20,21,22,23,24,25,26,27,28,29,30,31,32,33,34,35,36,37] represents an emerging area with great demand. Olefins, being fundamental and prevalent chemical feedstocks, have witnessed considerable progress in transition metal (TM) catalysis including radical hydromonofluoromethylation [25] and monofluoromethylative difunctionalization [26,27,28,29,30,31], where TMs serve as redox catalysts [25,26,27] or photocatalysts (PC) [28,29,30,31]. Metal-free transformations mediated by visible light are particularly attractive owing to their mild conditions and the potential to reduce costs and toxicity associated with metal catalysts. In this context, in 2021, the Chen and Wang group [32] achieved σ-hole effect-facilitated photolysis of phosphonium iodide salts for the monofluoromethylation/acylation of olefins, while the Gouverneur [33] and Gaunt [34] groups independently developed radical hydrofluoromethylation of alkenes using CH_2_FI. The utilization of organo-photocatalysts has further enriched the initiation pathways for CH_2_F radicals. Koike, Akita, and co-workers [35,36] developed a highly reducing organic PC (1,4-bis(diphenylamino)naphthalene) to catalyze the hydroxy-monofluoromethylation of alkenes via a radical–polar crossover strategy. Despite the significance of these accomplishments, developing a novel organic PC-catalyzed radical difunctionalization system with a bench-stable and cost-effective CFH_2_ source was highly desirable.

On the other hand, stemming from the groundbreaking contributions of Studer [37,38], Ohmiya [39,40], and Chi [41] et al., NHC-attached persistent Breslow intermediate radicals (BIR) [42,43,44] have emerged as valuable acyl radical equivalents, enabling radical-radical cross-coupling and thereby paving the way for a new paradigm in radical acylation chemistry [45,46,47,48]. In radical NHC catalysis, persistent BIR and transient radical species can be concurrently generated at a comparable rate through a single electron transfer process. The highly selective radical addition/coupling (RAC) scenario [49,50,51] involving olefins, wherein transient radicals add to double bonds and are subsequently trapped by BIR, provides alternative routes for the precise construction of functionalized ketone units. As demonstrated by Studer [52,53,54,55,56,57,58,59,60], Schedit [61,62,63,64,65,66,67], and others [68,69,70,71,72,73,74], carboxylic acid derivatives can act as both sources of BIR and oxidants under the cooperation of NHC with PC, which in combination with reductive radical sources realizing radical acylative functionalization of olefins [52,53,54,55,56,57,67,68,69,70,71,72,73], make it a promising area of research. However, the photoredox cycle has predominantly focused on the reductive quenching mechanism. Inspired by those elegant approaches and our continuous interests in NHC catalysis [75,76,77,78] and greener transformation [79,80,81], we now report our discovery in visible light-mediated dual organocatalyzed three-component radical monofluoromethylation/acylation of olefins, in which BIR was generated by oxidative quenching [67,75] of PC* by NHC-acyl adduct. 

## 2. Results and Discussion

We tested the monofluoromethylation/acylation system by employing styrene (**1a**), benzoyl fluoride (**2a**), and H_2_FCSO_2_Na (**3**) as model substrates; NHC-1 as a catalyst; 4-CzIPN as a PC; Cs_2_CO_3_ as a base; and dichloromethane (DCM) as the solvent under blue LED irradiation. To our delight, the desired *β*-monofluoromethylated ketone **4** was separated in 50% yield (Table 1, Entry 1). Switching the PC to an Ir-based photocatalyst could generate **4** in slightly lower yields (Entries 2–4). Screening of NHCs (Entries 5–8) indicates that NHC-2 is the optimal choice (Entry 5). Alternative solvents were tested, and it was found that dichloroethane (DCE, 55%), acetone (61%), toluene (64%), and chloroform (65%) exhibited similar efficiency to DCM, while benzo trifluoride (PhCF_3_, 36%) and tetrahydrofuran (THF, 20%) provided substantially reduced yields (Entries 9–15). Fortunately, by switching the solvent to acetonitrile (CH_3_CN), the desired **4** was isolated in an 89% yield and 12% ee (Entry 14). Further evaluation of bases was carried out, and decreased yields were obtained for **4** (Entries 16–17). Because of the challenges originated from chiral induction of radical–radical cross-coupling (RRCC), no satisfied enantioselectivity was observed (see Figure S1). Employment of racemic NHC-2 has no apparent effect on the reaction efficiency, and **4** could be separated in 90% yield (Entry 18); thus, these conditions were identified as conditions A for evaluation of the substrate scope.

**Scheme 2:**
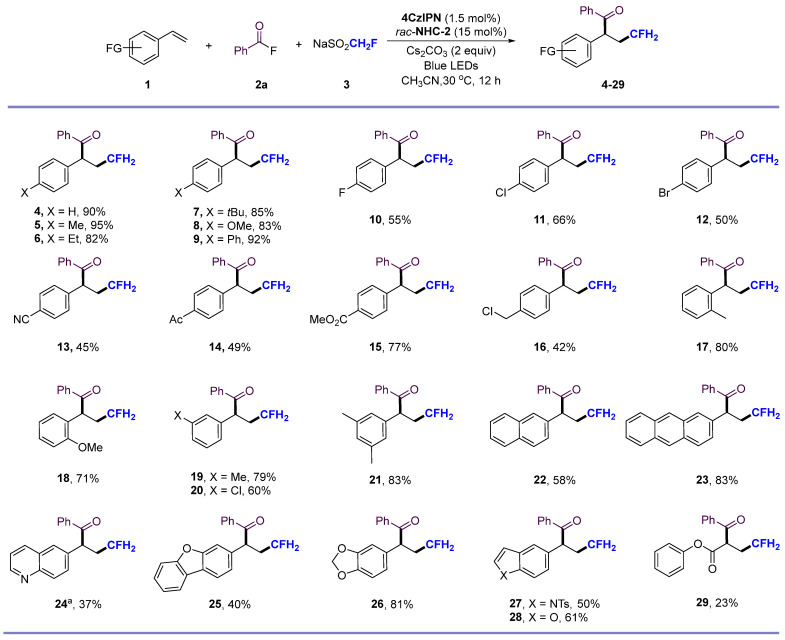
Substrate scope for monofluoromethylation/acylation of olefins. Reaction conditions: unless otherwise noted, all the reactions were carried out with **1** (0.1 mmol), **2a** (0.3 mmol), **3** (0.3 mmol), *rac*-**NHC-2** (0.015 mmol), **PC-3** (0.0015 mmol), and Cs_2_CO_3_ (0.2 mmol) in CH_3_CN (2 mL) under N_2_, 30 °C, and irradiation with blue LED (453.5 nm, 10 W) for 12 h. Isolated yield. ^a^ Acyl fluoride (0.5 mmol) for 18 h.

**Scheme 3:**
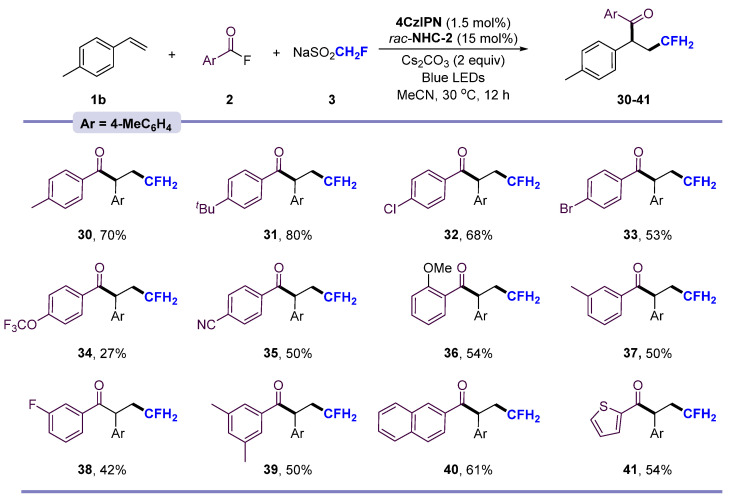
Substrate scope for monofluoromethylation/acylation of olefins. Reaction conditions: unless otherwise noted, all the reactions were carried out with **1b** (0.1 mmol), **2** (0.3 mmol), **3** (0.3 mmol), *rac*-NHC-2 (0.015 mmol), PC-3 (0.0015 mmol), and Cs_2_CO_3_ (0.2 mmol) in CH_3_CN (2 mL) under N_2_, 30 °C, and irradiation with blue LED (453.5 nm, 10 W) for 12 h. Isolated yield.

With the optimized conditions in hand, the application scope and limitations of the olefins were evaluated by employing benzoyl fluoride (**2a**) as an acylating reagent and H_2_FCSO_2_Na as a CH_2_F radical precursor, and the results are summarized in Scheme 2. Aryl olefins bearing electron-donating (alkyl, alkoxy, phenyl), halogen (F, Cl, Br), and electron-withdrawing groups (cyano, carbonyl, and ester carbonyl) at the *para*-position of aryl rings were well tolerated, affording monofluoromethylation/acylation products **4**–**16** in 46–95% yields. Aryl alkenes with electron-donating groups exhibit extremely high reactivity (82–95% yields), while halogen (50–66%) and electron-withdrawing groups (45–77%) deliver targets with moderate yields. Moreover, the benzyl chloride motif was also tolerated in our catalytic system, as identified by the formation of **16** (42%). *Ortho*- and *meta*-substituted aryl alkenes proved to be valuable substrates, delivering **17**–**20** in 60–80% yields. High reactivity (**17**, 80%; **18**, 71%) was observed for ortho-substituted aryl olefins, indicating excellent compatibility with sterically hindered olefins. Di-substituted aryl olefins were well tolerated, generating **21** in 83% yield. Fused carbon ring (naphthalene, anthracene)- and fused heterocyclic (quinoline, dibenzo[*b*,*d*]furan, 1,3-benzodioxole, indole, benzofuran)-substituted olefins were also suitable for this transformation, delivering the desired ketones **22**–**28** in 37–83% yields. Michael alkenes could deliver monofluoromethylation/acylation product **29** in 23% yield. Various non-activated aliphatic olefins, including terminal, internal, and cyclo-olefins, were tested but did not work at all, which might be due to hindrance by a polar mismatch of nucleophilic H2FC radical and aliphatic olefin. Additionally, substrate scope regarding aroyl fluoride was subsequently investigated under the established photocatalytic system (Scheme 3). In general, the reactions proceed smoothly with various substituted aroyl fluorides **2**, 4-methylstyrene (**1b**), and H_2_FCSO_2_Na. Para-substituted aroyl fluorides were first investigated, and electron-rich (**30**, **31**) and halogen-substituted (**32**, **33**) aroyl fluorides reacted well, with desired products being obtained in 53–80% yields. Electron-withdrawing groups (CN, OCF_3_) could also be tolerated, albeit with low reactivity compared to electron-rich ones; for strong electron-withdrawing trifluoromethoxy substituted **34**, only a 27% yield was observed. *Ortho*- (**36**, 54%), *meta*- (**37**, 50%; **38**, 42%) substituted, and 3,5-disubstituted (**39**, 50%) aroyl fluorides were applicable, indicating tolerance to steric effect. Finally, the aroyl fluorides could be extended to naphthyl or thiophene ones, delivering **40** and **41** in 61% and 54% yields, respectively. Broad substrate scope and good functional group tolerance were observed for this metal-free monofluoromethylation/acylation system.

## 3. Mechanistic Investigation

**Scheme 4:**
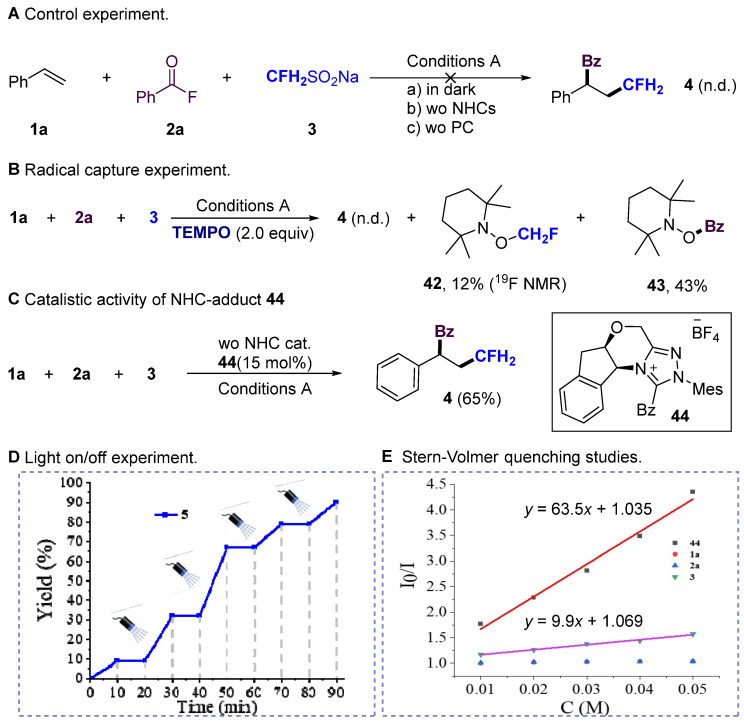
Mechanistic investigation.

Preliminary mechanistic investigations were carried out to gain insight into this transformation (Scheme 4). The control experiment indicates that the NHCs, PC, and LED irradiation were indispensable for the formation of **4** (Scheme 4A). The addition of the radical scavenger 2,2,6,6-tetramethyl-1-piperidinyloxy (TEMPO; 2.0 equiv) could completely inhibit the formation of **4**; CH_2_F-trapped byproduct **42** (12%) and benzoyl-trapped byproduct (**43**, 43%) were observed (Scheme 4B), indicating the possibility of involvement of radical species. Acyl NHC-adduct **44** exhibits good catalytic reactivity, suggesting the intermediacy of **44** (Scheme 4C). Light on/off experiments were carried out employing CD_3_CN as solvents, demonstrating that formation **5** only occurs under blue LED irradiation; thus, the radical chain mechanism seems not preferred (Scheme 4D). Stern–Volmer quenching studies were carried out to gain insight into the photo redox cycle (Scheme 4E). Emission of excited state PC* was preferentially quenched by **44** instead of alkene **1a** or H_2_FCSO_2_Na. Thus, the oxidative quenching progress of PC* by **44** seems more favorable than the reductive quenching cycles. 

**Scheme 5:**
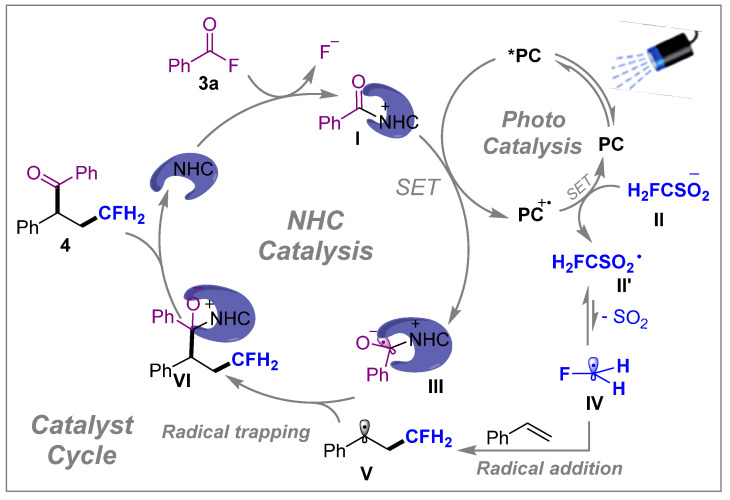
Proposed reaction pathway.

Based on the above mechanistic investigations and previous reports, a plausible reaction mechanism is depicted in Scheme 5. Firstly, benzoyl fluoride 3a undergoes substitution by NHC, generating NHC-adduct **I** (**44**). Oxidative quenching between PC* and **I** might occur, delivering BIR **III** and oxidative state PC^+•^. Single electron oxidation of CH_2_FSO_2_Na by PC^+•^ affords CH_2_F radical via sequential release of SO_2_. Selective addition of CH_2_F radical to double bonds generates benzyl radicals that are trapped by persistent BIR, delivering **VI** and realizing the RA/RACC cascade. Finally, NHC-fragmentation of **VI** could provide target **4** and regenerate the NHC catalyst. The excellent regioselectivity may originate from the persistent radical effect [82]. 

## 4. Materials and Methods

### 4.1. Materials and Instruments

All reactions were conducted under a nitrogen atmosphere. Reagents were sourced from commercial suppliers and utilized without further purification unless stated otherwise. Anhydrous solvents were employed as per distillation procedures. Reaction progress was monitored using thin-layer chromatography (TLC) on 0.25 mm pre-coated silica gel plates. 

^1^H NMR spectra were recorded at 25 °C using either a Bruker 600 or 500, Varian 500 MHz instrument, while ^13^C NMR spectra were obtained at 25 °C using a Bruker 151, Varian 126 MHz instrument, both in CDCl_3_ with TMS as the internal standard. ^19^F NMR spectra were recorded at 25 °C using a Bruker 565 MHz spectrometer. Chemical shifts for ^1^H and ^13^C NMR spectra are reported in parts per million (ppm) relative to the internal standards tetramethylsilane (0 ppm for ^1^H NMR) and CDCl_3_ (77.0 ppm for ^13^C NMR), respectively. ^19^F NMR chemical shifts were determined relative to CFCl_3_ as the external standard; low field was positive.

The designations m, s, d, t, and q represent multiplet, singlet, doublet, triplet, and quartet, respectively. High-resolution mass spectra (HRMS) were acquired using a Bruck micrOTOF instrument. 

For photochemical reactions, we employed the RLH-18 8-position Photo Reaction System manufactured by Beijing Rogertech Co. Ltd. based in Beijing, China. This photo reactor is equipped with 8 blue light 40 W LEDs. The energy peak wavelength of these 10 W blue light LEDs is 453.6 nm, with a peak width at half-height of 20.4. The irradiation vessel is a borosilicate glass test tube, with LED irradiation passing through a high-reflection channel to the test tube. The path length is 2 cm, and there is no filter between the LED and the test tube.

### 4.2. General Procedure for the Synthesis of ***4***

In a nitrogen-filled glove box, a vial equipped with a magnetic stir bar was charged with *rac*-**NHC-2** (6.3 mg, 0.015 mmol), Cs_2_CO_3_ (65.2 mg, 0.2 mmol), **PC-1** (1.2 mg, 0.0015 mmol), **3** (36.0 mg, 0.3 mmol) and CH_3_CN (2.0 mL). Then **1a** (0.1 mmol) and sulfinate **2a** (0.3 mmol) were added. The vial was removed from the glovebox, and then the reaction mixture was irradiated with blue LED at 30 °C for 12 h. After that, the residue was purified by flash column chromatography (petroleum ether/ethyl acetate = 100:1) to give the corresponding product **4**.

*4-fluoro-1,2-diphenylbutan-1-one* (**4**) Purification by column chromatography on silica- gel (petroleum ether/ethyl acetate = 100:1, *v*/*v*) affords the title compound as a yellow oil (yield 22.3 mg, 92%). ^1^H NMR (500 MHz, Chloroform-d) *δ* 7.99–7.92 (m, 2H), 7.51–7.46 (m, 1H), 7.39 (d, *J* = 7.9 Hz, 2H), 7.34–7.29 (m, 4H), 7.26–7.18 (m, 1H), 4.84 (t, *J* = 7.4 Hz, 1H), 4.57–4.29 (m, 2H), 2.74–2.46 (m, 1H), 2.34–2.08 (m, 1H). ^13^C NMR (151 MHz, Chloroform-d) *δ* 199.07, 138.49, 136.46, 133.01, 129.14, 128.79, 128.54, 128.35, 127.35, 81.76 (d, *J* = 164.1 Hz), 49.02 (d, *J* = 3.1 Hz), 34.41 (d, *J* = 19.2 Hz). ^19^F NMR (565 MHz, Chloroform-d) *δ* −221.37 (tdd, *J* = 45.20, 28.25, 22.60 Hz, 1F). HRMS (ESI) (*m*/*z*): calcd for C_16_H_15_FNaO ([M + Na]^+^), 265.0999; found, 265.1003.
*4-fluoro-1-phenyl-2-(p-tolyl)butan-1-one* (**5**) Purification by column chromatography on silica gel (petroleum ether/ethyl acetate = 100:1, *v*/*v*) affords the title compound as a yellow oil (yield 24.4 mg, 92%). ^1^H NMR (500 MHz, Chloroform-d) *δ* 8.13–7.84 (m, 2H), 7.49–7.44 (m, 1H), 7.37 (t, *J* = 7.7 Hz, 2H), 7.22–7.14 (m, 2H), 7.10 (d, *J* = 7.9 Hz, 2H), 4.80 (t, *J* = 7.4 Hz, 1H), 4.55–4.27 (m, 2H), 2.62–2.50 (m, 1H), 2.28 (s, 3H), 2.24–2.08 (m, 1H). ^13^C NMR (151 MHz, Chloroform-d) *δ* 199.20, 137.03, 136.50, 135.42, 132.94, 129.84, 128.78, 128.51, 128.21, 81.81 (d, *J* = 163.7 Hz), 48.62 (d, *J* = 3.1 Hz), 34.37 (d, *J* = 19.5 Hz), 21.01. ^19^F NMR (565 MHz, Chloroform-d) *δ* -221.34 (tdd, *J* = 45.20, 28.25, 22.60 Hz, 1F). HRMS (ESI) (*m*/*z*): calcd for C_17_H_17_FNaO ([M + Na]^+^), 279.1156; found, 279.1162.
*2-(4-ethylphenyl)-4-fluoro-1-phenylbutan-1-one* (**6**) Purification by column chromatography on silica gel (petroleum ether/ethyl acetate = 100:1, *v*/*v*) affords the title compound as a yellow oil (yield 21.8 mg, 82%). ^1^H NMR (500 MHz, Chloroform-d) *δ* 7.97 (dd, *J* = 8.1, 1.4 Hz, 2H), 7.52–7.44 (m, 1H), 7.38 (t, *J* = 7.7 Hz, 2H), 7.22 (d, *J* = 7.9 Hz, 2H), 7.13 (d, *J* = 7.8 Hz, 2H), 4.82 (t, *J* = 7.4 Hz, 1H), 4.54–4.31 (m, 2H), 2.58 (q, *J* = 7.6 Hz, 3H), 2.30–2.10 (m, 1H), 1.19 (t, *J* = 7.7 Hz, 3H). ^13^C NMR (151 MHz, Chloroform-d) *δ* 199.22, 143.31, 136.51, 135.58, 132.95, 128.81, 128.62, 128.52, 128.25, 81.85 (d, *J* = 163.8 Hz), 48.59 (d, *J* = 3.2 Hz), 34.42 (d, *J* = 19.2 Hz), 28.39, 15.31. ^19^F NMR (565 MHz, Chloroform-d) *δ* −221.37 (tdd, *J* = 45.20, 28.25, 22.60 Hz, 1F). HRMS (ESI) (*m*/*z*): calcd for C_18_H_19_FNaO ([M + Na]^+^), 293.1312; found, 293.1307.
*2-(4-(tert-butyl)phenyl)-4-fluoro-1-phenylbutan-1-one* (**7**) Purification by column chromatography on silica gel (petroleum ether/ethyl acetate = 100:1, *v*/*v*) affords the title compound as a yellow oil (yield 25.2 mg, 85%). ^1^H NMR (500 MHz, Chloroform-d) *δ* 8.02–7.96 (m, 2H), 7.51–7.46 (m, 1H), 7.43–7.35 (m, 2H), 7.33–7.28 (m, 2H), 7.24–7.21 (m, 2H), 4.82 (t, *J* = 7.4 Hz, 1H), 4.52–4.29 (m, 2H), 2.63–2.48 (m, 1H), 2.24–2.09 (m, 1H), 1.26 (s, 9H). ^13^C NMR (151 MHz, Chloroform-d) *δ* 199.25, 150.19, 136.57, 135.22, 132.95, 128.83, 128.52, 127.93, 126.02, 81.88 (d, *J* = 163.7 Hz), 48.40 (d, *J* = 3.5 Hz), 34.52, 34.41 (d, *J* = 7.0 Hz), 31.27. ^19^F NMR (565 MHz, Chloroform-d) *δ* -221.32 (tdd, *J* = 45.20, 28.25, 22.60 Hz, 1F). HRMS (ESI) (*m*/*z*): calcd for C_20_H_23_FNaO ([M + Na]^+^), 321.1625; found, 321.1625.
*4-fluoro-2-(4-methoxyphenyl)-1-phenylbutan-1-one* (**8**) Purification by column chromatography on silica gel (petroleum ether/ethyl acetate = 100:1, *v*/*v*) affords the title compound as a yellow oil (yield 22.6 mg, 83%). ^1^H NMR (500 MHz, Chloroform-d) *δ* 8.03–7.89 (m, 2H), 7.51–7.46 (m, 1H), 7.39 (t, *J* = 7.7 Hz, 2H), 7.34–7.29 (m, 4H), 4.86 (t, *J* = 7.3 Hz, 1H), 4.52 (s, 3H), 4.51–4.26 (m, 2H), 2.65–2.49 (m, 1H), 2.26–2.08 (m, 1H). ^13^C NMR (151 MHz, Chloroform-d) *δ* 198.86, 138.73, 136.60, 136.32, 133.17, 129.36, 128.77, 128.72, 128.62, 81.65 (d, *J* = 164.1 Hz), 48.60 (d, *J* = 3.3 Hz), 45.73, 34.39 (d, *J* = 19.5 Hz). ^19^F NMR (565 MHz, Chloroform-d) *δ* -221.42 (tdd, *J* = 45.20, 28.25, 22.60 Hz, 1F). HRMS (ESI) (*m*/*z*): calcd for C_17_H_17_FNaO_2_ ([M + Na]^+^), 295.1105; found, 295.1107.
*2-([1,1’-biphenyl]-4-yl)-4-fluoro-1-phenylbutan-1-one* (**9**) Purification by column chromatography on silica gel (petroleum ether/ethyl acetate = 100:1, *v*/*v*) affords the title compound as a yellow oil (yield 28.6 mg, 90%). ^1^H NMR (500 MHz, Chloroform-d) *δ* 8.27–8.12 (m, 2H), 8.06–7.94 (m, 2H), 7.73–7.59 (m, 1H), 7.54–7.46 (m, 3H), 7.39 (dt, *J* = 21.2, 7.8 Hz, 3H), 7.27–7.19 (m, 2H), 7.12 (dd, *J* = 7.9, 2.3 Hz, 1H), 4.90 (t, *J* = 7.3 Hz, 1H), 4.60–4.25 (m, 2H), 2.67–2.40 (m, 1H), 2.36–2.13 (m, 1H). ^13^C NMR (151 MHz, Chloroform-d) *δ* 198.78, 164.92, 151.46, 140.12, 136.31, 133.66, 133.24, 130.16, 130.11, 129.40, 128.80, 128.66, 128.59, 125.86, 121.55, 120.85, 81.62 (d, *J* = 164.2 Hz), 48.65 (d, *J* = 3.3 Hz), 34.48 (d, *J* = 19.2 Hz). ^19^F NMR (565 MHz, Chloroform-d) *δ* -221.44 (tdd, *J* = 45.20, 28.25, 22.60 Hz, 1F). HRMS (ESI) (*m*/*z*): calcd for C_22_H_19_FNaO ([M + Na]^+^), 341.1312; found, 341.1309.
**7. ***4-fluoro-2-(4-fluorophenyl)-1-phenylbutan-1-one* (**10**) Purification by column chromatography on silica gel (petroleum ether/ethyl acetate = 100:1, *v*/*v*) affords the title compound as a yellow oil (yield 14.3 mg, 55%). ^1^H NMR (500 MHz, Chloroform-d) *δ* 8.07–7.88 (m, 2H), 7.53–7.47 (m, 1H), 7.39 (dd, *J* = 8.5, 7.1 Hz, 2H), 7.31–7.24 (m, 2H), 7.03–6.94 (m, 2H), 4.84 (t, *J* = 7.4 Hz, 1H), 4.63–4.20 (m, 2H), 2.65–2.48 (m, 1H), 2.30–2.06 (m, 1H). ^13^C NMR (151 MHz, Chloroform-d) δ 199.04, 162.04 (d, *J* = 246.3 Hz), 161.22, 136.28, 134.14 (d, *J* = 3.5 Hz), 133.18, 129.91 (d, *J* = 8.1 Hz), 128.74, 128.62, 116.06 (d, *J* = 21.4 Hz), 81.65 (d, *J* = 164.2 Hz), 48.08 (d, *J* = 3.1 Hz), 34.42 (d, *J* = 19.3 Hz).^19^F NMR (565 MHz, Chloroform-d) δ −114.93–−115.04 (m, 1F), −221.57 (tdd, *J* = 45.20, 28.25, 22.60 Hz, 1F). HRMS (ESI) (*m*/*z*): calcd for C_16_H_14_F_2_NaO ([M + Na]^+^), 283.0904; found, 283.0900.
*2-(4-chlorophenyl)-4-fluoro-1-phenylbutan-1-one* (**11**) Purification by column chromatography on silica gel (petroleum ether/ethyl acetate = 100:1, *v*/*v*) affords the title compound as a yellow oil (yield 18.2 mg, 66%). ^1^H NMR (500 MHz, Chloroform-d) *δ* 7.95–7.91 (m, 2H), 7.54–7.48 (m, 1H), 7.39 (t, *J* = 7.7 Hz, 2H), 7.31–7.20 (m, 4H), 4.83 (t, *J* = 7.4 Hz, 1H), 4.63–4.22 (m, 2H), 2.64–2.49 (m, 1H), 2.30–2.06 (m, 1H). ^13^C NMR (151 MHz, Chloroform-d) *δ* 198.78, 136.93, 136.19, 133.35, 133.26, 129.70, 129.34, 128.74, 128.65, 81.55 (d, *J* = 164.2 Hz), 48.24 (d, *J* = 3.2 Hz), 34.31 (d, *J* = 19.3 Hz). ^19^F NMR (565 MHz, Chloroform-d) *δ* −221.55 (tdd, *J* = 45.20, 28.25, 22.60 Hz, 1F). HRMS (ESI) (*m*/*z*): calcd for C_16_H_14_ClFNaO ([M + Na]^+^), 299.0609; found, 299.0613.
**9. ***2-(4-bromophenyl)-4-fluoro-1-phenylbutan-1-one* (**12**) Purification by column chromatography on silica gel (petroleum ether/ethyl acetate = 100:1, *v*/*v*) affords the title compound as a yellow oil (yield 16.1 mg, 50%). ^1^H NMR (500 MHz, Chloroform-d) *δ* 7.98–7.88 (m, 2H), 7.50 (t, *J* = 7.5 Hz, 1H), 7.45–7.37 (m, 4H), 7.19 (d, *J* = 8.4 Hz, 2H), 4.82 (t, *J* = 7.4 Hz, 1H), 4.54–4.28 (m, 2H), 2.65–2.42 (m, 1H), 2.23–2.03 (m, 1H). ^13^C NMR (151 MHz, Chloroform-d) *δ* 198.68, 137.46, 136.16, 133.29, 132.30, 130.06, 128.74, 128.67, 121.46, 81.54 (d, *J* = 164.2 Hz), 48.31 (d, *J* = 3.2 Hz), 34.27 (d, *J* = 19.3 Hz). ^19^F NMR (565 MHz, Chloroform-d) *δ* −221.54 (tdd, *J* = 45.20, 28.25, 22.60 Hz, 1F). HRMS (ESI) (*m*/*z*): calcd for C_16_H_15_BrFO ([M + H]^+^), 321.0285; found, 321.0283.
*4-(4-fluoro-1-oxo-1-phenylbutan-2-yl)benzonitrile* (**13**) Purification by column chromatography on silica gel (petroleum ether/ethyl acetate = 100:1, *v*/*v*) affords the title compound as a yellow oil (yield 12.0 mg, 45%). ^1^H NMR (500 MHz, Chloroform-d) *δ* 7.96–7.89 (m, 2H), 7.60 (d, *J* = 8.4 Hz, 2H), 7.55–7.50 (m, 1H), 7.48–7.37 (m, 4H), 4.93 (t, *J* = 7.3 Hz, 1H), 4.58–4.16 (m, 2H), 2.68–2.52 (m, 1H), 2.24–2.08 (m, 1H). ^13^C NMR (151 MHz, Chloroform-d) δ 198.09, 143.85, 135.97, 133.61, 132.89, 129.19, 128.81, 128.71, 118.42, 111.51, 81.76 (d, *J* = 164.2 Hz), 48.84 (d, *J* = 3.2 Hz), 34.32 (d, *J* = 19.3 Hz). ^19^F NMR (565 MHz, Chloroform-d) *δ* −221.48 (tdd, *J* = 45.20, 28.25, 22.60 Hz, 1F). HRMS (ESI) (*m*/*z*): calcd for C_17_H_14_FNNaO ([M + Na]^+^), 290.0952; found, 290.0961.
*2-(4-acetylphenyl)-4-fluoro-1-phenylbutan-1-one* (**14**) Purification by column chromatography on silica gel (petroleum ether/ethyl acetate = 100:1, *v*/*v*) affords the title compound as a yellow oil (yield 13.9 mg, 49%). ^1^H NMR (500 MHz, Chloroform-d) *δ* 7.99–7.94 (m, 2H), 7.92 (d, *J* = 1.9 Hz, 1H), 7.81 (dt, *J* = 7.8, 1.4 Hz, 1H), 7.56–7.48 (m, 2H), 7.40 (td, *J* = 7.6, 5.6 Hz, 3H), 4.94 (t, *J* = 7.4 Hz, 1H), 4.58–4.17 (m, 2H), 2.68–2.59 (m, 1H), 2.57 (s, 3H), 2.26–2.10 (m, 1H). ^13^C NMR (151 MHz, Chloroform-d) *δ* 198.75, 197.74, 139.19, 137.92, 136.18, 133.32, 132.90, 129.43, 128.76, 128.70, 128.11, 127.55, 81.56 (d, *J* = 164.2 Hz), 48.71 (d, *J* = 3.4 Hz), 34.46 (d, *J* = 19.3 Hz), 26.69. ^19^F NMR (565 MHz, Chloroform-d) *δ* −221.30 (tdd, *J* = 45.20, 28.25, 22.60 Hz, 1F). HRMS (ESI) (*m*/*z*): calcd for C_18_H_17_FNaO_2_ ([M + Na]^+^), 307.1104; found, 307.1112.
*methyl-4-(4-fluoro-1-oxo-1-phenylbutan-2-yl)benzoate* (**15**) Purification by column chromatography on silica gel (petroleum ether/ethyl acetate = 100:1, *v*/*v*) affords the title compound as a yellow oil (yield 23.1 mg, 77%). ^1^H NMR (500 MHz, Chloroform-d) *δ* 7.97 (d, *J* = 8.2 Hz, 2H), 7.94 (dd, *J* = 7.0, 5.5 Hz, 2H), 7.52–7.46 (m, 1H), 7.38 (dd, *J* = 8.2, 6.5 Hz, 4H), 4.92 (t, *J* = 7.3 Hz, 1H), 4.61–4.27 (m, 2H), 3.87 (s, 3H), 2.63–2.57 (m, 1H), 2.24–2.10 (m, 1H). ^13^C NMR (151 MHz, Chloroform-d) *δ* 198.99, 169.26, 149.89, 136.33, 135.87, 133.18, 129.34, 128.77, 128.62, 122.19, 81.65 (d, *J* = 164.0 Hz), 48.20 (d, *J* = 3.0 Hz), 34.49 (d, *J* = 19.5 Hz), 21.11. ^19^F NMR (565 MHz, Chloroform-d) *δ* −221.35 (tdd, *J* = 45.20, 28.25, 22.60 Hz, 1F). HRMS (ESI) (*m*/*z*): calcd for C_18_H_17_FNaO_3_ ([M + Na]^+^), 323.1054; found, 323.1053.
*2-(4-(chloromethyl)phenyl)-4-fluoro-1-phenylbutan-1-one* (**16**) Purification by column chromatography on silica gel (petroleum ether/ethyl acetate = 100:1, *v*/*v*) affords the title compound as a yellow oil (yield 12.2 mg, 42%). ^1^H NMR (500 MHz, Chloroform-d) *δ* 7.99–7.90 (m, 2H), 7.54–7.47 (m, 1H), 7.39 (t, *J* = 7.7 Hz, 2H), 7.35–7.28 (m, 4H), 4.86 (t, *J* = 7.3 Hz, 1H), 4.52 (s, 2H), 4.51–4.28 (m, 2H), 2.66–2.49 (m, 1H), 2.27–2.10 (m, 1H). ^13^C NMR (151 MHz, Chloroform-d) *δ* 198.86, 138.73, 136.60, 136.32, 133.17, 129.35, 128.77, 128.71, 128.61, 81.65 (d, *J* = 164.2 Hz), 48.61 (d, *J* = 3.1 Hz), 45.73, 34.38 (d, *J* = 19.2 Hz). ^19^F NMR (565 MHz, Chloroform-d) *δ* −221.43 (tdd, *J* = 45.20, 28.25, 22.60 Hz, 1F). HRMS (ESI) (*m*/*z*): calcd for C_17_H_16_ClFNaO ([M + Na]^+^), 313.0765; found, 313.0763.
*4-fluoro-1-phenyl-2-(o-tolyl)butan-1-one* (**17**) Purification by column chromatography on silica- gel (petroleum ether/ethyl acetate = 100:1, *v*/*v*) affords the title compound as a yellow oil (yield 20.5 mg, 80%). ^1^H NMR (500 MHz, Chloroform-d) *δ* 7.85–7.82 (m, 2H), 7.46 (t, *J* = 7.4 Hz, 1H), 7.36 (t, *J* = 7.7 Hz, 2H), 7.21 (d, *J* = 7.3 Hz, 1H), 7.14–7.04 (m, 3H), 4.97 (dd, *J* = 8.1, 6.1 Hz, 1H), 4.63–4.30 (m, 2H), 2.67–2.56 (m, 1H), 2.54 (s, 3H), 2.23–1.97 (m, 1H). ^13^C NMR (151 MHz, Chloroform-d) *δ* 199.84, 137.38, 136.82, 135.46, 132.91, 131.22, 128.55, 128.45, 127.29 (d, *J* = 3.5 Hz), 126.82, 81.87 (d, *J* = 164.5 Hz), 45.32 (d, *J* = 2.9 Hz), 34.12(d, *J* = 19.6 Hz), 19.59. ^19^F NMR (565 MHz, Chloroform-d) *δ* −219.78 (tt, *J* = 45.20, 22.60 Hz, 1F). HRMS (ESI) (*m*/*z*): calcd for C_17_H_17_FNaO ([M + Na]^+^), 279.1155; found, 279.1159.
*4-fluoro-2-(2-methoxyphenyl)-1-phenylbutan-1-one* (**18**) Purification by column chromatography on silica gel (petroleum ether/ethyl acetate = 100:1, *v*/*v*) affords the title compound as a yellow oil (yield 19.3 mg, 71%). ^1^H NMR (500 MHz, Chloroform-d) *δ* 8.06–7.89 (m, 2H), 7.44 (t, *J* = 7.4 Hz, 1H), 7.34 (t, *J* = 7.8 Hz, 2H), 7.18 (ddd, *J* = 8.2, 7.4, 1.7 Hz, 1H), 7.11 (dd, *J* = 7.6, 1.7 Hz, 1H), 6.94–6.82 (m, 2H), 5.25 (t, *J* = 7.2 Hz, 1H), 4.54–4.34 (m, 2H), 3.90 (s, 3H), 2.72–2.47 (m, 1H), 2.19–2.03 (m, 1H). ^13^C NMR (151 MHz, Chloroform-d) *δ* 199.80, 156.22, 136.46, 132.80, 128.57, 128.51 128.49, 128.35, 127.41, 111.09, 82.22 (d, *J* = 164.1 Hz), 55.62, 41.48 (d, *J* = 4.5 Hz), 33.40 (d, *J* = 19.9 Hz). ^19^F NMR (565 MHz, Chloroform-d) *δ* −219.92 (tt, *J* = 45.20, 22.60 Hz, 1F). HRMS (ESI) (*m*/*z*): calcd for C_17_H_17_FNaO_2_ ([M + Na]^+^), 295.1104; found, 295.1104.
*4-fluoro-1-phenyl-2-(m-tolyl)butan-1-one* (**19**) Purification by column chromatography on silica- gel (petroleum ether/ethyl acetate = 100:1, *v*/*v*) affords the title compound as a yellow oil (yield 20.2 mg, 79%). ^1^H NMR (500 MHz, Chloroform-d) *δ* 8.02–7.93 (m, 2H), 7.54–7.43 (m, 1H), 7.38 (dd, *J* = 8.4, 7.0 Hz, 2H), 7.19 (td, *J* = 7.4, 1.2 Hz, 1H), 7.11 (d, *J* = 7.7 Hz, 2H), 7.02 (d, *J* = 7.6 Hz, 1H), 4.80 (t, *J* = 7.3 Hz, 1H), 4.55–4.28 (m, 2H), 2.64–2.48 (m, 1H), 2.30 (s, 3H), 2.23–2.09 (m, 1H). ^13^C NMR (151 MHz, Chloroform-d) *δ* 199.17, 138.86, 138.40, 136.52, 133.00, 128.99, 128.84, 128.82, 128.54, 128.16, 125.53, 81.69 (d, *J* = 165.1 Hz), 48.96 (d, *J* = 3.1 Hz), 34.45 (d, *J* = 19.3 Hz), 21.42. ^19^F NMR (565 MHz, Chloroform-d) *δ* −221.30 (tdd, *J* = 45.20, 28.25, 22.60 Hz, 1F). HRMS (ESI) (*m*/*z*): calcd for C_17_H_17_FNaO ([M + Na]^+^), 279.1155; found, 279.1147.
*2-(3-chlorophenyl)-4-fluoro-1-phenylbutan-1-one* (**20**). Purification by column chromatography on silica gel (petroleum ether/ethyl acetate = 100:1, *v*/*v*) affords the title compound as a yellow oil (yield 16.6 mg, 60%). ^1^H NMR (500 MHz, Chloroform-d) *δ* 7.96 (d, *J* = 6.9 Hz, 2H), 7.56–7.49 (m, 1H), 7.41 (t, *J* = 7.7 Hz, 2H), 7.32 (d, *J* = 1.8 Hz, 1H), 7.28–7.18 (m, 3H), 4.84 (t, *J* = 7.4 Hz, 1H), 4.61–4.22 (m, 2H), 2.69–2.49 (m, 1H), 2.34–2.07 (m, 1H). ^13^C NMR (151 MHz, Chloroform-d) *δ* 198.52, 140.45, 136.18, 134.94, 133.31, 130.36, 128.76, 128.69, 128.39, 127.68, 126.60, 81.56 (d, *J* = 164.3 Hz), 80.97, 48.50 (d, *J* = 3.0 Hz), 34.39 (d, *J* = 19.5 Hz). ^19^F NMR (565 MHz, Chloroform-d) *δ* −221.47 (tdd, *J* = 45.20, 28.25, 22.60 Hz, 1F). HRMS (ESI) (*m*/*z*): calcd for C_16_H_14_ClFNaO ([M + Na]^+^), 299.0609; found, 299.0602.
*2-(3,5-dimethylphenyl)-4-fluoro-1-phenylbutan-1-1* (**21**) Purification by column chromatography on silica gel (petroleum ether/ethyl acetate = 100:1, *v*/*v*) affords the title compound as a yellow oil (yield 22.4 mg, 83%).^1^H NMR (500 MHz, Chloroform-d) *δ* 7.97 (dd, *J* = 8.4, 1.4 Hz, 2H), 7.52–7.46 (m, 1H), 7.38 (dd, *J* = 8.4, 7.1 Hz, 2H), 6.91 (s, 2H), 6.84 (s, 1H), 4.76 (t, *J* = 7.3 Hz, 1H), 4.57–4.30 (m, 2H), 2.62–2.46 (m, 1H), 2.28–2.22 (m, 6H), 2.20–2.09 (m, 1H). ^13^C NMR (151 MHz, Chloroform-d) *δ* 199.20, 138.65, 138.32, 136.57, 132.94, 129.05, 128.81, 128.51, 126.03, 81.76 (d, *J* = 165.3 Hz), 48.89 (d, *J* = 3.4 Hz), 34.49 (d, *J* = 19.2 Hz), 21.28. ^19^F NMR (565 MHz, Chloroform-d) *δ* −221.23 (tdd, *J* = 45.20, 28.25, 22.60 Hz, 1F). HRMS (ESI) (*m*/*z*): calcd for C_18_H_19_FNaO ([M + Na]^+^), 271.1492; found, 271.1495.
*4-fluoro-2-(naphthalen-2-yl)-1-phenylbutan-1-one* (**22**) Purification by column chromatography on silica gel (petroleum ether/ethyl acetate = 100:1, *v*/*v*) affords the title compound as a yellow oil (yield 17.0 mg, 58%). ^1^H NMR (500 MHz, Chloroform-d) *δ* 8.00 (d, *J* = 7.7 Hz, 2H), 7.82–7.75 (m, 4H), 7.45 (dt, *J* = 8.1, 2.3 Hz, 4H), 7.37 (d, *J* = 7.7 Hz, 2H), 5.01 (t, *J* = 7.3 Hz, 1H), 4.64–4.14 (m, 2H), 2.77–2.51 (m, 1H), 2.45–2.17 (m, 1H). ^13^C NMR (151 MHz, Chloroform-d) *δ* 199.03, 136.43, 135.95, 133.64, 133.06, 132.56, 129.04, 128.83, 128.57, 127.78, 127.65, 127.38, 126.34, 126.11, 126.06, 81.72 (d, *J* = 164.9 Hz), 49.14 (d, *J* = 3.2 Hz), 34.42 (d, *J* = 19.3 Hz). ^19^F NMR (565 MHz, Chloroform-d) *δ* -221.32 (tdd, *J* = 45.20, 28.25, 22.60 Hz, 1F). HRMS (ESI) (*m*/*z*): calcd for C_20_H_17_FNaO ([M + Na]^+^), 315.1155; found, 315.1159.
*2-(anthracen-2-yl)-4-fluoro-1-phenylbutan-1-one* (**23**) Purification by column chromatography on silica gel (petroleum ether/ethyl acetate = 100:1, *v*/*v*) affords the title compound as a yellow oil (yield 28.4 mg, 83%). ^1^H NMR (500 MHz, Chloroform-d) *δ* 8.18 (d, *J* = 7.0 Hz, 2H), 7.99 (d, *J* = 7.7 Hz, 2H), 7.63 (d, *J* = 7.4 Hz, 1H), 7.56–7.49 (m, 3H), 7.40 (dt, *J* = 20.8, 7.8 Hz, 3H), 7.28–7.20 (m, 2H), 7.12 (dd, *J* = 8.1, 2.3 Hz, 1H), 4.90 (t, *J* = 7.4 Hz, 1H), 4.63–4.19 (m, 2H), 2.68–2.50 (m, 1H), 2.28–2.07 (m, 1H). ^13^C NMR (151 MHz, Chloroform-d) *δ* 198.79, 164.93, 151.46, 140.13, 136.31, 133.67, 133.24, 130.17, 130.12, 129.40, 128.81, 128.66, 128.59, 125.87, 121.55, 120.86, 82.17, 81.09, 48.65 (d, *J* = 3.3 Hz), 34.48 (d, *J* = 19.4 Hz). ^19^F NMR (565 MHz, Chloroform-d) *δ* −221.44 (tdd, *J* = 45.20, 28.25, 22.60 Hz, 1F). HRMS (ESI) (*m*/*z*): calcd for C_24_H_19_FNaO ([M + Na]^+^), 365.1312; found, 365.1315.
*4-fluoro-1-phenyl-2-(quinolin-6-yl)butan-1-one* (**24**) Purification by column chromatography on silica gel (petroleum ether/ethyl acetate = 100:1, *v*/*v*) affords the title compound as a yellow oil (yield 10.8 mg, 37%). ^1^H NMR (500 MHz, Chloroform-d) *δ* 8.92–8.83 (m, 1H), 8.08 (dd, *J* = 10.6, 8.4 Hz, 2H), 8.00 (s, 2H), 7.79–7.69 (m, 2H), 7.47 (t, *J* = 7.4 Hz, 1H), 7.40–7.35 (m, 3H), 5.06 (t, *J* = 7.3 Hz, 1H), 4.60–4.28 (m, 2H), 2.74–2.58 (m, 1H), 2.33–2.20 (m, 1H). ^13^C NMR (151 MHz, Chloroform-d) *δ* 198.81, 150.57, 147.51, 136.88, 136.28, 135.92, 133.26, 130.53, 129.88, 128.79, 128.66, 121.48, 81.61 (d, *J* = 164.4 Hz), 48.83 (d, *J* = 3.2 Hz), 34.50 (d, *J* = 19.5 Hz). ^19^F NMR (565 MHz, Chloroform-d) *δ* −221.36 (tdd, *J* = 45.20, 28.25, 22.60 Hz, 1F). HRMS (ESI) (*m*/*z*): calcd for C_19_H_16_FNNaO ([M + Na]^+^), 316.1108; found, 316.1113.
*2-(5a,9a-dihydrodibenzo[b,d]furan-3-yl)-4-fluoro-1-phenylbutan-1-one* (**25**) Purification- by column chromatography on silica gel (petroleum ether/ethyl acetate = 100:1, *v*/*v*) affords the title compound as a yellow oil (yield 13.3 mg, 40%). ^1^H NMR (500 MHz, Chloroform-d) *δ* 8.50 (d, *J* = 1.7 Hz, 1H), 8.01 (dd, *J* = 8.7, 1.8 Hz, 1H), 7.90 (d, *J* = 8.1 Hz, 1H), 7.81 (dd, *J* = 8.5, 3.5 Hz, 2H), 7.58–7.53 (m, 2H), 7.53–7.48 (m, 2H), 7.30 (t, *J* = 7.6 Hz, 2H), 7.20 (t, *J* = 7.4 Hz, 1H), 5.01 (t, *J* = 7.3 Hz, 1H), 4.60–4.31 (m, 2H), 2.73–2.56 (m, 1H), 2.31–2.15 (m, 1H). ^13^C NMR (151 MHz, Chloroform-d) *δ* 199.05, 138.60, 135.49, 133.79, 132.44, 130.60, 129.67, 129.17, 128.51, 128.39, 128.35, 127.66, 127.36, 126.69, 124.43, 81.83 (d, *J* = 163.7 Hz), 49.07 (d, *J* = 3.3 Hz), 34.46 (d, *J* = 19.2 Hz). ^19^F NMR (565 MHz, Chloroform-d) *δ* −221.34 (tdd, *J* = 50.85, 33.90, 28.25 Hz, 1F). HRMS (ESI) (*m*/*z*): calcd for C_22_H_17_FNaO_2_ ([M + Na]^+^), 355.1104; found, 355.1104.
*2-(benzo[d][1,3]dioxol-5-yl)-4-fluoro-1-phenylbutan-1-one* (**26**) Purification by column chromatography on silica gel (petroleum ether/ethyl acetate = 100:1, *v*/*v*) affords the title compound as a yellow oil (yield 23.2 mg, 81%). ^1^H NMR (500 MHz, Chloroform-d) δ 8.04–7.84 (m, 2H), 7.52–7.46 (m, 1H), 7.40 (d, *J* = 7.7 Hz, 2H), 6.77 (d, *J* = 7.4 Hz, 2H), 6.73 (d, *J* = 8.0 Hz, 1H), 5.92–5.87 (m, 2H), 4.75 (t, *J* = 7.4 Hz, 1H), 4.56–4.28 (m, 2H), 2.60–2.45 (m, 1H), 2.19–2.01 (m, 1H). ^13^C NMR (151 MHz, Chloroform-d) *δ* 199.02, 148.23, 146.89, 136.39, 133.04, 132.04, 128.76, 128.56, 121.87, 108.79, 108.45, 101.14, 81.73 (d, *J* = 165.1 Hz), 48.51 (d, *J* = 3.1 Hz), 34.34 (d, *J* = 19.2 Hz), 29.71. ^19^F NMR (565 MHz, Chloroform-d) *δ* −221.48 (tdd, *J* = 50.85, 33.90, 22.60 Hz, 1F). HRMS (ESI) (*m*/*z*): calcd for C_17_H_15_FNaO_3_ ([M + Na]^+^), 309.0897; found, 309.0896.
*4-fluoro-1-phenyl-2-(1-tosyl-1H-indol-5-yl)butan-1-one* (**27**) Purification by column chromatography on silica gel (petroleum ether/ethyl acetate = 100:1, *v*/*v*) affords the title compound as a yellow oil (yield 21.7 mg, 50%). ^1^H NMR (500 MHz, Chloroform-d) *δ* 7.95 (dd, *J* = 8.4, 1.4 Hz, 2H), 7.90 (d, *J* = 8.6 Hz, 1H), 7.80–7.72 (m, 2H), 7.52 (d, *J* = 3.7 Hz, 1H), 7.49–7.41 (m, 2H), 7.36 (t, *J* = 7.7 Hz, 2H), 7.29–7.22 (m, 1H), 7.21 (d, *J* = 8.1 Hz, 2H), 6.57 (d, *J* = 3.7 Hz, 1H), 4.91 (t, *J* = 7.4 Hz, 1H), 4.54–4.23 (m, 2H), 2.65–2.50 (m, 1H), 2.32 (s, 3H), 2.23–2.07 (m, 1H). ^13^C NMR (151 MHz, Chloroform-d) *δ* 199.17, 145.02, 136.41, 135.32, 133.98, 133.41, 133.04, 131.34, 129.94, 128.81, 128.55, 126.86, 126.85, 124.94, 121.04, 114.05, 108.68, 81.59 (d, *J* = 164.1 Hz), 48.61 (d, *J* = 3.3 Hz), 34.72 (d, *J* = 19.3 Hz), 21.56. ^19^F NMR (565 MHz, Chloroform-d) *δ* −221.38 (tdd, *J* = 45.20, 28.25, 22.60 Hz, 1F). HRMS (ESI) (*m*/*z*): calcd for C_25_H_22_FNNaO_3_S ([M + Na]^+^), 458.1196; found, 458.1198.
*2-(benzofuran-5-yl)-4-fluoro-1-phenylbutan-1-one* (**28**) Purification by column chromatography on silica gel (petroleum ether/ethyl acetate = 100:1, *v*/*v*) affords the title compound as a yellow oil (yield 51.9 mg, 62%). ^1^H NMR (500 MHz, Chloroform-d) *δ* 7.99 (dd, *J* = 8.4, 1.4 Hz, 2H), 7.70 (dd, *J* = 7.5, 1.6 Hz, 1H), 7.54 (s, 1H), 7.53–7.48 (m, 1H), 7.45 (dd, *J* = 7.6, 1.2 Hz, 1H), 7.39 (t, *J* = 7.6 Hz, 2H), 7.33–7.25 (m, 2H), 5.08 (t, *J* = 7.3 Hz, 1H), 4.68–4.31 (m, 2H), 2.72–2.55 (m, 1H), 2.42–2.24 (m, 1H).^13^C NMR (151 MHz, Chloroform-d) *δ* 198.49, 155.52, 142.98, 136.06, 133.33, 128.67, 128.58, 126.48, 124.75, 122.95, 119.93, 117.81, 111.73, 81.69 (d, *J* = 164.2 Hz), 38.70 (d, *J* = 3.3 Hz), 32.95 (d, *J* = 19.4 Hz). ^19^F NMR (565 MHz, Chloroform-d) *δ* −221.32 (tdd, *J* = 45.20, 28.25, 22.60 Hz, 1F). HRMS (ESI) (*m*/*z*): calcd for C_18_H_15_FNaO_2_ ([M + Na]^+^), 305.0948; found, 305.0956.
*phenyl-2-benzoyl-4-fluorobutanoate* (**29**) Purification by column chromatography on silica gel (petroleum ether/ethyl acetate = 100:1, *v*/*v*) affords the title compound as a yellow oil (yield 6.5 mg, 23%). ^1^H NMR (500 MHz, Chloroform-d) *δ* 8.12–8.04 (m, 1H), 7.64 (td, *J* = 7.2, 1.3 Hz, 0H), 7.53 (t, *J* = 7.8 Hz, 1H), 7.38–7.29 (m, 1H), 7.24–7.17 (m, 1H), 6.98 (dd, *J* = 8.5, 1.3 Hz, 1H), 4.85 (dd, *J* = 7.8, 6.2 Hz, 0H), 4.75–4.54 (m, 1H), 2.60–2.41 (m, 1H).^13^C NMR (151 MHz, Chloroform-d) *δ* 193.34, 167.18, 149.34, 134.64, 132.98, 128.42, 127.94, 127.79, 125.15, 120.16, 80.58 (d, *J* = 165.6 Hz), 48.89 (d, *J* = 2.9 Hz), 28.86 (d, *J* = 19.6 Hz).^19^F NMR (565 MHz, Chloroform-d) *δ* −220.48 (tdd, *J* = 45.20, 28.25, 22.60 Hz, 1F). HRMS (ESI) (*m*/*z*): calcd for C_17_H_15_FNaO_3_ ([M + Na]^+^), 309.0897; found, 309.0891.
*4-fluoro-1,2-di-p-tolylbutan-1-one* (**30**) Purification by column chromatography on silica gel (petroleum ether/ethyl acetate = 100:1, *v*/*v*) affords the title compound as a yellow oil (yield 18.9 mg, 70%). ^1^H NMR (500 MHz, Chloroform-d) *δ* 7.86 (d, *J* = 8.4 Hz, 2H), 7.21–7.12 (m, 4H), 7.09 (d, *J* = 7.9 Hz, 2H), 4.78 (t, *J* = 7.4 Hz, 1H), 4.53–4.28 (m, 2H), 2.62–2.46 (m, 1H), 2.33 (s, 3H), 2.27 (s, 3H), 2.23–2.06 (m, 1H). ^13^C NMR (151 MHz, Chloroform-d) *δ* 198.80, 143.77, 136.92, 135.67, 133.95, 129.79, 129.21, 128.92, 128.17, 81.89 (d, *J* = 163.8 Hz), 48.44 (d, *J* = 3.2 Hz), 34.36 (d, *J* = 19.3 Hz), 21.58, 21.01. ^19^F NMR (565 MHz, Chloroform-d) *δ* −221.31 (tdd, *J* = 50.85, 33.90, 28.25 Hz, 1F). HRMS (ESI) (*m*/*z*): calcd for C_18_H_19_FNaO ([M + Na]^+^), 293.1312; found, 293.1315.
*1-(4-(tert-butyl)phenyl)-4-fluoro-2-(p-tolyl)butan-1-one* (**31**) Purification by column chromatography on silica gel (petroleum ether/ethyl acetate = 100:1, *v*/*v*) affords the title compound as a yellow oil (yield 24.9 mg, 80%). ^1^H NMR (500 MHz, Chloroform-d) *δ* 8.09–7.79 (m, 2H), 7.39 (d, *J* = 8.5 Hz, 2H), 7.20 (d, *J* = 8.0 Hz, 2H), 7.11 (d, *J* = 8.0 Hz, 2H), 4.80 (t, *J* = 7.4 Hz, 1H), 4.57–4.25 (m, 2H), 2.60–2.48 (m, 1H), 2.28 (s, 3H), 2.23–2.06 (m, 1H), 1.28 (s, 9H). ^13^C NMR (151 MHz, Chloroform-d) *δ* 198.72, 156.68, 136.93, 135.69, 133.86, 129.80, 128.77, 128.20, 125.50, 81.89 (d, *J* = 163.8 Hz), 48.44 (d, *J* = 3.3 Hz), 35.06, 34.47 (d, *J* = 19.3 Hz), 31.02, 21.02. ^19^F NMR (565 MHz, Chloroform-d) *δ* −221.28 (tdd, *J* = 45.20, 28.25, 22.60 Hz, 1F). HRMS (ESI) (*m*/*z*): calcd for C_21_H_25_FNaO ([M + Na]^+^), 335.1781; found, 335.1786.
*1-(4-chlorophenyl)-4-fluoro-2-(p-tolyl)butan-1-*one (**32**) Purification by column chromatography on silica gel (petroleum ether/ethyl acetate = 100:1, *v*/*v*) affords the title compound as a yellow oil (yield 15.4 mg, 68%). ^1^H NMR (500 MHz, Chloroform-d) *δ* 7.88 (d, *J* = 8.7 Hz, 2H), 7.34 (d, *J* = 8.6 Hz, 2H), 7.15 (d, *J* = 8.1 Hz, 2H), 7.11 (d, *J* = 7.9 Hz, 2H), 4.73 (t, *J* = 7.3 Hz, 1H), 4.62–4.22 (m, 2H), 2.62–2.38 (m, 1H), 2.28 (s, 3H), 2.23–2.04 (m, 1H). ^13^C NMR (151 MHz, Chloroform-d) *δ* 197.96, 139.38, 137.25, 135.09, 134.73, 130.20, 129.96, 128.84, 128.15, 81.69 (d, *J* = 163.9 Hz), 48.74 (d, *J* = 3.3 Hz), 34.23 (d, *J* = 19.2 Hz), 21.03. ^19^F NMR (565 MHz, Chloroform-d) *δ* −221.51 (tdd, *J* = 45.20, 28.25, 22.60 Hz, 1F). HRMS (ESI) (*m*/*z*): calcd for C_17_H_16_ClFNaO ([M + Na]^+^), 313.0765; found, 313.0765.
*1-(4-bromophenyl)-4-fluoro-2-(p-tolyl)butan-1-one* (**33**) Purification by column chromatography on silica gel (petroleum ether/ethyl acetate = 100:1, *v*/*v*) affords the title compound as a yellow oil (yield 17.7 mg, 53%). ^1^H NMR (500 MHz, Chloroform-d) *δ* 7.80 (d, *J* = 8.4 Hz, 2H), 7.50 (d, *J* = 8.3 Hz, 2H), 7.14 (d, *J* = 7.8 Hz, 2H), 7.10 (d, *J* = 7.9 Hz, 2H), 4.72 (t, *J* = 7.3 Hz, 1H), 4.53–4.24 (m, 2H), 2.62–2.46 (m, 1H), 2.28 (s, 3H), 2.20–2.04 (m, 1H). ^13^C NMR (151 MHz, Chloroform-d) *δ* 198.15, 137.26, 135.15, 135.06, 131.83, 130.30, 129.96, 128.14, 81.67 (d, *J* = 163.7 Hz), 48.74 (d, *J* = 3.2 Hz), 34.21 (d, *J* = 19.5 Hz), 21.01. ^19^F NMR (565 MHz, Chloroform-d) *δ* −221.52 (tdd, *J* = 50.85, 33.90, 28.25 Hz, 1F). HRMS (ESI) (*m*/*z*): calcd for C_17_H_16_BrFNaO ([M + Na]^+^), 357.0260; found, 357.0265.
*4-fluoro-2-(p-tolyl)-1-(4-(trifluoromethoxy)phenyl)butan-1-one* (**34**) Purification by column chromatography on silica gel (petroleum ether/ethyl acetate = 100:1, *v*/*v*) affords the title compound as a yellow oil (yield 9.2 mg, 27%). ^1^H NMR (500 MHz, Chloroform-d) *δ* 7.80 (d, *J* = 8.4 Hz, 2H), 7.50 (d, *J* = 8.3 Hz, 2H), 7.14 (d, *J* = 7.8 Hz, 2H), 7.10 (d, *J* = 7.9 Hz, 2H), 4.72 (t, *J* = 7.3 Hz, 1H), 4.53–4.24 (m, 2H), 2.62–2.46 (m, 1H), 2.28 (s, 3H), 2.20–2.04 (m, 1H). ^13^C NMR (151 MHz, Chloroform-d) *δ* 198.80, 137.05, 136.18, 133.34, 129.73, 128.75, 128.70, 121.54, 120.04 (d, *J* = 259.2 Hz), 81.52 (d, *J* = 164.3 Hz), 48.07 (d, *J* = 2.9 Hz), 34.47 (d, *J* = 19.2 Hz), 21.02. ^19^F NMR (565 MHz, Chloroform-d) *δ* −57.88 (s, 3F), −221.36 (tdd, *J* = 50.85, 33.90, 28.25 Hz, 1F). HRMS (ESI) (*m*/*z*): calcd for C_18_H_16_F_4_NaO_2_ ([M + Na]^+^), 363.0978; found, 363.0971.
*4-(4-fluoro-2-(p-tolyl)butanoyl)benzonitrile* (**35**) Purification by column chromatography on silica gel (petroleum ether/ethyl acetate = 100:1, *v*/*v*) affords the title compound as a yellow oil (yield 14.0 mg, 50%). ^1^H NMR (500 MHz, Chloroform-d) *δ* 7.73 (d, *J* = 8.2 Hz, 2H), 7.65 (d, *J* = 8.2 Hz, 2H), 7.14 (d, *J* = 7.9 Hz, 2H), 7.10 (d, *J* = 8.0 Hz, 2H), 4.71 (t, *J* = 7.3 Hz, 1H), 4.54–4.29 (m, 2H), 2.62–2.44 (m, 1H), 2.28 (s, 3H), 2.21–2.03 (m, 1H). ^13^C NMR (151 MHz, Chloroform-d) *δ* 198.44, 137.84, 137.26, 135.67, 135.04, 130.19, 129.97, 128.15, 100.98, 81.68 (d, *J* = 163.7 Hz), 48.67 (d, *J* = 3.0 Hz), 34.18 (d, *J* = 19.4 Hz), 21.03. ^19^F NMR (565 MHz, Chloroform-d) *δ* −221.51 (tdd, *J* = 45.20, 28.25, 22.60 Hz, 1F). HRMS (ESI) (*m*/*z*): calcd for C_18_H_16_FNNaO ([M + Na]^+^), 304.1108; found, 304.1105.
*4-fluoro-1-(2-methoxyphenyl)-2-(p-tolyl)butan-1-one* (**36**) Purification by column chromatography on silica gel (petroleum ether/ethyl acetate = 100:1, *v*/*v*) affords the title compound as a yellow oil (yield 15.4 mg, 54%). ^1^H NMR (500 MHz, Chloroform-d) *δ* 7.55 (dd, *J* = 7.8, 1.4 Hz, 1H), 7.51–7.46 (m, 1H), 7.27 (t, *J* = 8.1 Hz, 1H), 7.18 (d, *J* = 7.8 Hz, 2H), 7.10 (d, *J* = 7.9 Hz, 2H), 7.02 (dd, *J* = 8.2, 2.7 Hz, 1H), 4.78 (t, *J* = 7.4 Hz, 1H), 4.54–4.27 (m, 2H), 3.79 (s, 3H), 2.66–2.46 (m, 1H), 2.28 (s, 3H), 2.23–2.06 (m, 1H). ^13^C NMR (151 MHz, Chloroform-d) *δ* 198.99, 159.71, 137.81, 137.05, 135.43, 129.86, 129.48, 128.18, 121.43, 119.53, 113.04, 81.81 (d, *J* = 163.9 Hz), 55.36, 48.75 (d, *J* = 3.2 Hz), 34.38 (d, *J* = 19.3 Hz), 21.03. ^19^F NMR (565 MHz, Chloroform-d) *δ* −221.34 (tdd, *J* = 58.85, 33.90, 28.25 Hz, 1F). HRMS (ESI) (*m*/*z*): calcd for C_18_H_19_FNaO_2_ ([M + Na]^+^), 309.1261; found, 309.1261.
*4-fluoro-1-(m-tolyl)-2-(p-tolyl)butan-1-one* (**37**) Purification by column chromatography on silica gel (petroleum ether/ethyl acetate = 100:1, *v*/*v*) affords the title compound as a yellow oil (yield 14.6 mg, 50%). ^1^H NMR (500 MHz, Chloroform-d) *δ* 7.57 (d, *J* = 7.7 Hz, 1H), 7.28 (d, *J* = 7.4 Hz, 1H), 7.20–7.04 (m, 6H), 4.64 (t, *J* = 7.4 Hz, 1H), 4.56–4.30 (m, 2H), 2.67–2.51 (m, 1H), 2.31 (s, 3H), 2.27 (s, 3H), 2.21–2.06 (m, 1H). ^13^C NMR (151 MHz, Chloroform-d) *δ* 203.29, 138.39, 138.09, 137.03, 134.59, 131.61, 130.94, 129.69, 128.32, 128.04, 125.46, 81.97 (d, *J* = 164.0 Hz), 51.55 (d, *J* = 3.5 Hz), 33.79 (d, *J* = 19.1 Hz), 21.04, 20.78. ^19^F NMR (565 MHz, Chloroform-d) *δ* −221.33 (tdd, *J* = 45.20, 33.90, 28.25 Hz, 1F). HRMS (ESI) (*m*/*z*): calcd for C_18_H_19_FNaO ([M + Na]^+^), 293.1312; found, 293.1312.
*4-fluoro-1-(3-fluorophenyl)-2-(p-tolyl)butan-1-one* (**38**) Purification by column chromatography on silica gel (petroleum ether/ethyl acetate = 100:1, *v*/*v*) affords the title compound as a yellow oil (yield 11.5 mg, 42%). ^1^H NMR (500 MHz, Chloroform-d) *δ* 7.73 (dd, *J* = 7.8, 1.5 Hz, 1H), 7.62 (dt, *J* = 9.6, 2.1 Hz, 1H), 7.34 (td, *J* = 8.0, 5.5 Hz, 1H), 7.20–7.13 (m, 3H), 7.11 (d, *J* = 8.0 Hz, 2H), 4.73 (t, *J* = 7.3 Hz, 1H), 4.74–4.43 (m, 2H), 2.59–2.047 (m, 1H), 2.28 (s, 3H), 2.23–2.06 (m, 1H). ^13^C NMR (151 MHz, Chloroform-d) *δ* 197.94 (d, *J* = 2.2 Hz), 163.57, 161.93, 138.57 (d, *J* = 6.2 Hz), 137.28, 134.93, 130.14 (d, *J* = 7.5 Hz), 129.97, 128.17, 124.51 (d, *J* = 2.9 Hz), 119.96 (d, *J* = 21.5 Hz), 115.52 (d, *J* = 22.5 Hz), 81.66 (d, *J* = 164.1 Hz), 48.94 (d, *J* = 3.3 Hz), 34.28 (d, *J* = 19.5 Hz), 21.03. ^19^F NMR (565 MHz, Chloroform-d) *δ* -111.88 (d, *J* = 14.0 Hz, 1F), −221.41 (tdd, *J* = 50.85, 33.90, 28.25 Hz, 1F). HRMS (ESI) (*m*/*z*): calcd for C_17_H_16_F_2_NaO ([M + Na]^+^), 297.1061; found, 297.1066.
*1-(3,5-dimethylphenyl)-4-fluoro-2-(p-tolyl)butan-1-one* (**39**) Purification by column chromatography on silica gel (petroleum ether/ethyl acetate = 100:1, *v*/*v*) affords the title compound as a yellow oil (yield 14.2 mg, 50%).^1^H NMR (500 MHz, Chloroform-d) *δ* 7.56 (s, 2H), 7.18 (d, *J* = 7.8 Hz, 2H), 7.10 (d, *J* = 8.1 Hz, 3H), 4.79 (t, *J* = 7.4 Hz, 1H), 4.53–4.28 (m, 2H), 2.61–2.46 (m, 1H), 2.30 (s, 6H), 2.27 (s, 3H), 2.19–2.07 (m, 1H).^13^C NMR (151 MHz, Chloroform-d) *δ* 199.62, 138.09, 136.90, 136.60, 135.54, 134.67, 129.77, 128.16, 126.56, 81.92 (d, *J* = 163.6 Hz), 48.44 (d, *J* = 3.1 Hz), 34.43 (d, *J* = 19.3 Hz), 21.25, 21.02. ^19^F NMR (565 MHz, Chloroform-d) *δ* −221.29 (tdd, *J* = 50.85, 33.90, 28.25 Hz, 1F). HRMS (ESI) (*m*/*z*): calcd for C_19_H_21_FNaO ([M + Na]^+^), 307.1468; found, 307.1473.
*4-fluoro-1-(naphthalen-2-yl)-2-(p-tolyl)butan-1-one* (**40**) Purification by column chromatography on silica gel (petroleum ether/ethyl acetate = 100:1, *v*/*v*) affords the title compound as a yellow oil (yield 18.7 mg, 61%). ^1^H NMR (500 MHz, Chloroform-d) *δ* 8.36 (d, *J* = 8.5 Hz, 1H), 7.90 (d, *J* = 8.3 Hz, 1H), 7.85 (dd, *J* = 7.2, 1.2 Hz, 1H), 7.80 (dd, *J* = 8.0, 1.5 Hz, 1H), 7.55–7.43 (m, 3H), 7.42 (t, *J* = 7.7 Hz, 1H), 7.19 (d, *J* = 7.9 Hz, 2H), 7.06 (d, *J* = 7.8 Hz, 2H), 4.83 (t, *J* = 7.4 Hz, 1H), 4.63–4.29 (m, 2H), 2.80–2.64 (m, 1H), 2.30–2.17 (m, 4H). ^13^C NMR (151 MHz, Chloroform-d) *δ* 203.07, 137.06, 136.38, 134.74, 133.86, 132.37, 130.54, 129.75, 128.29, 128.26, 127.76, 127.27, 126.35, 125.60, 124.28, 82.00 (d, *J* = 164.0 Hz), 52.06 (d, *J* = 3.4 Hz), 34.12 (d, *J* = 19.2 Hz), 21.01. ^19^F NMR (565 MHz, Chloroform-d) *δ* −221.21 (tdd, *J* = 50.85, 33.90, 28.25 Hz, 1F). HRMS (ESI) (*m*/*z*): calcd for C_21_H_20_NaO ([M + H]^+^), 307.1492; found, 307.1485.
*4-fluoro-1-(thiophen-2-yl)-2-(p-tolyl)butan-1-one* (**41**) Purification by column chromatography on silica gel (petroleum ether/ethyl acetate = 100:1, *v*/*v*) affords the title compound as a yellow oil (yield 14.1 mg, 54%). ^1^H NMR (500 MHz, Chloroform-d) *δ* 7.71 (dd, *J* = 3.9, 1.1 Hz, 1H), 7.56 (dd, *J* = 5.0, 1.1 Hz, 1H), 7.22 (d, *J* = 7.9 Hz, 2H), 7.12 (d, *J* = 7.8 Hz, 2H), 7.04 (dd, *J* = 4.9, 3.8 Hz, 1H), 4.62 (t, *J* = 7.4 Hz, 1H), 4.56–4.27 (m, 2H), 2.61–2.45 (m, 1H), 2.28 (s, 3H), 2.18–2.03 (m, 1H). ^13^C NMR (151 MHz, Chloroform-d) *δ* 198.44, 137.84, 137.26, 135.67, 135.04, 130.19, 129.97, 128.15, 100.98, 81.68 (d, *J* = 163.7 Hz), 48.67 (d, *J* = 3.0 Hz), 34.18 (d, *J* = 19.4 Hz), 21.03. ^19^F NMR (565 MHz, Chloroform-d) *δ* −221.51 (tdd, *J* = 45.20, 28.25, 22.60 Hz, 1F). HRMS (ESI) (*m*/*z*): calcd for C_15_H_15_FNaOS ([M + Na]^+^), 285.0719; found, 285.0724.
*2,2,6,6-tetramethylpiperidin-1-yl benzoate* (**43**) Purification by column chromatography on silica gel (petroleum ether/ethyl acetate = 3:1, *v*/*v*) affords the title compound as a red solid (yield 11.2 mg, 43%). ^1^H NMR (500 MHz, Chloroform-*d*) *δ* 8.08 (d, *J* = 7.0 Hz, 2H), 7.58 (t, *J* = 7.5 Hz, 1H), 7.46 (t, *J* = 7.5 Hz, 2H), 1.83–1.74 (m, 2H), 1.75–1.66 (m, 1H), 1.61–1.57 (m, 2H), 1.50–1.43 (m, 1H), 1.28 (s, 6H), 1.12 (s, 6H). ^13^C NMR (151 MHz, Chloroform-*d*) *δ* 166.42, 132.88, 129.79, 129.60, 128.49, 60.44, 39.11, 32.01, 20.89, 17.05. HRMS (ESI) (*m*/*z*): calcd for C_16_H_23_NNaO_2_, ([M + Na]^+^), 284.1621, found 284.1614.
(5aR,10bS)-1-benzoyl-2-mesityl-2,5a,6,10b-tetrahydro-4H-indeno [2,1-b][1,2,4]tri-zolo *[4,3d][1,4]oxazin-11-ium tetrafluoroborate* (**44**) The white precipitate was filtered off and washed with diethyl ether. Drying under vacuum afforded the corresponding product as a white solid. ^1^H NMR (500 MHz, Chloroform-d) *δ* 8.24 (d, *J* = 7.8 Hz, 2H), 7.78 (t, *J* = 7.4 Hz, 1H), 7.70 (t, *J* = 7.6 Hz, 2H), 7.35 (d, *J* = 4.4 Hz, 2H), 7.28–7.23 (m, 2H), 7.11 (d, *J* = 7.7 Hz, 1H), 7.01 (s, 1H), 6.93 (s, 1H), 5.72 (d, *J* = 3.3 Hz, 1H), 5.51 (d, *J* = 16.3 Hz, 1H), 5.49–5.46 (m, 1H), 5.12 (d, *J* = 16.3 Hz, 1H), 3.30 (dd, *J* = 17.1, 4.0 Hz, 1H), 3.20 (d, *J* = 17.1 Hz, 1H), 2.32 (s, 3H), 2.17 (d, *J* = 13.6 Hz, 6H). **^1^**^3^C NMR (151 MHz, Chloroform-d) *δ* 179.11, 150.88, 147.75, 142.66, 140.83, 138.21, 135.66, 135.31, 132.10, 131.04, 130.72, 130.25, 130.13, 129.86, 129.40, 127.82, 126.34, 123.02, 63.80, 60.82, 37.25, 21.20, 17.58, 17.47. ^19^F NMR (565 MHz, Chloroform-d) *δ* −151.49. HRMS (ESI) (*m*/*z*): calcd for C_28_H_26_N_3_O_2_^+^, 437.2098; found, 437.2090.

## 5. Conclusions

In summary, we have developed a robust method for visible light-mediated monofluoromethylation/acylation of olefins, employing dual organo-catalysis. A cost-effective and bench-stable sodium monofluorosulfite (NaSO_2_CH_2_F) served as a CH_2_F radical source, while benzoyl fluoride acted as an acylating reagent under redox-neutral and metal-free conditions. Mechanistic investigations reveal that the reaction is initiated through the oxidative quenching of PC* by acylazolium, generating Breslow intermediate radicals (BIR) and proceeds through a sequential selective radical addition/radical-radical cross-coupling (RA/RRCC) cascade. This transformation features mild conditions, ease of operation, and a broad substrate scope, represents an attractive alternative for synthesizing versatile α-aryl-β-monofluoromethyl ketones. Further applications of this mild and metal-free CH_2_F radical generation strategy are actively being pursued in our laboratory.

## Figures and Tables

**Table 1:** Optimization of alkene monofluoromethylation/acylation.

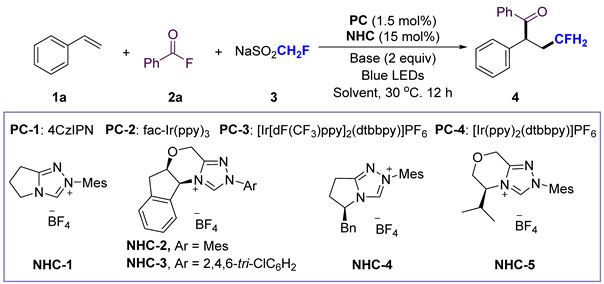					
Entry	NHCs (15 mol%)	PC (1.5 mol%)	Solvent (X mL)	Base (2.0 equiv)	Yield (%)
1	NHC-1	PC-1	DCM	Cs_2_CO_3_	50
2	NHC-1	PC-2	DCM	Cs_2_CO_3_	38
3	NHC-1	PC-3	DCM	Cs_2_CO_3_	49
4	NHC-1	PC-4	DCM	Cs_2_CO_3_	37
5	NHC-2	PC-1	DCM	Cs_2_CO_3_	60
6	NHC-3	PC-1	DCM	Cs_2_CO_3_	47
7	NHC-4	PC-1	DCM	Cs_2_CO_3_	6
8	NHC-5	PC-1	DCM	Cs_2_CO_3_	55
9	NHC-2	PC-1	PhCF_3_	Cs_2_CO_3_	36
10	NHC-2	PC-1	THF	Cs_2_CO_3_	20
11	NHC-2	PC-1	DCE	Cs_2_CO_3_	55
12	NHC-2	PC-1	Acetone	Cs_2_CO_3_	61
13	NHC-2	PC-1	Toluene	Cs_2_CO_3_	64
14	NHC-2	PC-1	CH_3_CN	Cs_2_CO_3_	89
15	NHC-2	PC-1	CHCl_3_	Cs_2_CO_3_	65
16	NHC-2	PC-1	CH_3_CN	K_2_CO_3_	32
17	NHC-2	PC-1	CH_3_CN	K_3_PO_4_	25
**18**	**rac-NHC-2**	**PC-1**	**CH_3_CN**	**Cs_2_CO_3_**	**90**

Reaction conditions: Unless otherwise noted, all the reactions were carried out with **1a** (0.1 mmol), **2a** (0.3 mmol), **3** (0.3 mmol), NHCs (0.015 mmol), Cs_2_CO_3_ (0.2 mmol), and PC (0.0015 mmol) in solvent (2 mL) at 30 °C with 10 W blue LEDs for 12 h.

## Data Availability

The data presented in this study are available in the Supplementary Materials.

## Supplementary Materials

The following supporting information can be downloaded at https://www.mdpi.com/article/10.3390/molecules29040790/s1: ^1^H and ^13^C NMR data and spectra for all compounds. Refs. [83,84,85] are cited in Supplementary Materials.
