# Supplementary material for: Visible Light-Mediated Monofluoromethylation/Acylation of Olefins by Dual Organo-Catalysis
Source: Molecules. 2024 Feb 8;29(4):790. doi: 10.3390/molecules29040790 (PMC10892033; doi:10.3390/molecules29040790)

Supporting Information

Visible Light-Mediated Monofluoromethylation/Acylation of Olefins by Dual Organo-Catalysis

Jiuli Xia [1], Yunliang Guo [2], Zhiguang Lv [1], Jiaqiong Sun [2,*], Guangfan Zheng [1,*] and Qian Zhang [1,3]

[1] Key Laboratory of Functional Organic Molecule Design & Synthesis of Jilin Province, Department of Chemistry, Northeast Normal University, Changchun 130024, China; xiajl699@nenu.edu.cn (J.X.); lvzhiguang@nenu.edu.cn (Z.L.); zhangq651@nenu.edu.cn (Q.Z.)

[2] School of Environment, Northeast Normal University, Changchun 130117, China;

guoyl367@nenu.edu.cn

[3] State Key Laboratory of Organometallic Chemistry, Shanghai Institute of Organic Chemistry,

Chinese Academy of Sciences, 345 Lingling Road, Shanghai 200032, China

*Correspondence: sunjq295@nenu.edu.cn (J.S.); zhenggf265@nenu.edu.cn (G.Z.)

Table of Contents

I. General Information······S1

II. General Procedure for the Monofluoromethylative Acylation of Olefins······S2

III. Preparation of the Starting Materials······S3

IV. Enantioselective Transformations······S5

V. Mechanistic Studies······S7

VI. References······S11

VII. ^{1}H, ^{13}C, ^{19}F NMR Spectra of New Compounds······S12

I. General Information

All reactions were carried out under nitrogen atmosphere. Reagents were purchased from commercial sources and used without further purification, unless otherwise noted. All of the solvents were anhydrous according to distillation. The reactions were monitored with the aid of thin-layer chromatography (TLC) on 0.25 mm precoated silica gel plates. Melting points were measured on Büchi B-540 apparatus. ^{1}H NMR spectra were recorded at 25 ºC on a Bruker 600 or 500, Varian 500 MHz, ^{13}C NMR spectra were recorded at 25 ºC on a Bruker 151, Varian 126 MHz, respectively in $CDCl_3$ by using TMS as internal standard. ^{19}F NMR spectra were recorded at 25 ºC on a Bruker 565 MHz. ^{1}H and ^{13}C NMR spectra are reported in parts per million (ppm) downfield from an internal standard, tetramethylsilane (0 ppm for ^{1}H NMR) and $CDCl_3$ (77.0 ppm for ^{13}C NMR), respectively. Letters m, s, d, t, and q stand for multiplet, singlet, doublet, triplet, and quartet, respectively. High-resolution mass spectra (HRMS) were recorded on Bruck microtof. We use RLH-18 8-position Photo Reaction System, which is manufactured by Beijing Rogertech Co.ltd base in Beijing PRC. This Photo reactor we used has equipped with 8 blue light 40 W LED. This blue light 40 W LED's energy peak wavelength is 453.6 nm, and peak width at half-height is 20.4 The irradiationtion vessel is a borosilicate glass test tube, LED irradiates through a high-reflection channel to the test tube, the path length is 2 cm and no filter between LED and test tube.

II. General Procedure for the Monofluoromethylative Acylation of Olefins

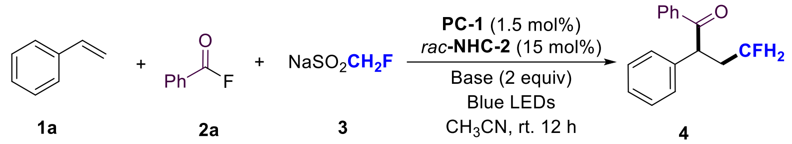

Taking 4 as an example: Into a nitrogen-filled glove box, a vial equipped with a magnetic stir bar was charged with *rac*-**NHC-2** (6.3 mg, 0.015 mmol), Cs_2CO_3 (65.2 mg, 0.2 mmol), **PC-1** (1.2 mg, 0.0015 mmol), **3** (36.0 mg, 0.3 mmol) and CH_3CN (2.0 mL). Then **1a** (0.1 mmol) and sulfinate **2a** (0.3 mmol) were added. The vial was removed from the glovebox, and then the reaction mixture was irradiated with Blue LED at 30 ^{o}C for 12 hours. After that, the residue was purified by flash column chromatography (petroleum ether/ethyl acetate = 100 : 1) to give the corresponding product **4**.

III. Preparation of the Starting Materials

List of styrene

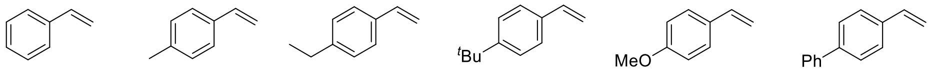

1a **1b** **1c** **1d** **1e** **1f**

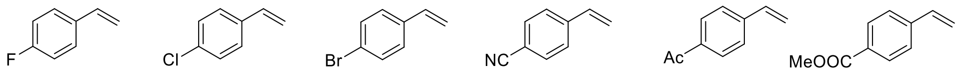

1g **1h** **1i** **1j** **1k** **1l**

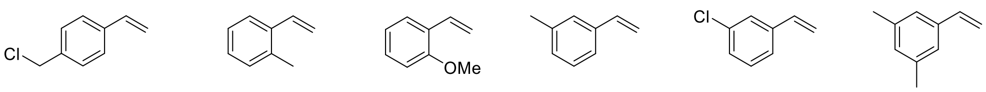

1m **1n** **1o** **1p** **1q** **1r**

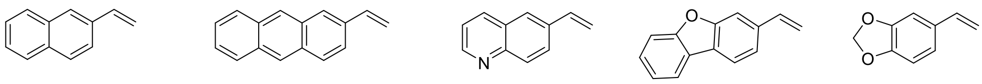

1s **1t** **1u** **1v** **1w**

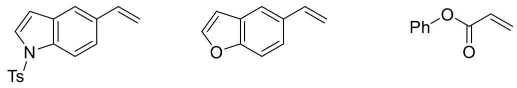

1x **1y** **1z**

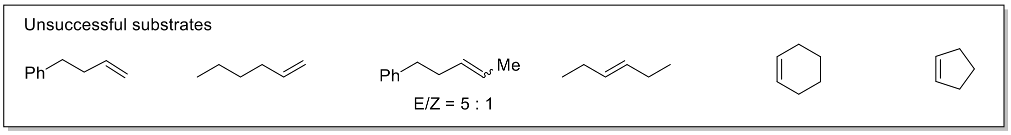

Table **S1**. The Substrates for Styrene

Vinylarenes **1a-1i, 1s, 1m-1q** were purchased from Alfa Aeser or Energy-Chemical Ltd. Substrates **1j-1l, 1r, 1t-1y** are known compounds and were prepared by previously reported procedures. [83-85]

List of Acyl Fluoride

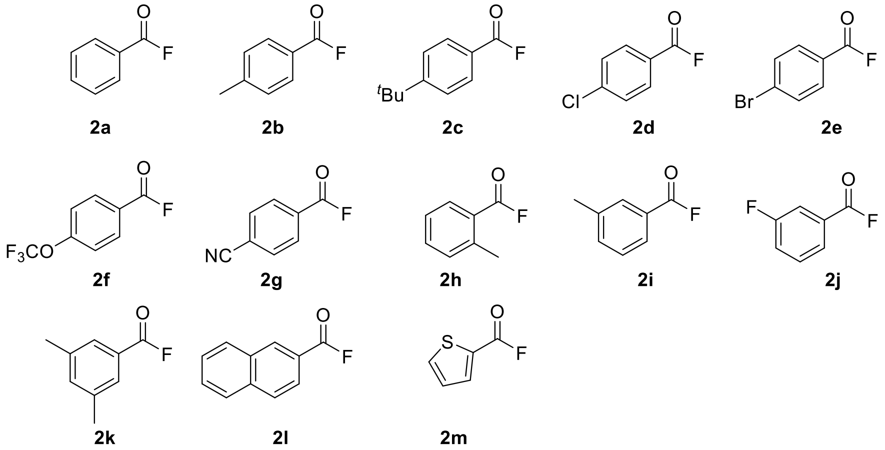

Table **S2**. The Substrates for Acyl Fluorides

Synthesis of Acyl Fluoride

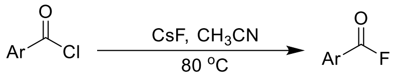

A 25.0 mL round-bottomed flask equipped with a magnetic stir bar was charged with benzoyl chloride (5.0 mmol) and anhydrous acetonitrile (5.0 mL). Cesium fluoride (1.1 g, 7.5 mmol, 1.5 equiv) was added and the mixture was stirred for 2-4 hours (monitored by TLC) at 80 °C under a nitrogen atmosphere. Then the resulting crude product was purified by column chromatography to yield the acyl fluorides. [73]

IV. Enantioselective Transformations

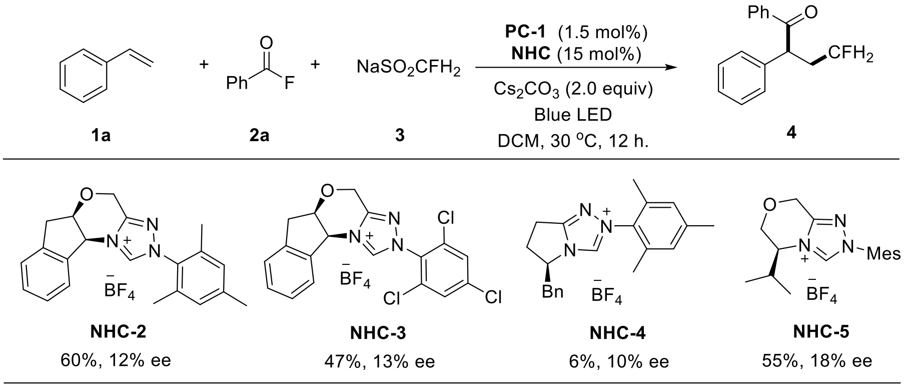

Figure **S1** Enantioselective Transformations

Screening of chiral NHC catalysts. Reaction conditions: **1a** (0.1 mmol), **2a** (0.3 mmol), **3** (0.3 mmol), **NHC** (15 mol%), **PC-1** (1.5 mol%), and Cs_2CO_3 (0.4 mmol) in CH_3CN (2.0 mL), Blue LEDs, nitrogen atmosphere, 30 °C, 12 h.

When *rac*-**NHC-2** was used as a catalyst, 50:50 er was determined by HPLC (IA-3, Hexane/IPA = 95/5, 0.8 mL/min, 254 nm). tR (major) = 9.060 min, tR (minor) = 10.075 min.

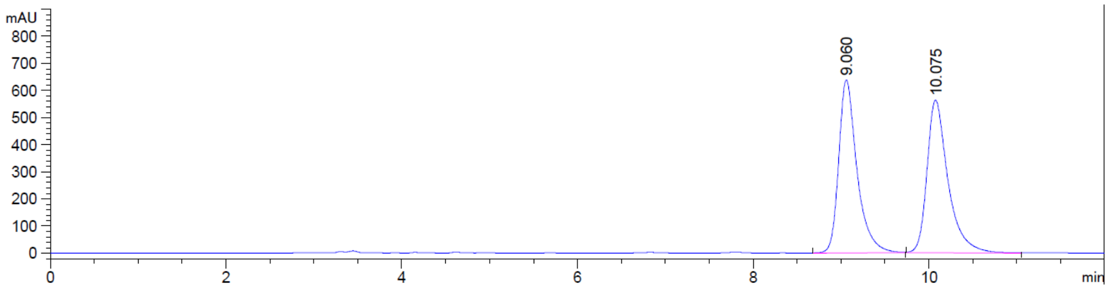

Peak	PetTime	Type	Area(mAU*S)	Hight(mAU)	Area%
1	9.060	M	9190.73535	637.48199	49.6328
2	10.075	M	9326.72656	562.58630	50.3672

When **NHC-2** was used as catalyst, 44:56 er was determined by HPLC (IA-3, Hexane/IPA = 95/5, 0.8 mL/min, 254 nm). tR (minor) = 8.957 min, tR (major) = 9.964 min.

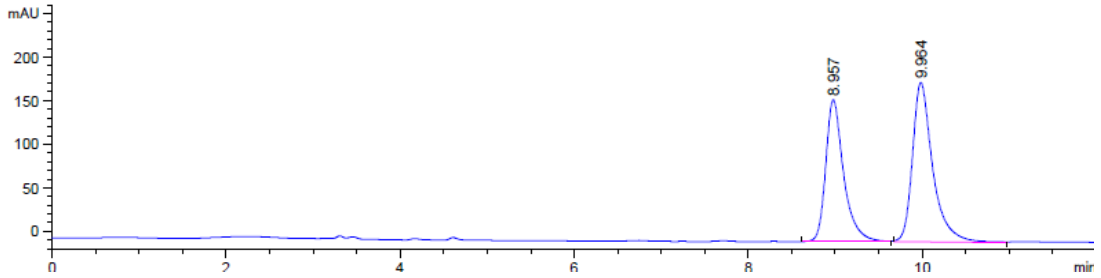

Peak	PetTime	Type	Area(mAU*S)	Hight(mAU)	Area%
1	8.957	M	2309.13672	162.48517	43.6037
2	9.964	M	2986.60474	183.00700	56.3963

When **NHC-3** was used as catalyst, 43:57 er was determined by HPLC (IA-3, Hexane/IPA = 95/5, 0.8 mL/min, 254 nm). tR (minor) = 9.001 min, tR (major) = 10.014 min.

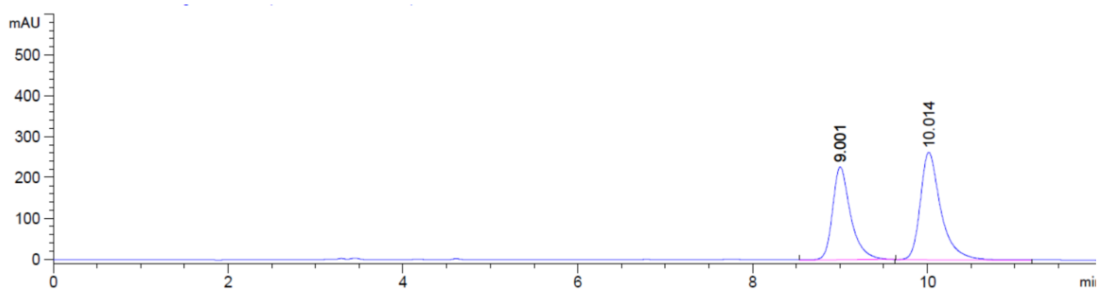

Peak	PetTime	Type	Area(mAU*S)	Hight(mAU)	Area%
1	9.001	M	3261.27393	227.21556	43.1065
2	10.014	M	4304.34082	263.01050	56.8935

When **NHC-4** was used as catalyst, 45:55 er was determined by HPLC (IA-3, Hexane/IPA = 95/5, 0.8 mL/min, 254 nm). tR (minor) = 9.009 min, tR (major) = 10.023 min.

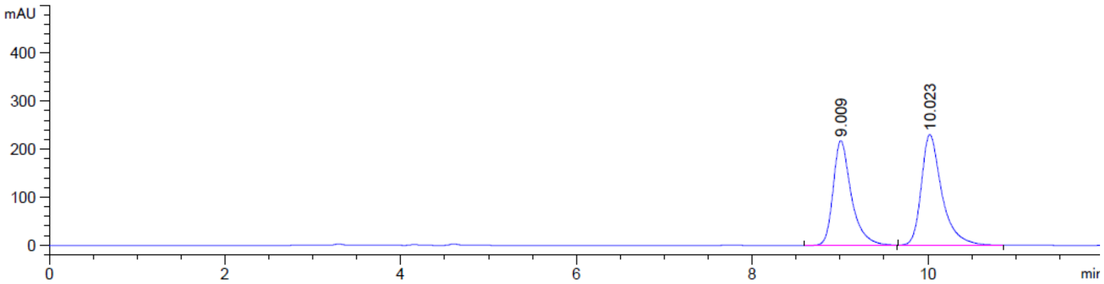

Peak	PetTime	Type	Area(mAU*S)	Hight(mAU)	Area%
1	9.009	M	3159.17114	218.07402	45.4967
2	10.023	M	3784.56396	230.90131	54.5033

When **NHC-5** was used as catalyst, 41:59 er was determined by HPLC (IA-3, Hexane/IPA = 95/5, 0.8 mL/min, 254 nm). tR (minor) = 8.973 min, tR (minor) = 9.985 min.

Peak	PetTime	Type	Area(mAU*S)	Hight(mAU)	Area%
1	8.973	M	3886.38062	259.66516	41.1715
2	9.985	M	5553.12158	331.30139	58.8285

Employing chiral **NHC-2** as catalyst and CH_3CN as solvent, **4** (89%, 12% ee)
(IA-3, Hexane/IPA = 95/5, 0.8 mL/min, 254 nm). tR (minor) = 9.025 min, tR (major) = 10.031 min.

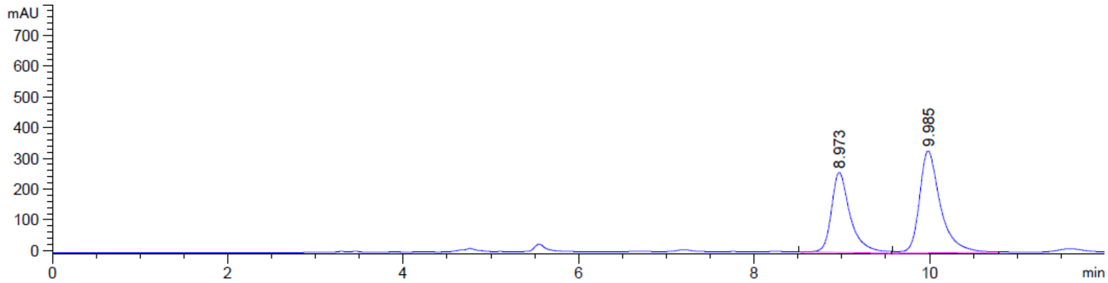

Peak	PetTime	Type	Area(mAU*S)	Hight(mAU)	Area%
1	9.025	M	3179.32223	438.80176	43.5378
2	10.031	M	4067.74152	454.54181	56.4622

V. Mechanistic Studies

Control Experiment

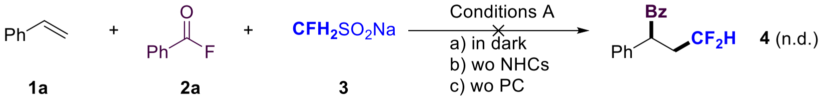

Into a nitrogen-filled glove box, a vial (10.0 mL) equipped with a magnetic stir bar was charged with *rac*-**NHC-2** (6.3 mg, 0.015 mmol), Cs_2CO_3 (65.2 mg, 0.2 mmol), **PC-1** (1.2 mg, 0.0015 mmol) **3** (36.0 mg, 0.3 mmol) and CH_3CN (2.0 mL). Then **1a** (0.1 mmol) and **2a** (0.3 mmol) were added. The vial was removed from the glovebox, and then the reaction mixture was stirred in the dark for 12 hours. After that, the residue was analyzed by ^{1}H NMR, the product **4** was not detected.

Into a nitrogen-filled glove box, a vial (10.0 mL) equipped with a magnetic stir bar was charged with Cs_2CO_3 (65.2 mg, 0.2 mmol), **PC-1** (1.2 mg, 0.0015 mmol) **3** (36.0 mg, 0.3 mmol) and CH_3CN (2.0 mL). Then **1a** (0.1 mmol) and **2a** (0.3 mmol) were added. The vial was removed from the glovebox, and then the reaction mixture was irradiated with Blue LED for 12 hours. After that， the residue was analyzed by ^{1}H NMR, and product **4** was not detected.

Into a nitrogen-filled glove box, a vial (10.0 mL) equipped with a magnetic stir bar was charged with *rac*-**NHC-2** (6.3 mg, 0.015 mmol), Cs_2CO_3 (65.2 mg, 0.2 mmol), **3** (36.0 mg, 0.3 mmol) and CH_3CN (2.0 mL). Then **1a** (0.1 mmol) and **2a** (0.3 mmol) were added. The vial was removed from the glovebox, and then the reaction mixture was irradiated with Blue LED for 12 hours. After that the residue was analyzed by ^{1}H NMR, the product **4** was not detected.

The Radical Scavenger Experiment

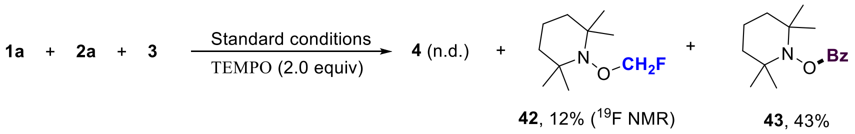

Into a nitrogen-filled glove box, a vial (10.0 mL) equipped with a magnetic stir bar was charged with *rac*-**NHC-2** (6.3 mg, 0.015 mmol), Cs_2CO_3 (65.2 mg, 0.2 mmol), **PC-1** (1.2 mg, 0.0015 mmol), sulfinate **3** (0.3 mmol) and CH_3CN (2.0 mL). Then **1a** (0.1 mmol) and **2a** (0.3 mmol) were added. Finally, TEMPO (0.2 mmol, 2.0 equiv) was added to the mixture. The vial was removed from the glovebox and then the reaction mixture was irradiated with Blue LED for 12 hours. After that， the residue was analyzed by ^{1}H NMR, the product **4** was not detected, then the residue was purified by flash column chromatography (petroleum ether/ethyl acetate = 20 : 1) to give the corresponding product **42** with 12% yield by ^{19}F NMR and **43** with 43% yield (11.2 mg).

Synthesis of Acyl Azolium 44

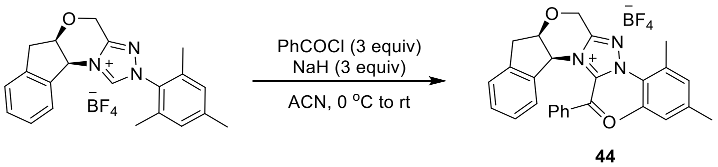

A dry Schlenk tube under an atmosphere of Argon, containing carbene precursor (2 mmol) and benzoyl chloride (3 equiv) in dry acetonitrile, is cooled to 0 °C. NaH (60% wt. in oil; 3 equiv) is added portion-wise to the stirred solution over 1.5 hours. The reaction mixture was allowed to warm to room temperature and monitored by TLC for completion. After that, the precipitate was collected by filtration,

washed with Et_2O several times and dried under vacuum to give the corresponding product **44** as a white solid (574.9 mg, 54%).

Catalytic activity and transformation of 44

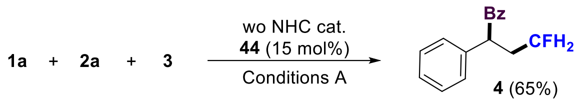

Into a nitrogen-filled glove box, a vial (10.0 mL) equipped with a magnetic stir bar was charged with Cs_2CO_3 (65.2 mg, 0.4 mmol), **PC-1** (1.2 mg, 0.0015 mmol) **3** (0.3 mmol) and CH_3CN (2.0 mL). Then **1a** (0.1 mmol), **2a** (0.3 mmol), and **44** (0.015 mmol) were added. The vial was removed from the glovebox, and then the reaction mixture was irradiated with Blue LED for 12 hours. After that, the residue was purified by flash column chromatography (petroleum ether/ethyl acetate = 100 : 1) to give the corresponding product **4** (15.7 mg, 65%) .

Light on-off Experiment

Into a nitrogen-filled glove box, a vial (15.0 mL) equipped with a magnetic stir bar was charged with *rac*-**NHC-2** (6.3 mg, 0.015 mmol), Cs_2CO_3 (65.2 mg, 0.2 mmol), **PC-1** (1.2 mg, 0.0015 mmol) sodium monofluoromethanesulfinate **3** (36.0 mg, 0.3 mmol) and CD_3CN (2.0 mL). Then **1a** (0.1 mmol), **2a** (0.3 mmol) and dibromomethane (14 μL, 0.2 mmol) were added. The vial was removed from the glovebox, and then the reaction mixture was irradiated with Blue LED and kept in the dark in 10 minutes intervals at room temperature. Yields of the **5** were determined by **^{1}H NMR** monitors with dibromomethane as the internal standard. The reaction proceeded well under the irradiation of visible light, but no further transformation was observed without the light irradiation, indicating that the continuous irradiation of visible light is essential for this catalytic reaction.

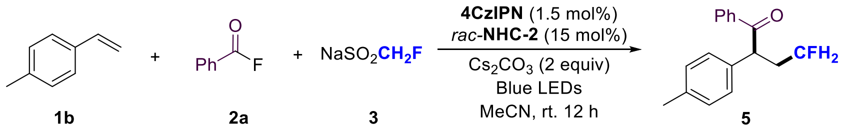

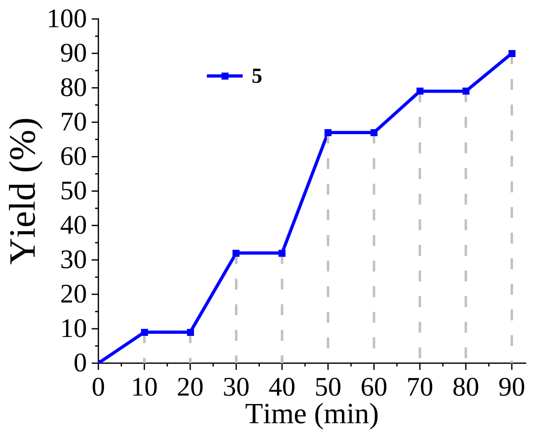

Figure **S2** Light on-off Experiment

Emission quenching experiments

Emission intensities were recorded using a spectrofluorometer (Edinburgh FS5) at ambient temperature. 4CzIPN solutions were excited at 430 nm. Firstly, the emission spectrum of a 5 x 10^{-5} M solution of 4CzIPN in CH_3CN was collected. Then, an appropriate amount of quencher was added to the measured solution, and the emission spectrum of the sample was collected. The emission spectra of a solution of

PC-1 (5 x 10^{-5} M in CH_3CN) upon excitation at 425 nm were recorded (Figure **S2**, black line). Then, sodium monofluoromethanesulfinate **3**, styrene **1a,** benzoyl fluoride **2a** and acyl azolium ion **44** were separately added to the solution, and the emission spectra were recorded separately. The Stern-Volmer emission quenching studies tell that the acyl azolium ion **44** are easier than sodium monofluoromethanesulfinate **3**, styrene **1a** and benzoyl fluoride **2a** to quench the excited photosensitizer.

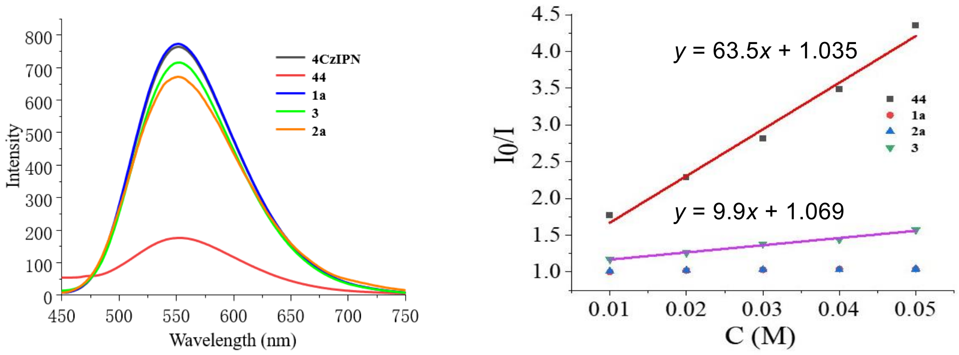

Figure **S3** 4CzIPN emission quenching by sodium monofluoromethanesulfinate **3**, styrene **1a**, benzoyl fluoride **2a** and azolium ion **44**

Emission intensities were recorded using a spectrofluorometer (HITACHI F4600) at ambient temperature. 4CzIPN solutions were excited at 430 nm, Then, sodium monofluoromethanesulfinate **3**, styrene **1a,** benzoyl fluoride **2a** and acyl azolium ion **44** were separately added to the solution, and the emission spectra were recorded separately.

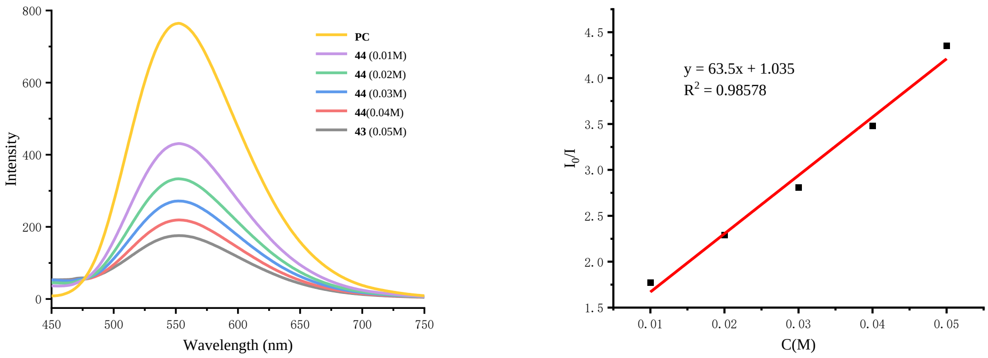

Figure **S4** 4CzIPN emission quenching by azolium ion **44**.

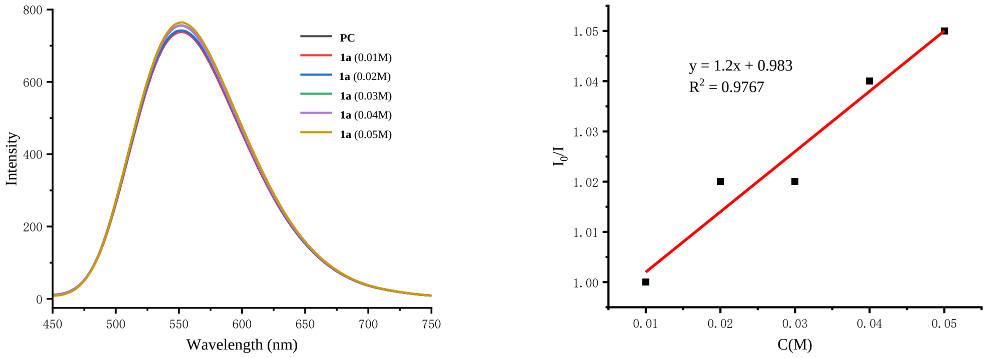

Figure **S5** 4CzIPN emission quenching by styrene **1a**.

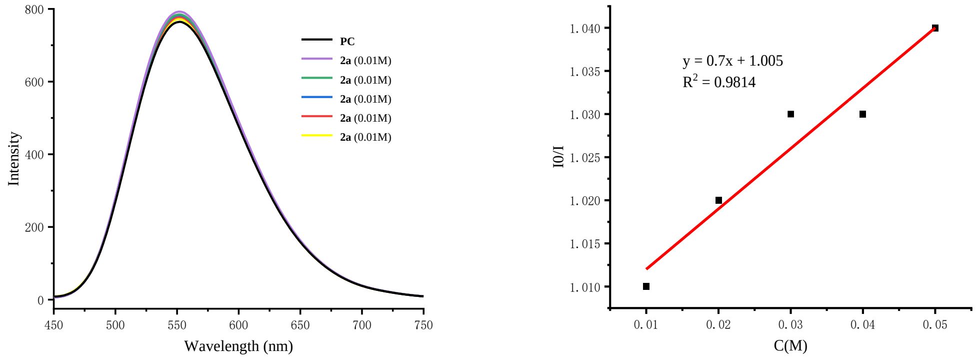

Figure **S6** 4CzIPN emission quenching by enzoyl fluoride **2a**.

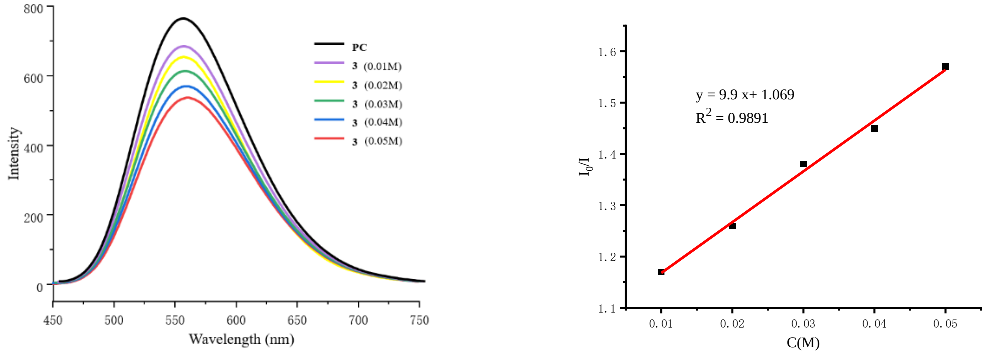

Figure **S7** 4CzIPN emission quenching by monofluoromethanesulfinate **3**.

VI. References

83. Qin T, Lv G, Meng Q, Zhang G, Xiong T, Zhang Q. *Angew. Chem. Int. Ed*. **2021**, *60*, 25949-25957.

84. MacKenzie IA, Wang L, Onuska NPR, Williams OF, Begam K, Moran AM, Dunietz BD, Nicewicz DA. *Nature*. **2020**, *580*, 76-80.

85. Molander GA, Brown AR. *J. Org. Chem.* **2006**, *71*, 9681-96864.

75. Wang L, Ma R, Sun J, Zheng G, and Zhang Q. *Chem. Sci*., **2022**, *13*, 3169-3175.

VII. ^{1}H, ^{13}C, ^{19}F NMR Spectra of New Compounds

^{1}H NMR (500 MHz, $CDCl_3$) spectrum for **4**.

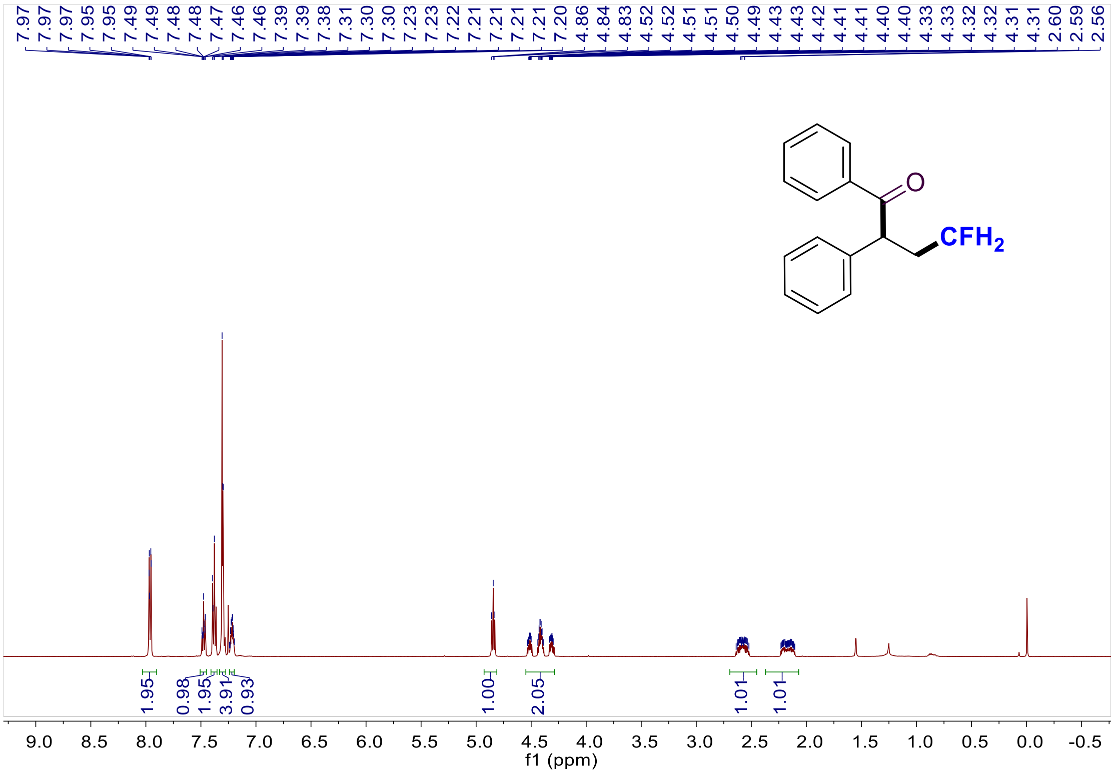

^{13}C NMR (151 MHz, $CDCl_3$) spectrum for **4**.

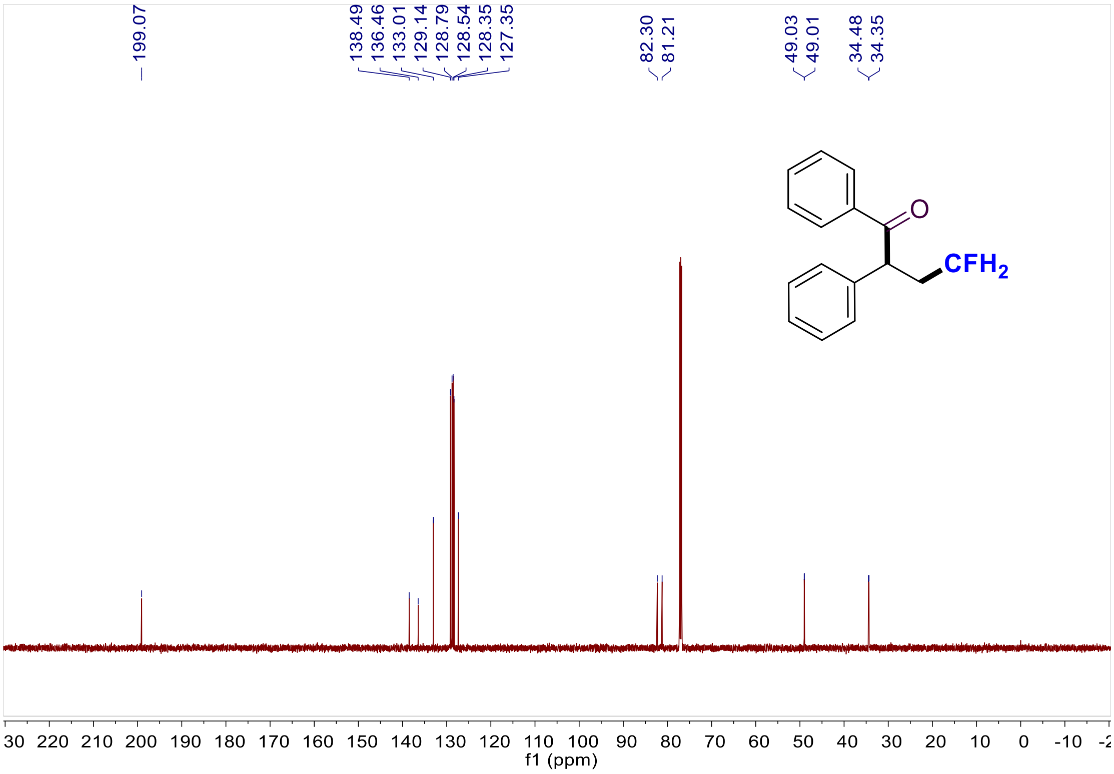

^{19}F NMR (565 MHz, $CDCl_3$) spectrum for **4**.

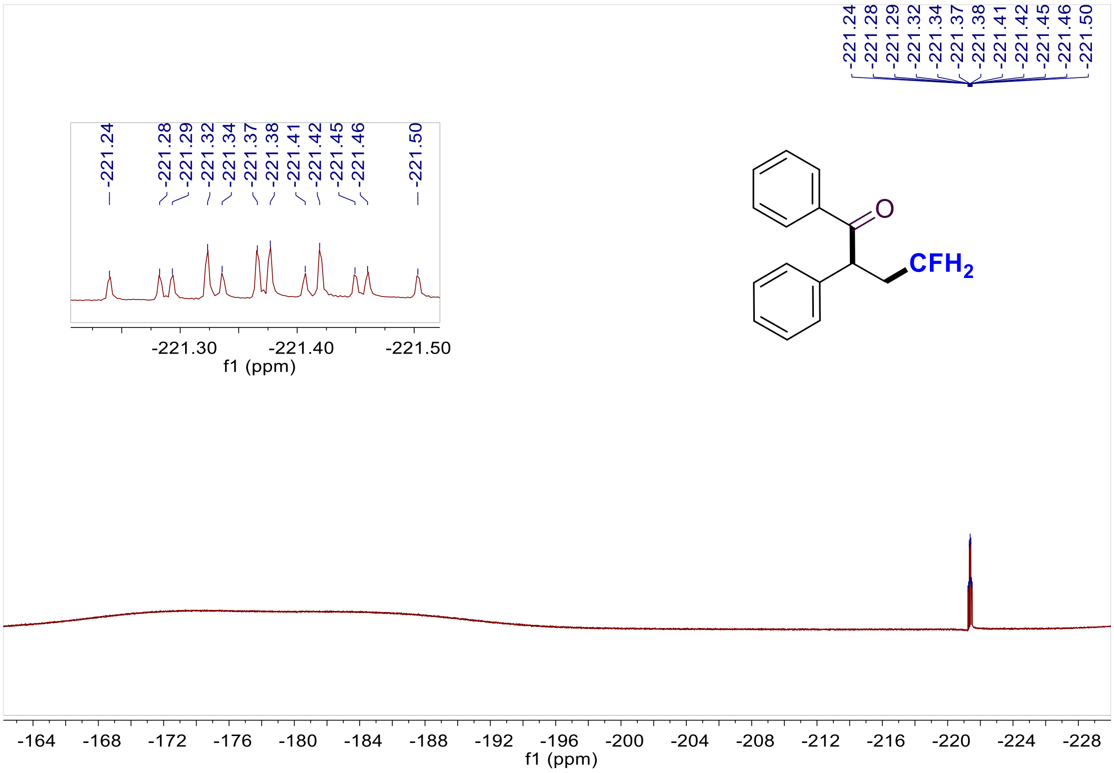

^{1}H NMR (500 MHz, $CDCl_3$) spectrum for **5**.

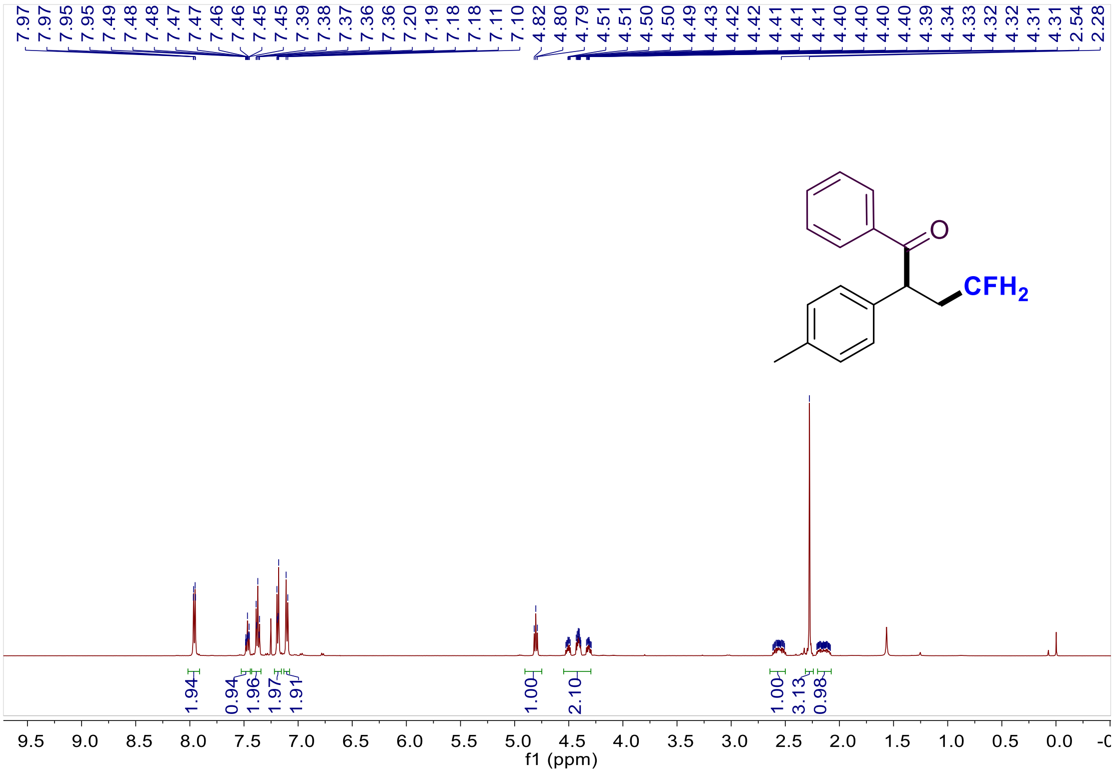

^{13}C NMR (151 MHz, $CDCl_3$) spectrum for **5.**

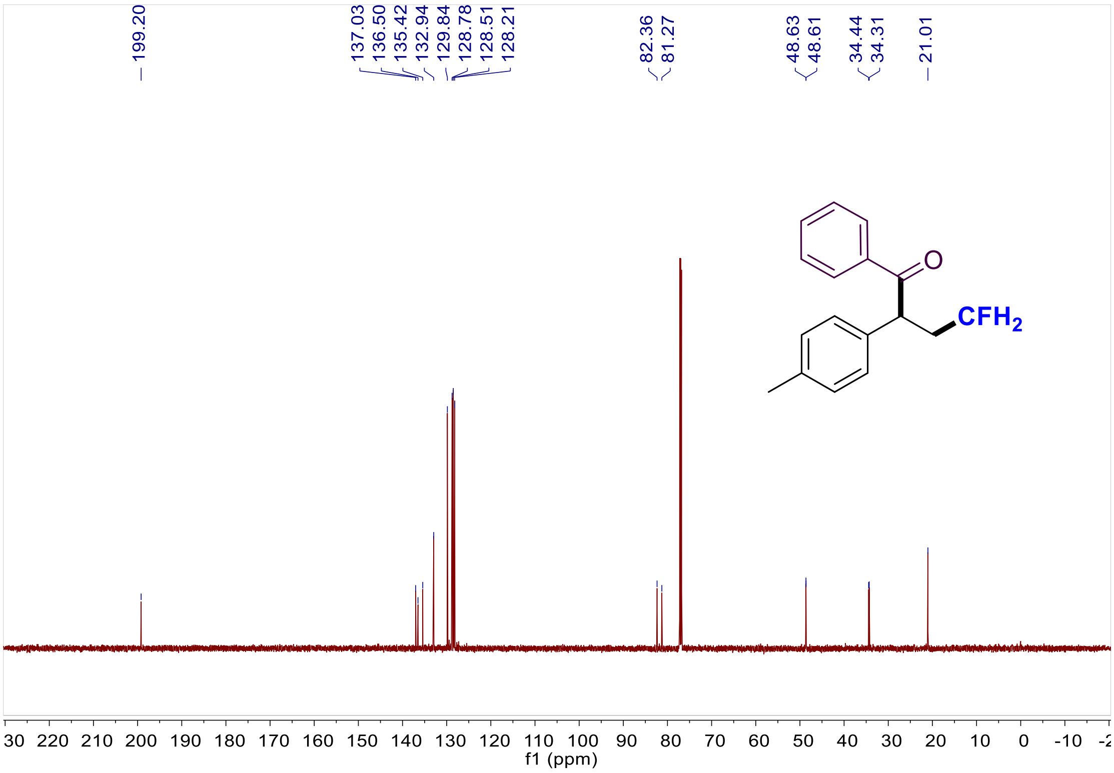

^{19}F NMR (565 MHz, $CDCl_3$) spectrum for **5**.

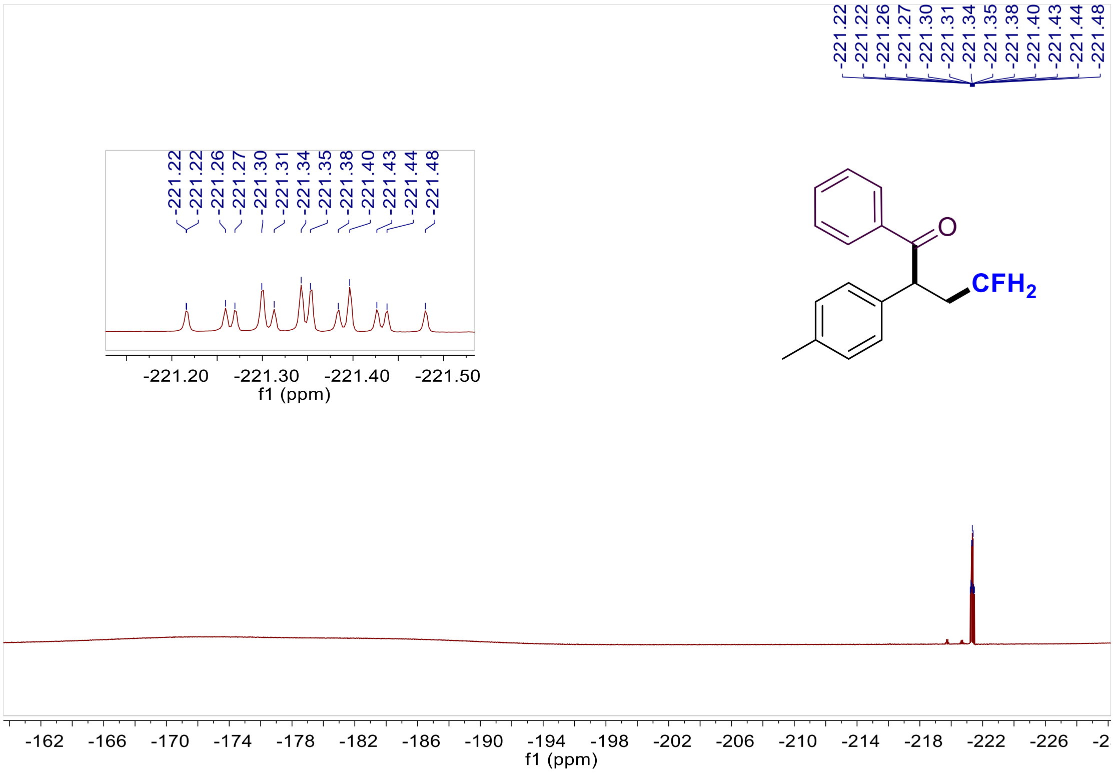

^{1}H NMR (500 MHz, $CDCl_3$) spectrum for **6**.

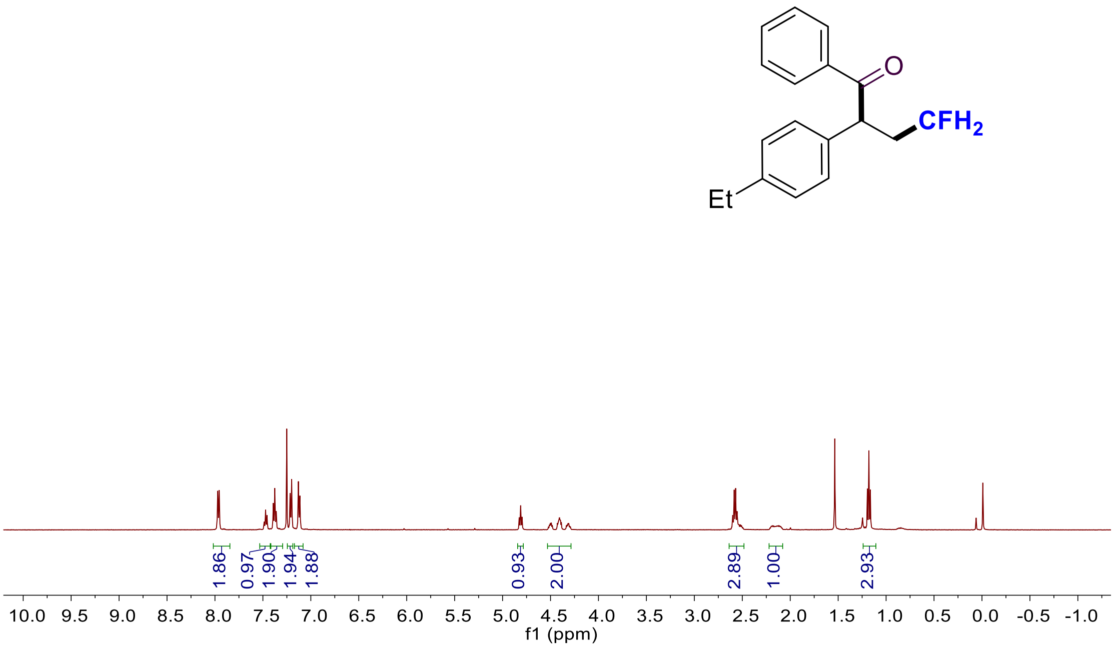

^{13}C NMR (151 MHz, $CDCl_3$) spectrum for **6.**

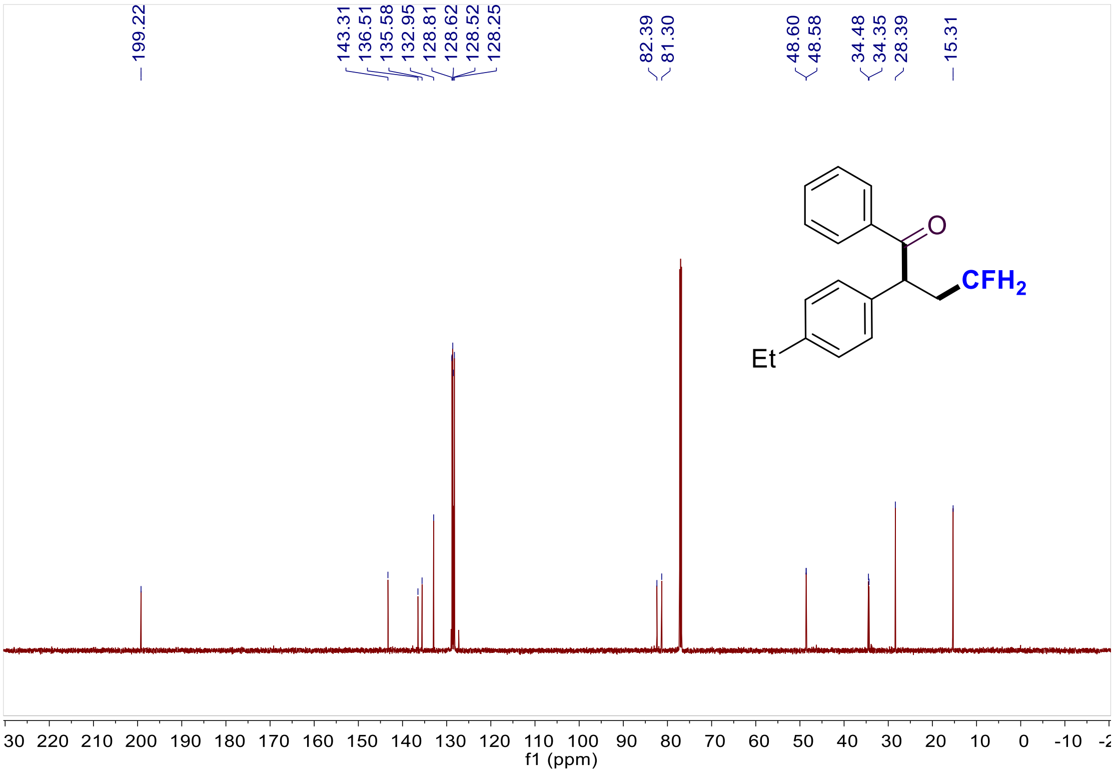

^{19}F NMR (565 MHz, $CDCl_3$) spectrum for **6**.

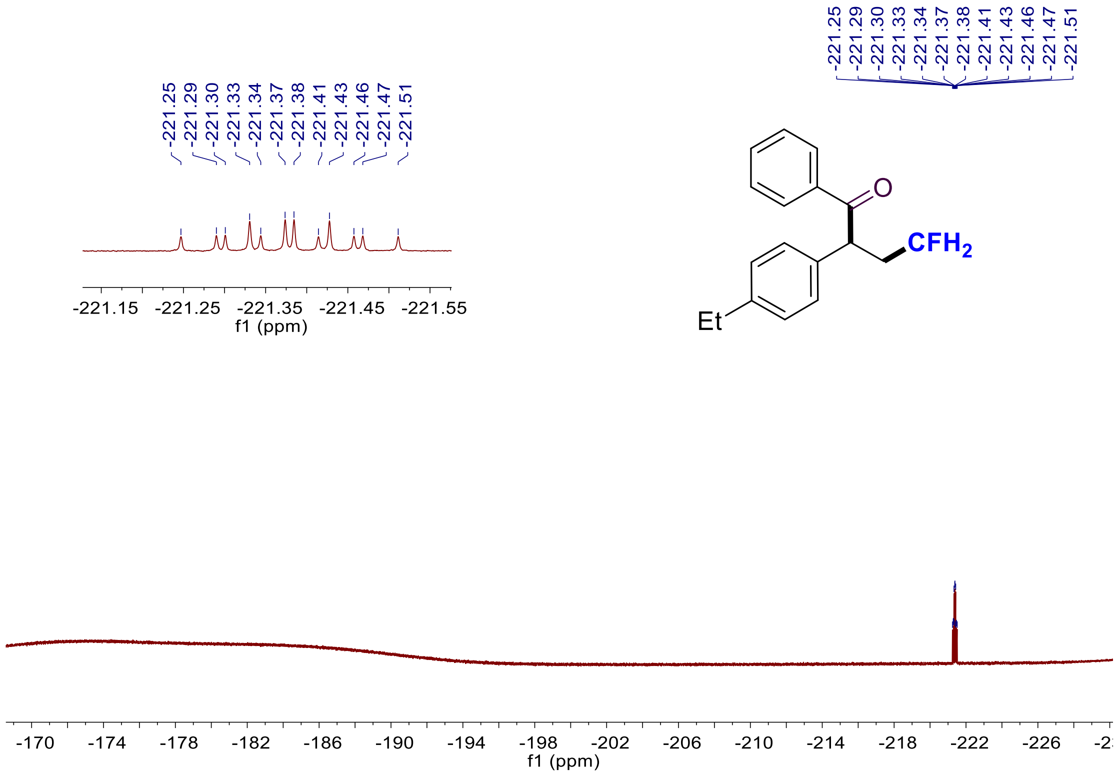

^{1}H NMR (500 MHz, $CDCl_3$) spectrum for **7**.

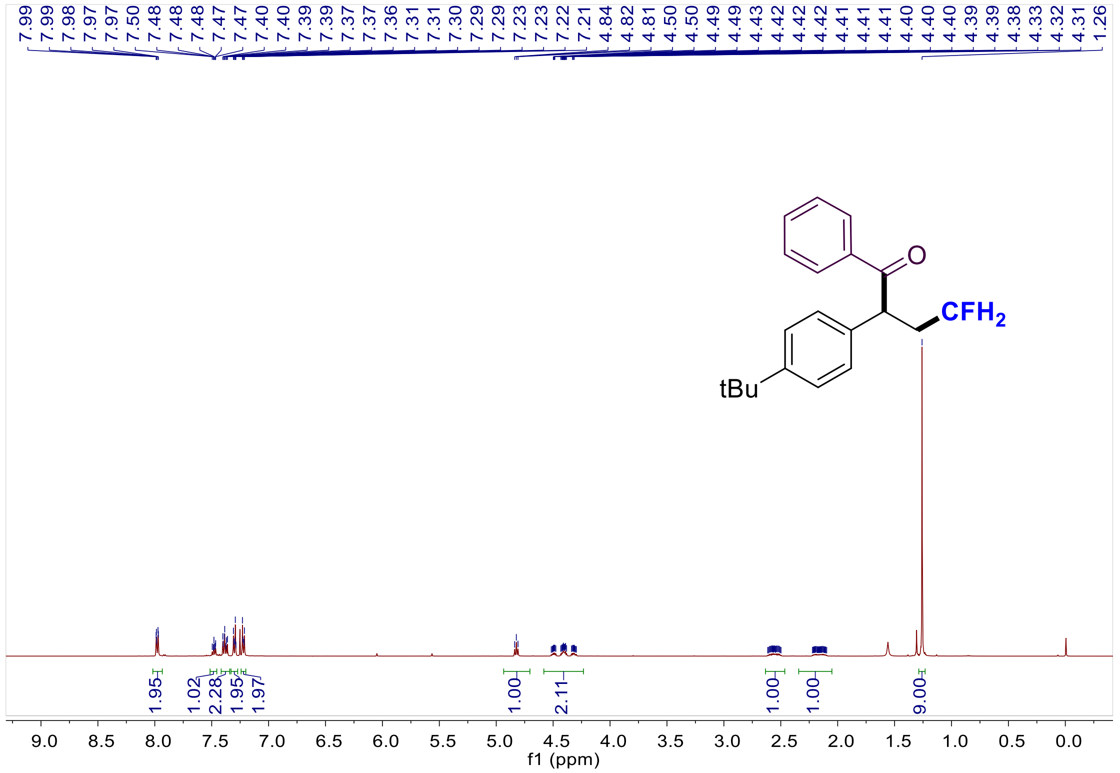

^{13}C NMR (151 MHz, $CDCl_3$) spectrum for **7**.

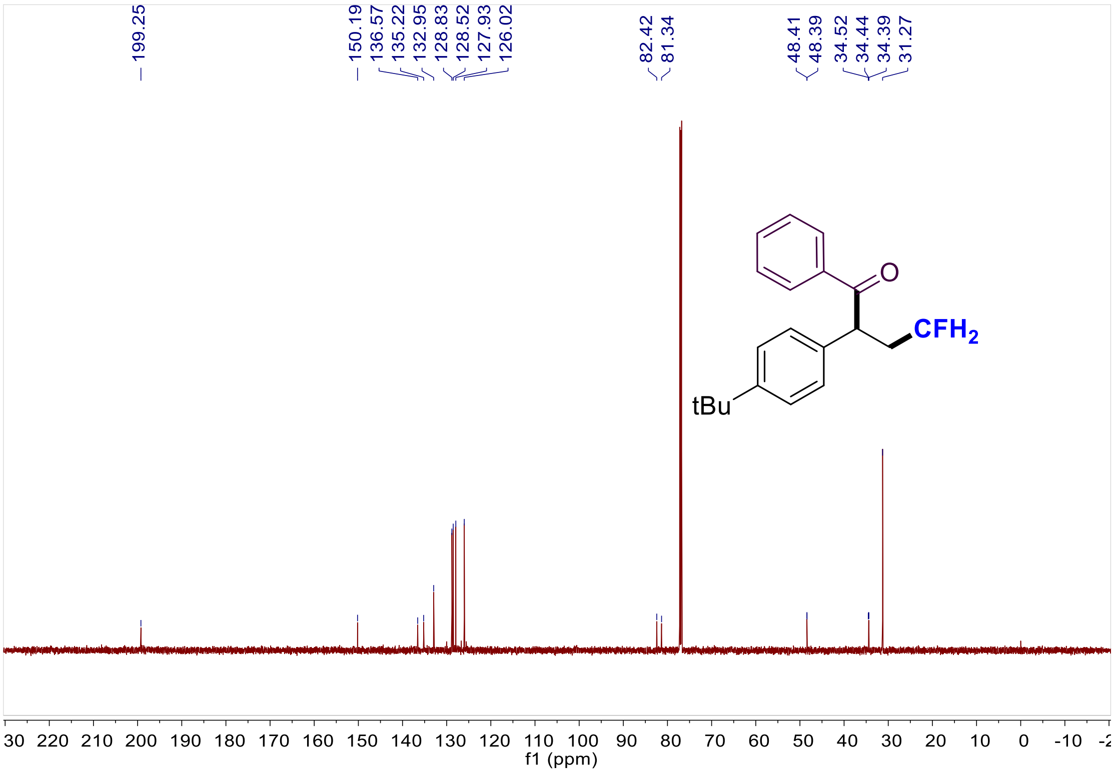

^{19}F NMR (565 MHz, $CDCl_3$) spectrum for **7**.

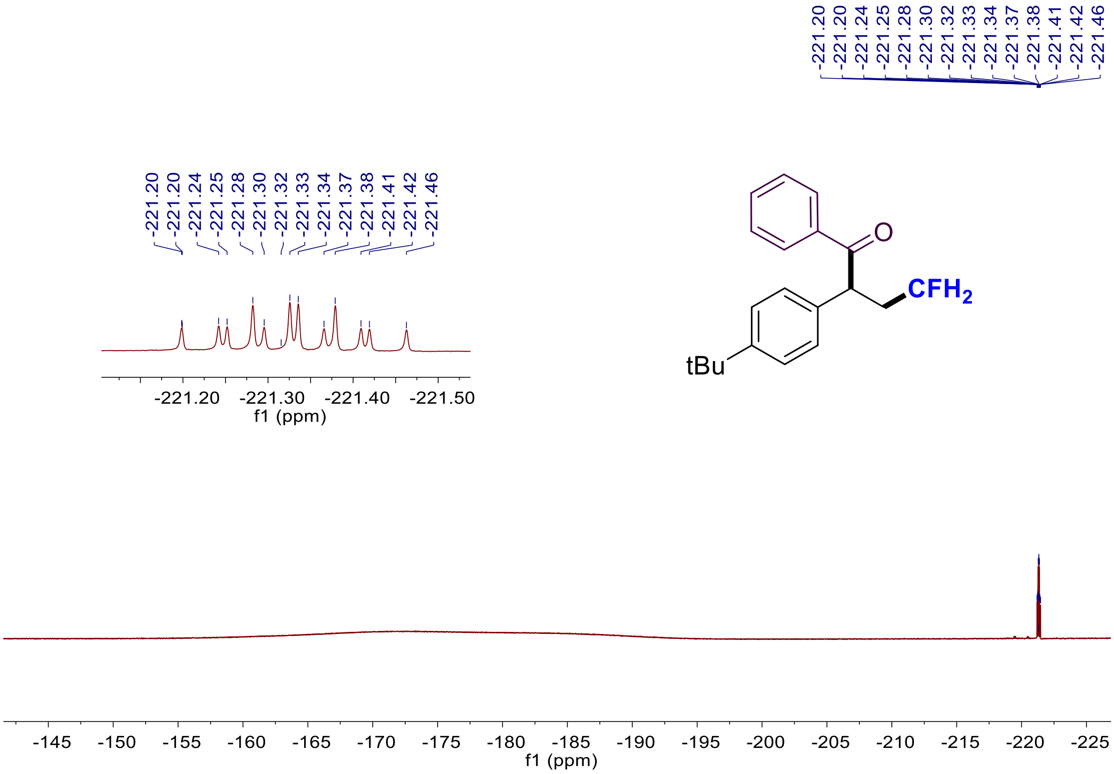

^{1}H NMR (500 MHz, $CDCl_3$) spectrum for **8**.

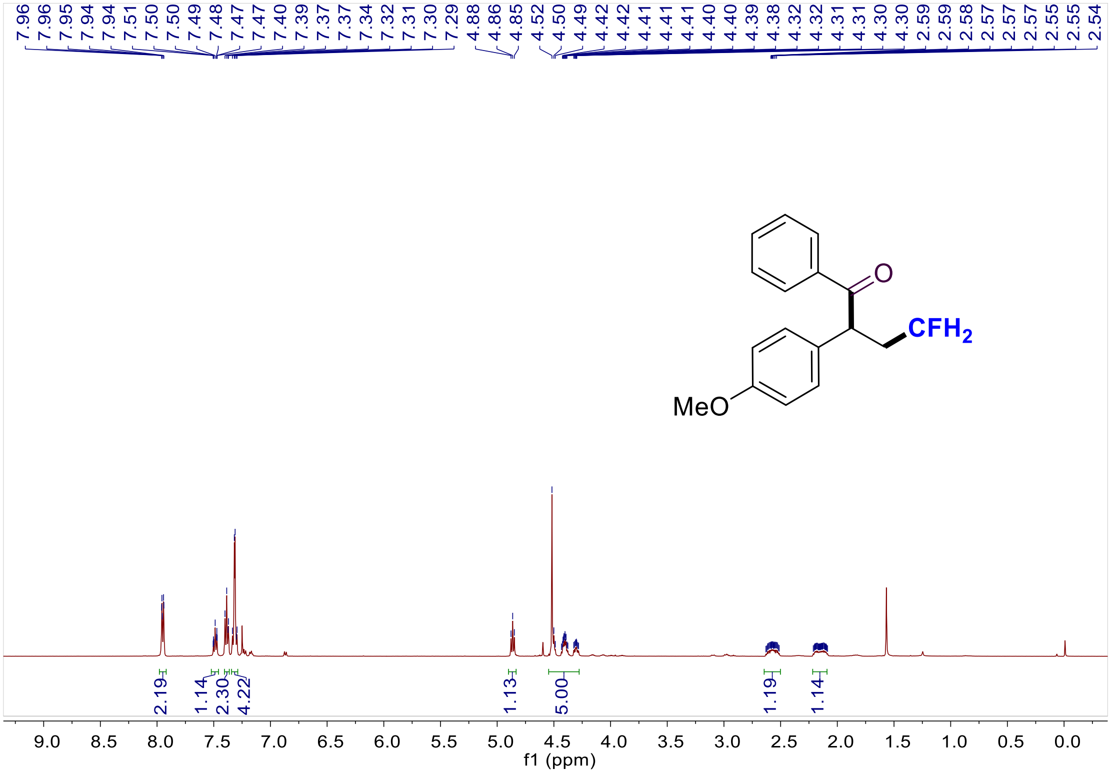

^{13}C NMR (151 MHz, $CDCl_3$) spectrum for **8**.

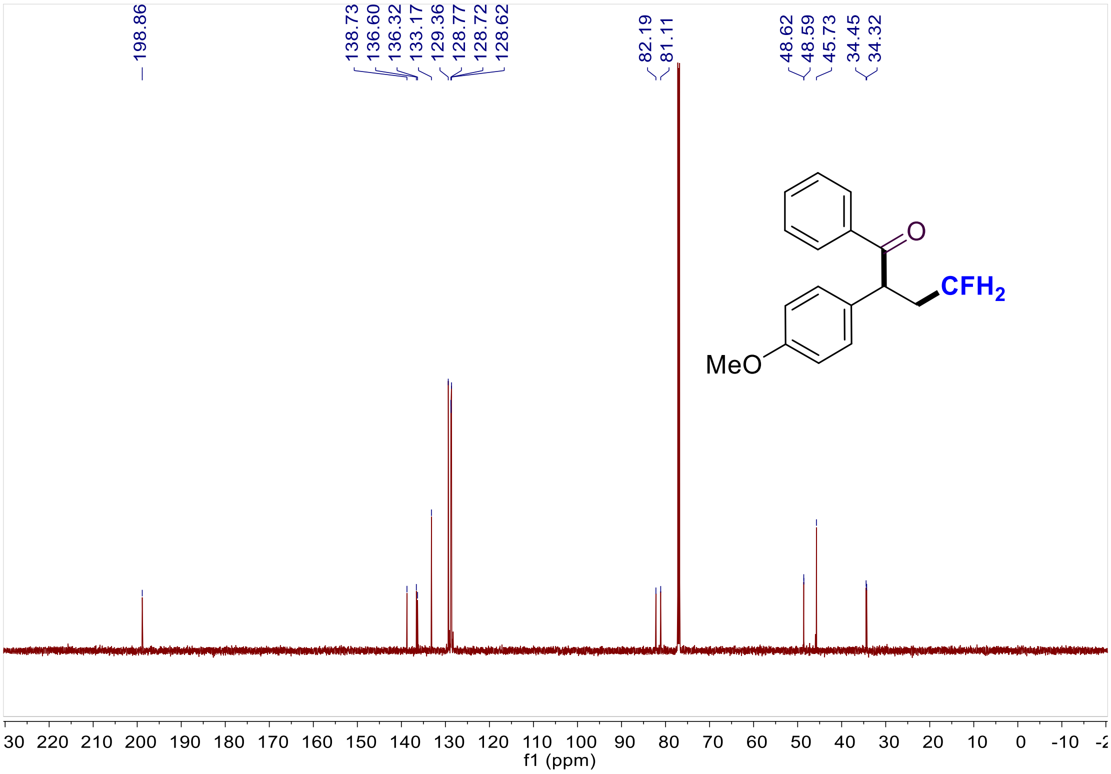

^{19}F NMR (565 MHz, $CDCl_3$) spectrum for **8**.

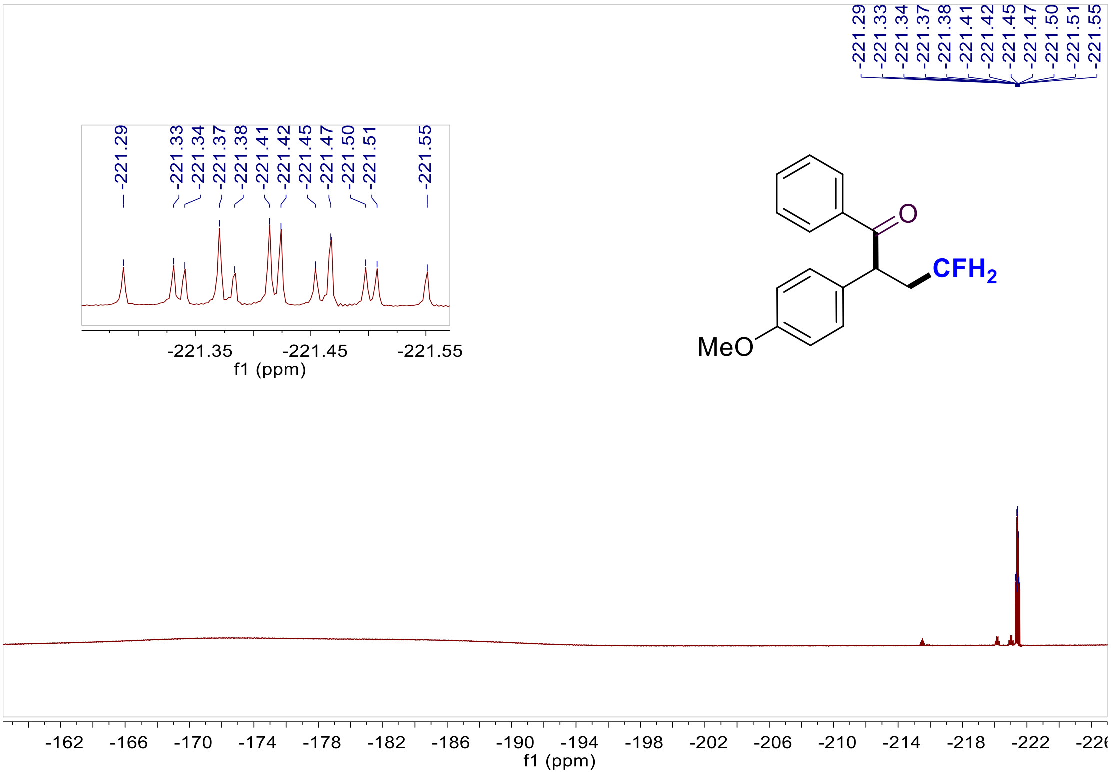

^{1}H NMR (500 MHz, $CDCl_3$) spectrum for **9**.

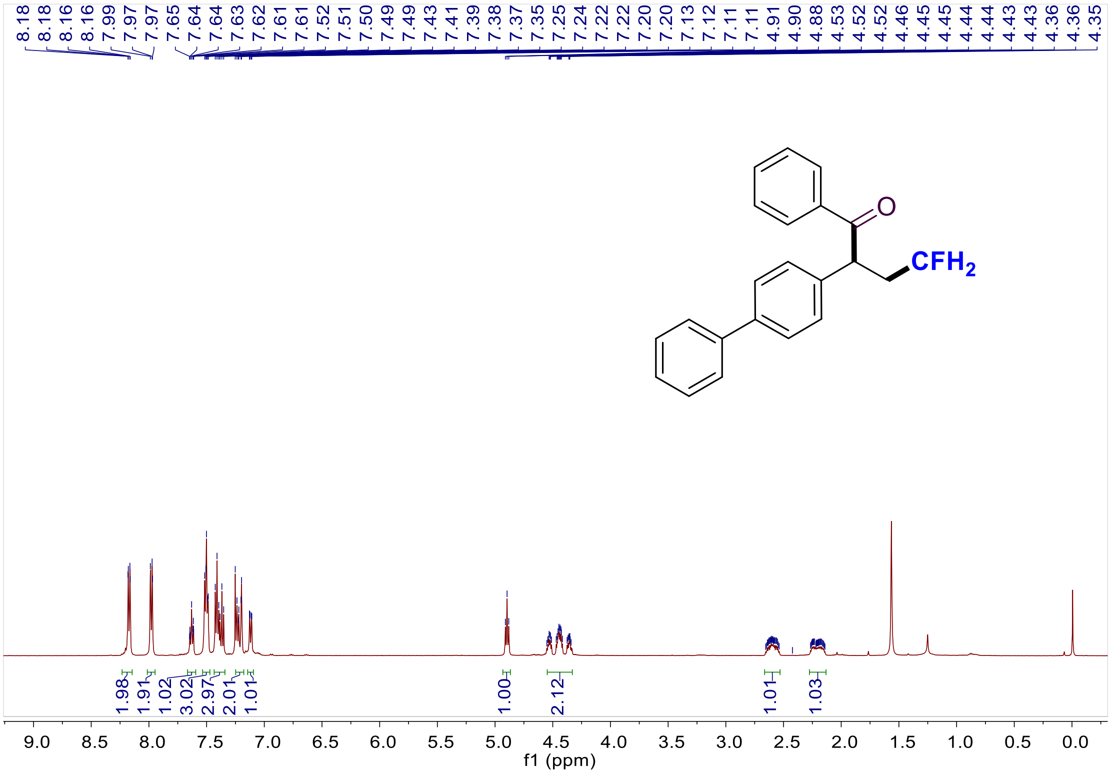

^{13}C NMR (151 MHz, $CDCl_3$) spectrum for **9**.

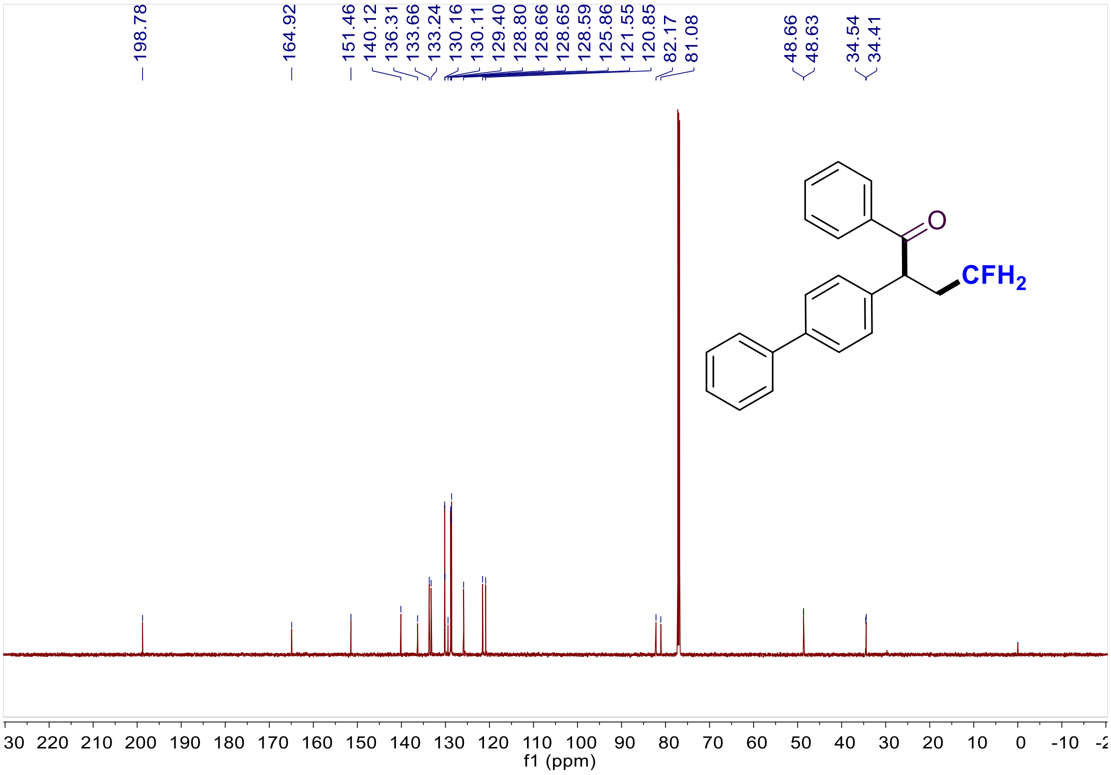

^{19}F NMR (565 MHz, $CDCl_3$) spectrum for **9**.

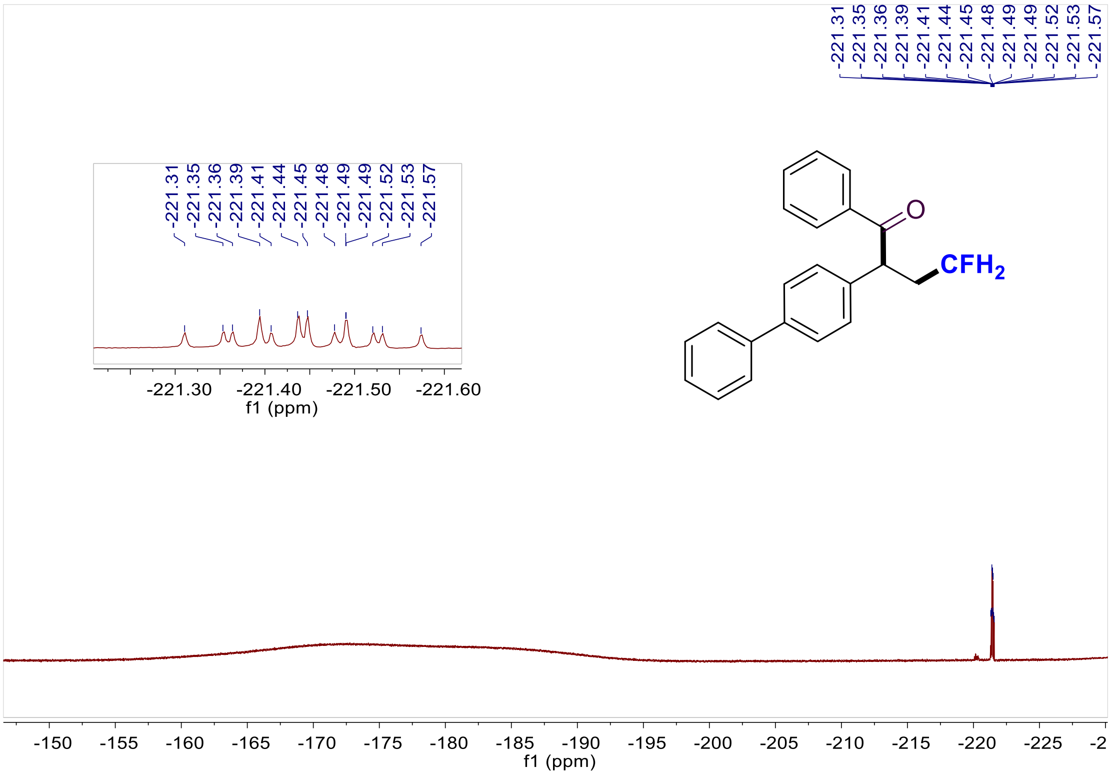

^{1}H NMR (500 MHz, $CDCl_3$) spectrum for **10**.

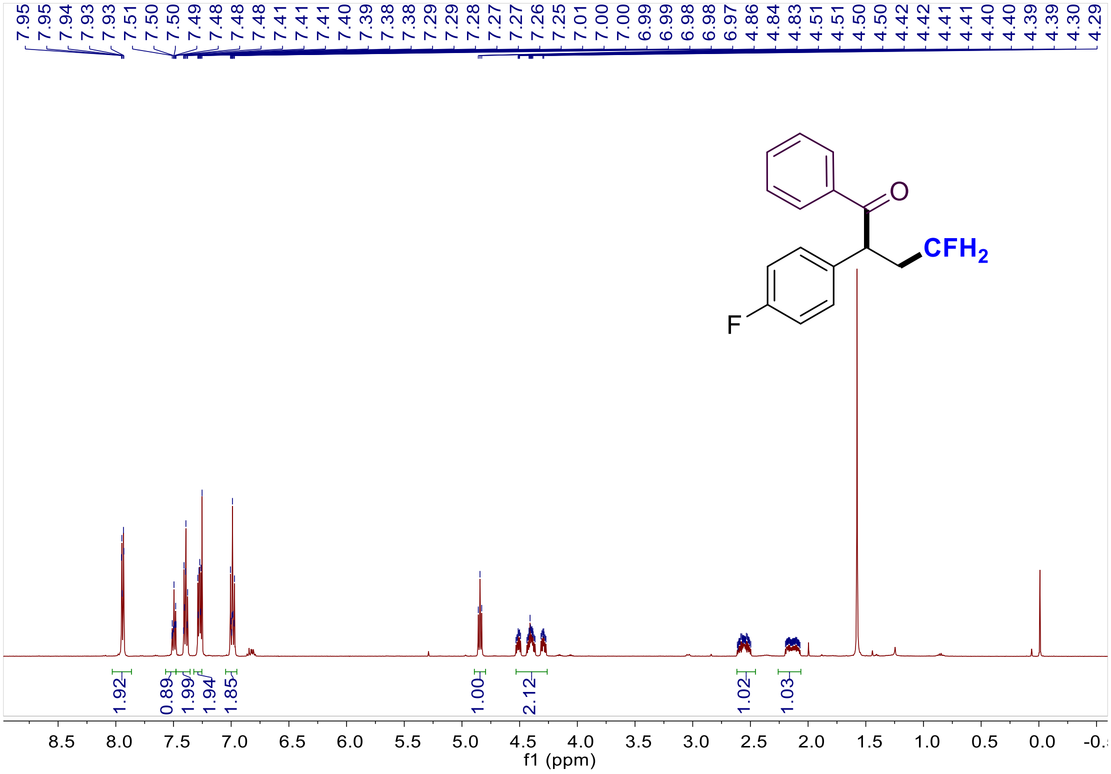

^{13}C NMR (151 MHz, $CDCl_3$) spectrum for **10**.

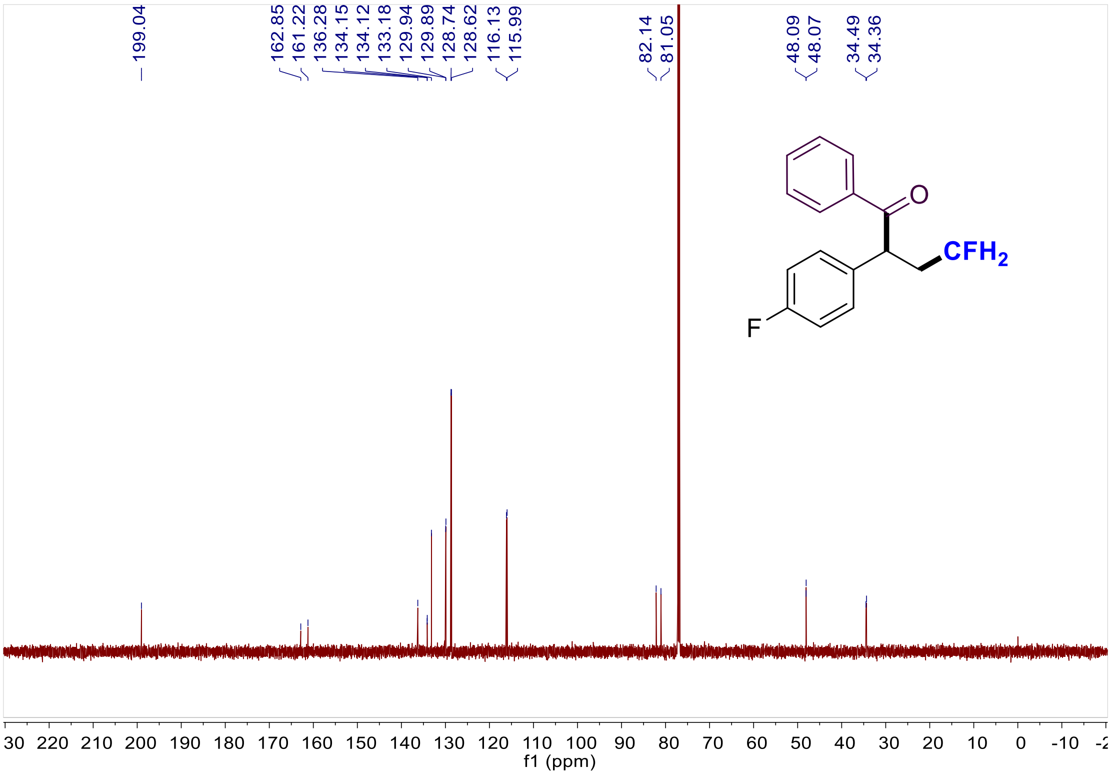

^{19}F NMR (565 MHz, $CDCl_3$) spectrum for **10**.

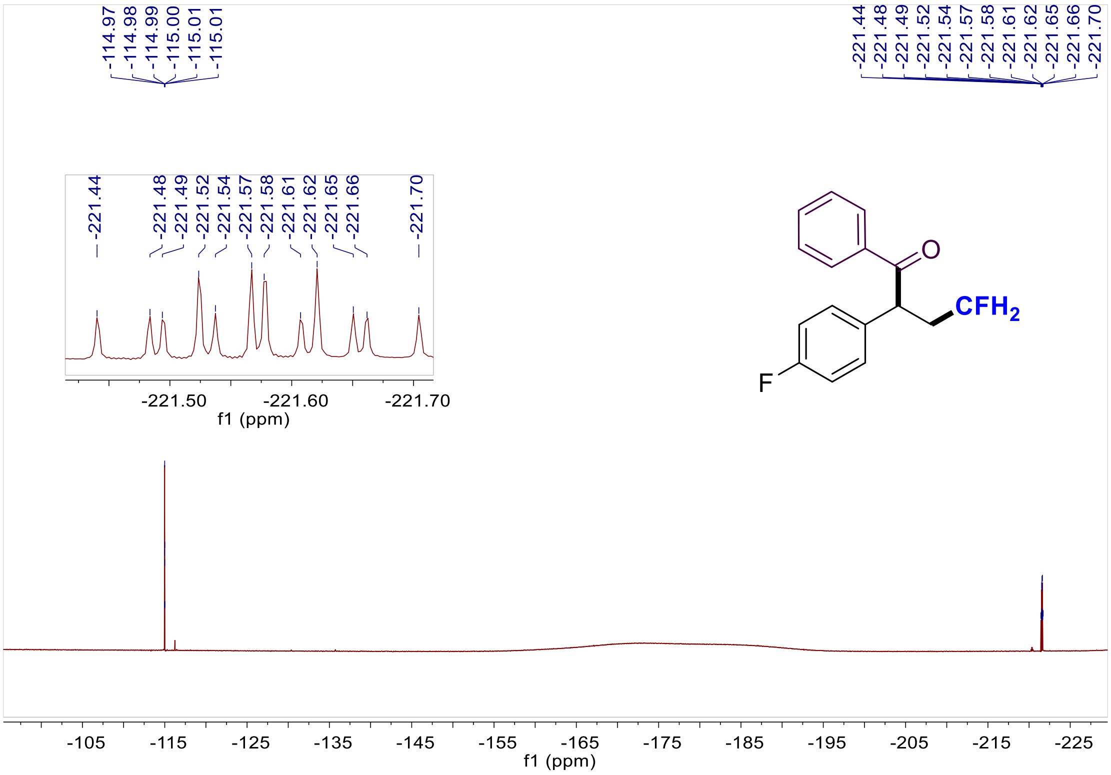

^{1}H NMR (500 MHz, $CDCl_3$) spectrum for **11**.

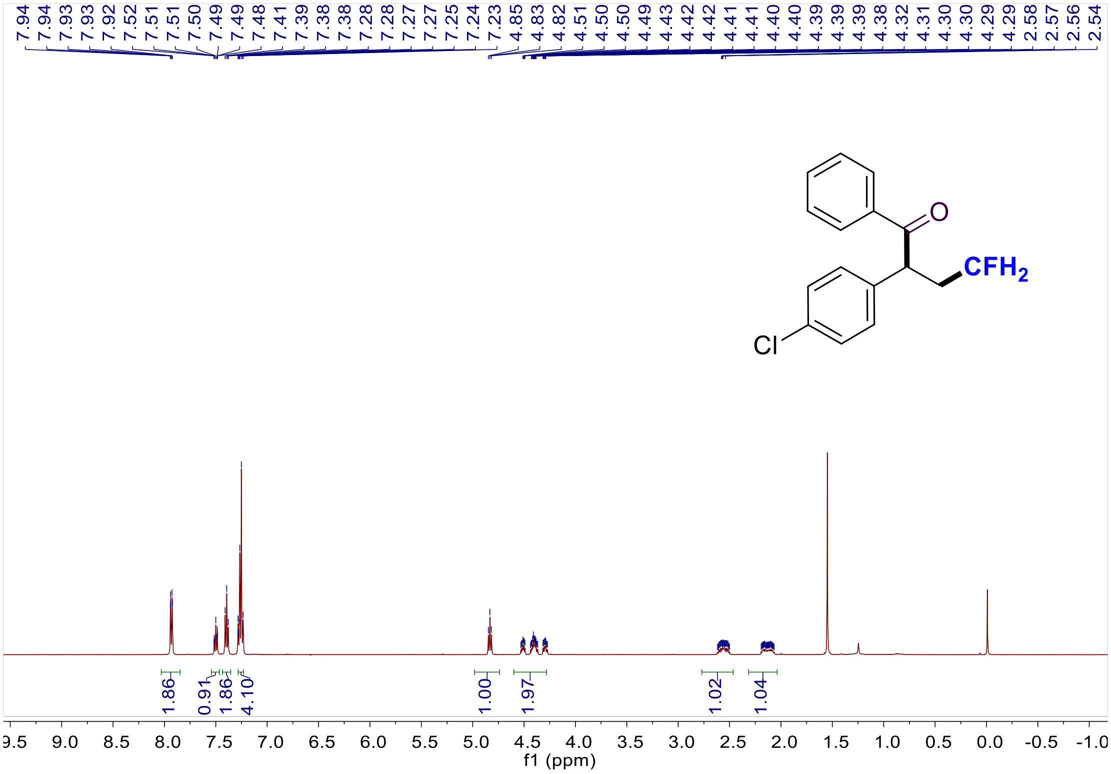

^{13}C NMR (151 MHz, $CDCl_3$) spectrum for **11**.

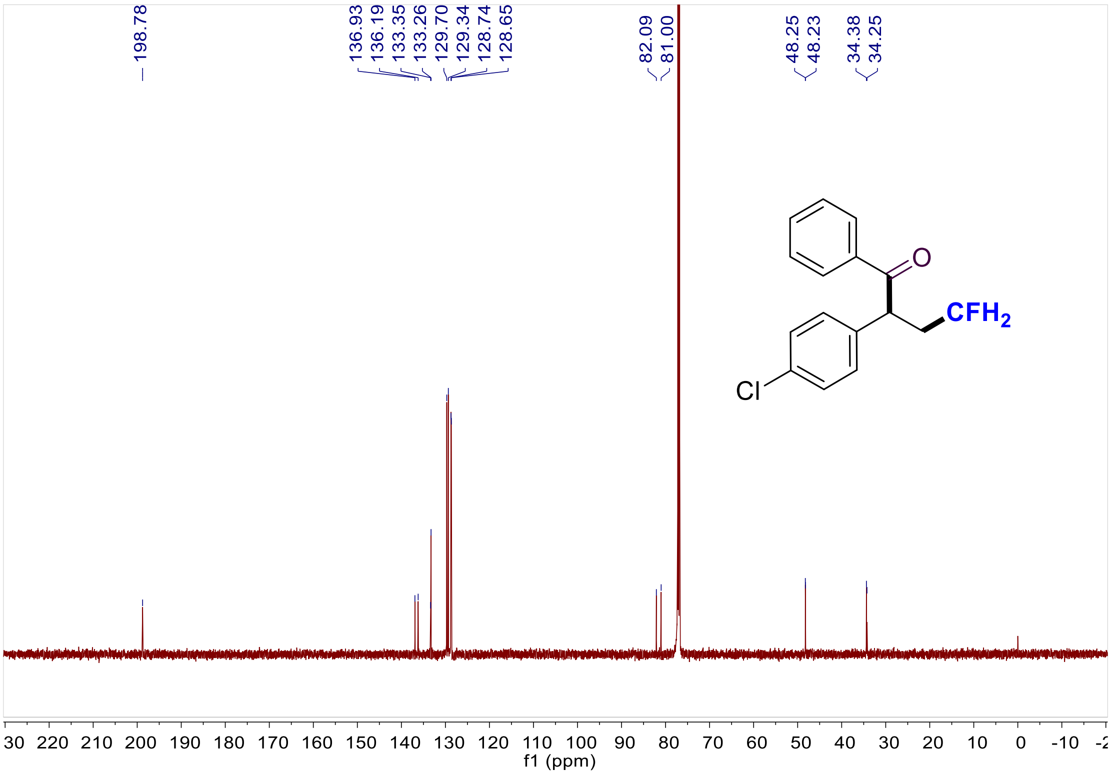

^{19}F NMR (565 MHz, $CDCl_3$) spectrum for **11**.

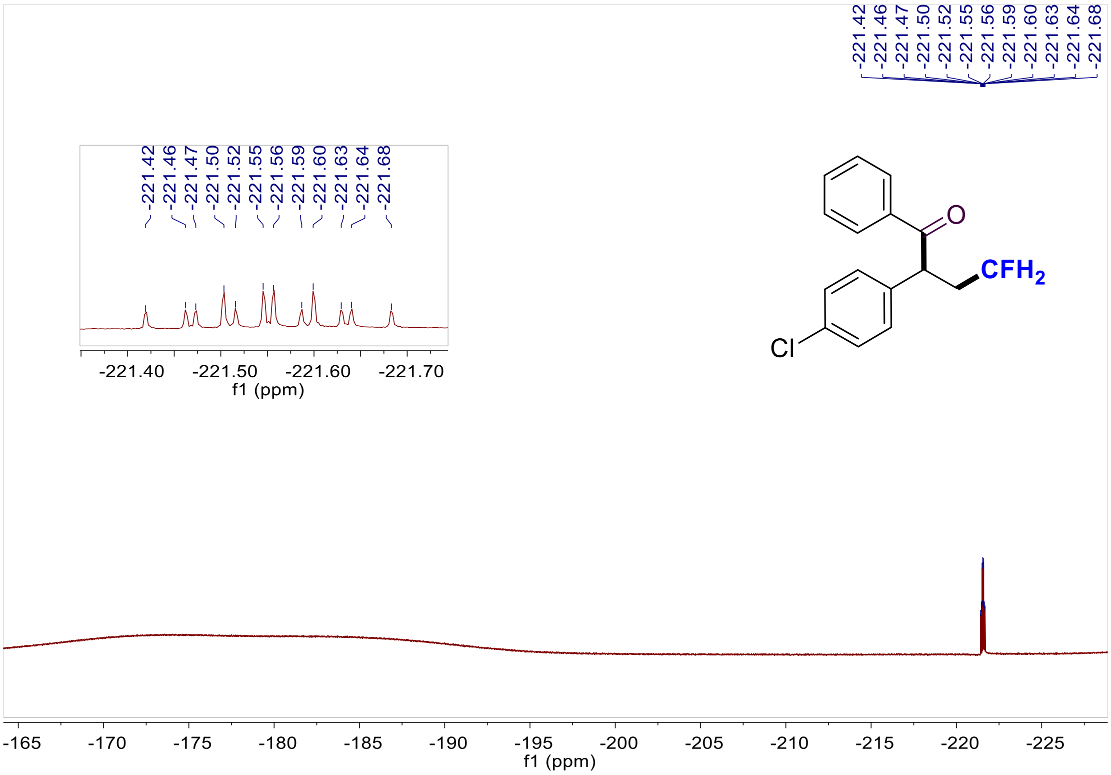

^{1}H NMR (500 MHz, $CDCl_3$) spectrum for **12**.

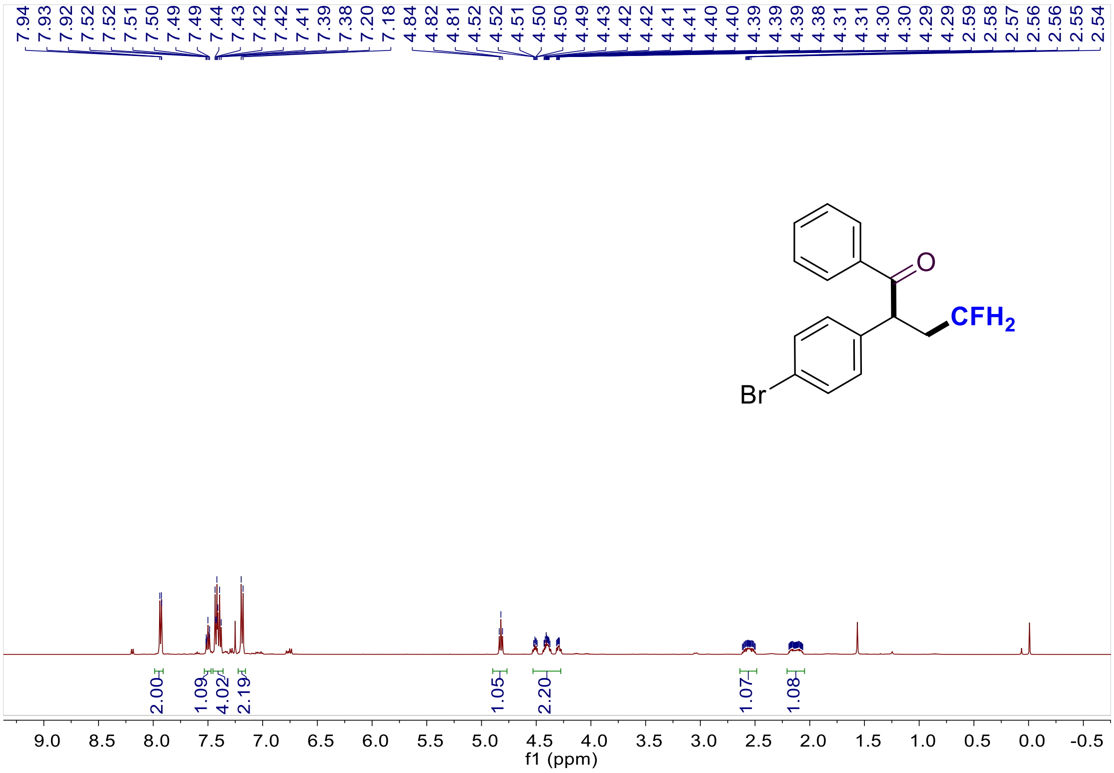

^{13}C NMR (151 MHz, $CDCl_3$) spectrum for **12**.

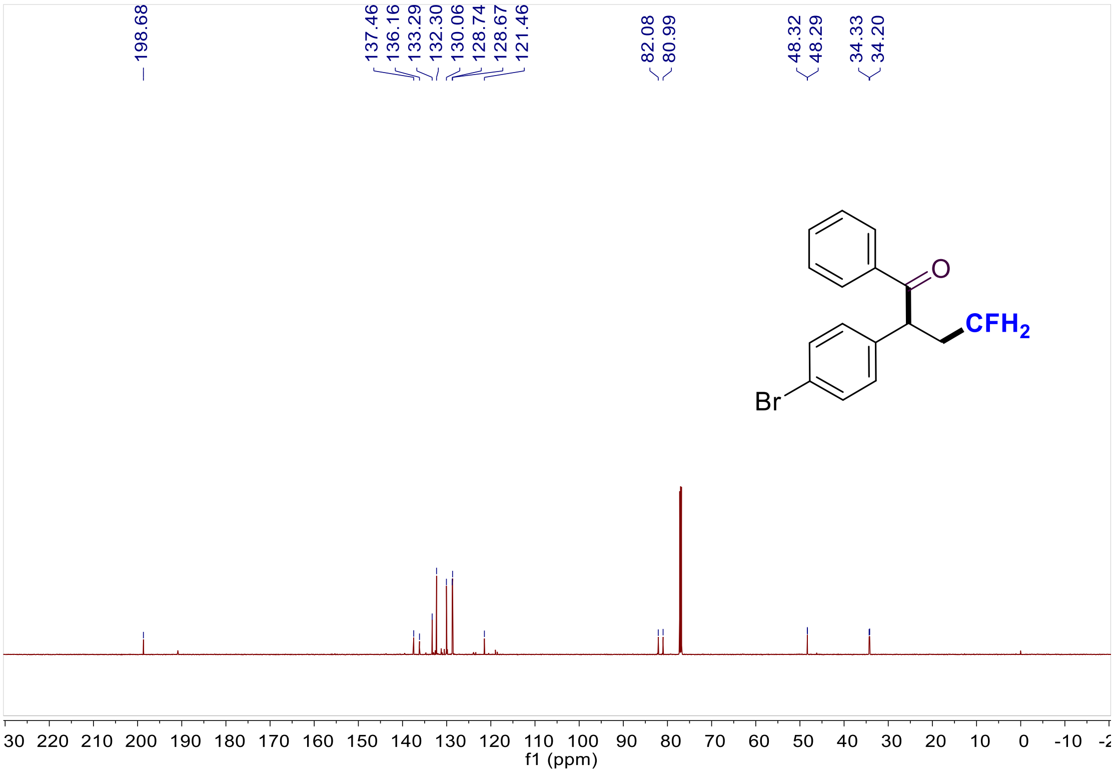

^{19}F NMR (565 MHz, $CDCl_3$) spectrum for **12**.

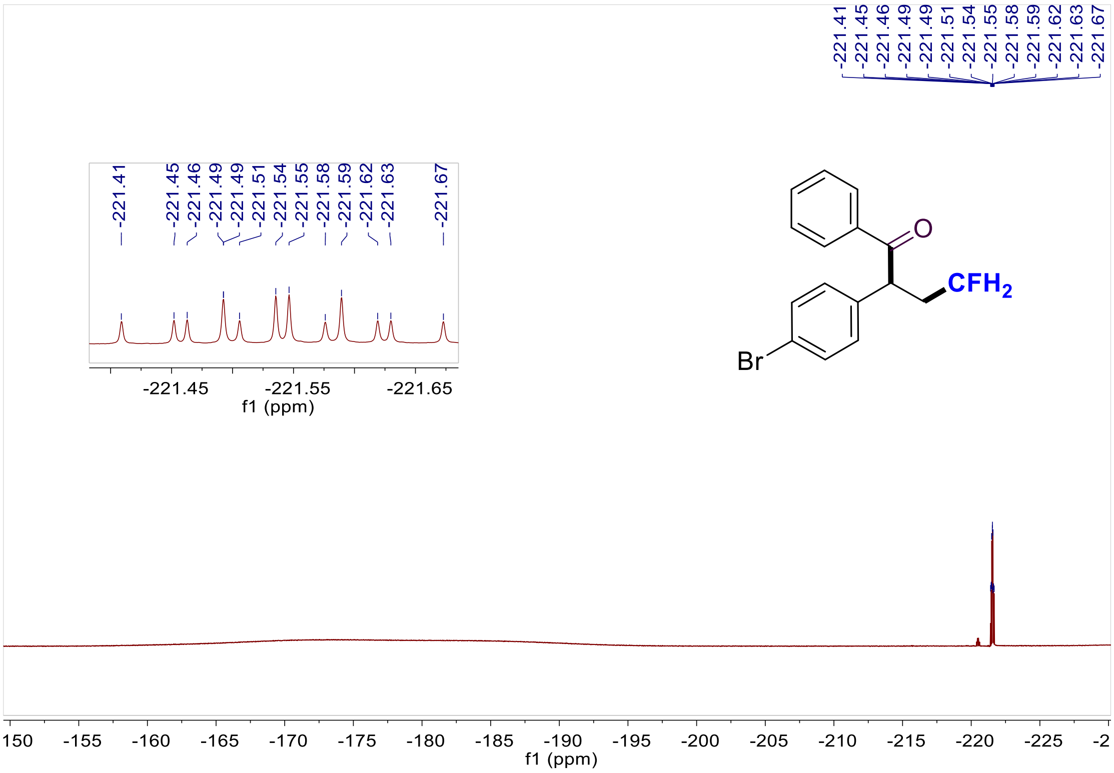

^{1}H NMR (500 MHz, $CDCl_3$) spectrum for **13**.

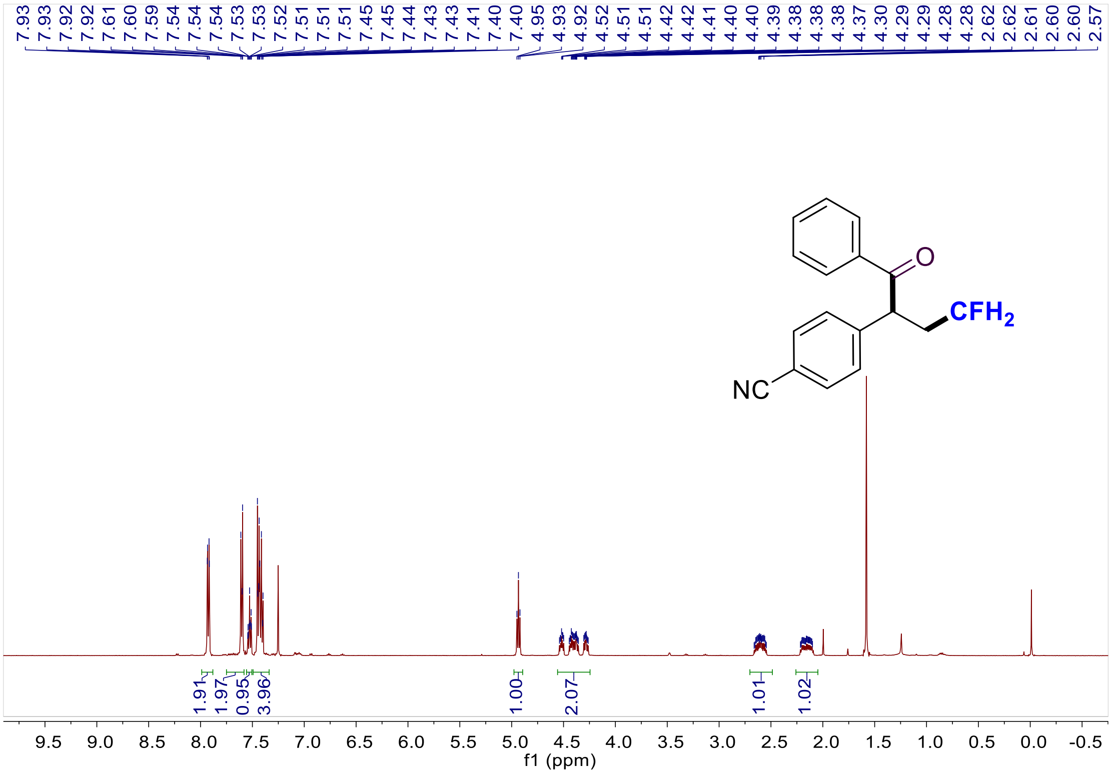

^{13}C NMR (151 MHz, $CDCl_3$) spectrum for **13**.

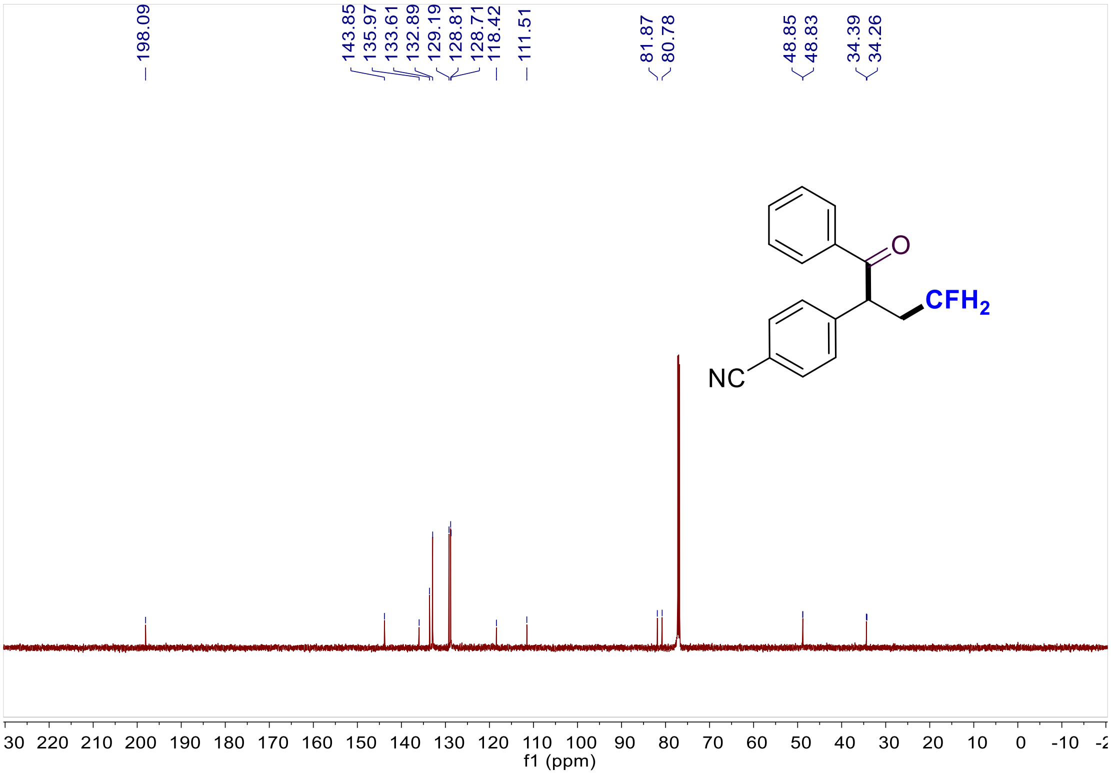

^{19}F NMR (565 MHz, $CDCl_3$) spectrum for **13**.

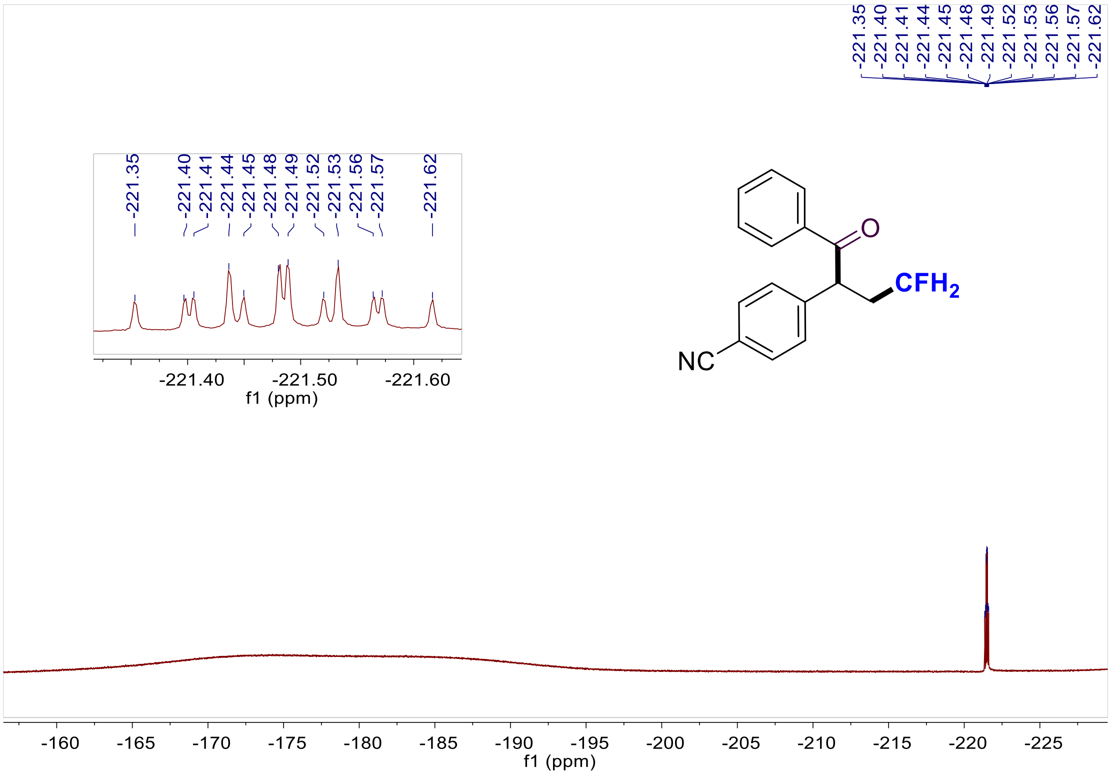

^{1}H NMR (500 MHz, $CDCl_3$) spectrum for **14**.

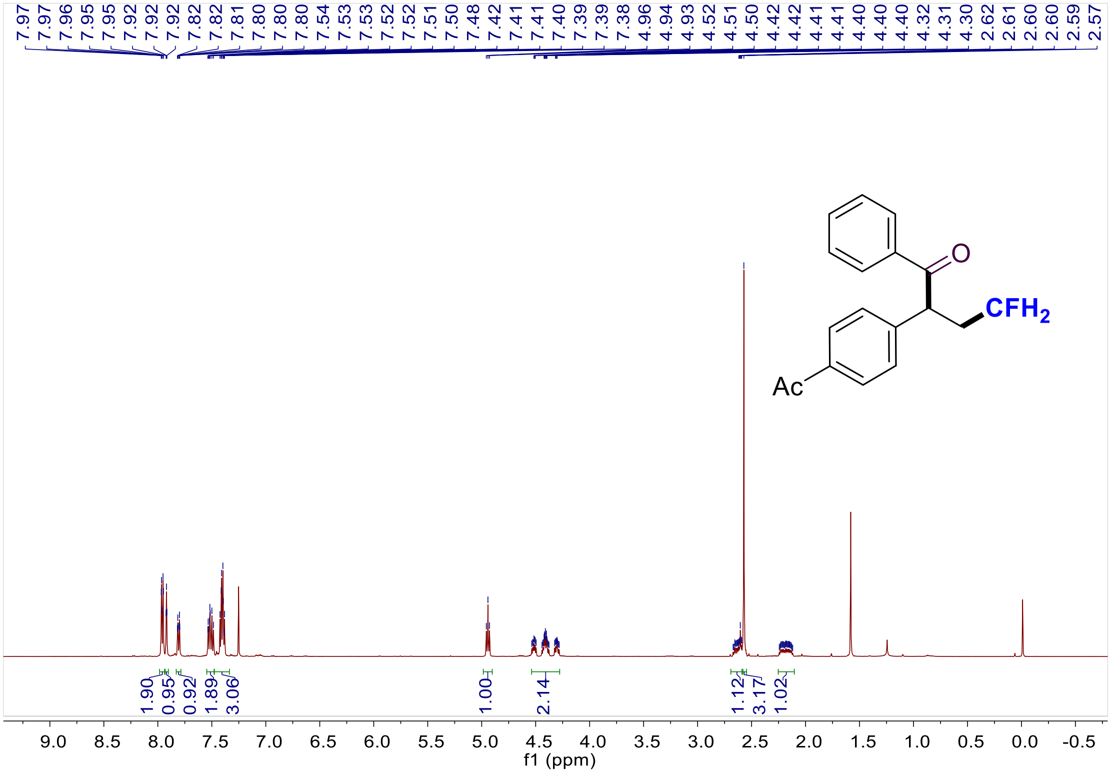

^{13}C NMR (151 MHz, $CDCl_3$) spectrum for **14**.

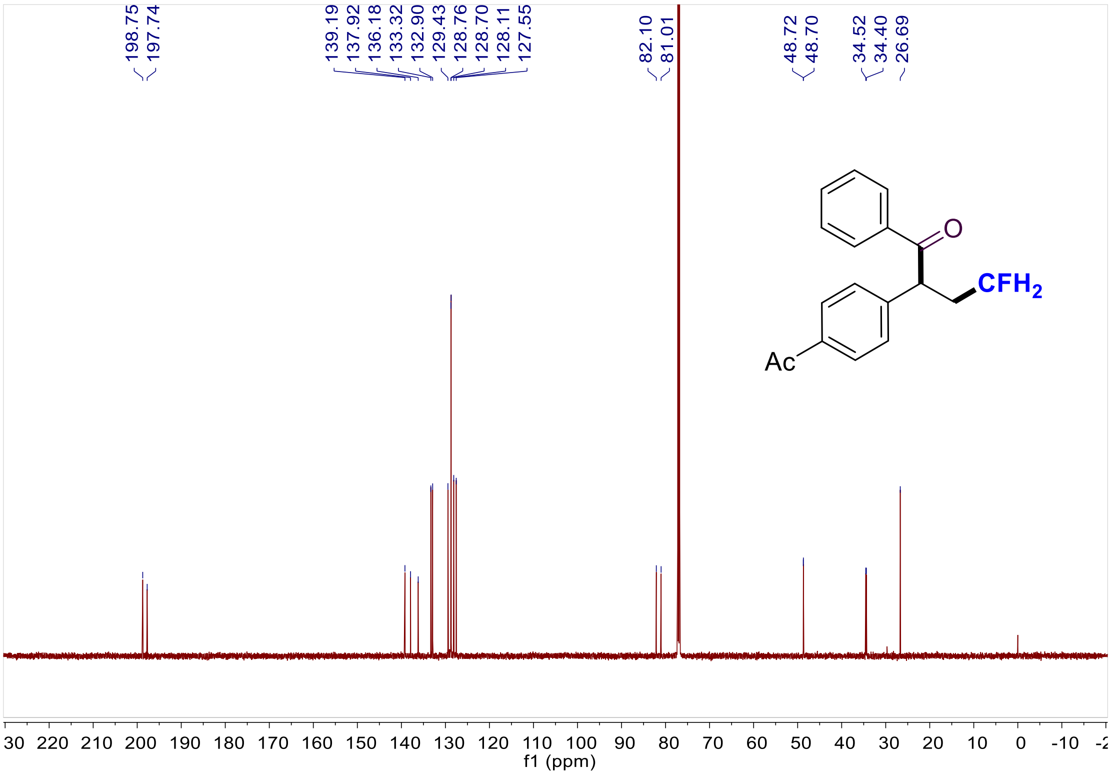

^{19}F NMR (565 MHz, $CDCl_3$) spectrum for **14**.

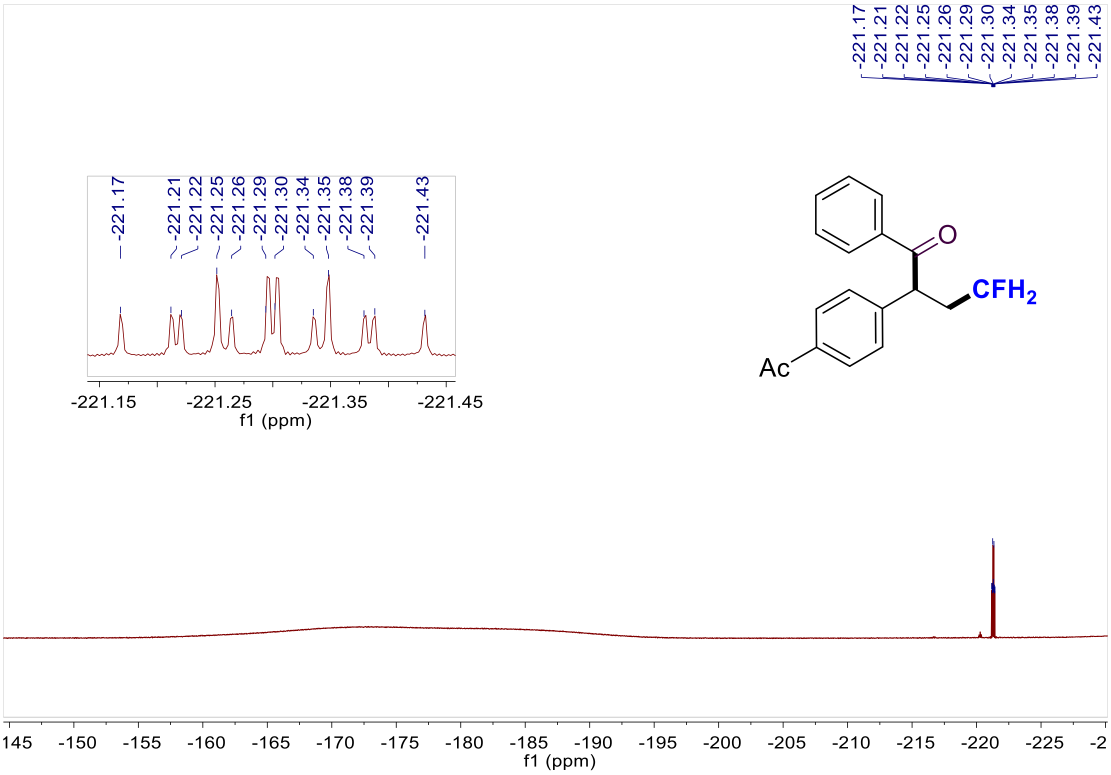

^{1}H NMR (500 MHz, $CDCl_3$) spectrum for **15**.

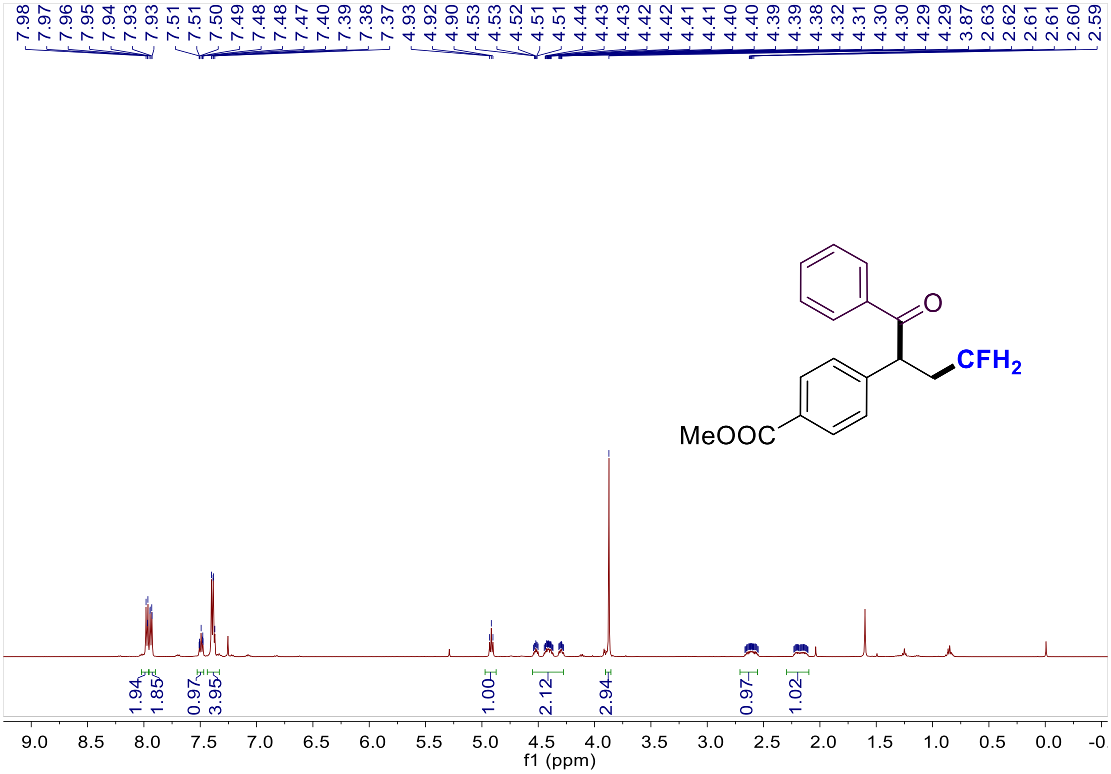

^{13}C NMR (151 MHz, $CDCl_3$) spectrum for **15**.

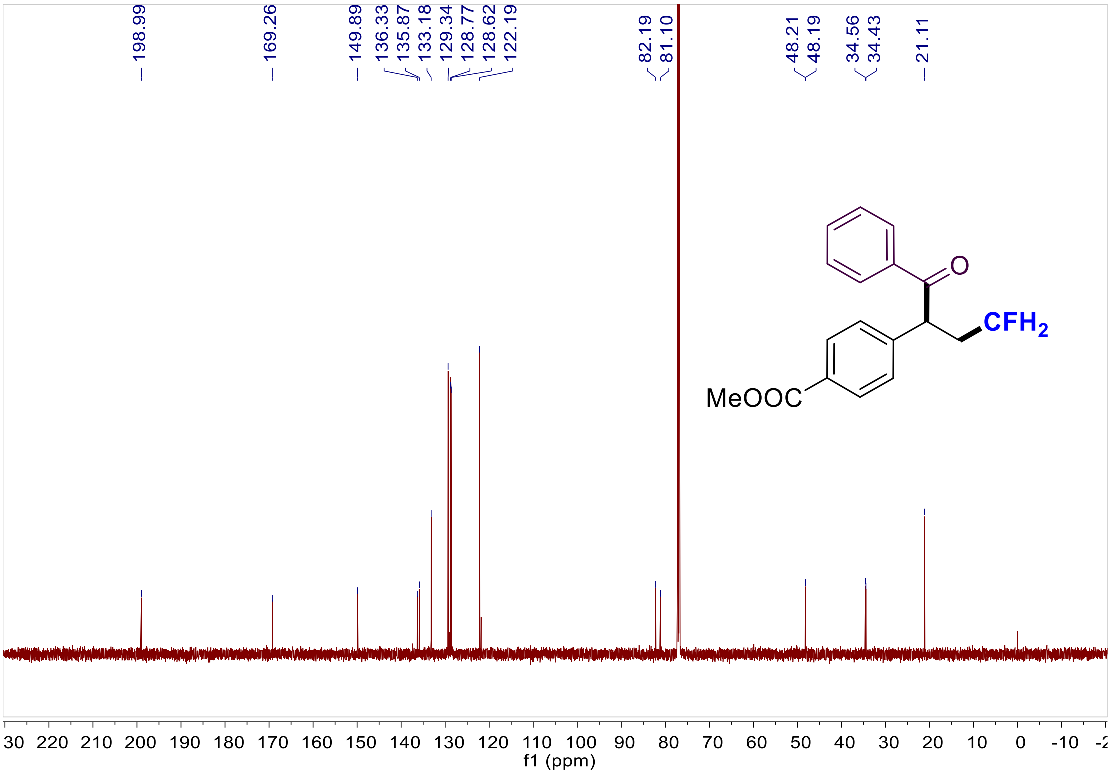

^{19}F NMR (565 MHz, $CDCl_3$) spectrum for **15**.

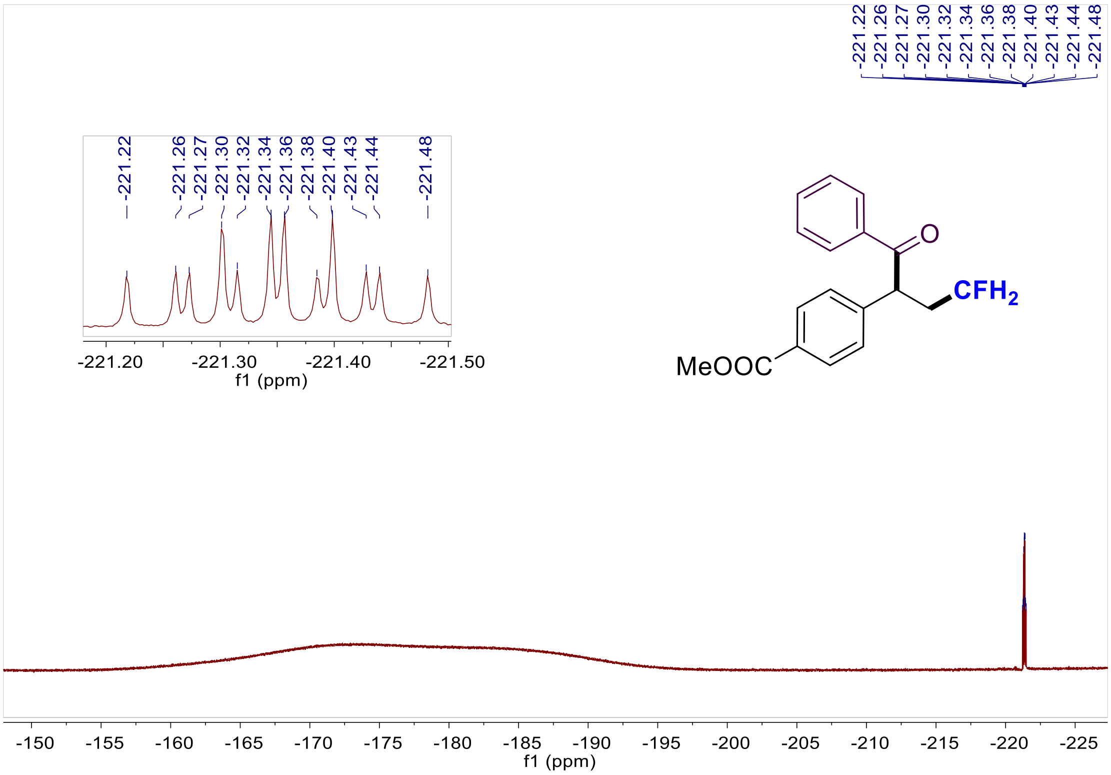

^{1}H NMR (500 MHz, $CDCl_3$) spectrum for **16**.

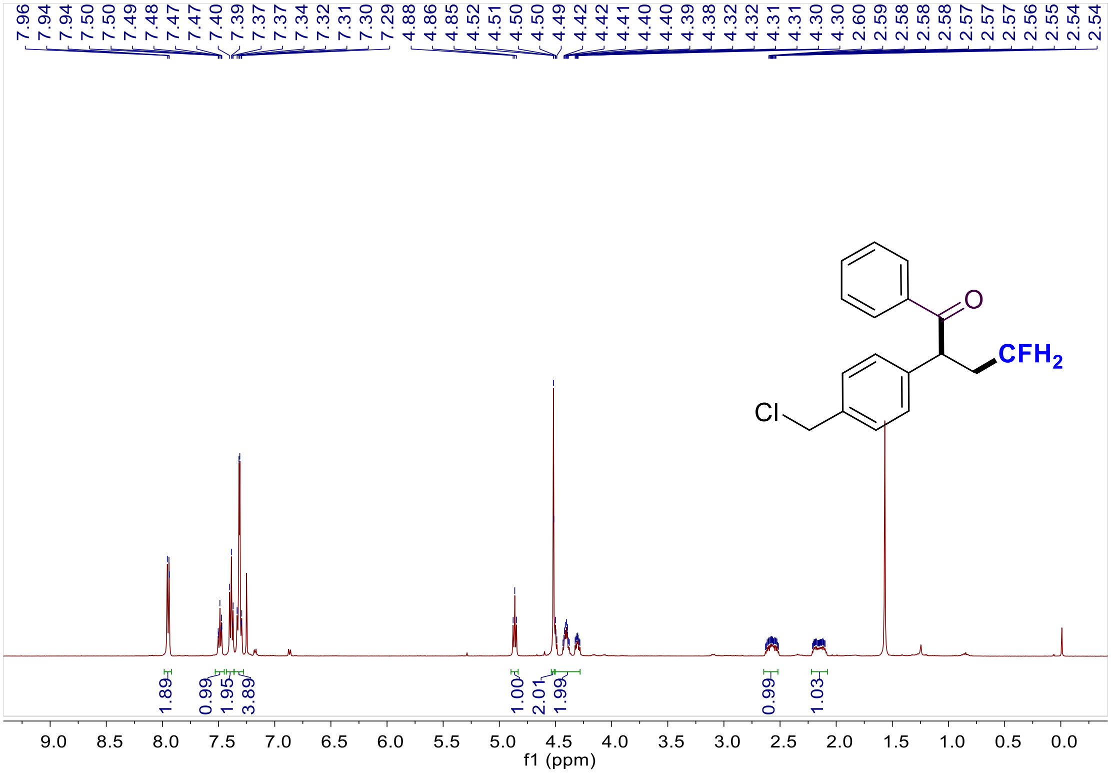

^{13}C NMR (151 MHz, $CDCl_3$) spectrum for **16**.

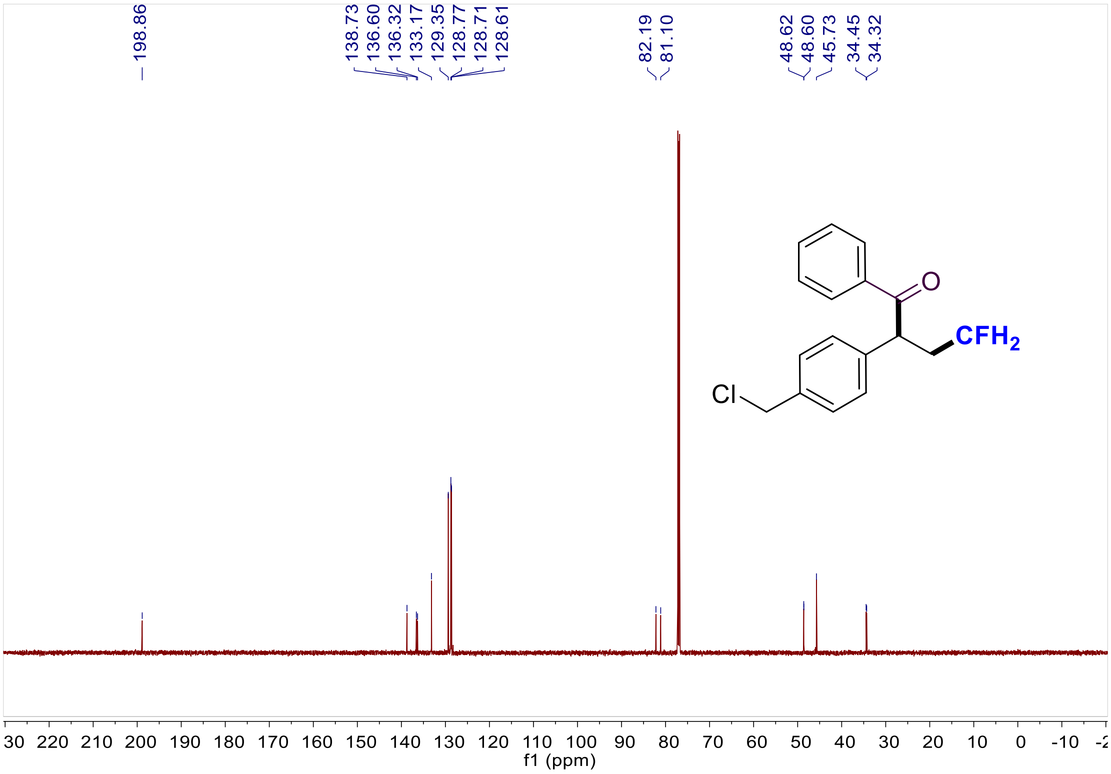

^{19}F NMR (565 MHz, $CDCl_3$) spectrum for **16**.

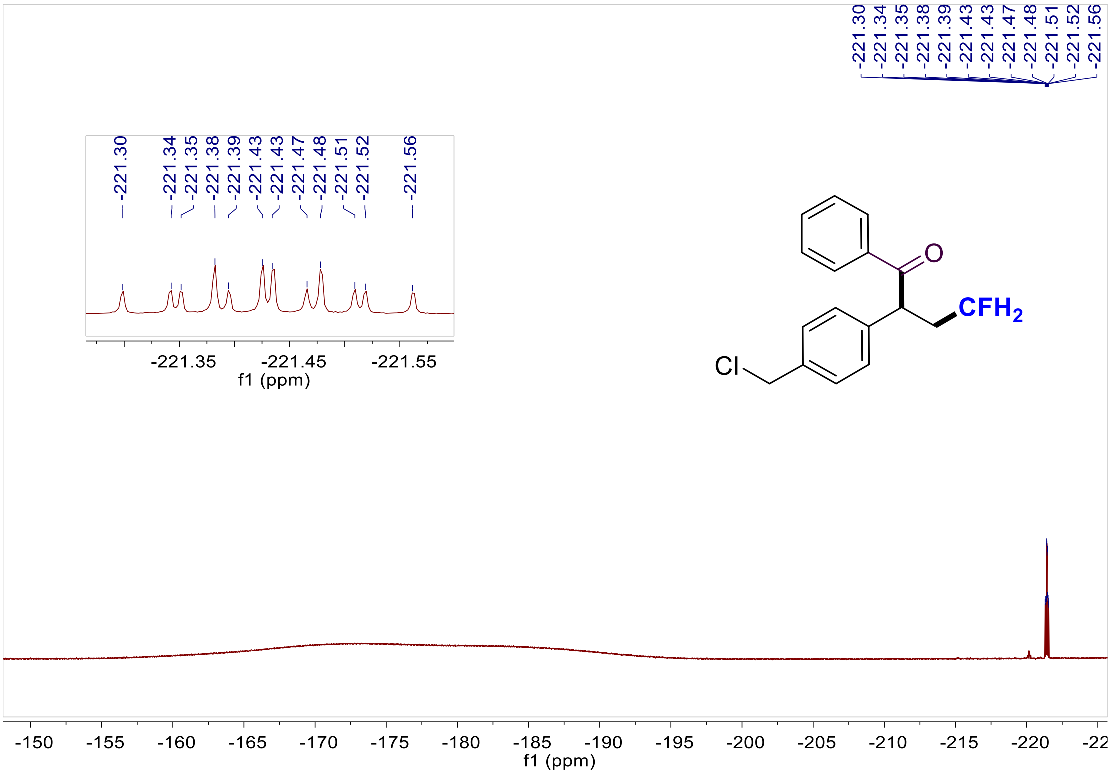

^{1}H NMR (500 MHz, $CDCl_3$) spectrum for **17**.

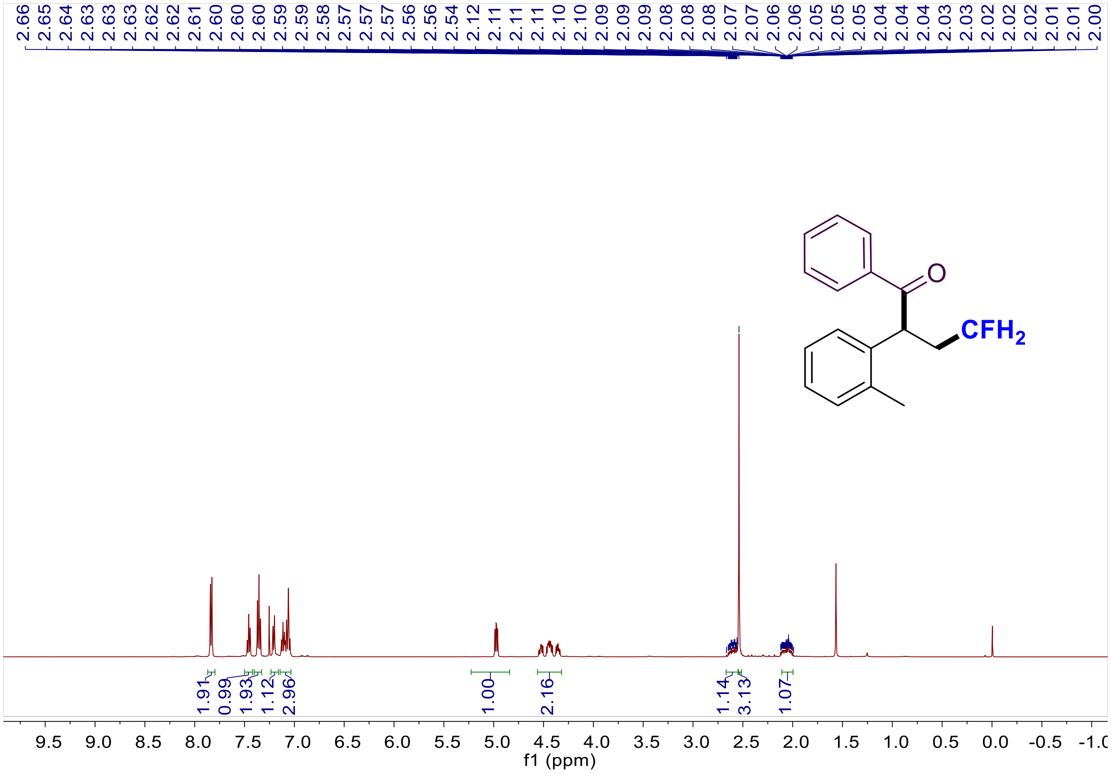

^{13}C NMR (151 MHz, $CDCl_3$) spectrum for **17**.

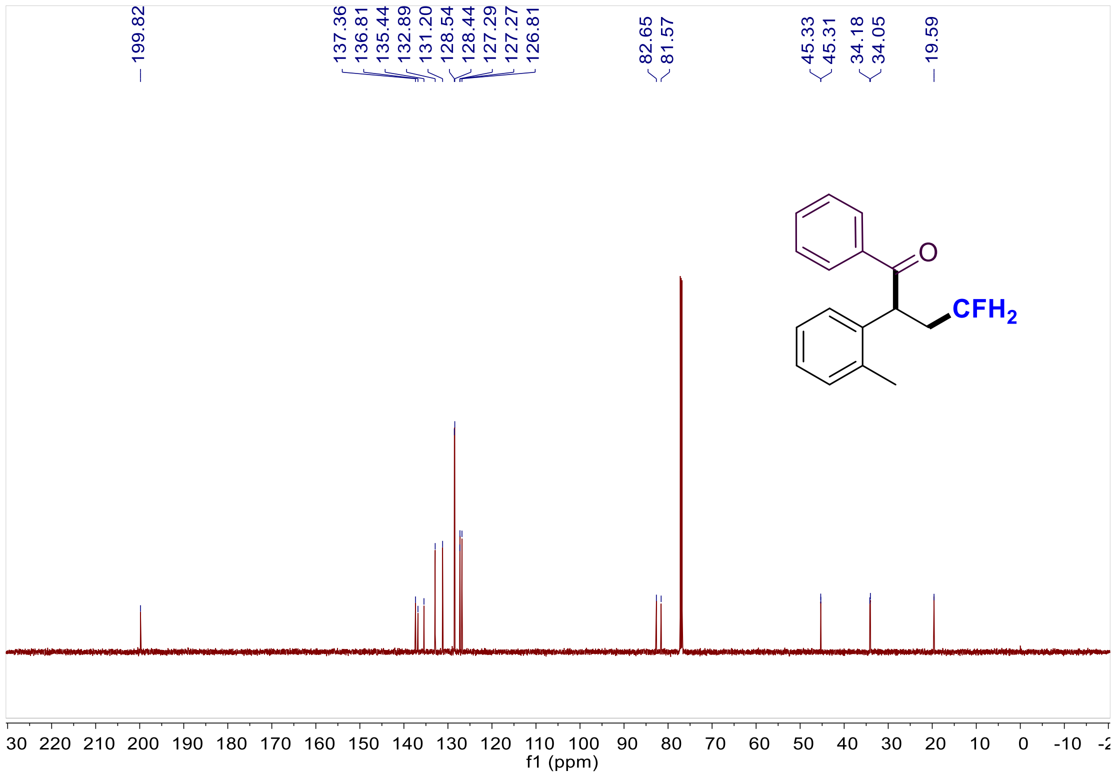

^{19}F NMR (565 MHz, $CDCl_3$) spectrum for **17**.

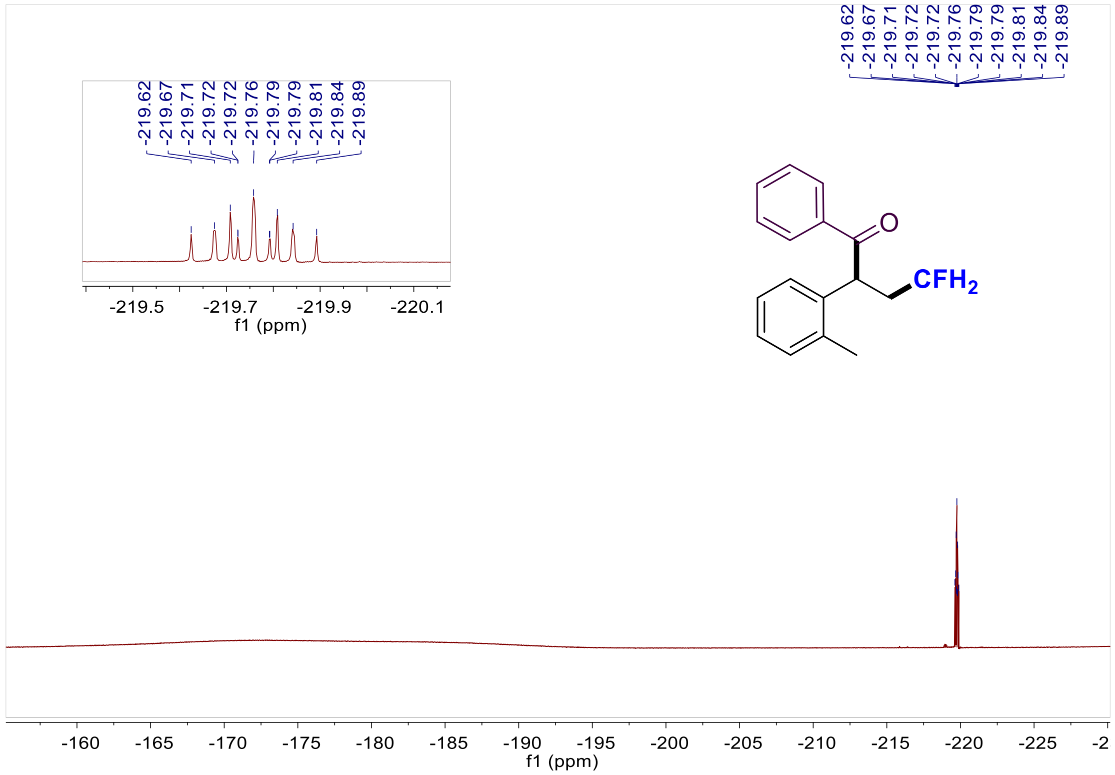

^{1}H NMR (500 MHz, $CDCl_3$) spectrum for **18**.

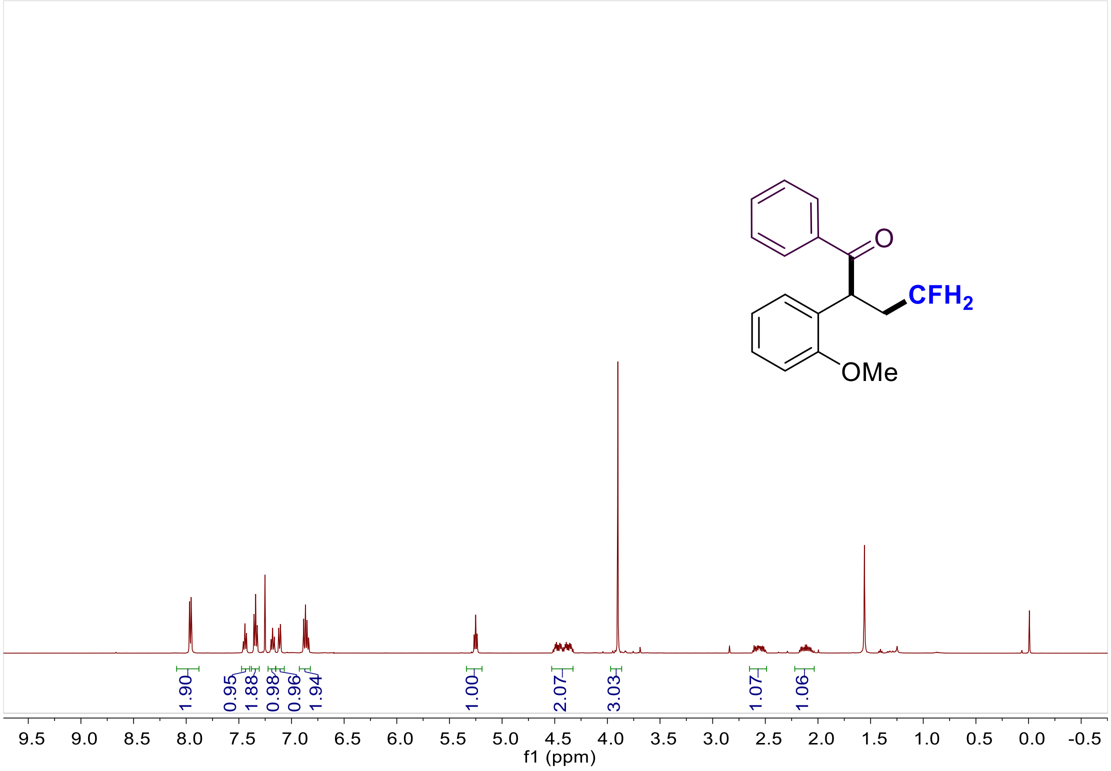

^{13}C NMR (151 MHz, $CDCl_3$) spectrum for **18**.

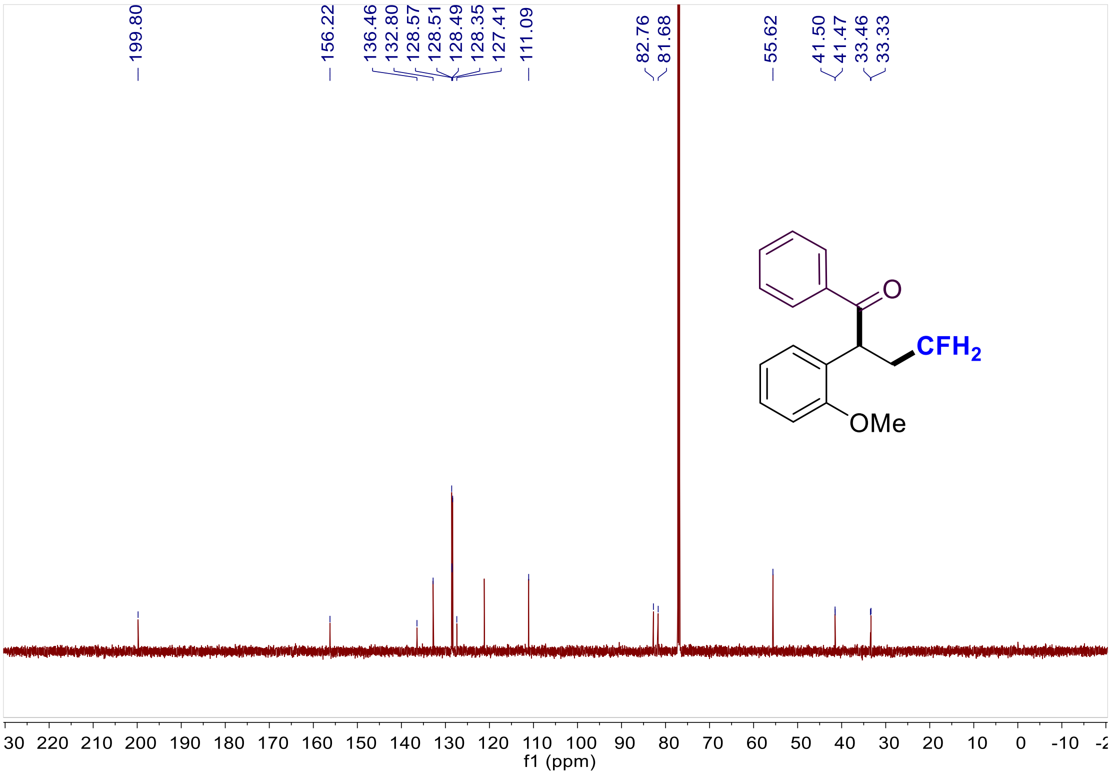

^{19}F NMR (565 MHz, $CDCl_3$) spectrum for **18**.

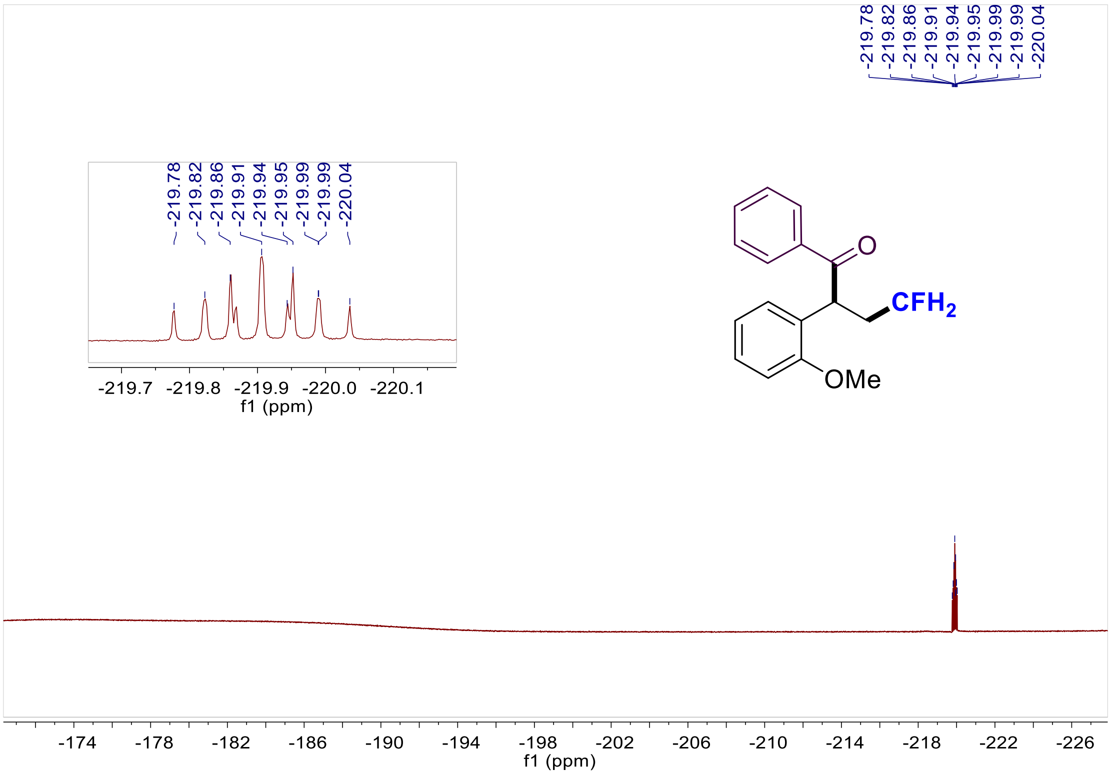

^{1}H NMR (500 MHz, $CDCl_3$) spectrum for **19**.

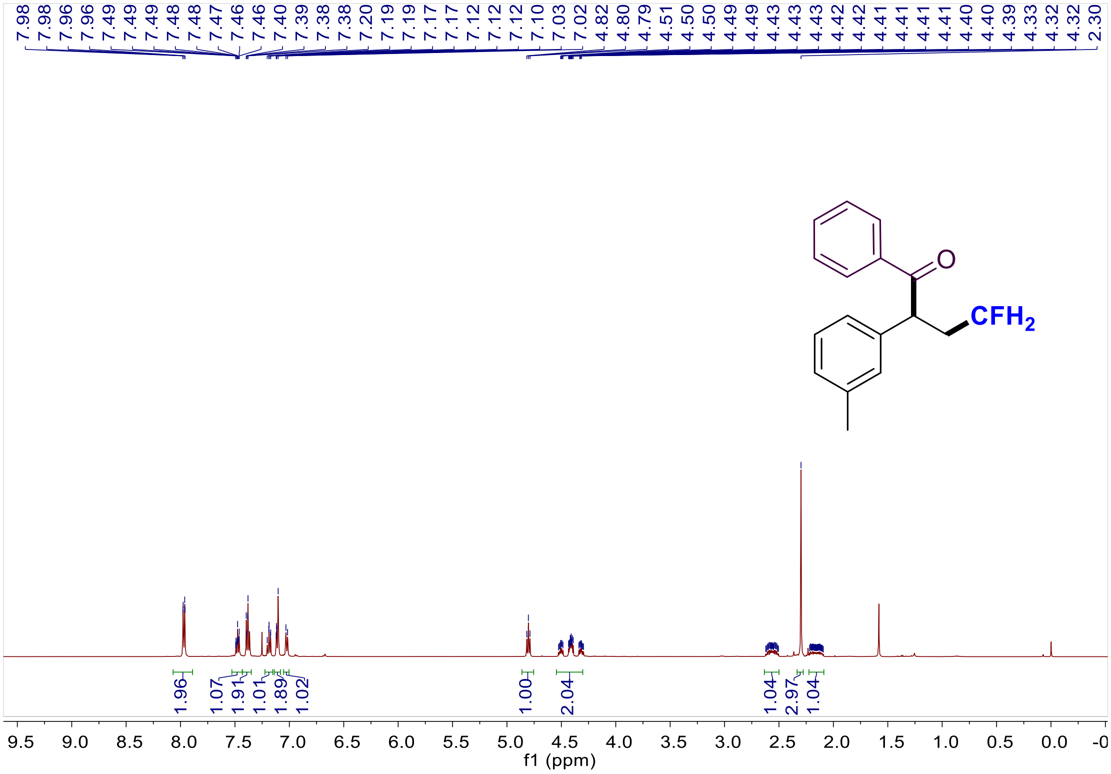

^{13}C NMR (151 MHz, $CDCl_3$) spectrum for **19**.

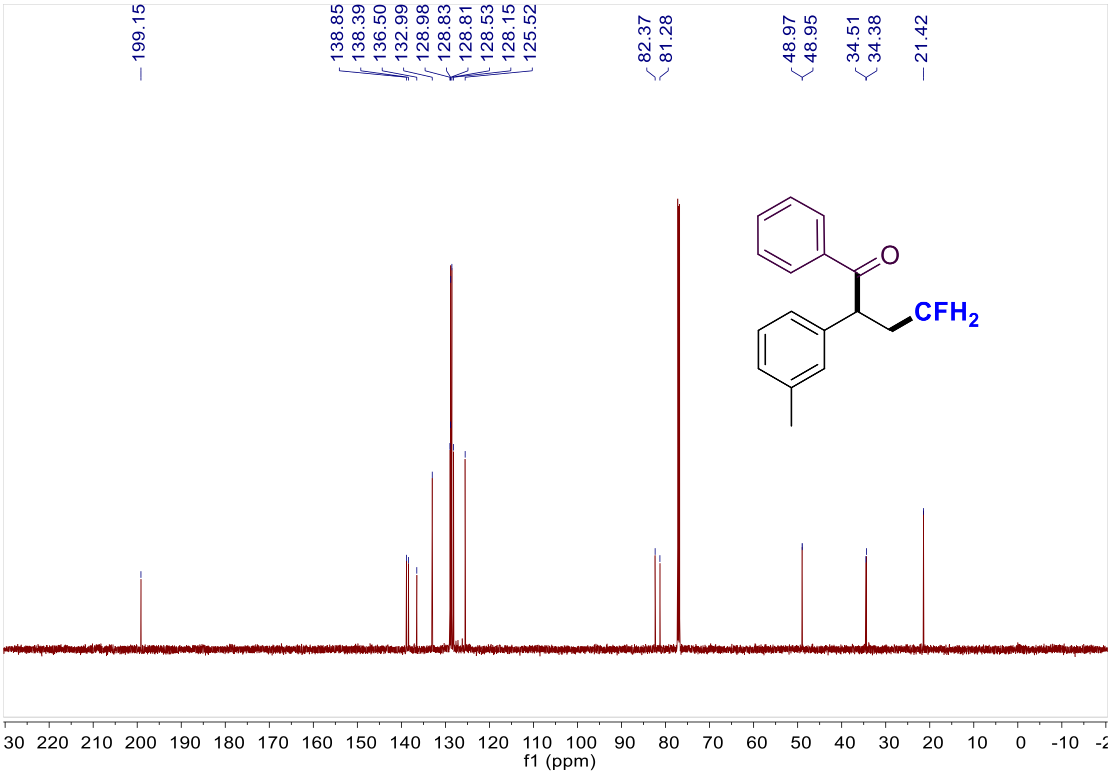

^{19}F NMR (565 MHz, $CDCl_3$) spectrum for **19**.

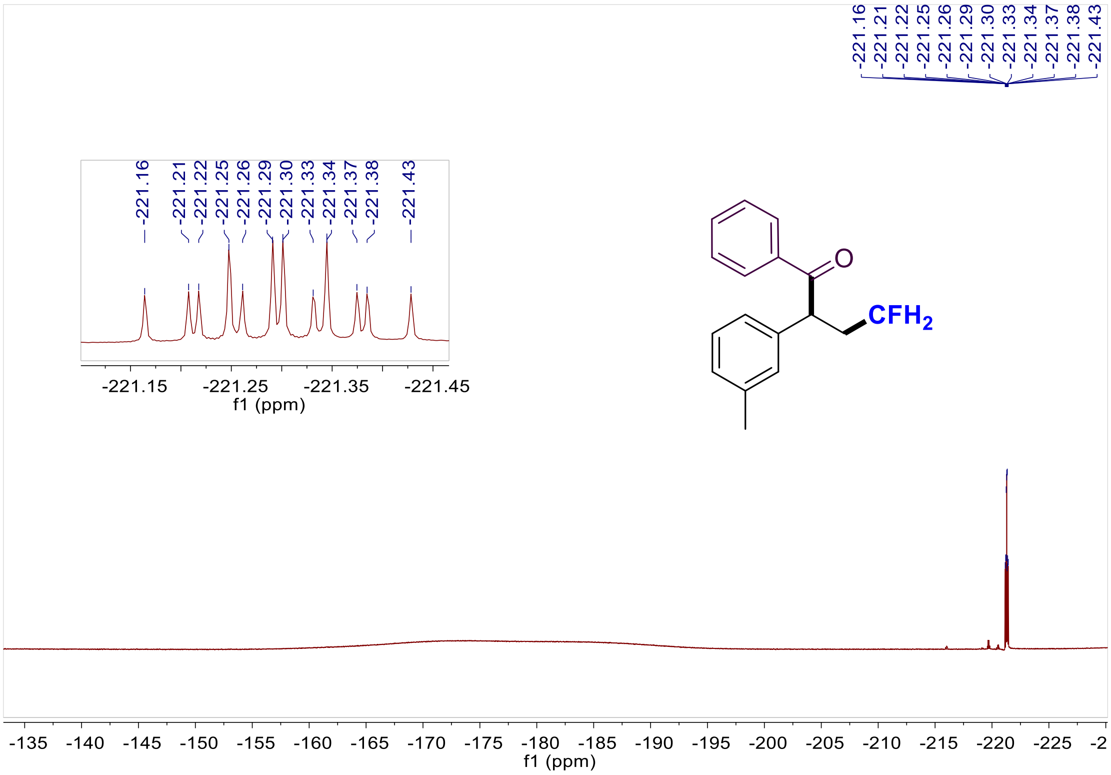

^{1}H NMR (500 MHz, $CDCl_3$) spectrum for **20**.

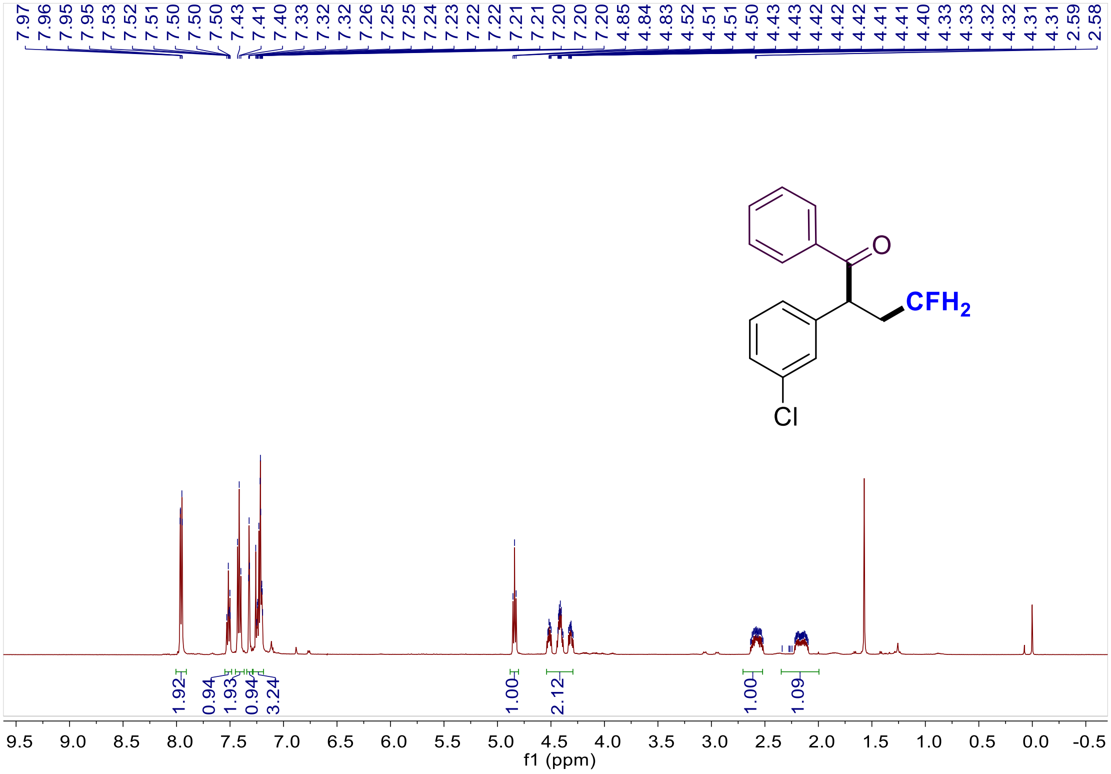

^{13}C NMR (151 MHz, $CDCl_3$) spectrum for **20**.

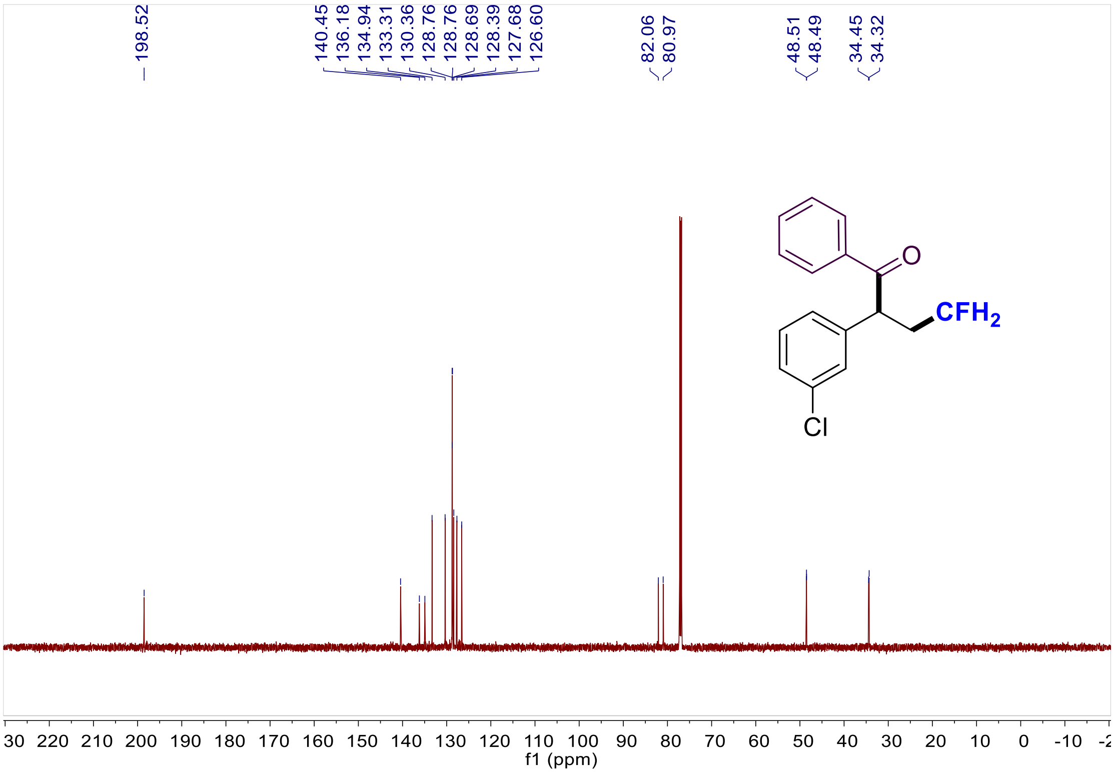

^{19}F NMR (565 MHz, $CDCl_3$) spectrum for **20**.

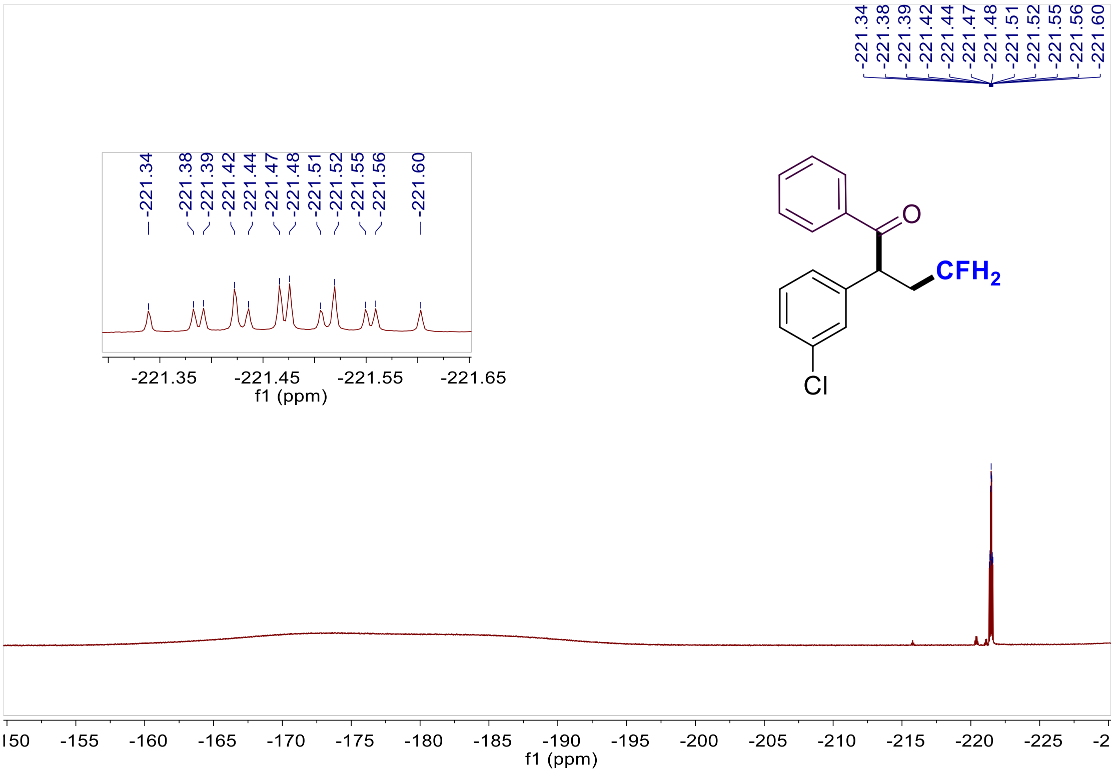

^{1}H NMR (500 MHz, $CDCl_3$) spectrum for **21**.

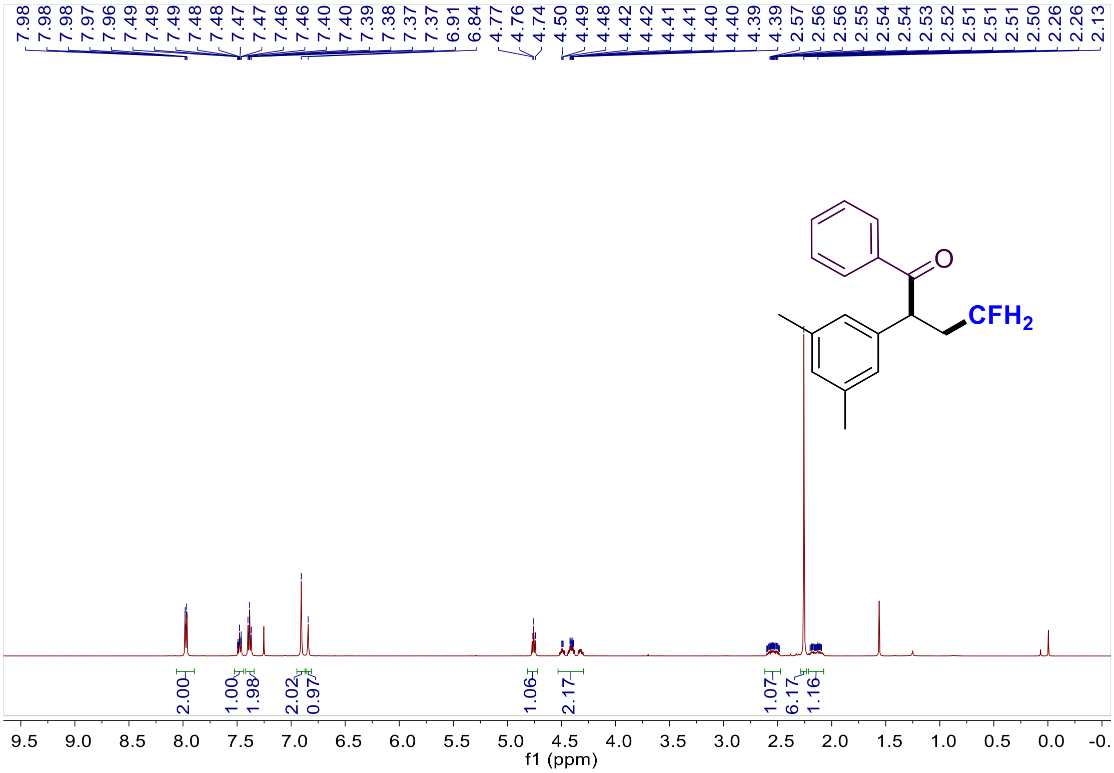

^{13}C NMR (151 MHz, $CDCl_3$) spectrum for **21**.

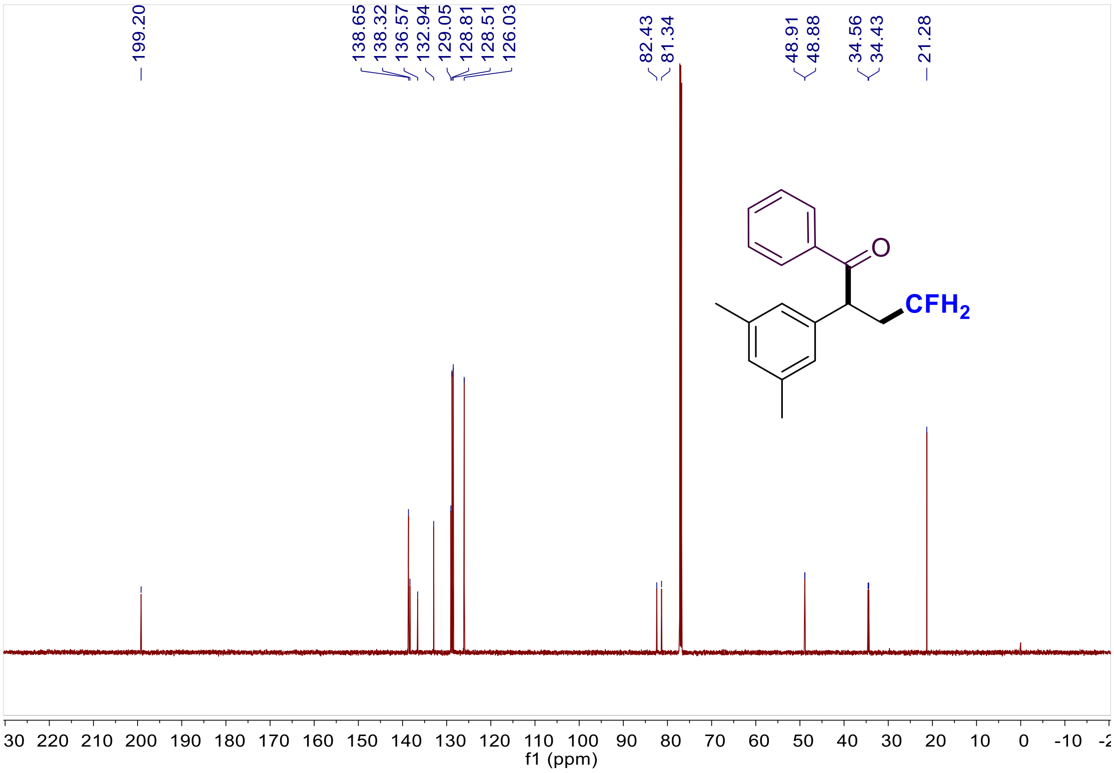

^{19}F NMR (565 MHz, $CDCl_3$) spectrum for **21**.

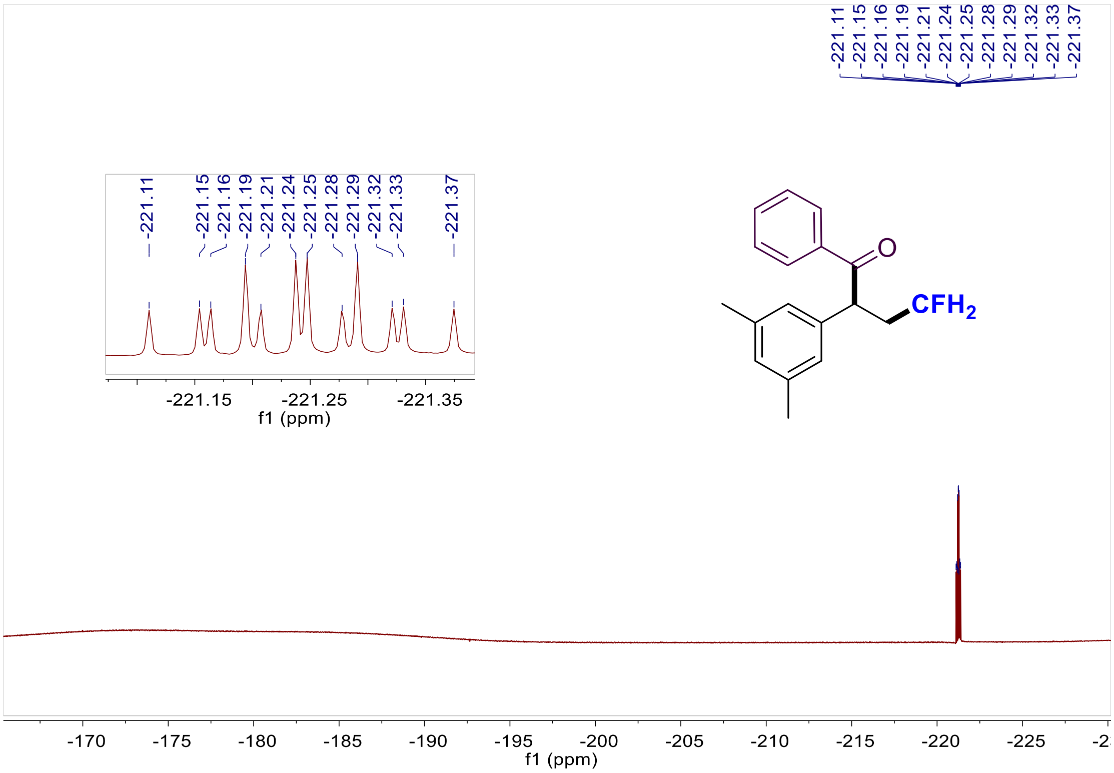

^{1}H NMR (500 MHz, $CDCl_3$) spectrum for **22**.

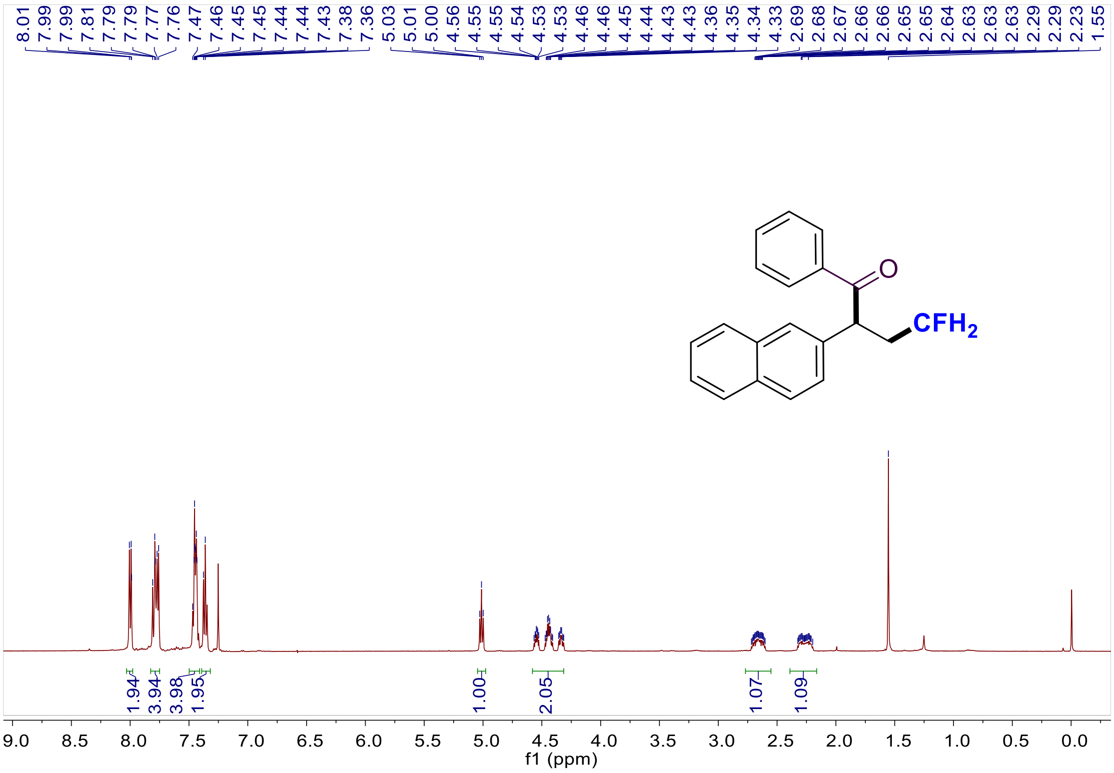

^{13}C NMR (151 MHz, $CDCl_3$) spectrum for **22**.

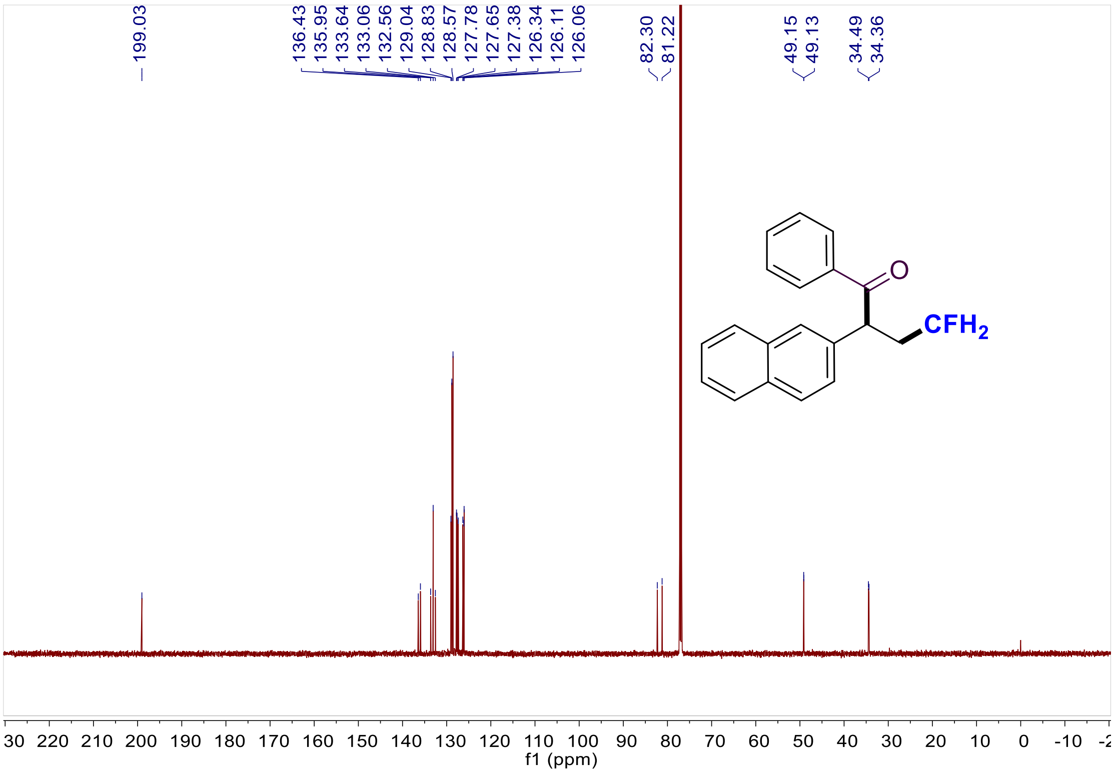

^{19}F NMR (565 MHz, $CDCl_3$) spectrum for **22**.

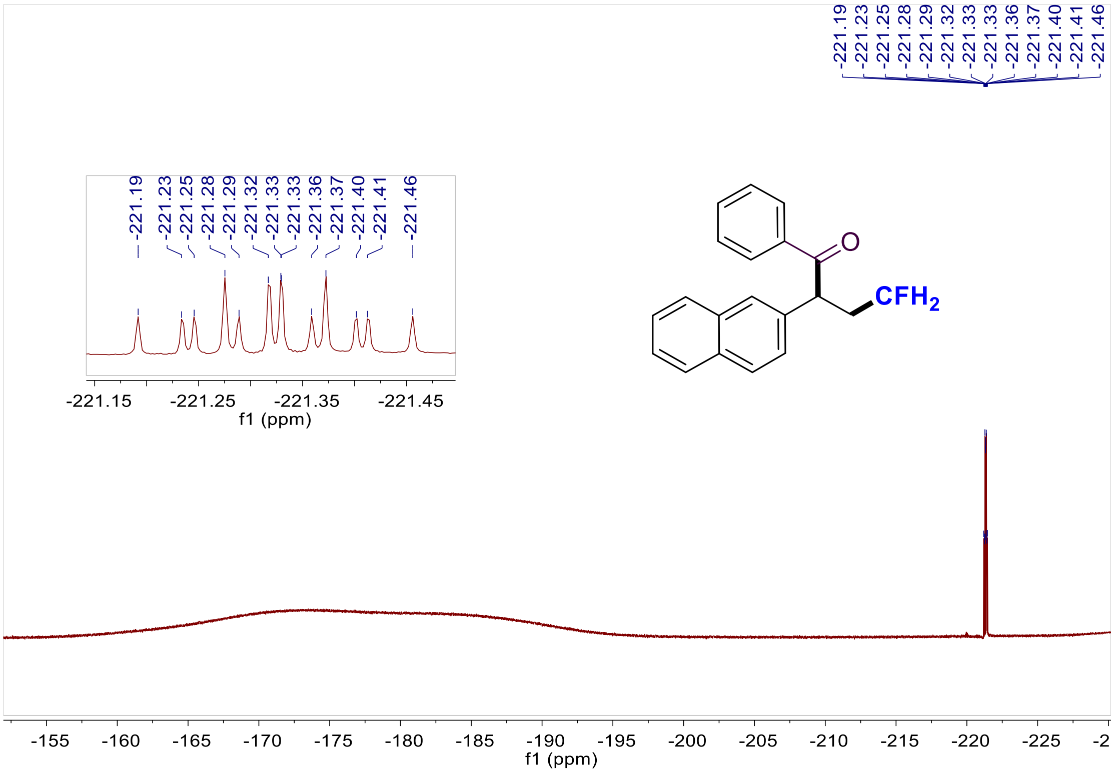

^{1}H NMR (500 MHz, $CDCl_3$) spectrum for **23**.

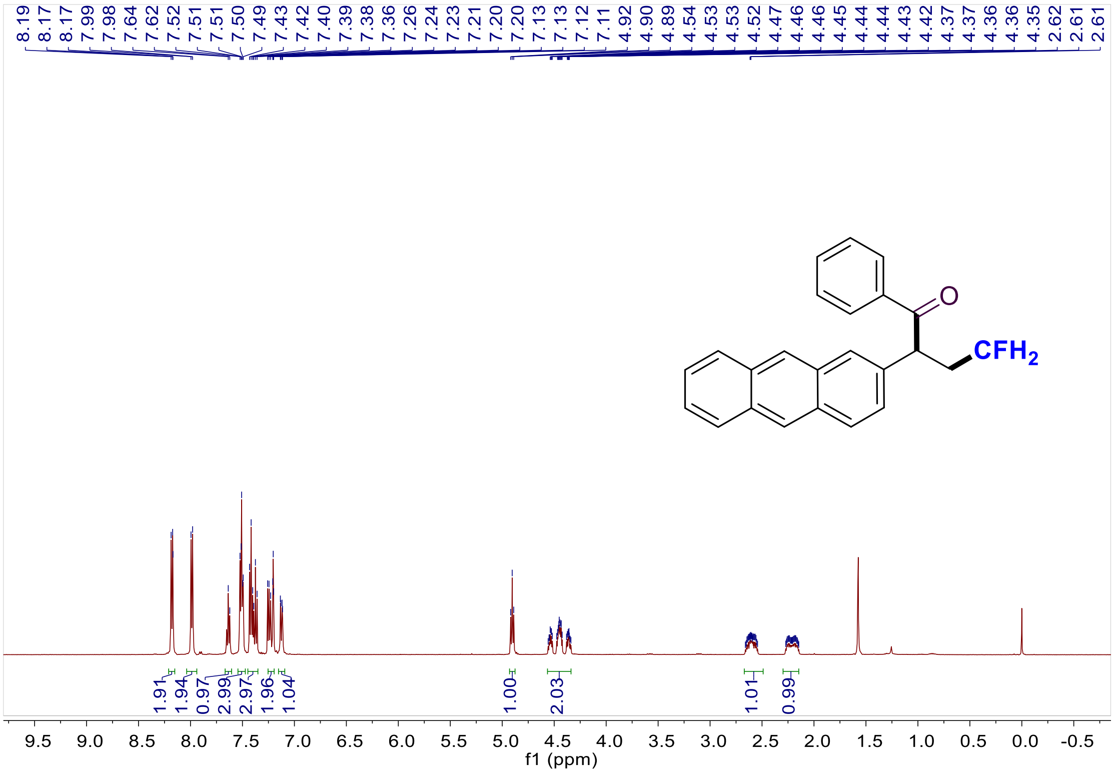

^{13}C NMR (151 MHz, $CDCl_3$) spectrum for **23**.

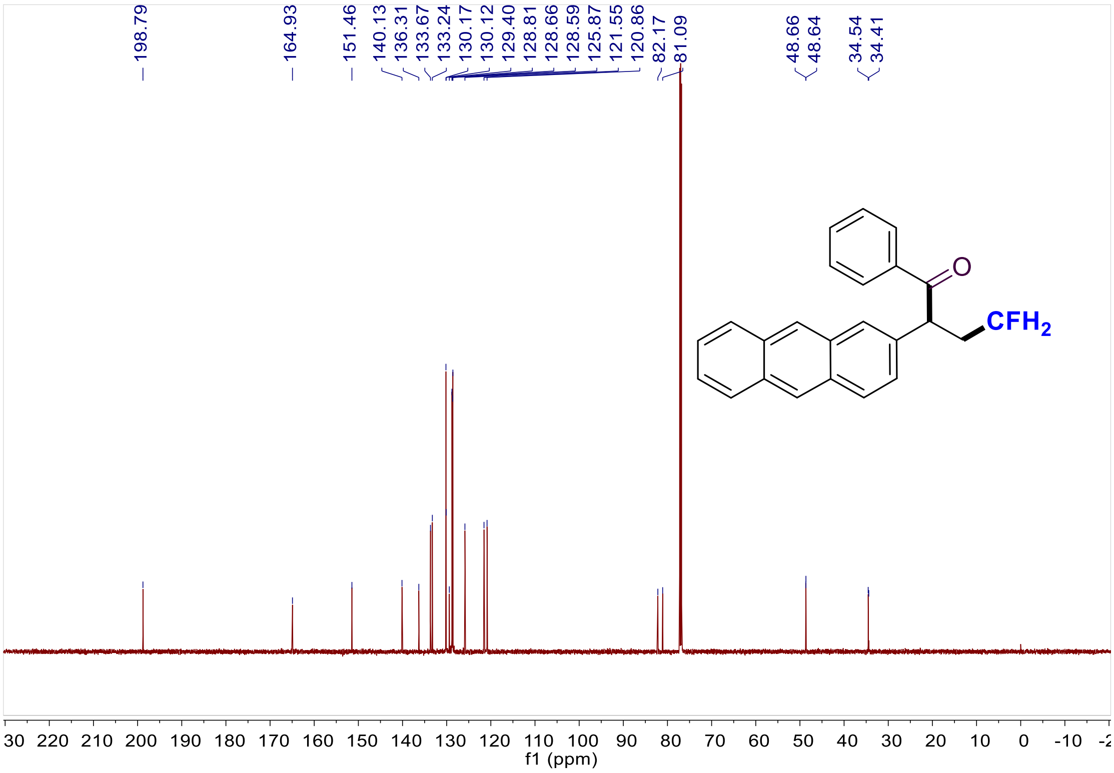

^{19}F NMR (565 MHz, $CDCl_3$) spectrum for **23**.

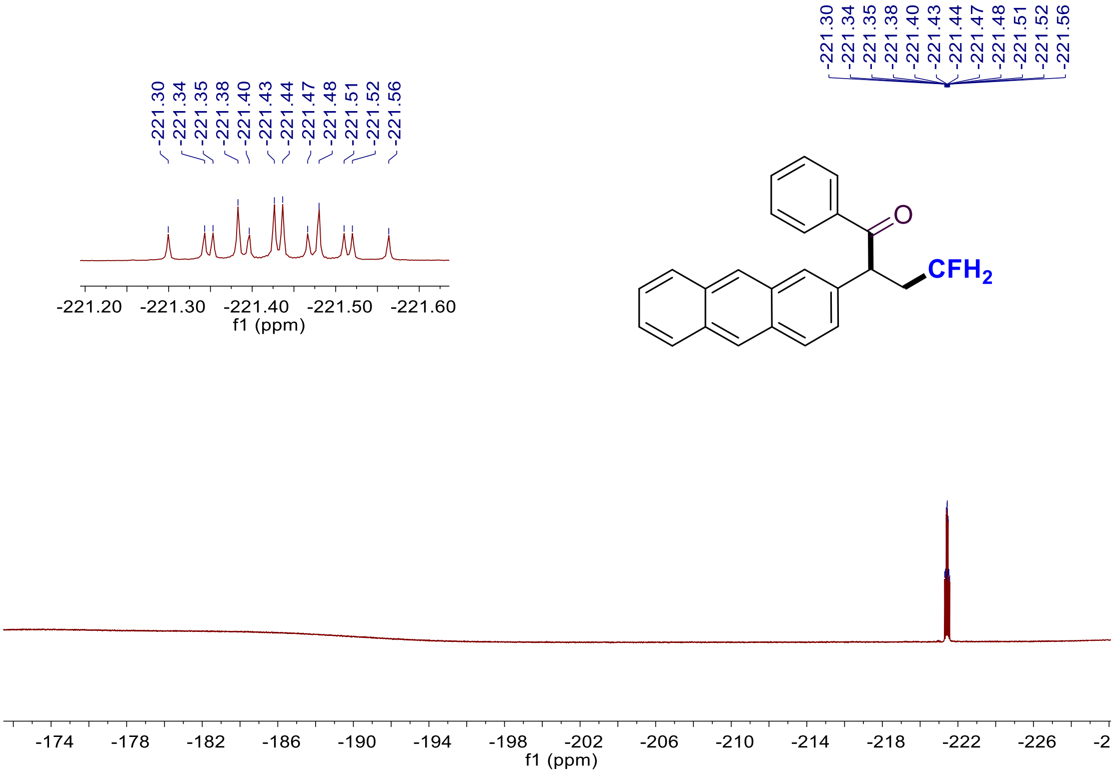

^{1}H NMR (500 MHz, $CDCl_3$) spectrum for **24**.

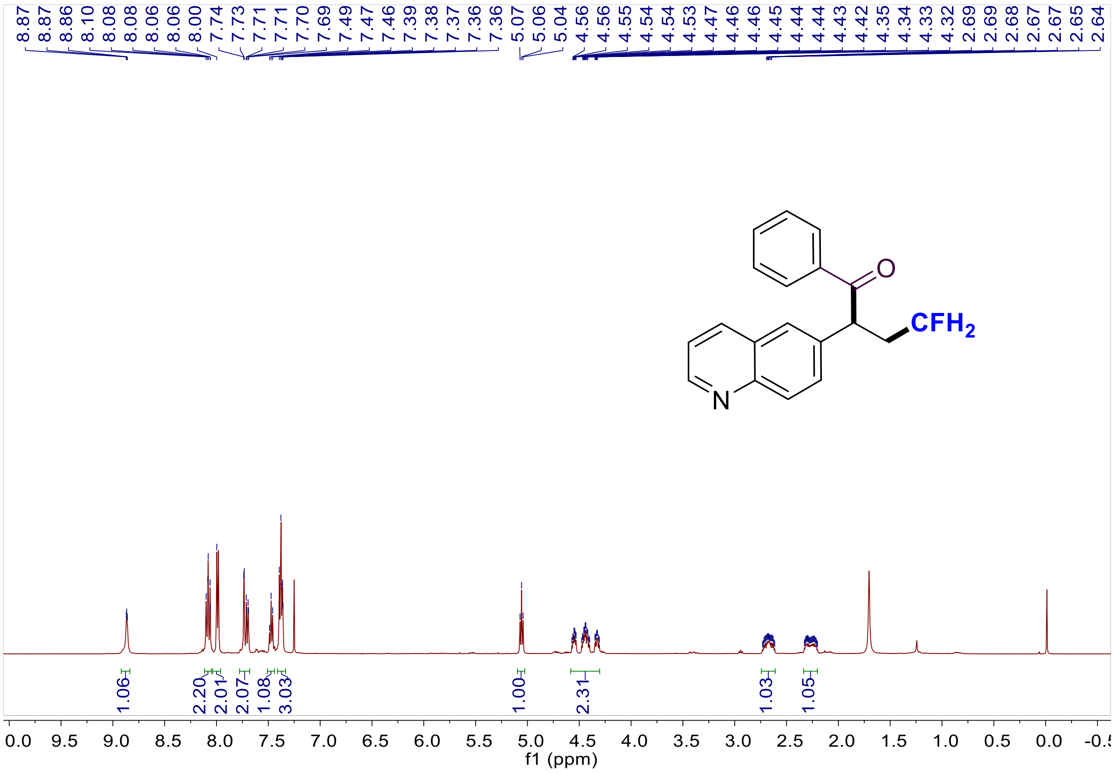

^{13}C NMR (151 MHz, $CDCl_3$) spectrum for **24**.

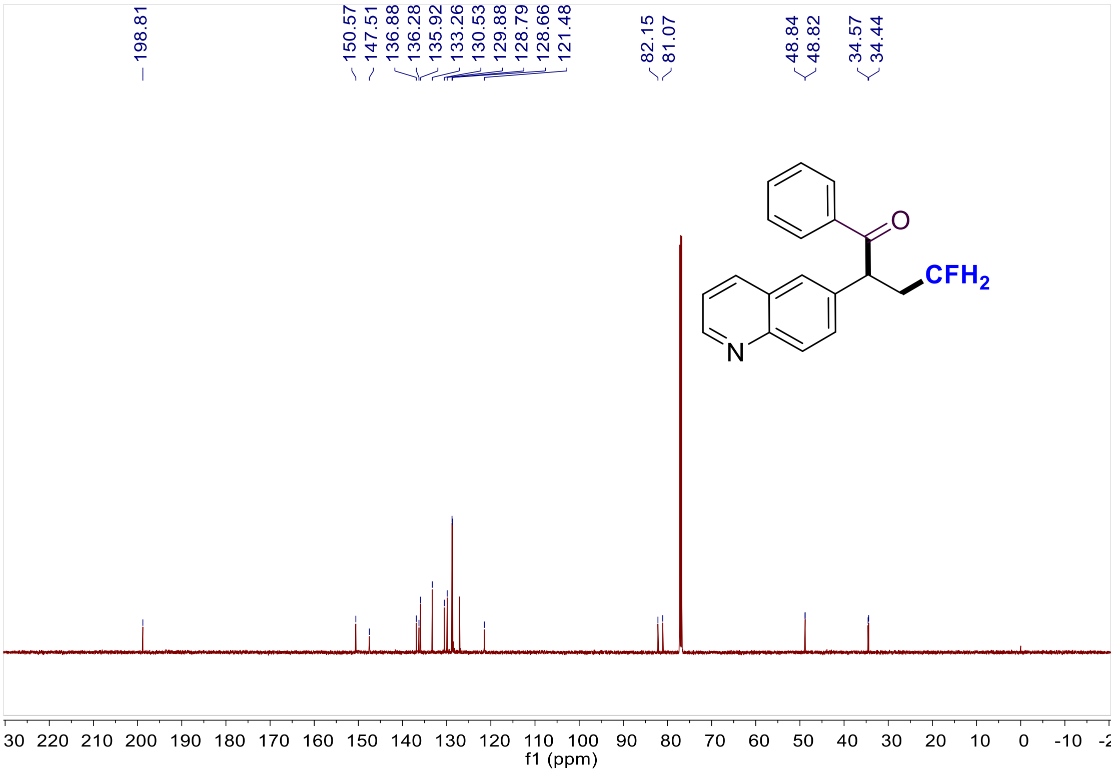

^{19}F NMR (565 MHz, $CDCl_3$) spectrum for **24**.

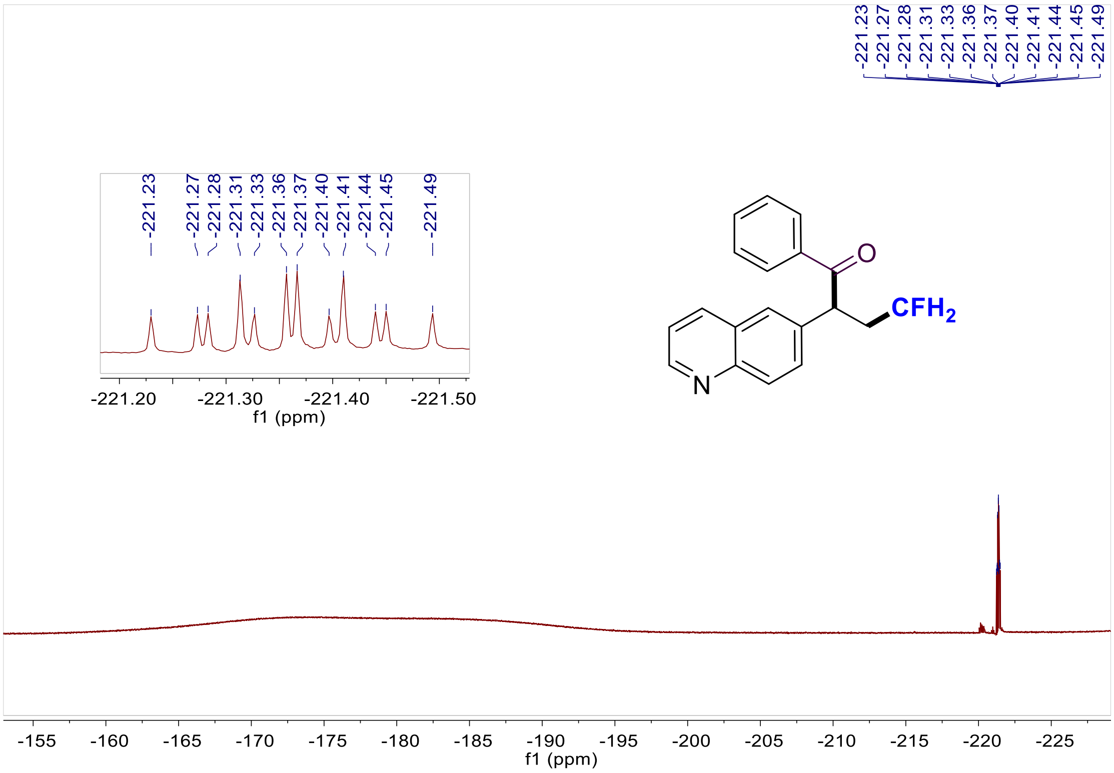

^{1}H NMR (500 MHz, $CDCl_3$) spectrum for **25**.

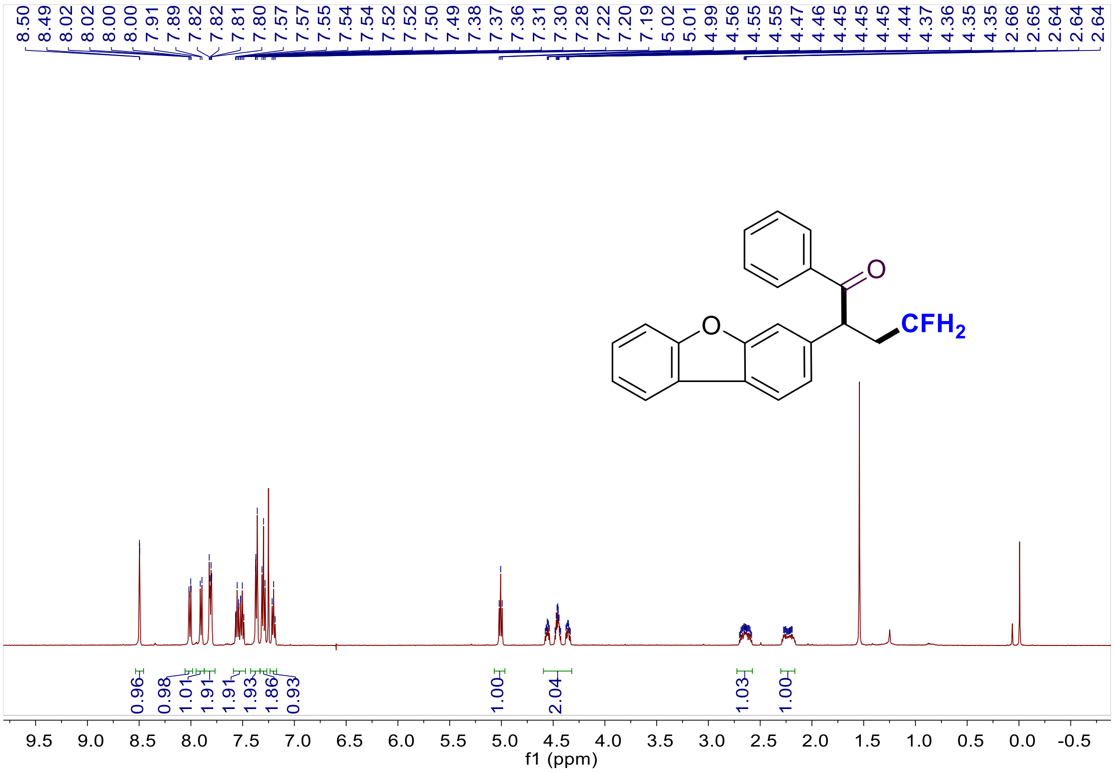

^{13}C NMR (151 MHz, CDCl$_3$) spectrum for **25**.

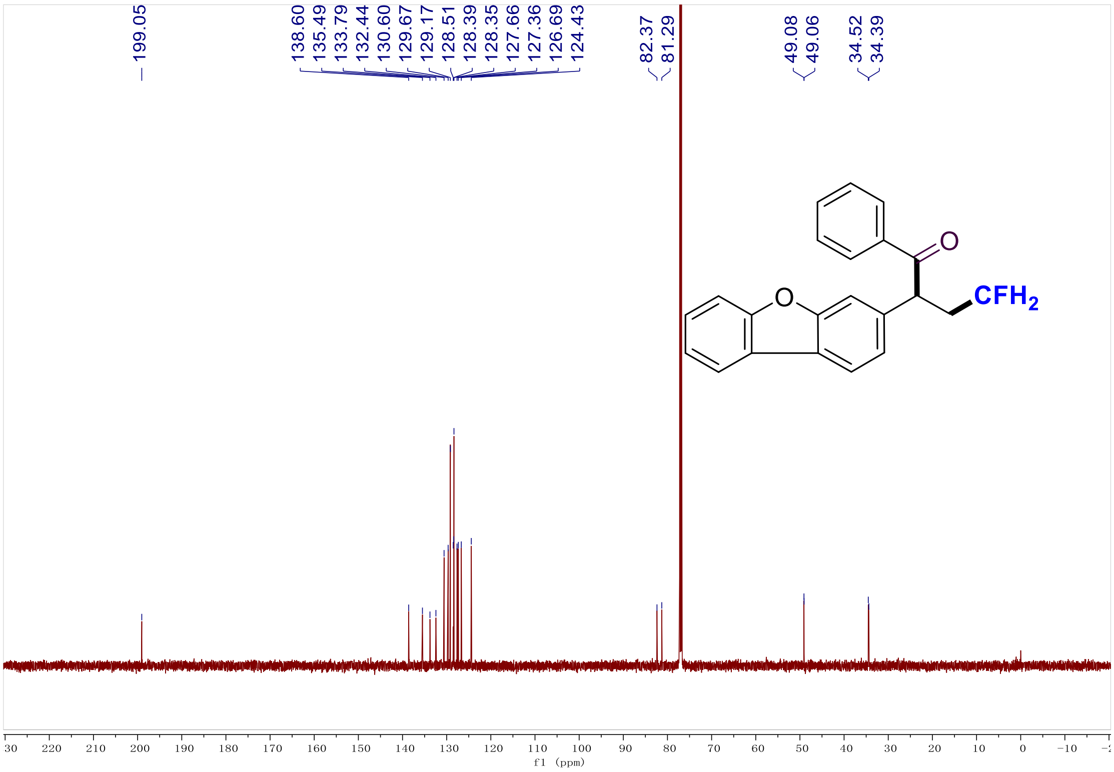

^{19}F NMR (565 MHz, CDCl$_3$) spectrum for **25**.

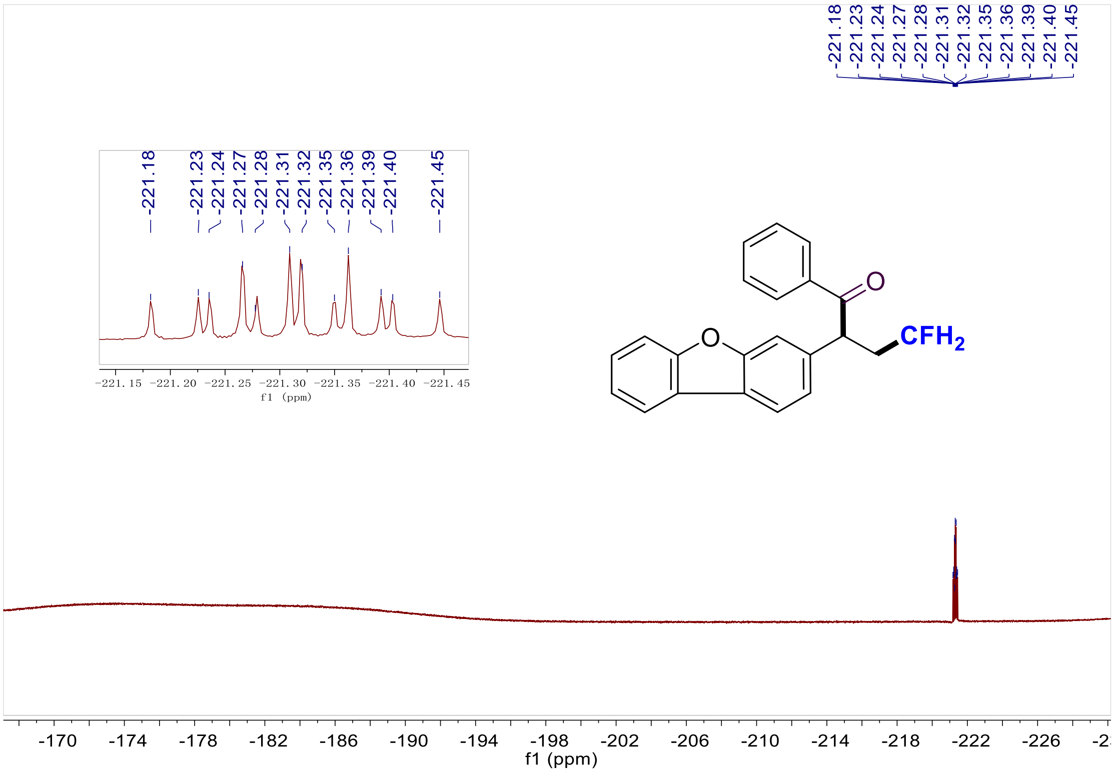

^{1}H NMR (500 MHz, $CDCl_3$) spectrum for **26**.

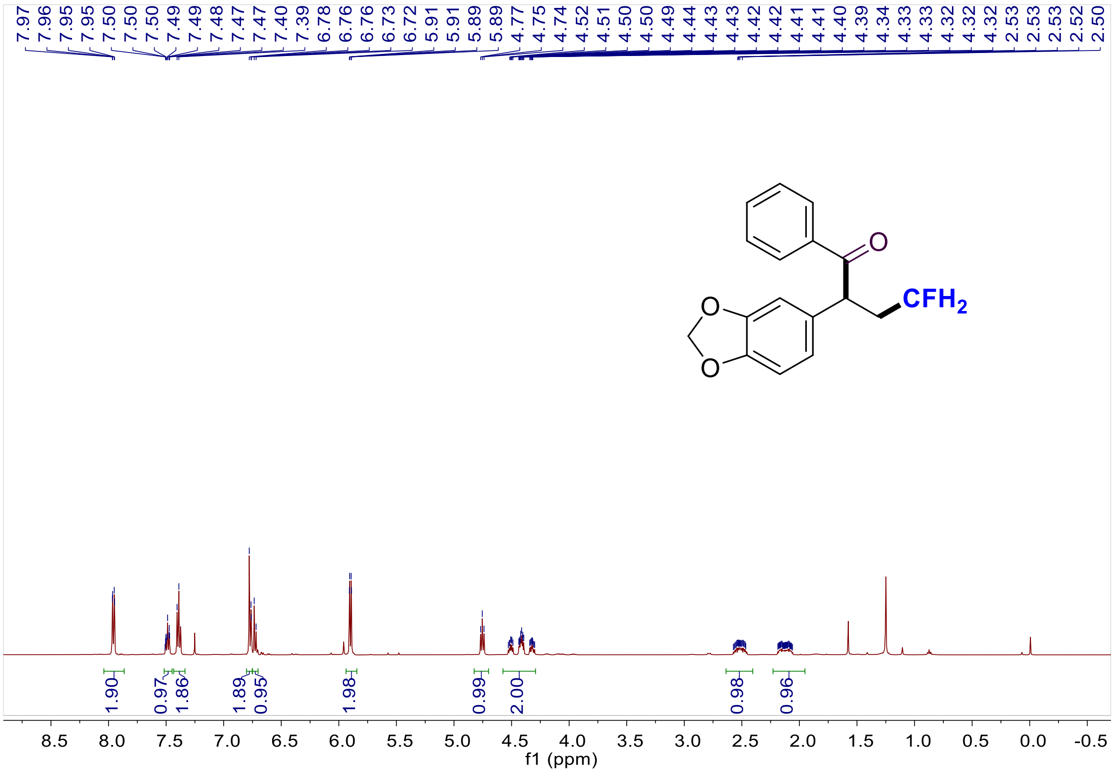

^{13}C NMR (151 MHz, $CDCl_3$) spectrum for **26**.

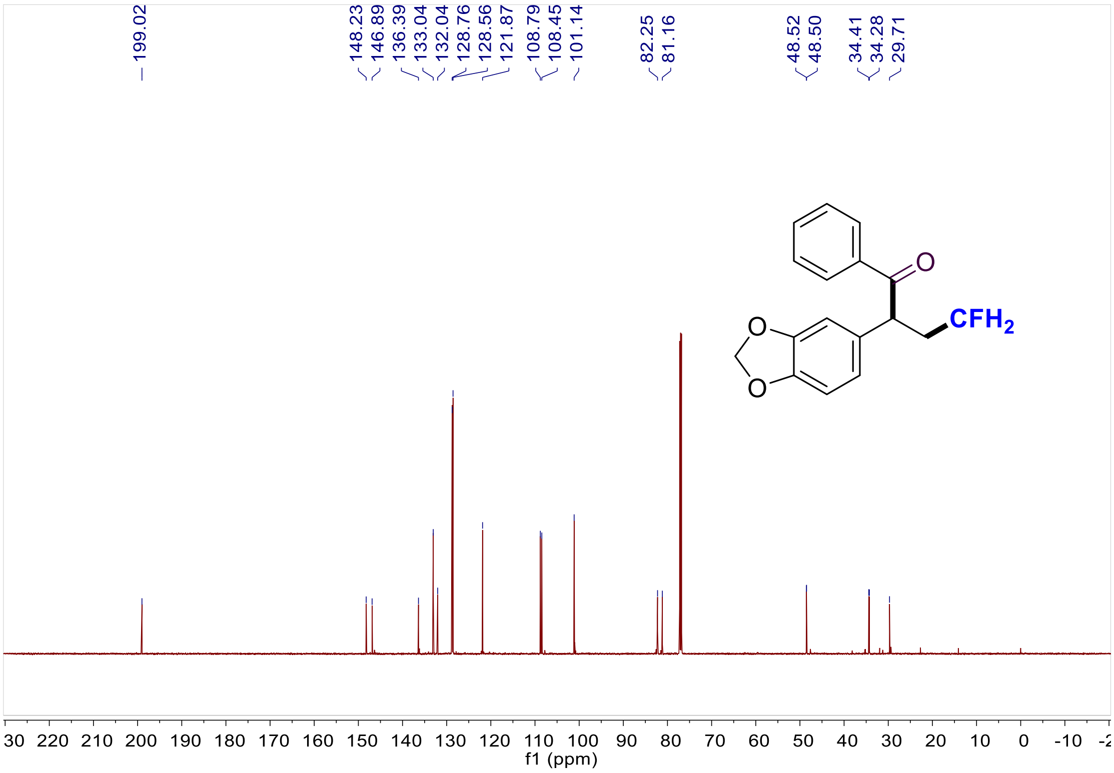

^{19}F NMR (565 MHz, $CDCl_3$) spectrum for **26**.

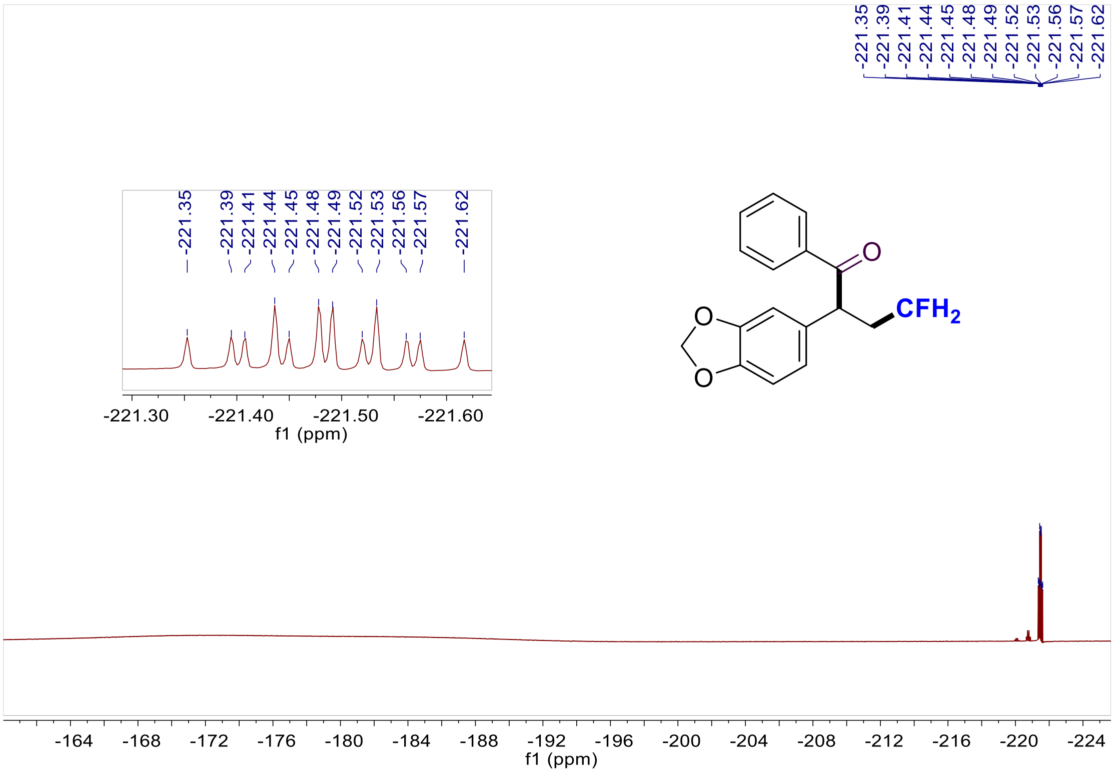

^{1}H NMR (500 MHz, $CDCl_3$) spectrum for **27**.

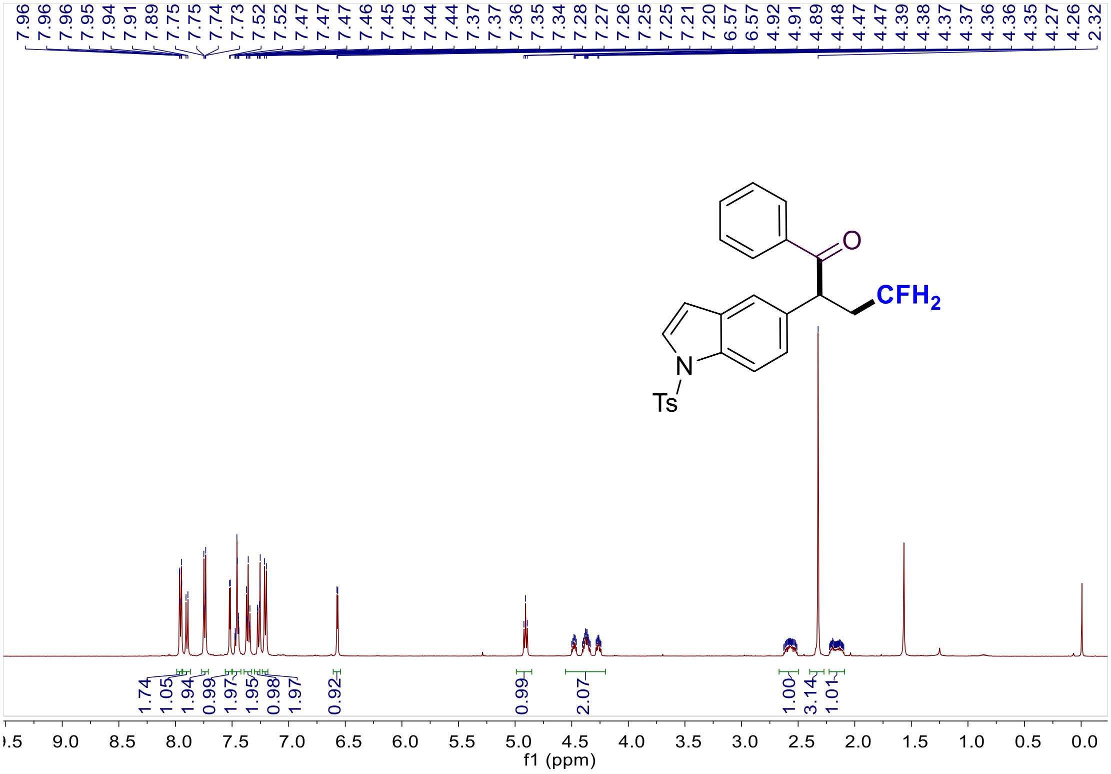

^{13}C NMR (151 MHz, $CDCl_3$) spectrum for **27**.

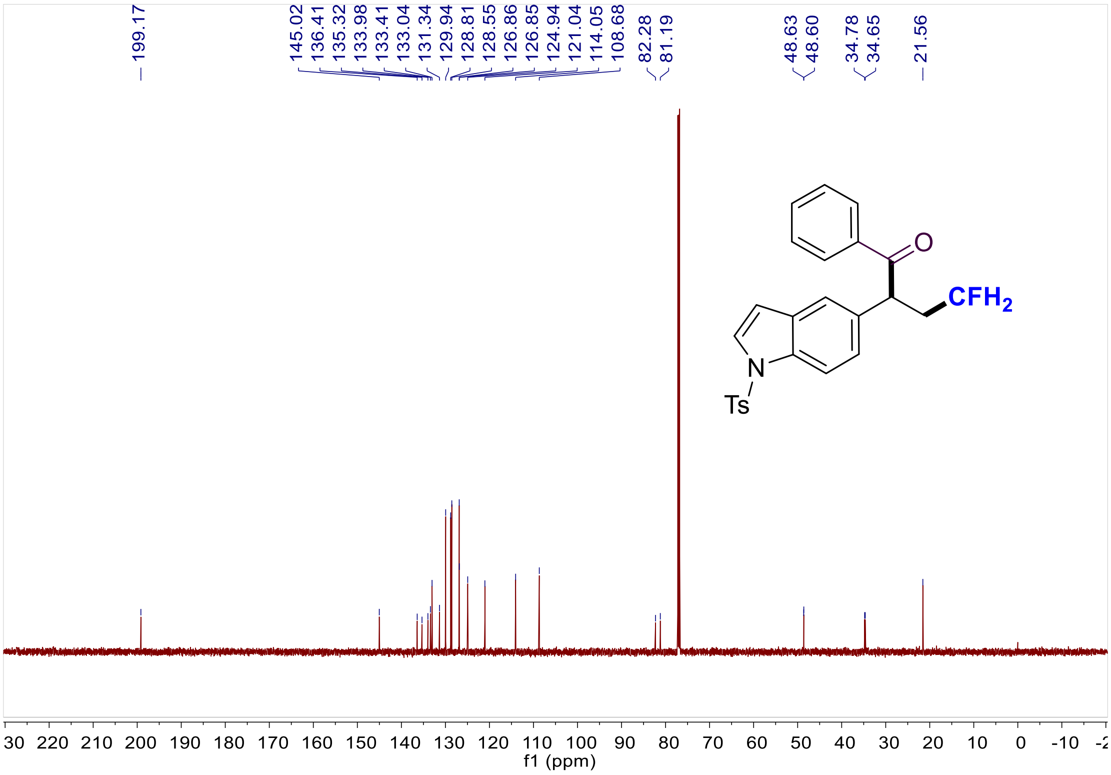

^{19}F NMR (565 MHz, $CDCl_3$) spectrum for **27**.

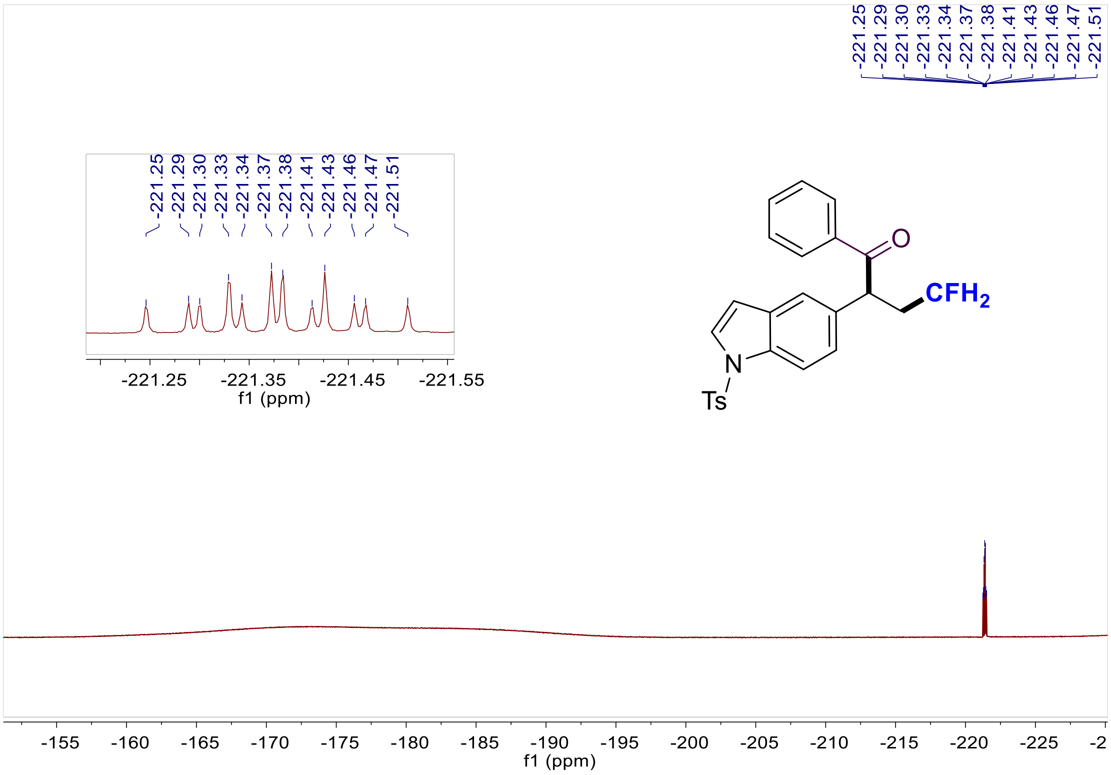

^{1}H NMR (500 MHz, $CDCl_3$) spectrum for **28**.

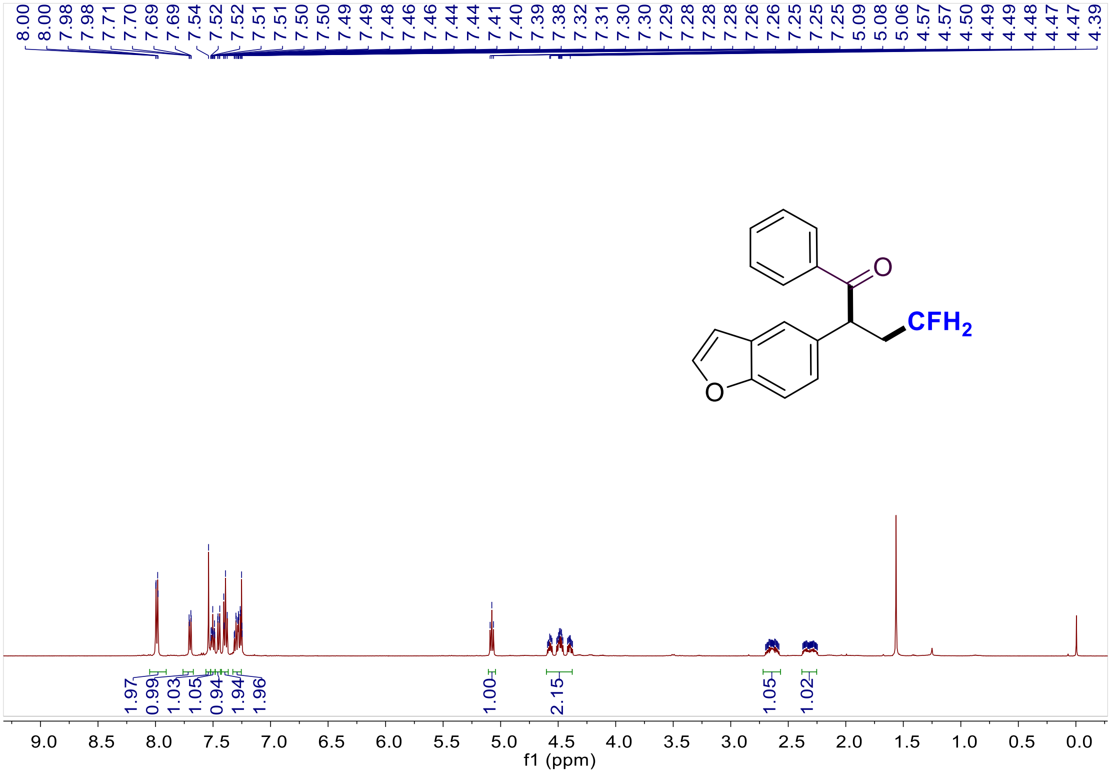

^{13}C NMR (151 MHz, $CDCl_3$) spectrum for **28**.

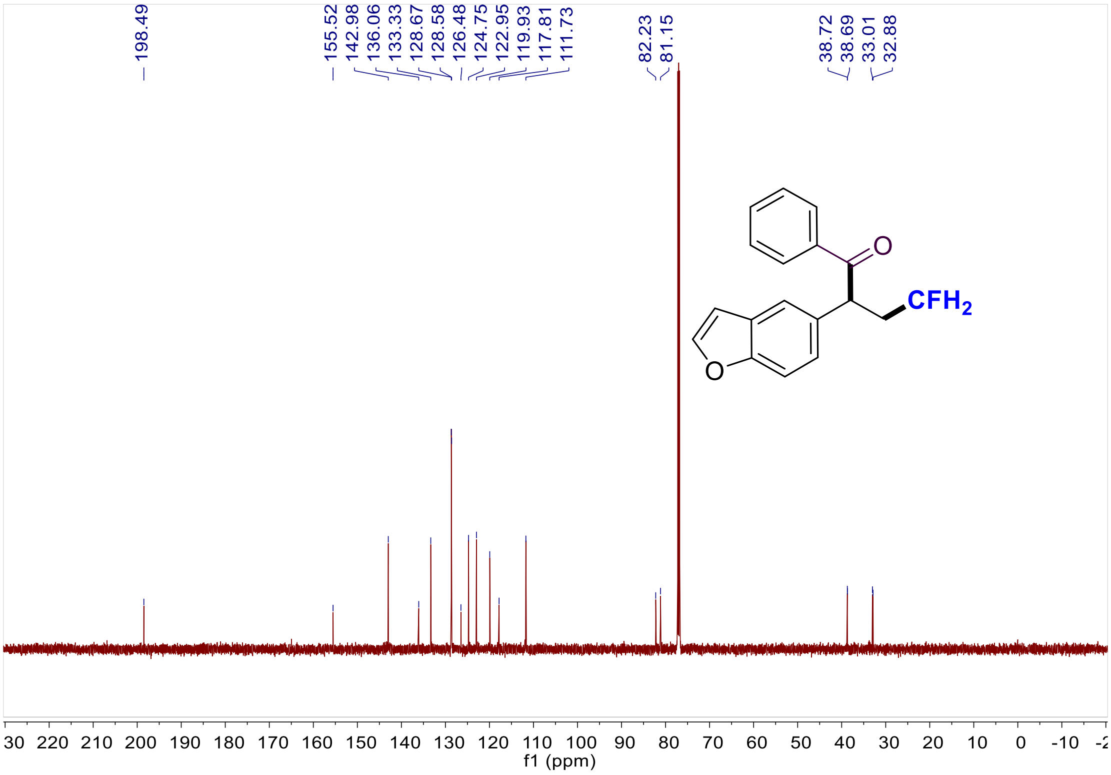

^{19}F NMR (565 MHz, $CDCl_3$) spectrum for **28**.

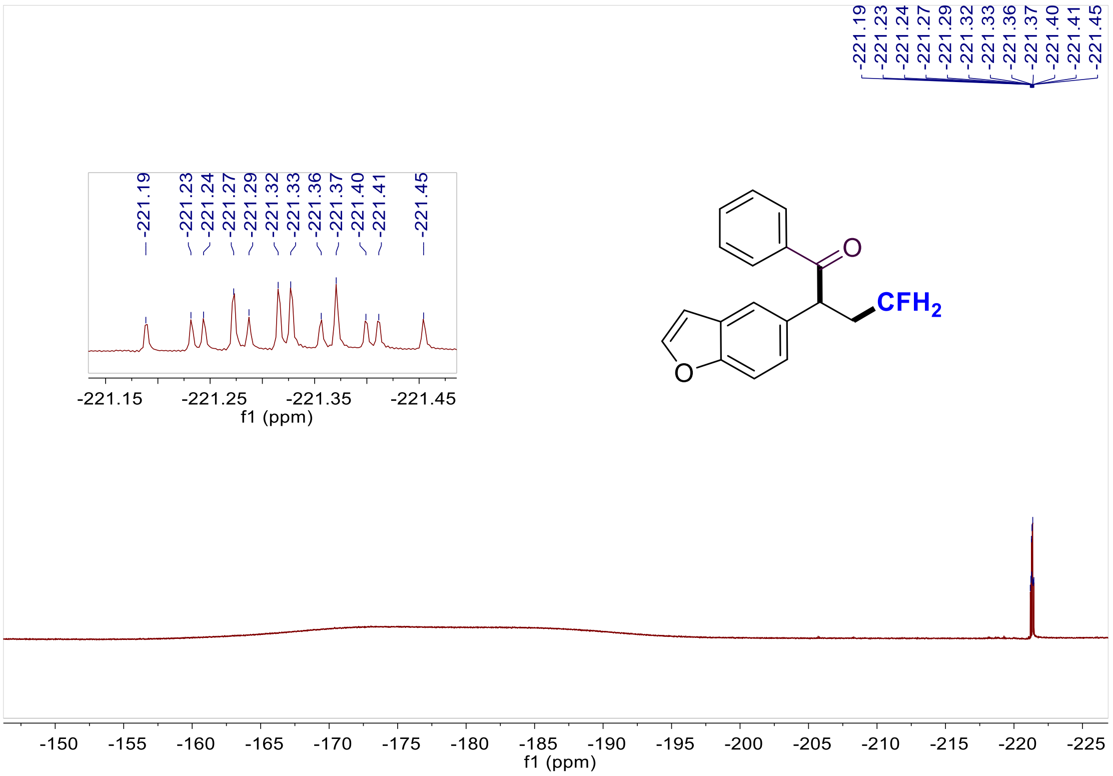

^{1}H NMR (500 MHz, $CDCl_3$) spectrum for **29**.

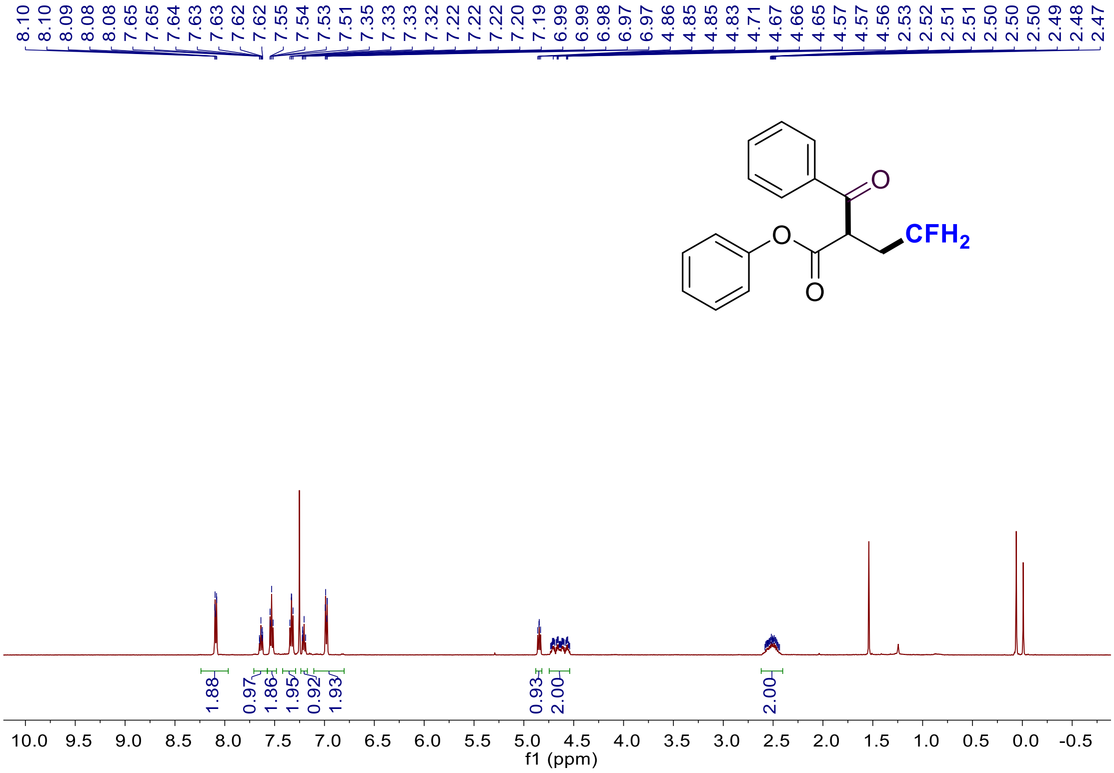

^{13}C NMR (151 MHz, $CDCl_3$) spectrum for **29**.

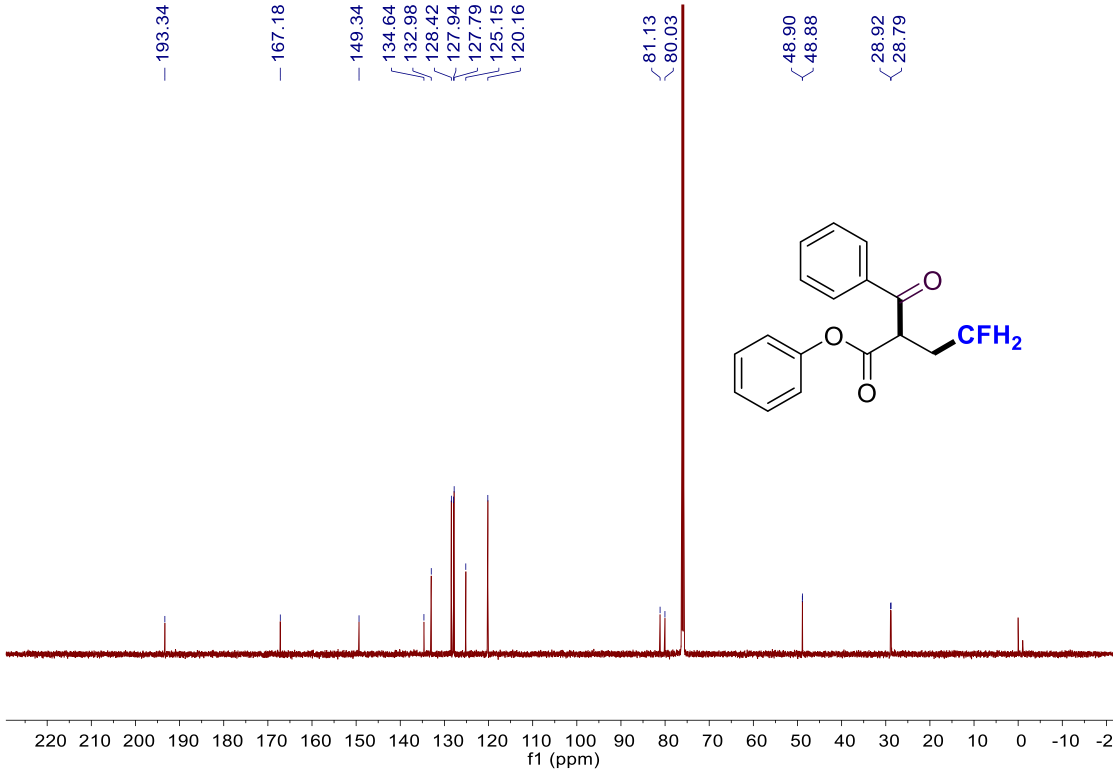

^{19}F NMR (565 MHz, $CDCl_3$) spectrum for **29**.

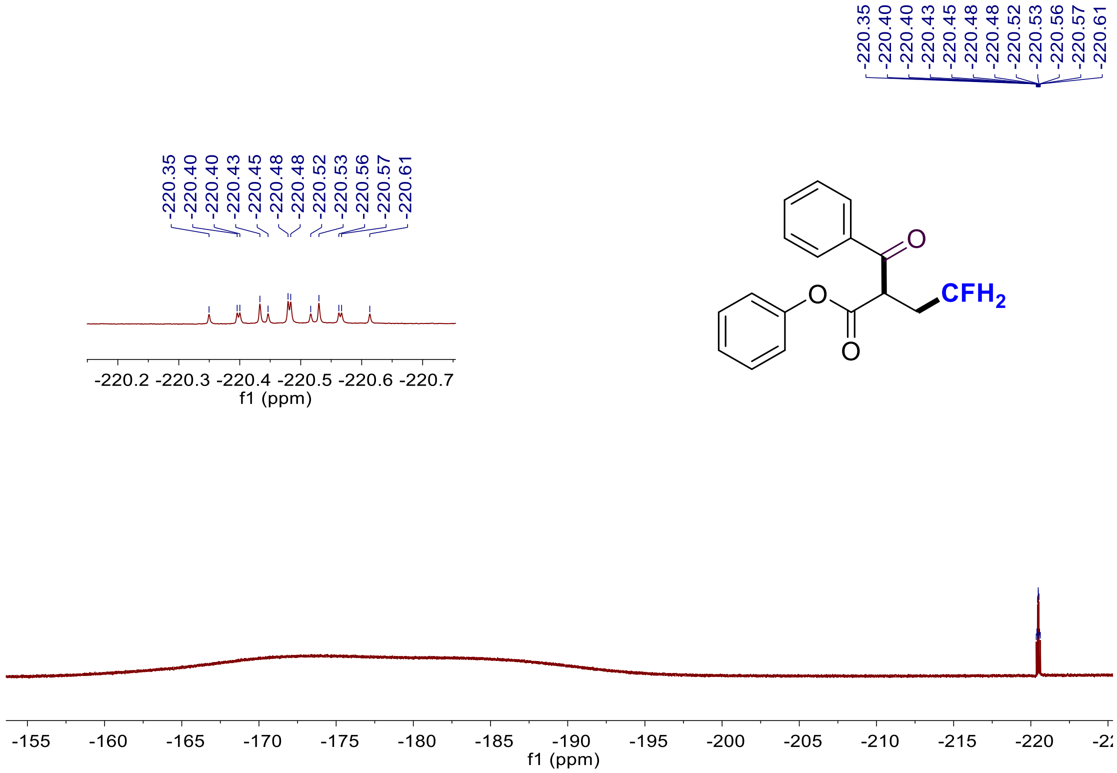

^{1}H NMR (500 MHz, $CDCl_3$) spectrum for **30**.

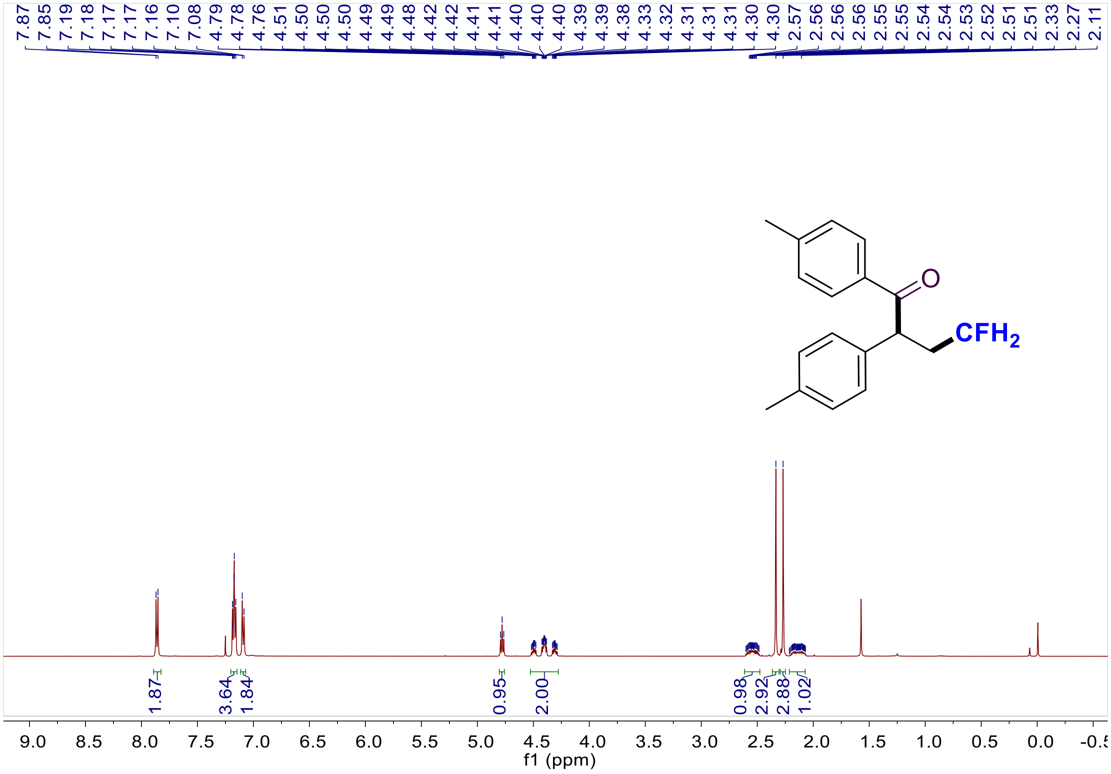

^{13}C NMR (151 MHz, $CDCl_3$) spectrum for **30**.

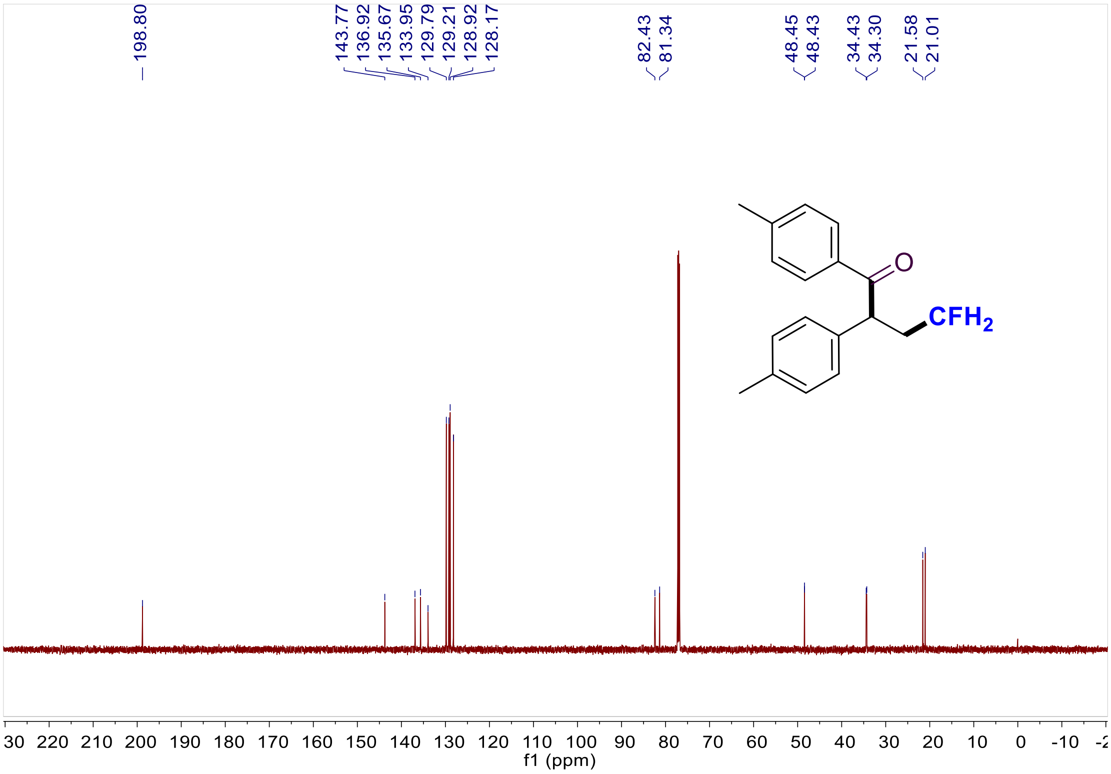

^{19}F NMR (565 MHz, $CDCl_3$) spectrum for **30**.

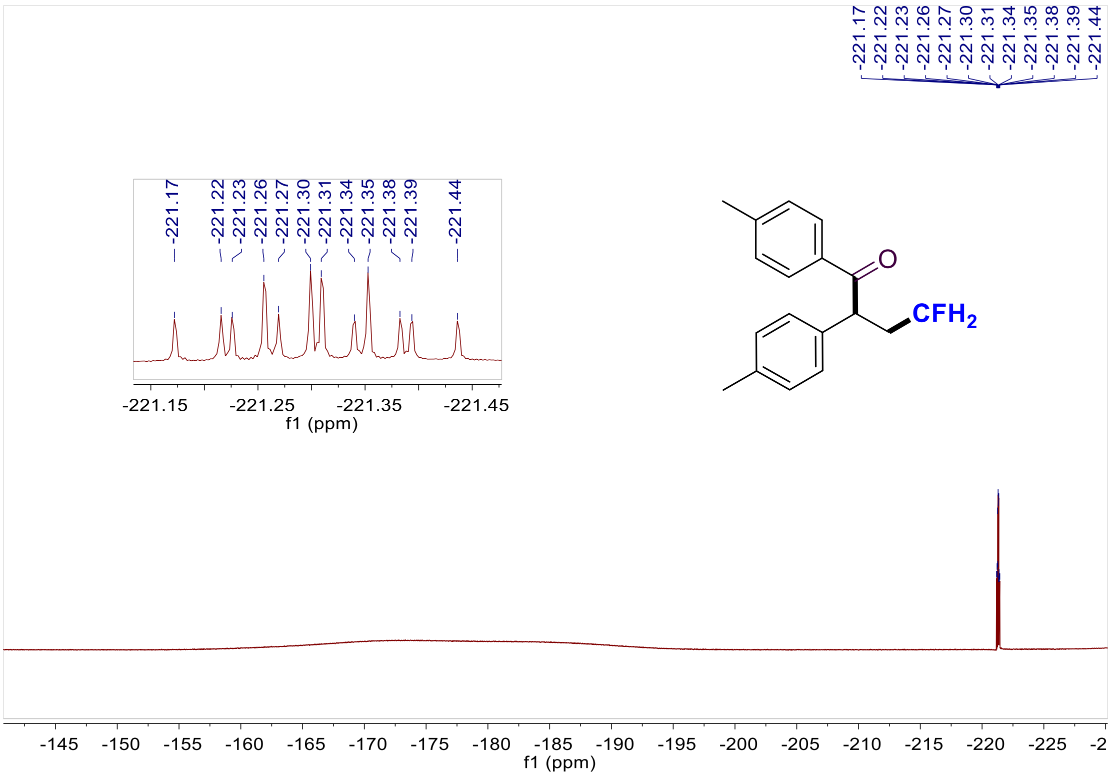

^{1}H NMR (500 MHz, $CDCl_3$) spectrum for **31**.

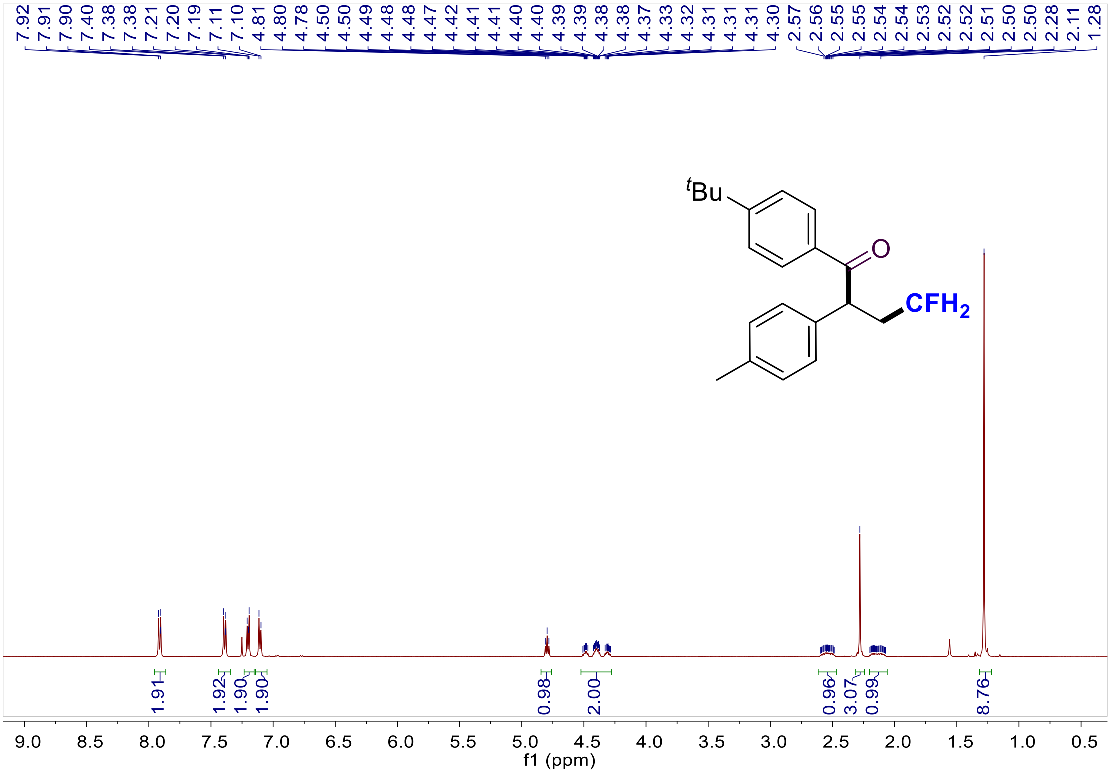

^{13}C NMR (151 MHz, $CDCl_3$) spectrum for **31**.

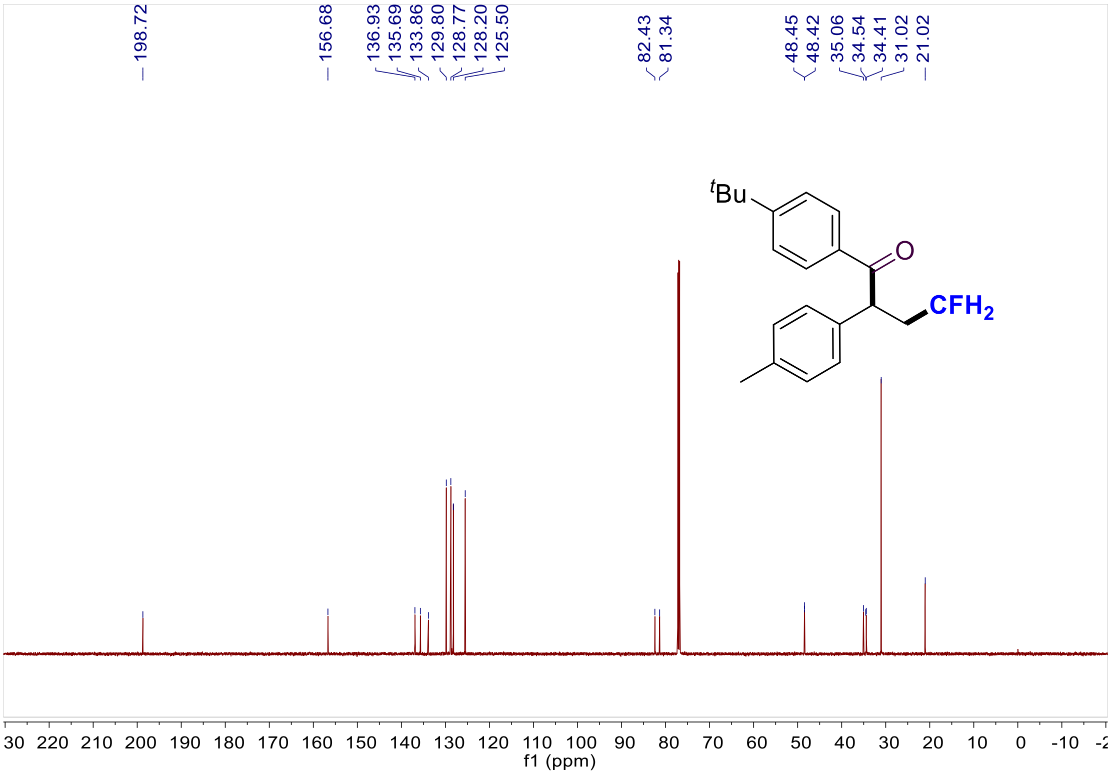

^{19}F NMR (565 MHz, $CDCl_3$) spectrum for **31**.

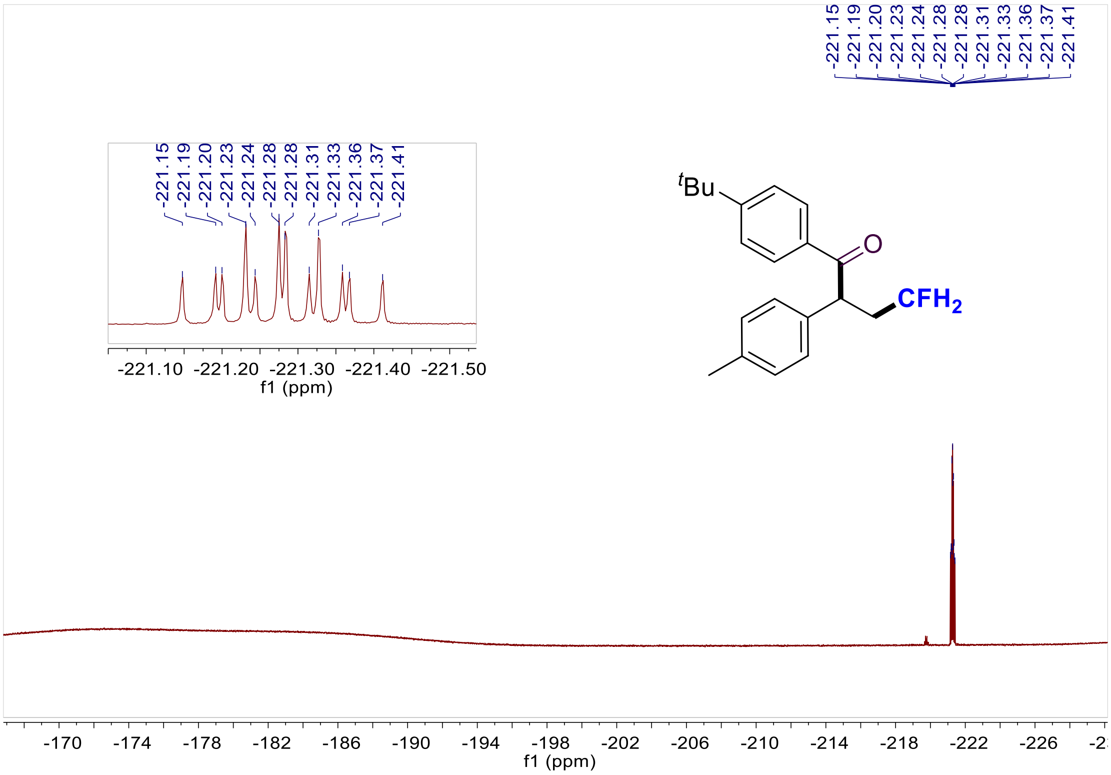

^{1}H NMR (500 MHz, $CDCl_3$) spectrum for **32**.

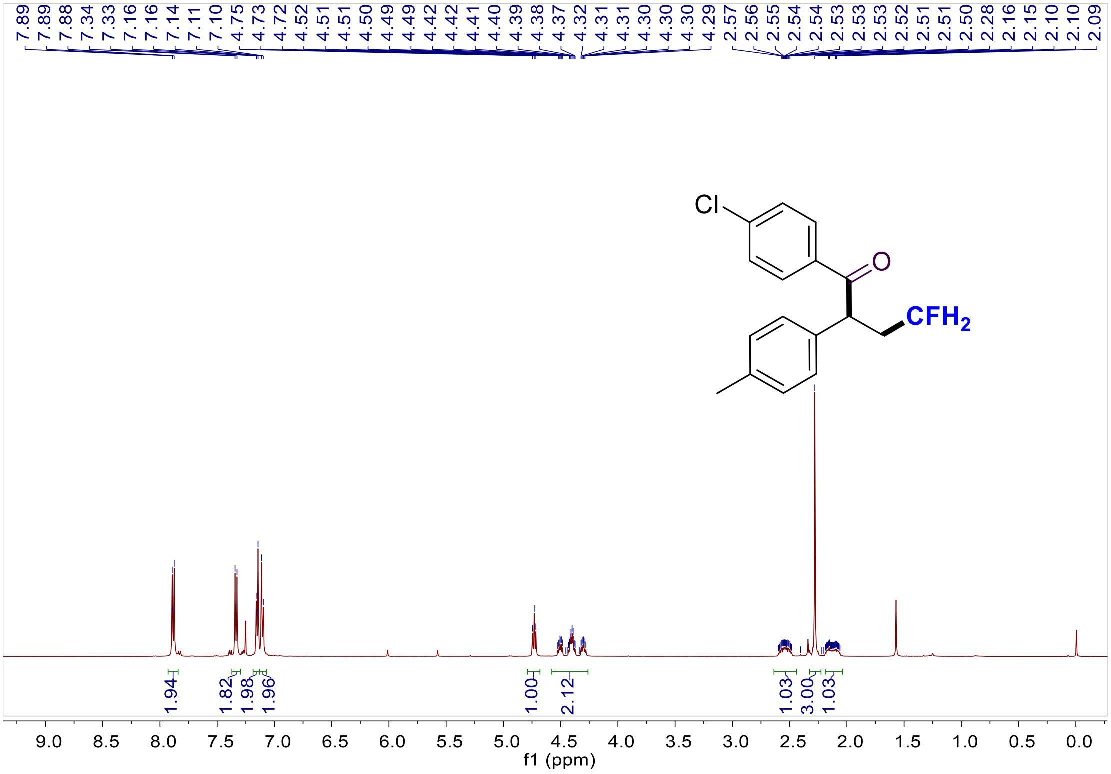

^{13}C NMR (151 MHz, $CDCl_3$) spectrum for **32**.

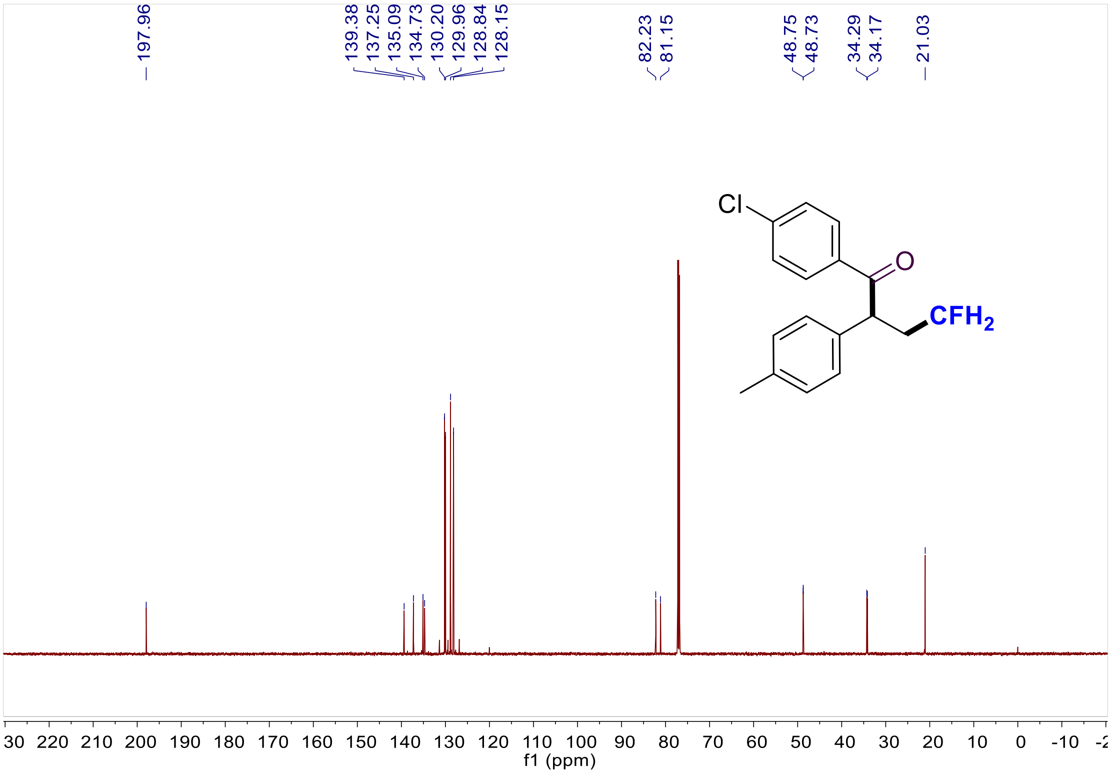

^{19}F NMR (565 MHz, $CDCl_3$) spectrum for **32**.

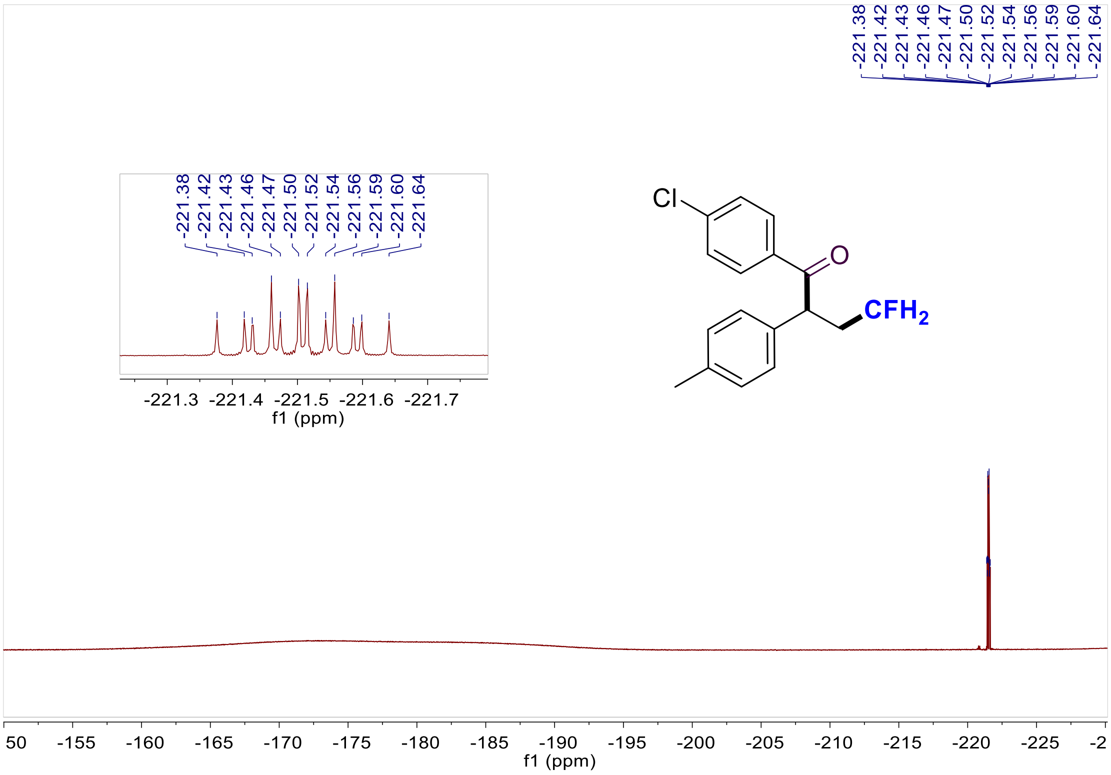

^{1}H NMR (500 MHz, $CDCl_3$) spectrum for **33**.

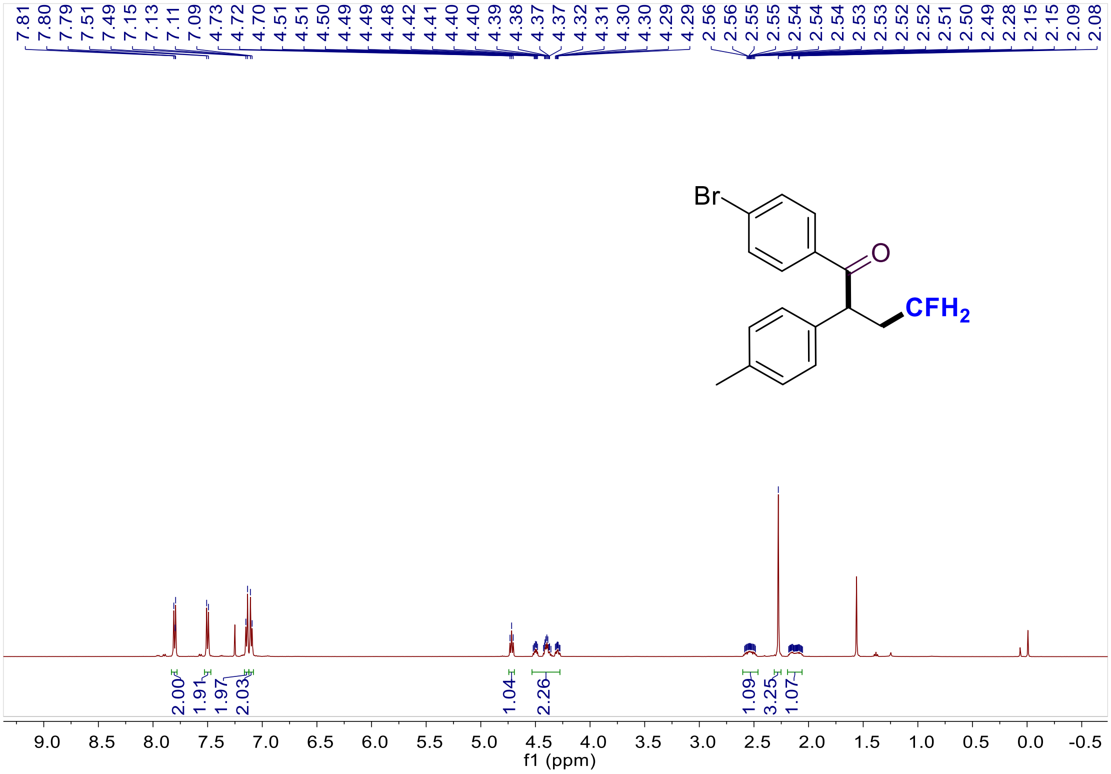

^{13}C NMR (151 MHz, $CDCl_3$) spectrum for **33**.

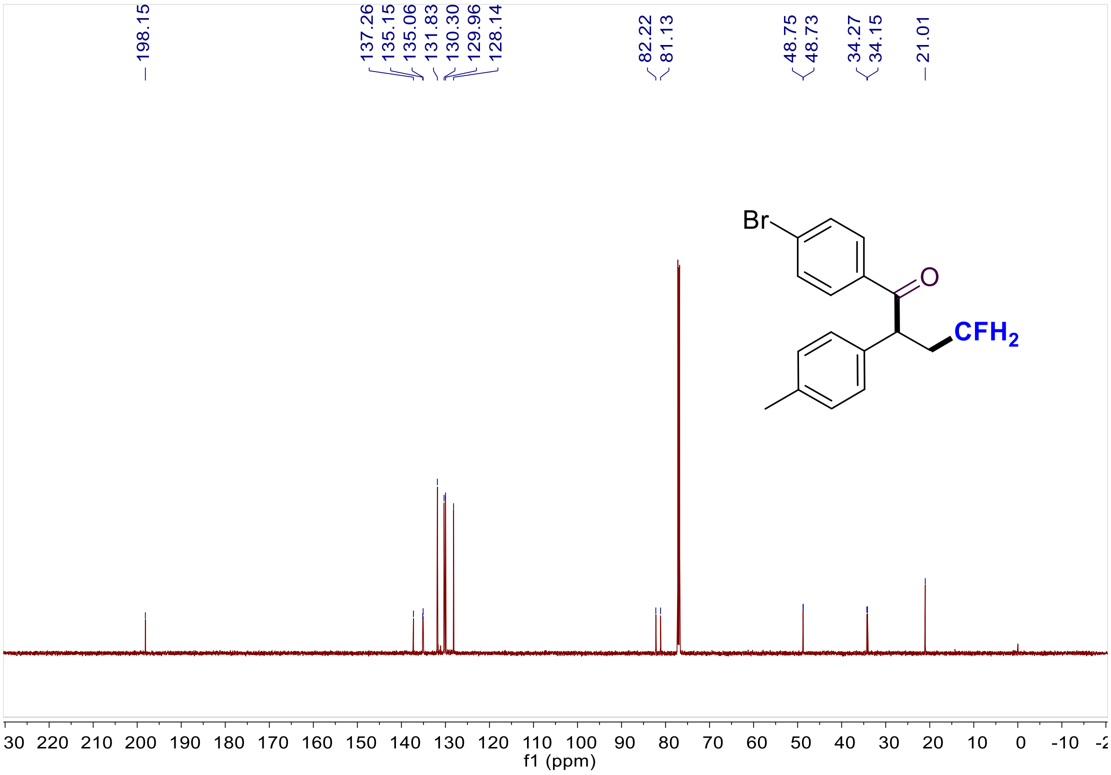

^{19}F NMR (565 MHz, $CDCl_3$) spectrum for **33**.

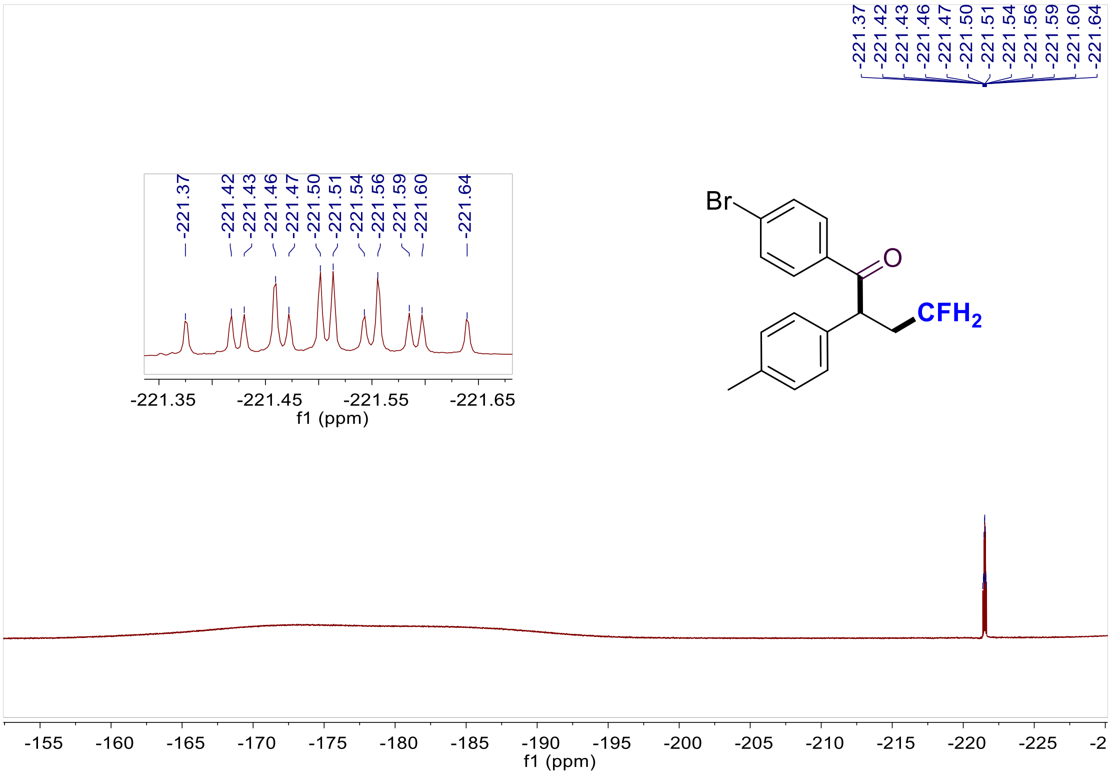

^{1}H NMR (500 MHz, $CDCl_3$) spectrum for **34**.

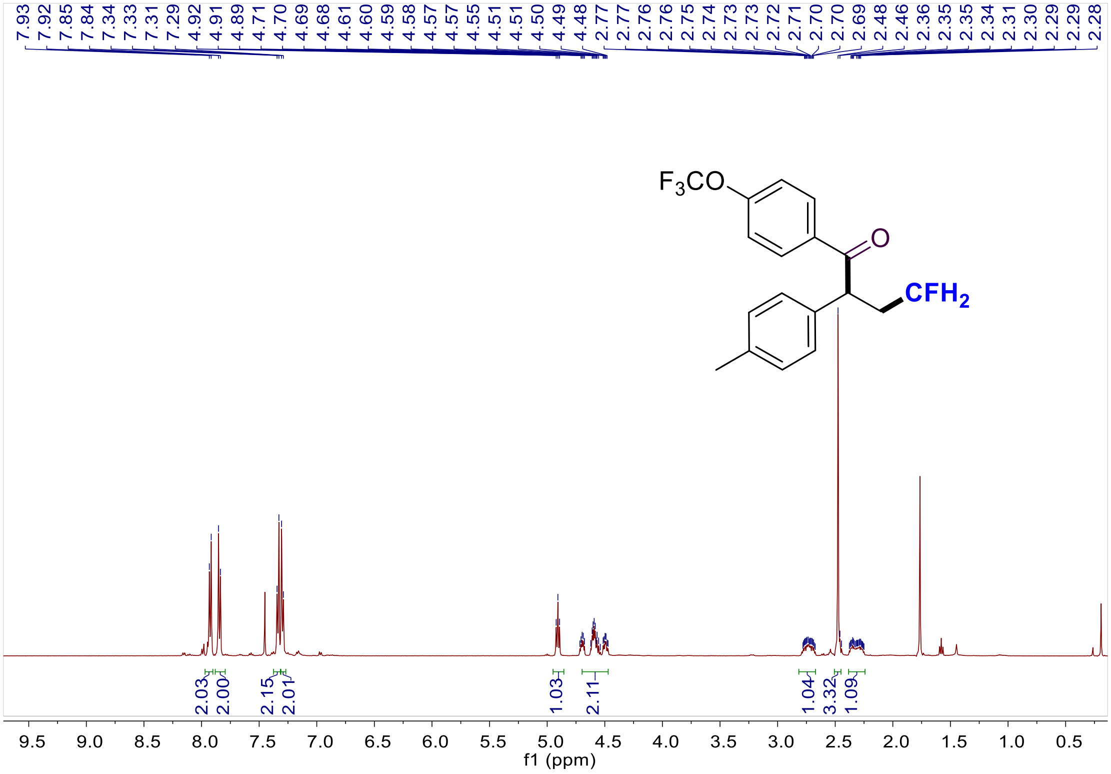

^{13}C NMR (151 MHz, $CDCl_3$) spectrum for **34**.

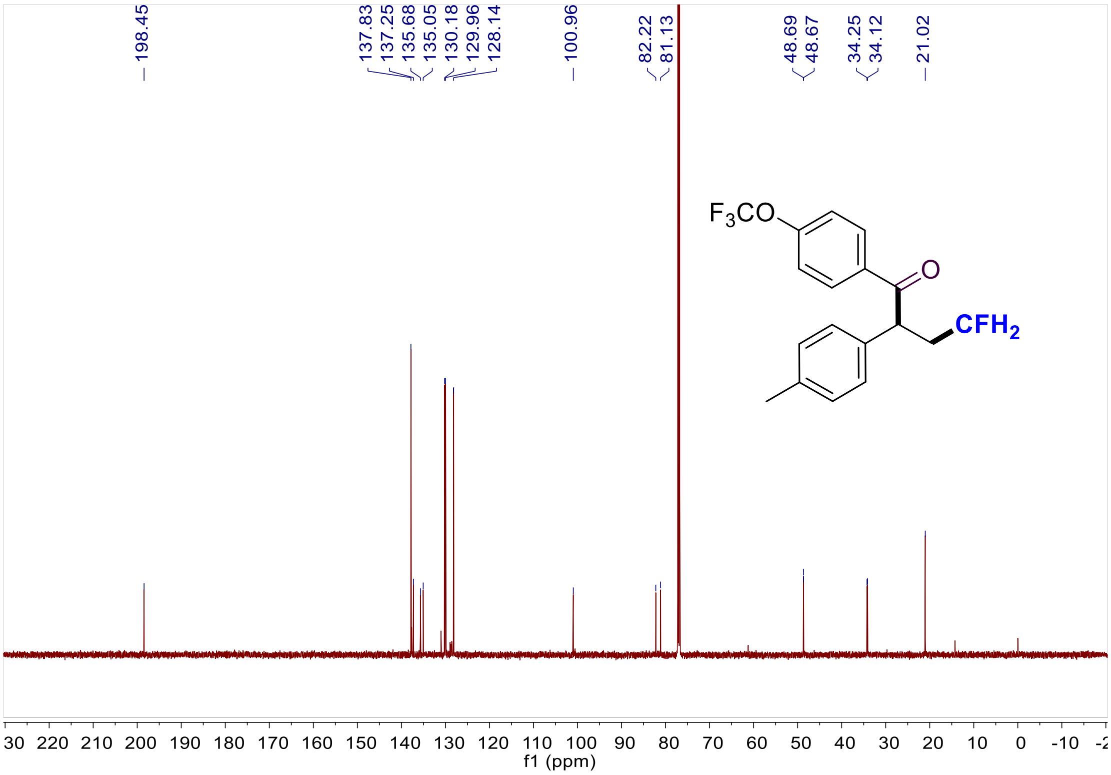

^{19}F NMR (565 MHz, $CDCl_3$) spectrum for **34**.

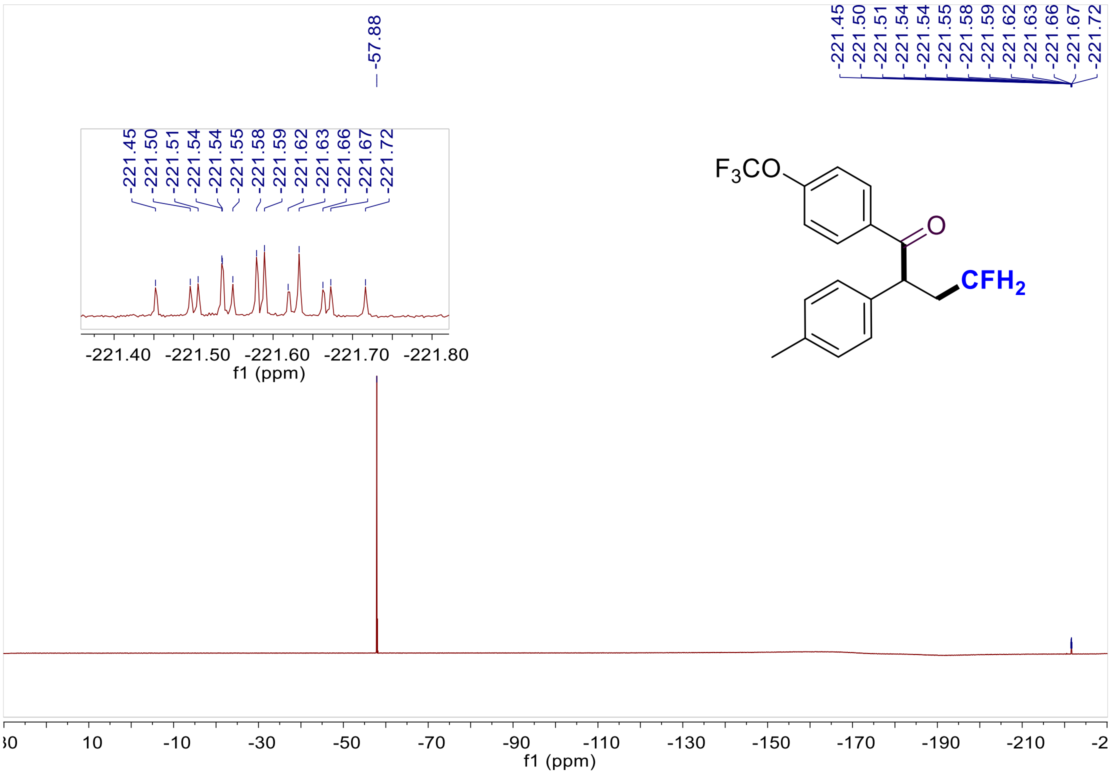

^{1}H NMR (500 MHz, $CDCl_3$) spectrum for **35**.

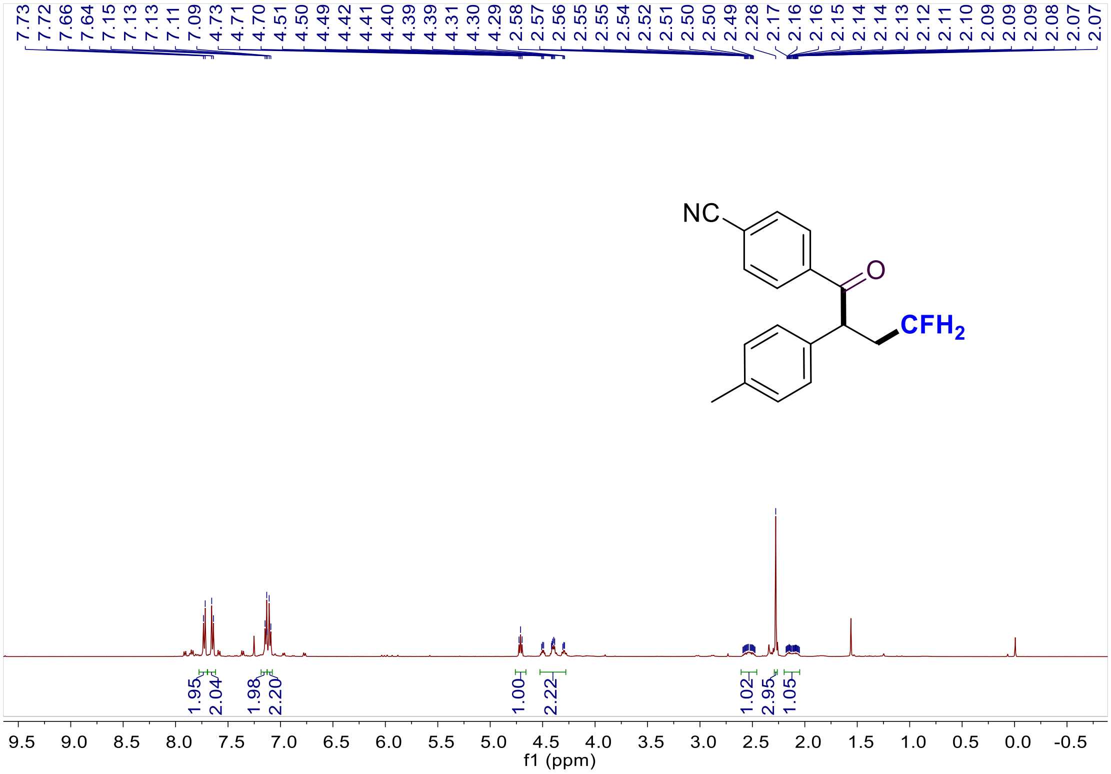

^{13}C NMR (151 MHz, CDCl$_3$) spectrum for **35**.

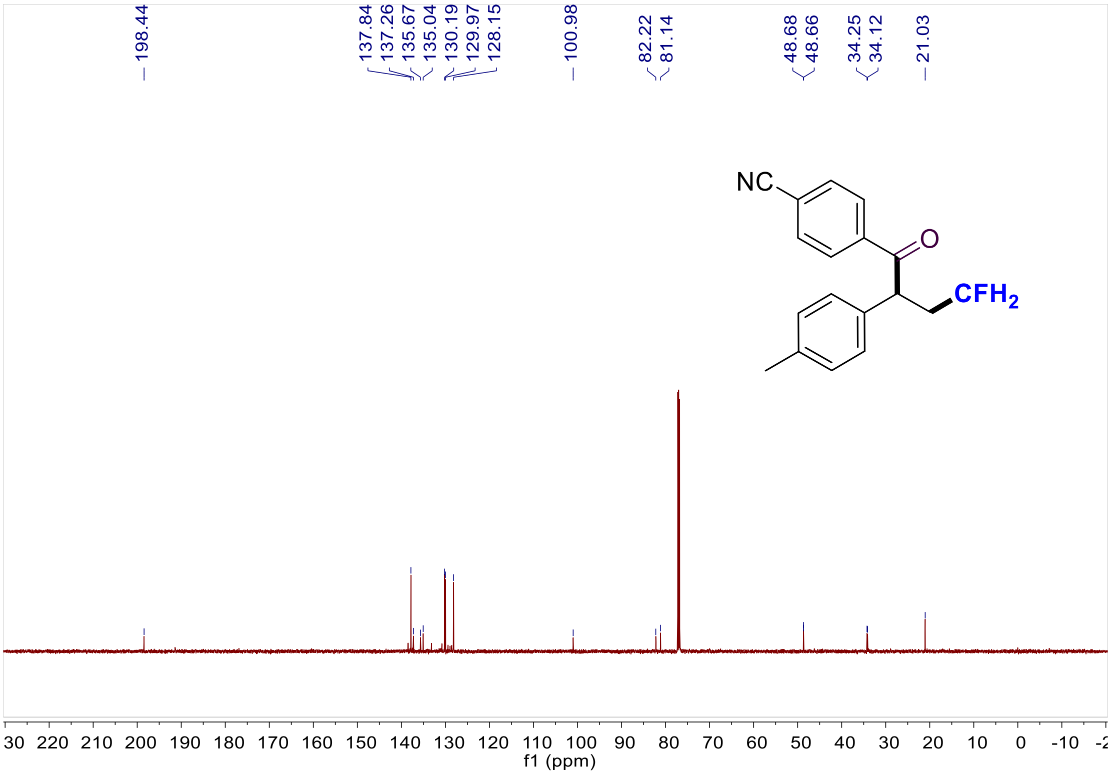

^{19}F NMR (565 MHz, CDCl$_3$) spectrum for **35**.

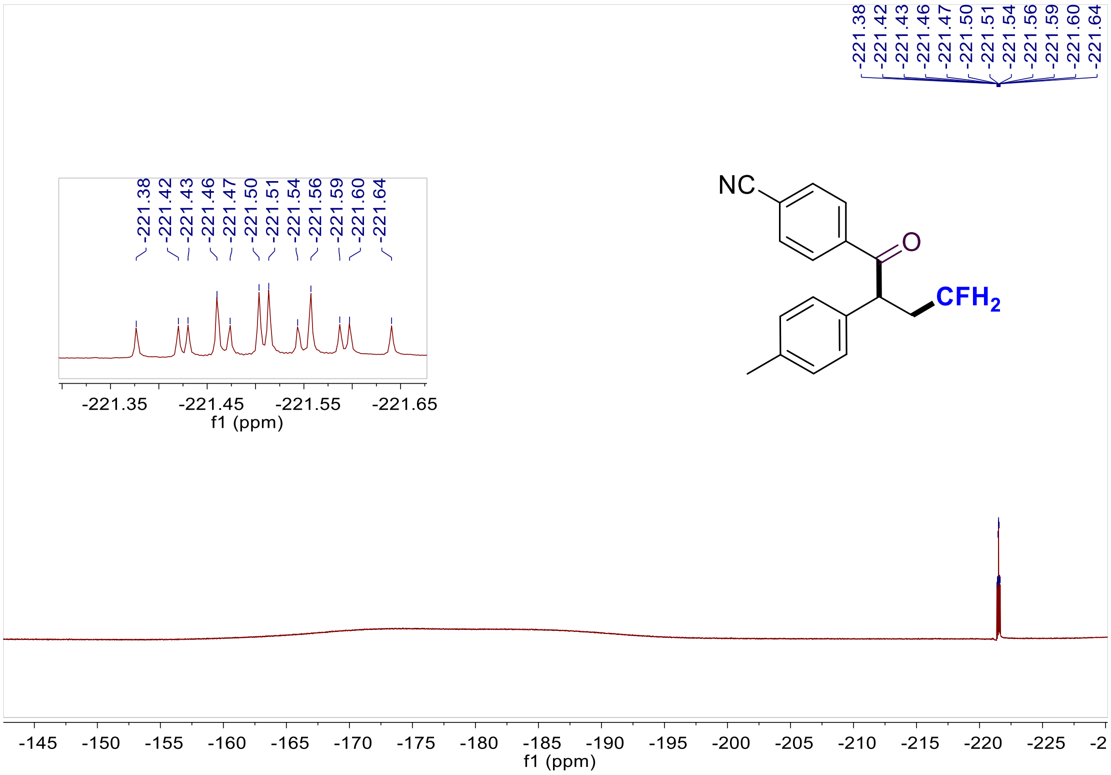

^{1}H NMR (500 MHz, $CDCl_3$) spectrum for **36**.

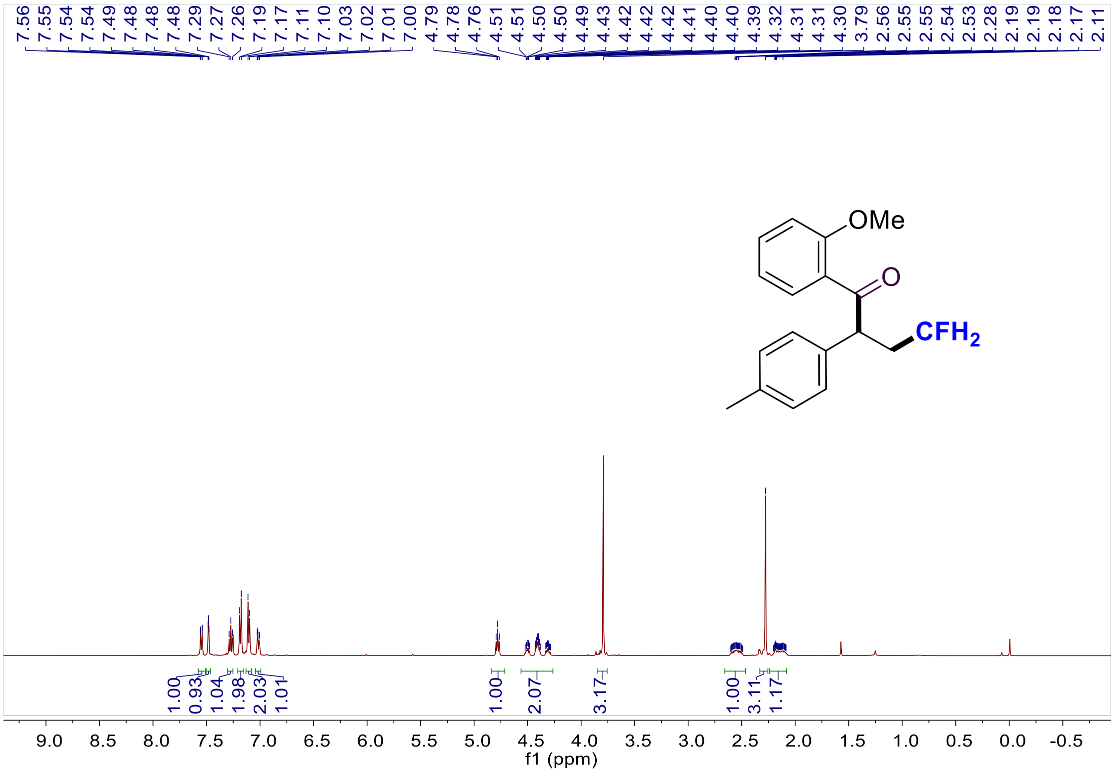

^{13}C NMR (151 MHz, $CDCl_3$) spectrum for **36**.

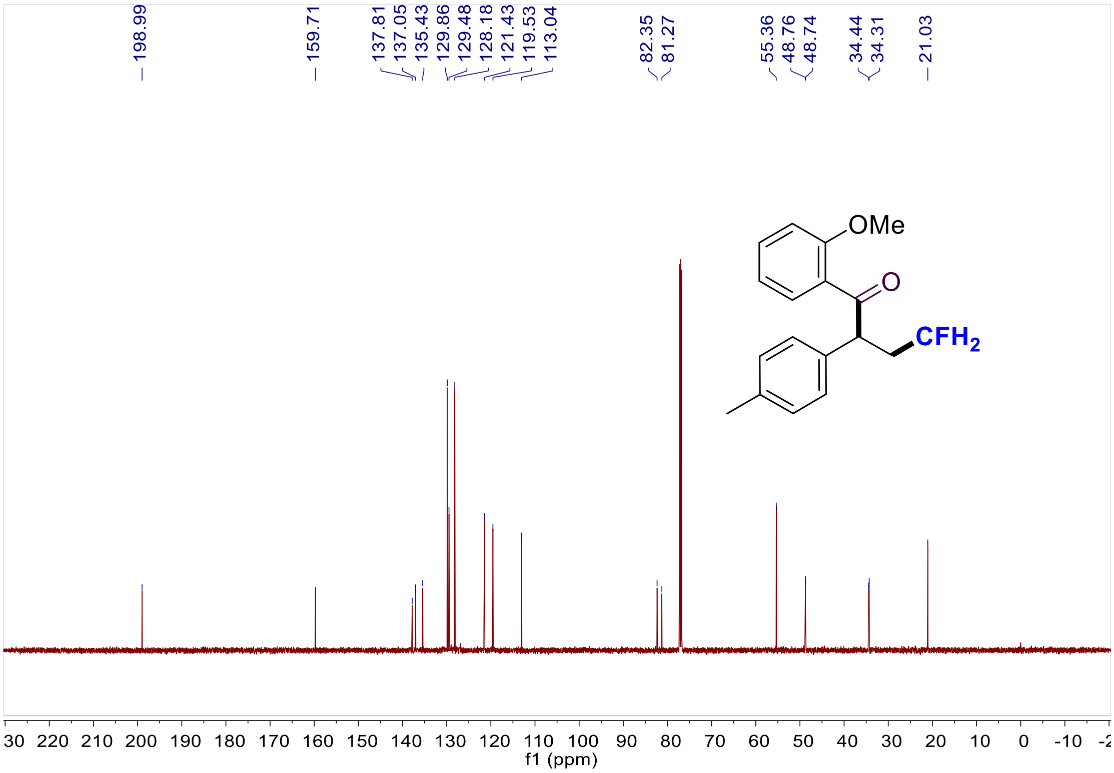

^{19}F NMR (565 MHz, $CDCl_3$) spectrum for **36**.

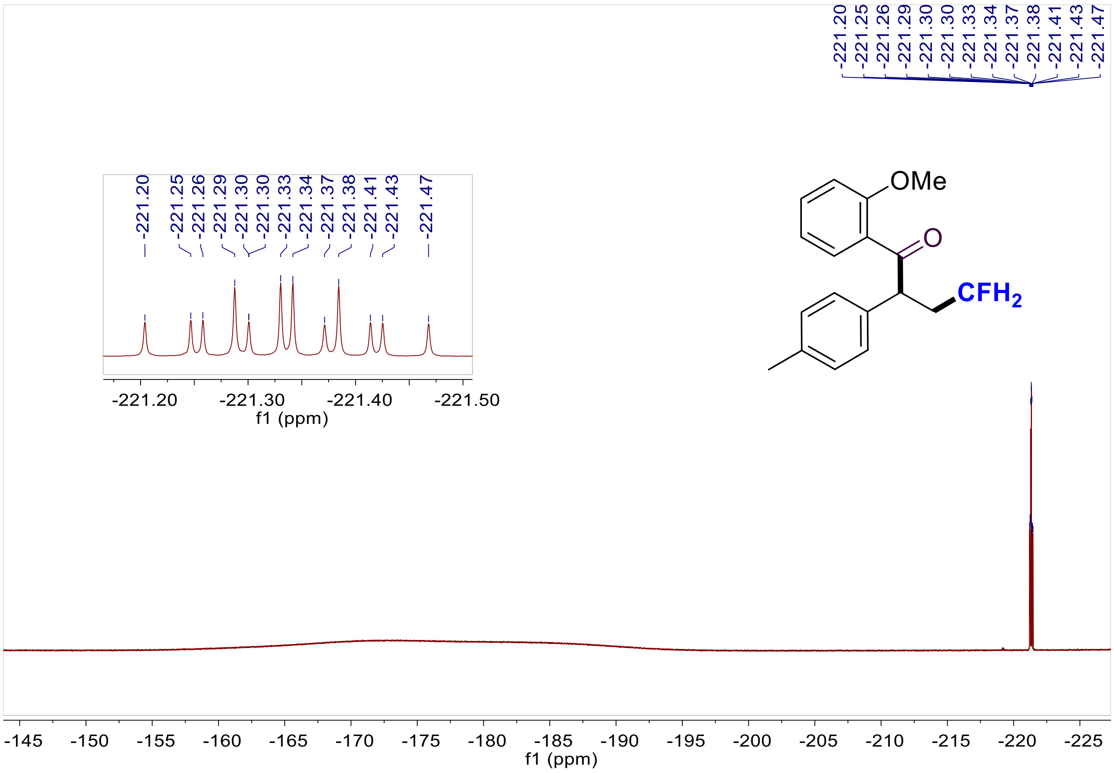

^{1}H NMR (500 MHz, $CDCl_3$) spectrum for **37**.

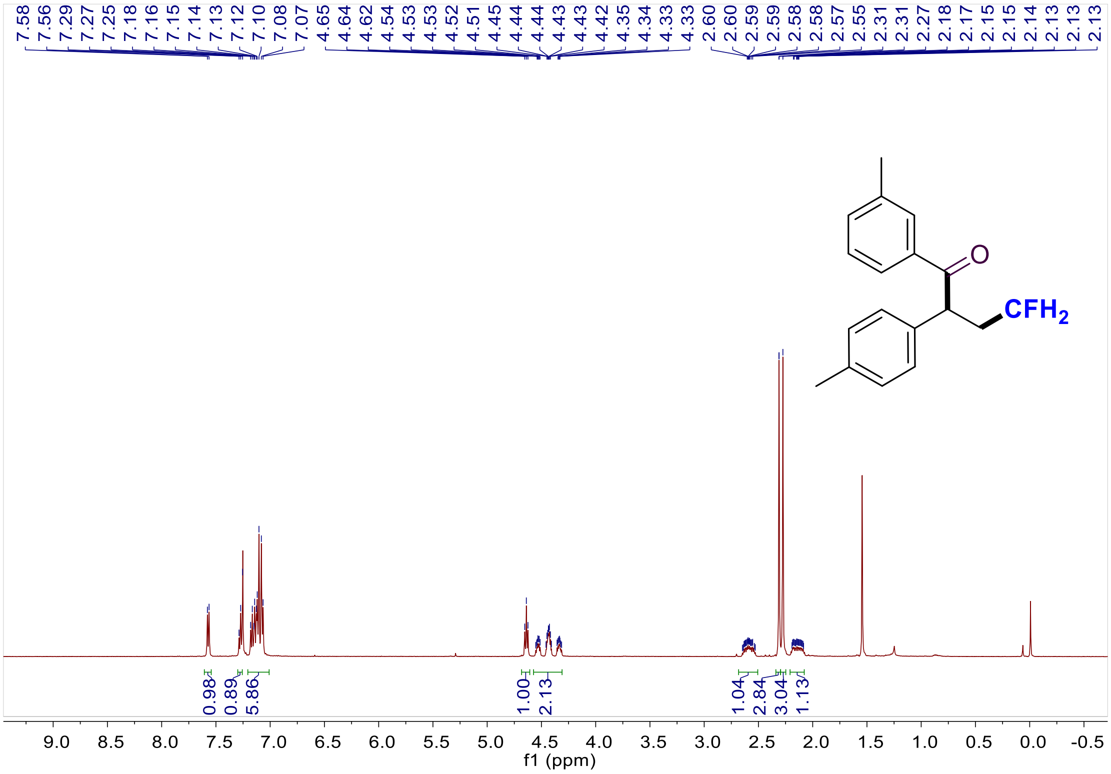

^{13}C NMR (151 MHz, $CDCl_3$) spectrum for **37**.

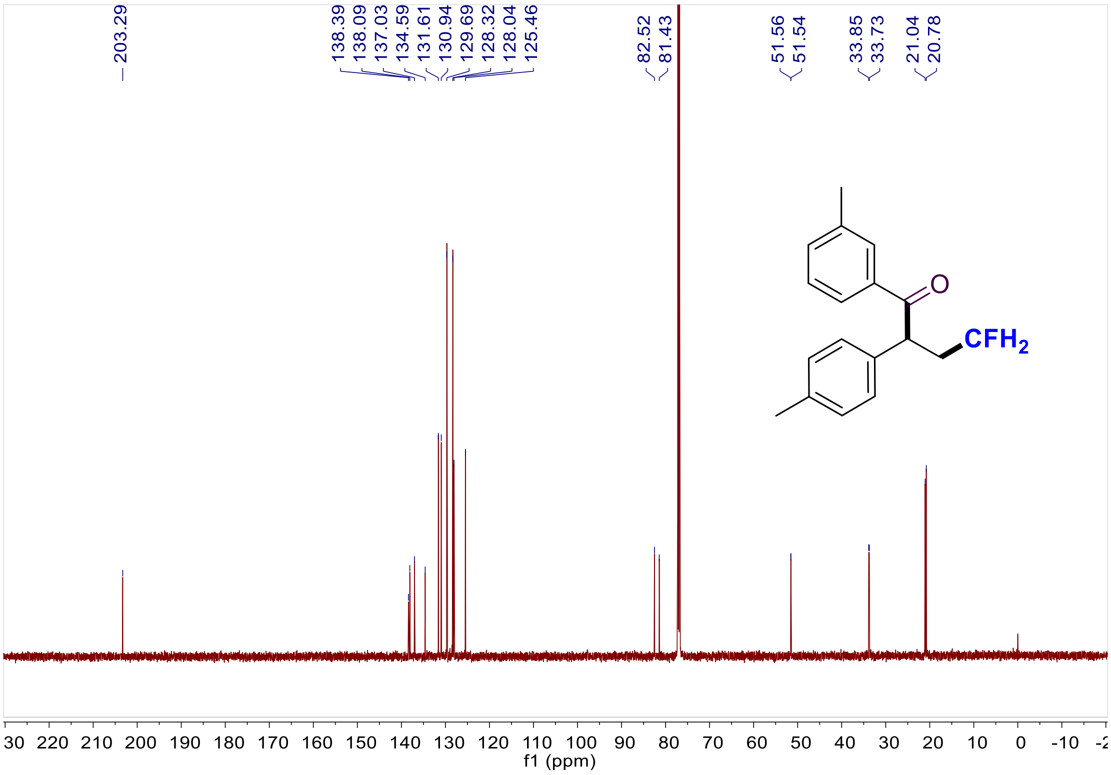

^{19}F NMR (565 MHz, $CDCl_3$) spectrum for **37**.

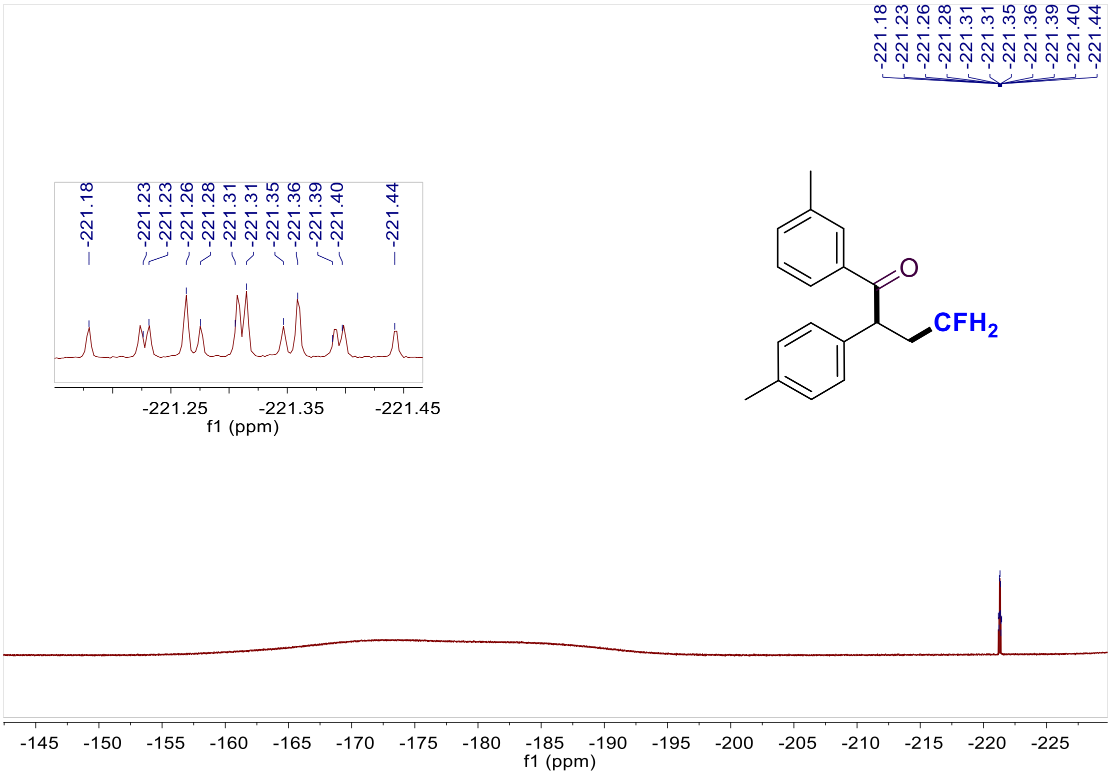

^{1}H NMR (500 MHz, $CDCl_3$) spectrum for **38**.

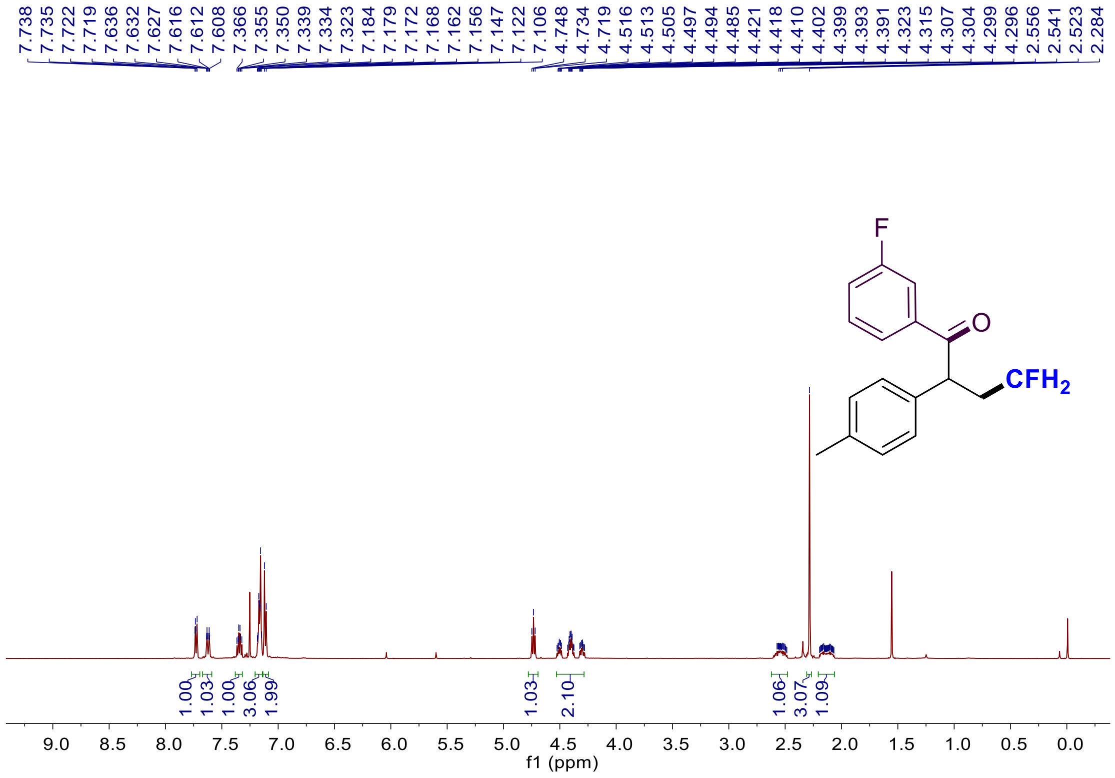

^{13}C NMR (151 MHz, $CDCl_3$) spectrum for **38.**

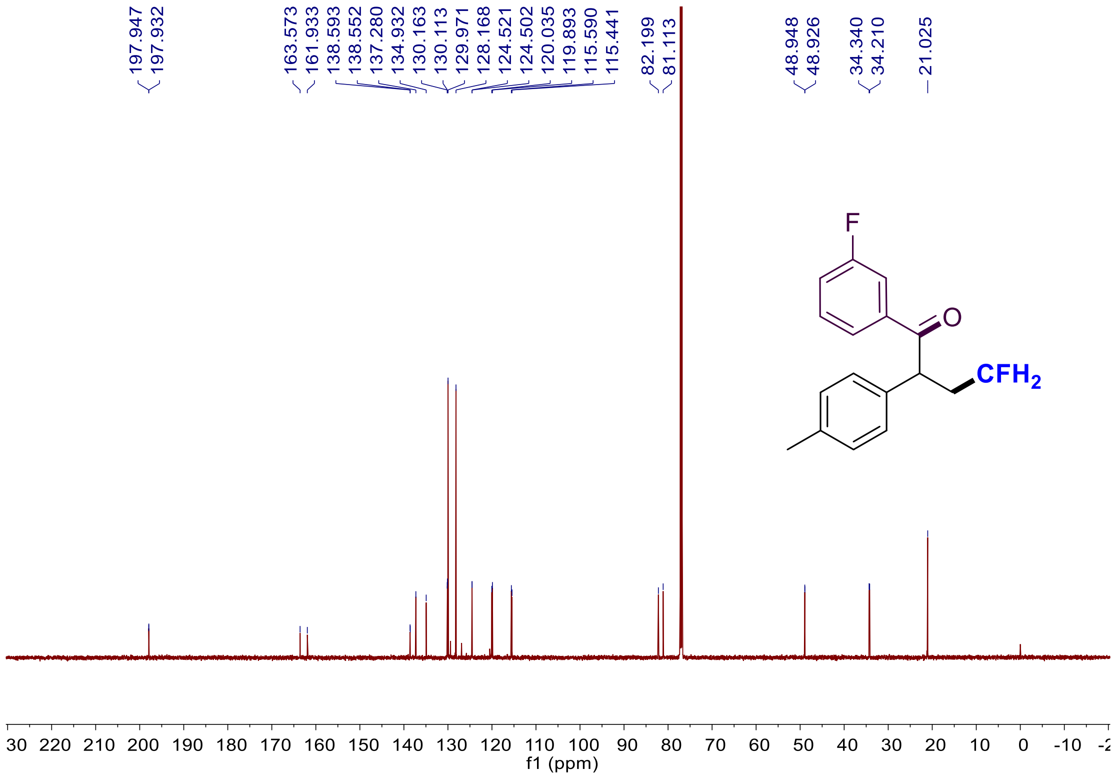

^{19}F NMR (565 MHz, $CDCl_3$) spectrum for **38**.

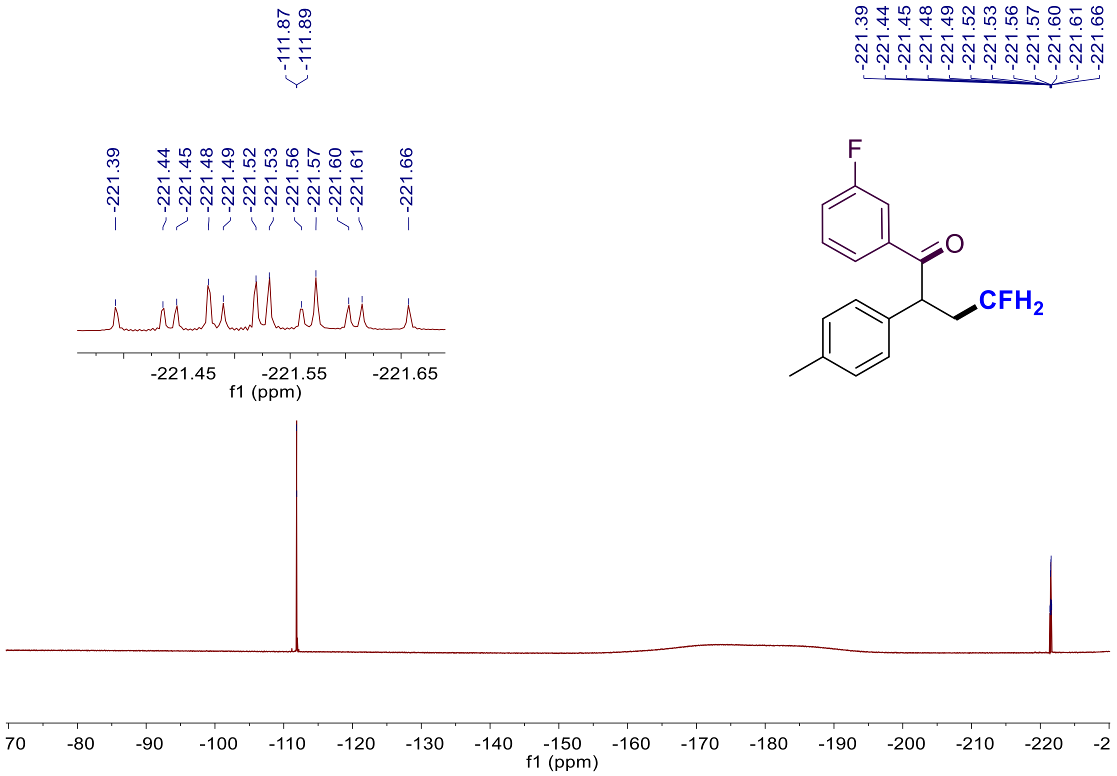

^{1}H NMR (500 MHz, $CDCl_3$) spectrum for **39**.

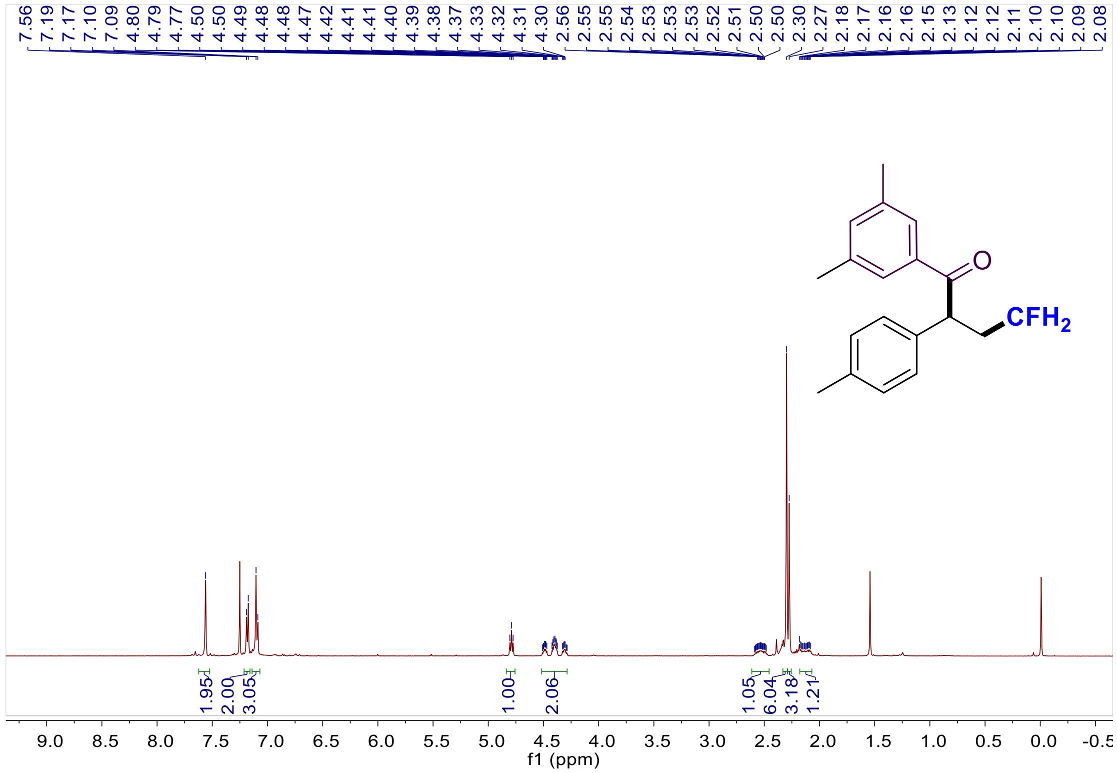

^{13}C NMR (151 MHz, $CDCl_3$) spectrum for **39**.

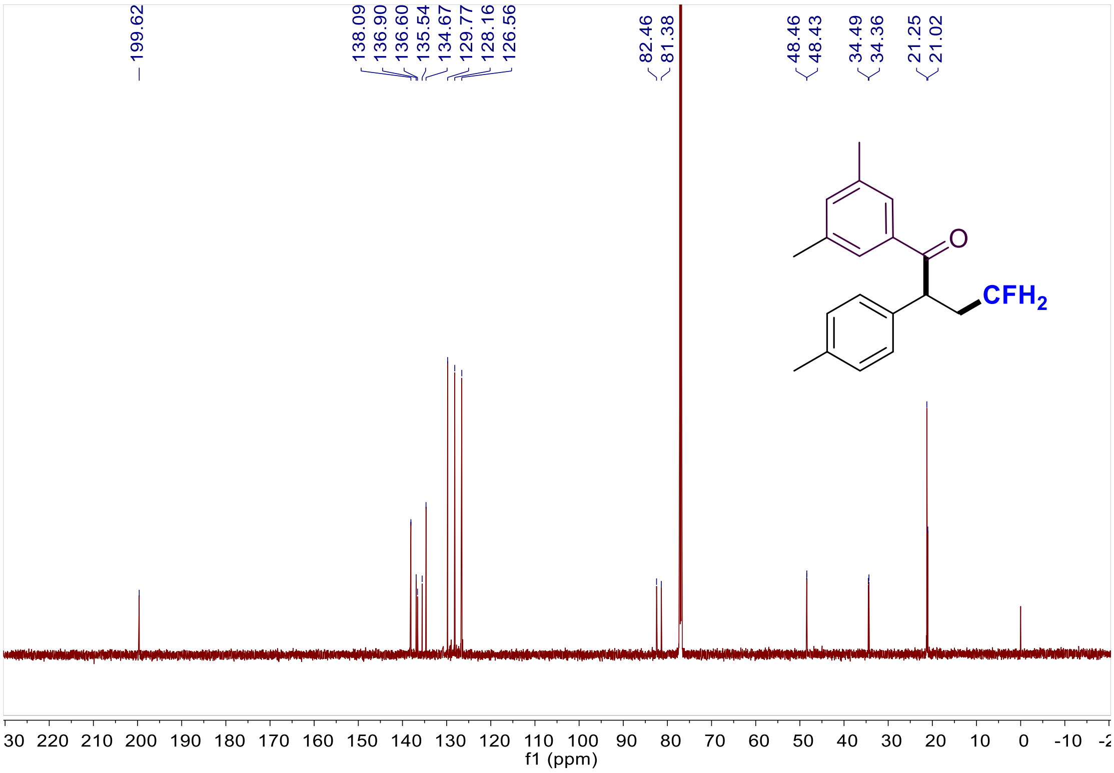

^{19}F NMR (565 MHz, $CDCl_3$) spectrum for **39**.

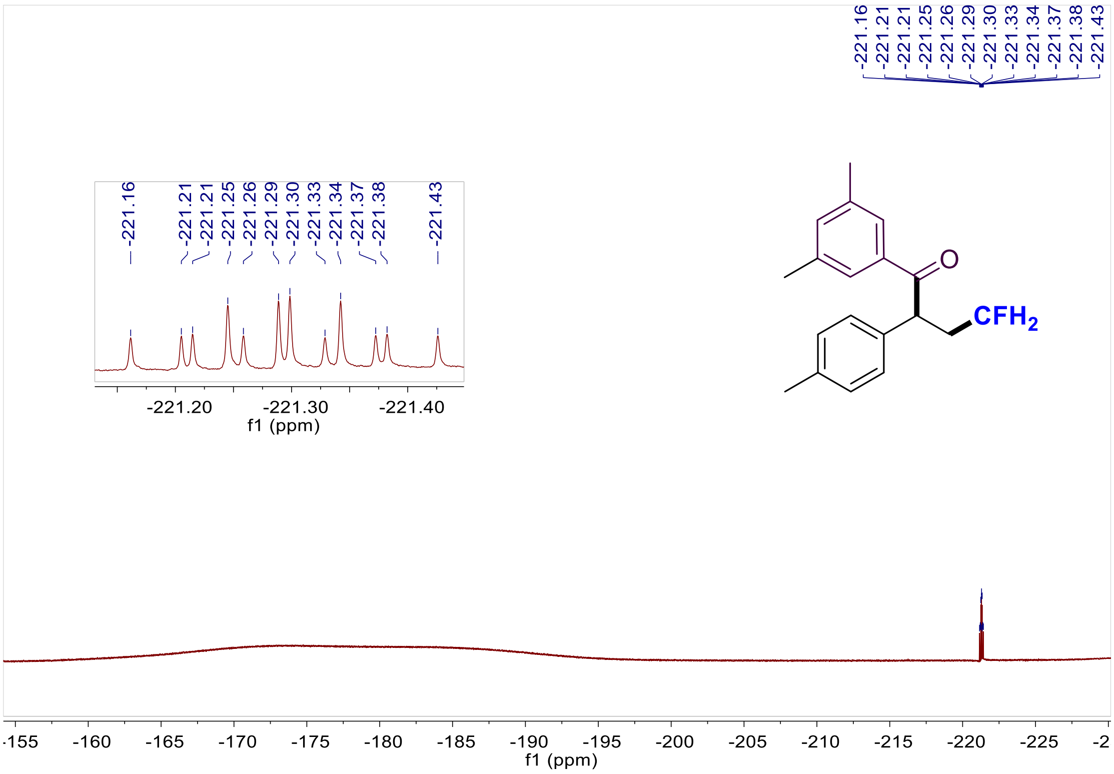

^{1}H NMR (500 MHz, $CDCl_3$) spectrum for **40**.

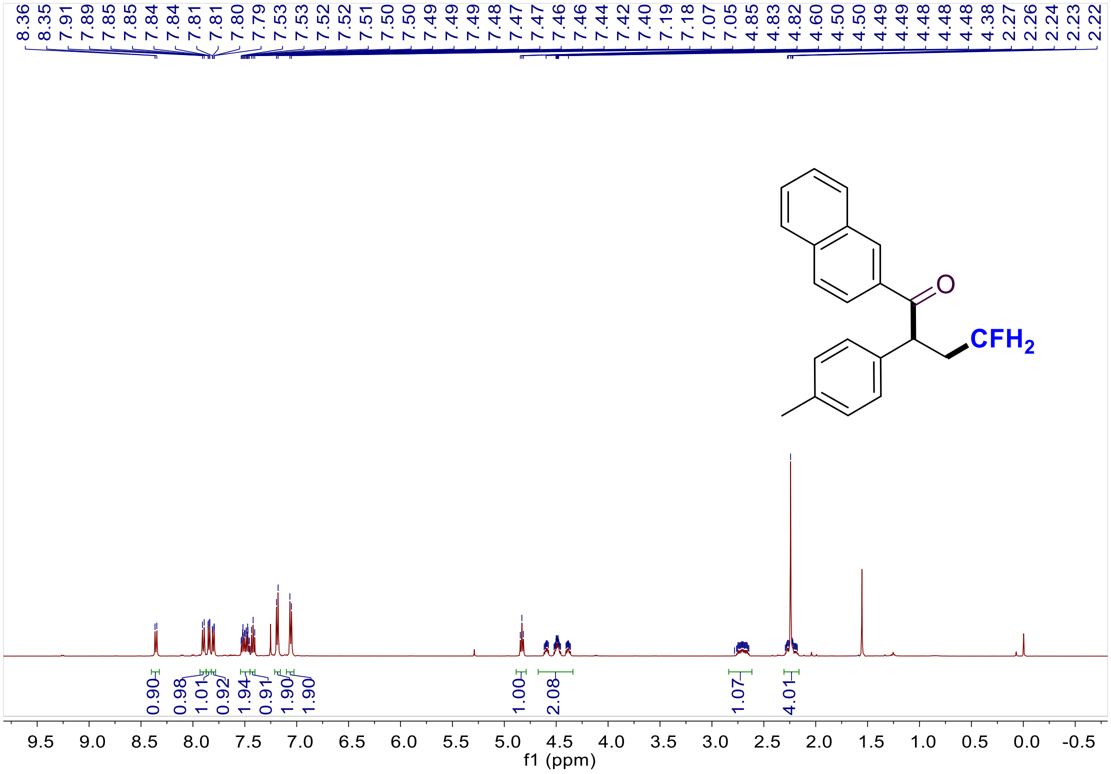

^{13}C NMR (151 MHz, $CDCl_3$) spectrum for **40**.

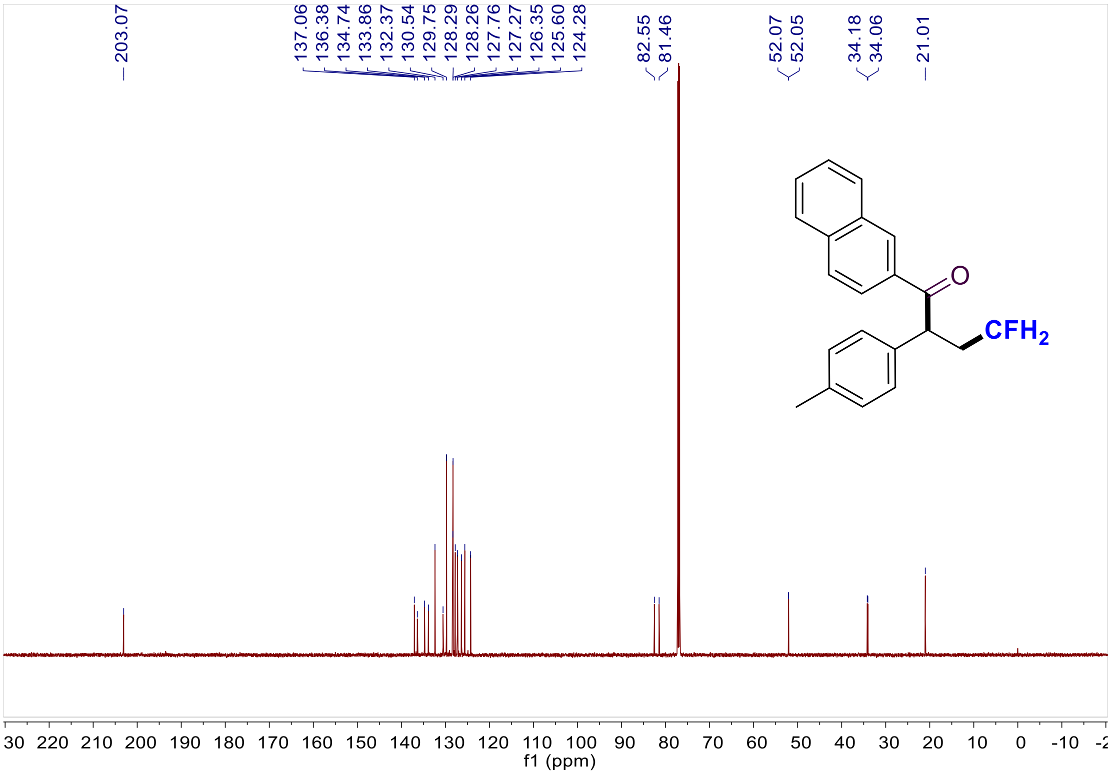

^{19}F NMR (565 MHz, $CDCl_3$) spectrum for **40**.

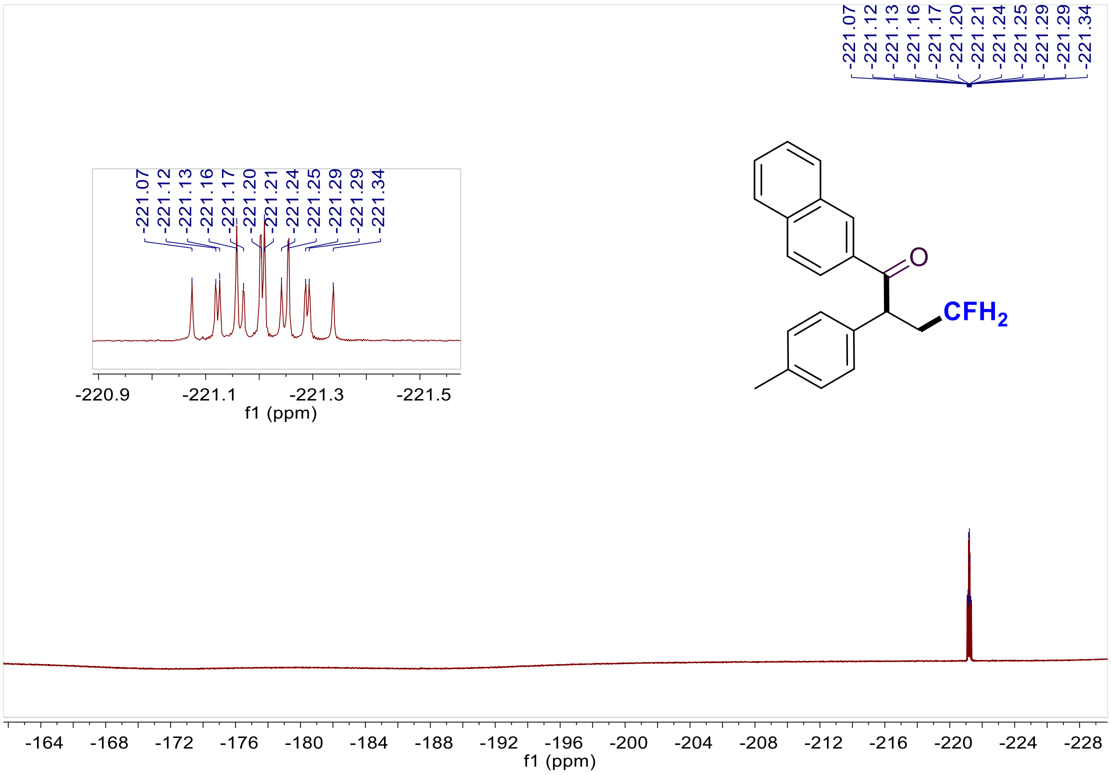

^{1}H NMR (500 MHz, $CDCl_3$) spectrum for **41**.

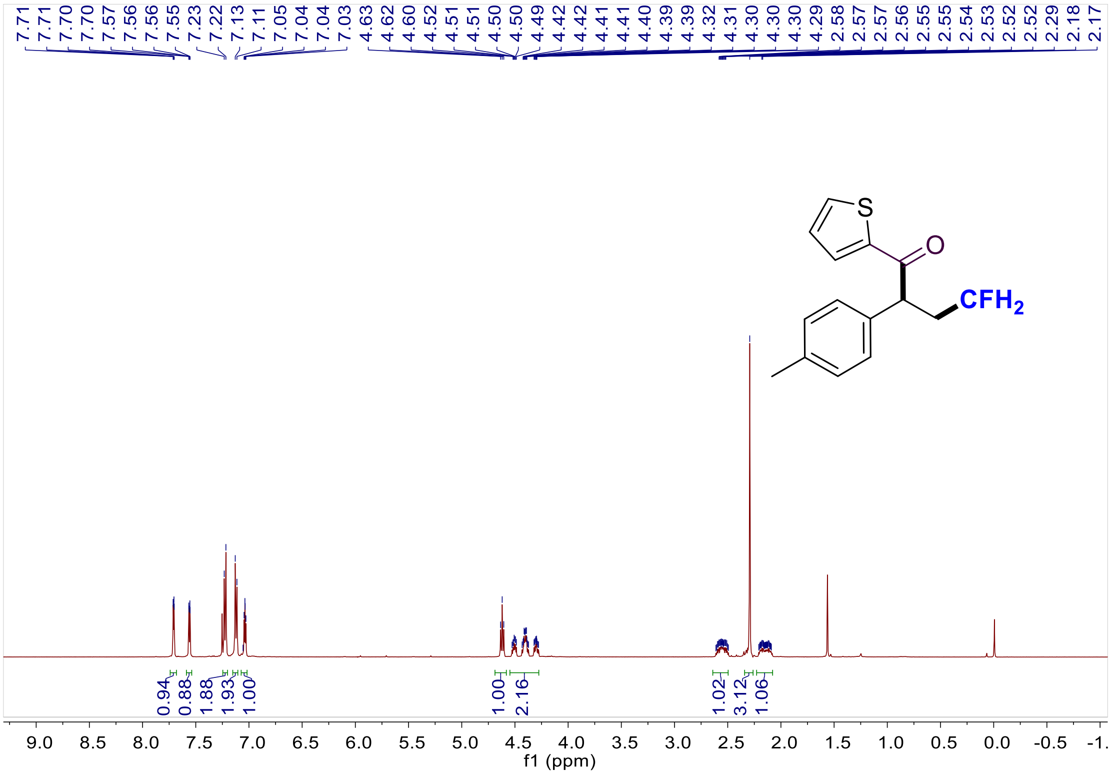

^{13}C NMR (151 MHz, $CDCl_3$) spectrum for **41**.

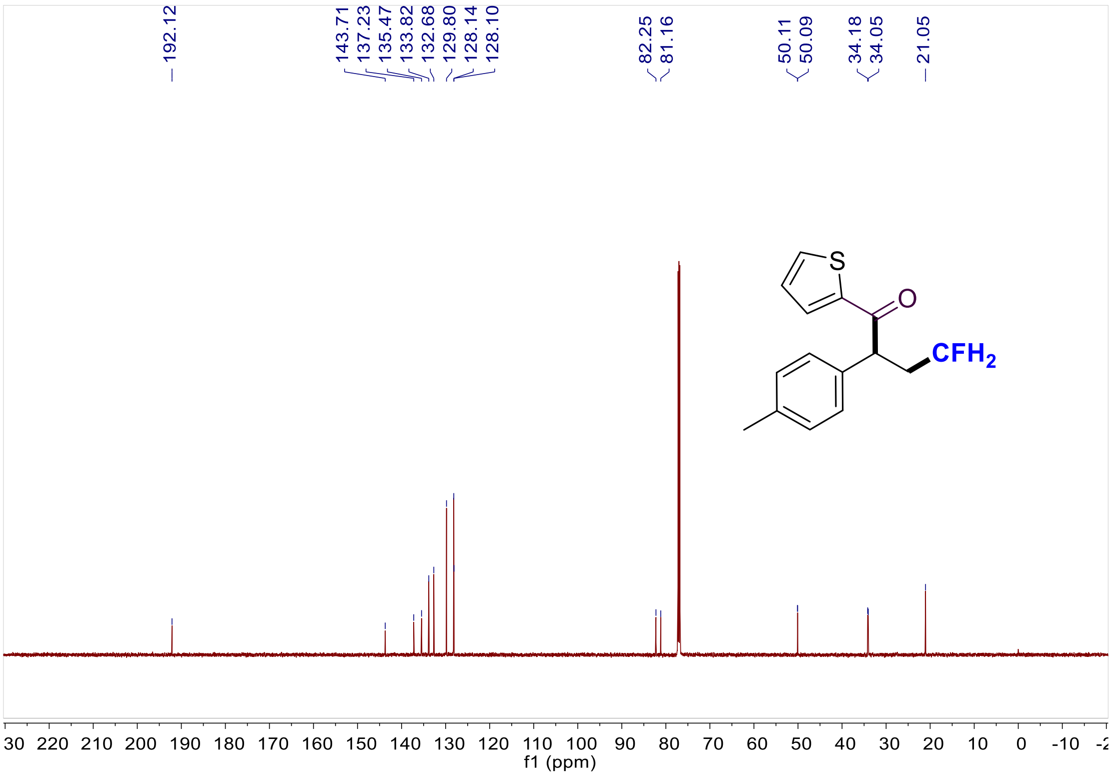

^{19}F NMR (565 MHz, $CDCl_3$) spectrum for **41**.

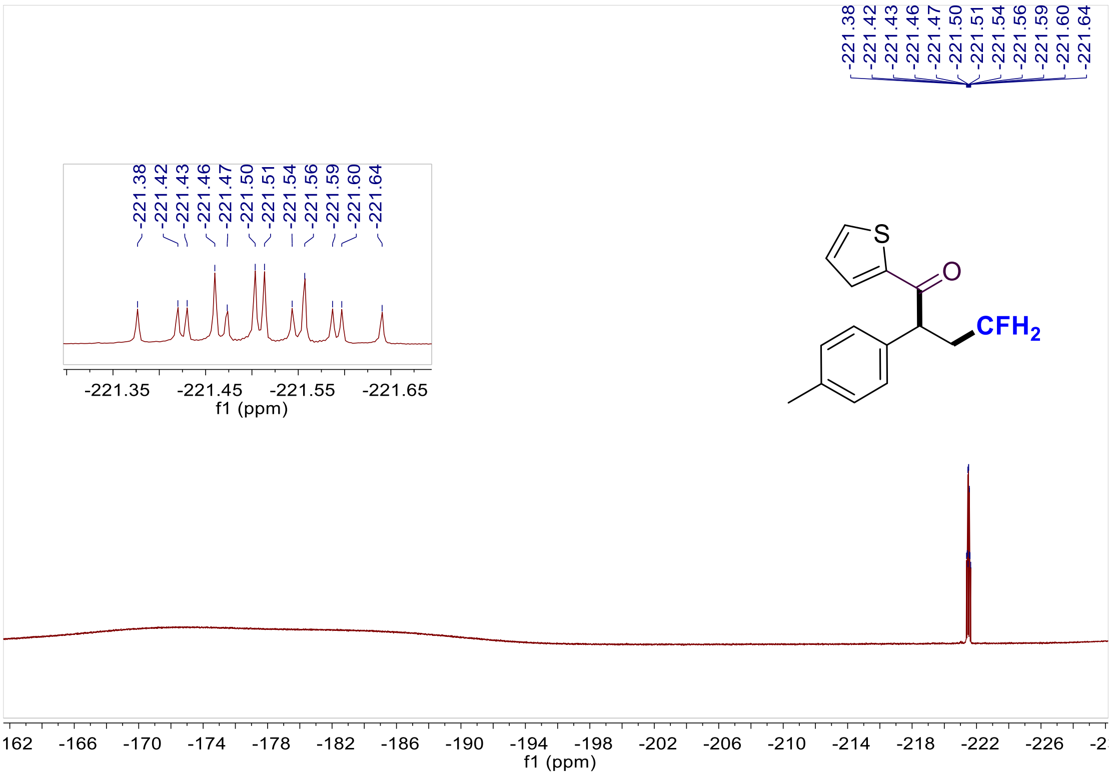

^1H NMR (500 MHz, $CDCl_3$) spectrum for **43**.

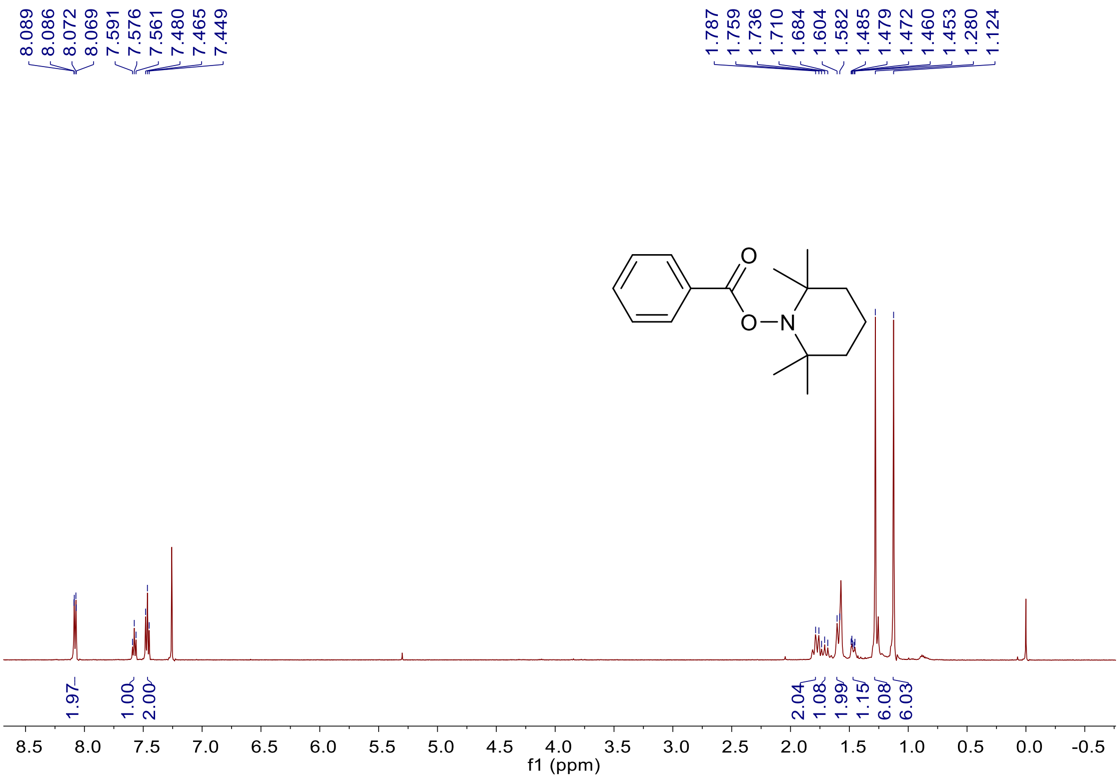

^{13}C NMR (151 MHz, $CDCl_3$) spectrum for **43**.

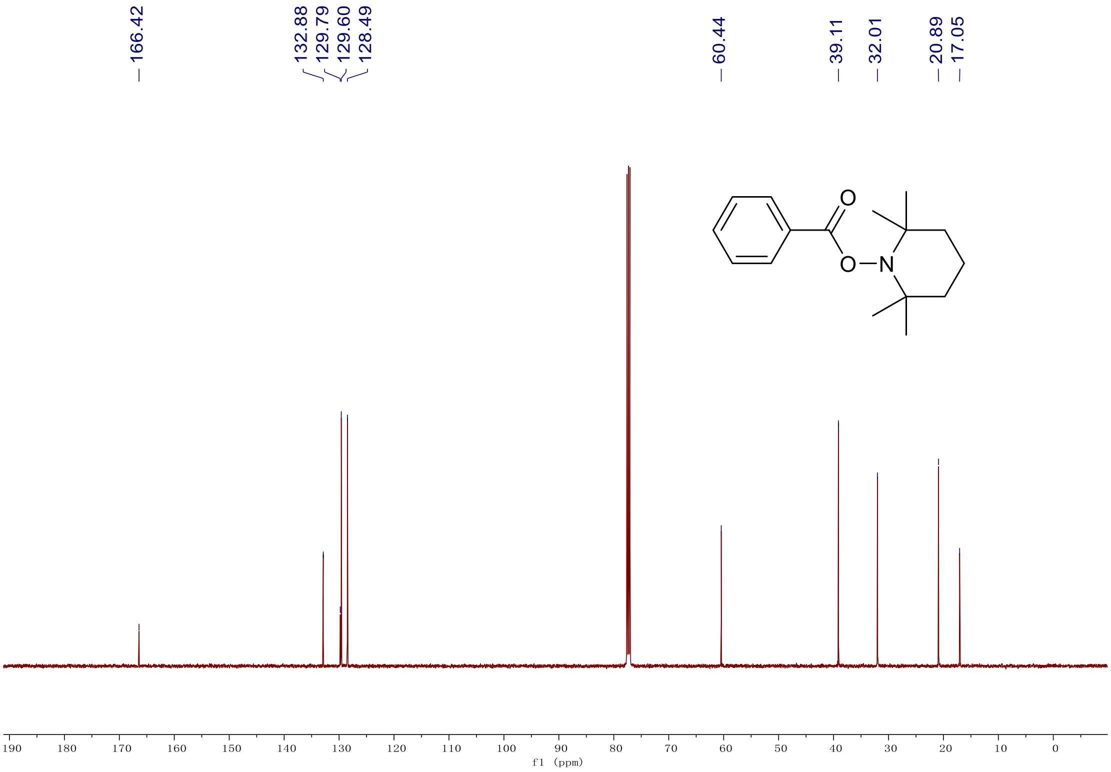

^{1}H NMR (500 MHz, $CDCl_3$) spectrum for **44**.

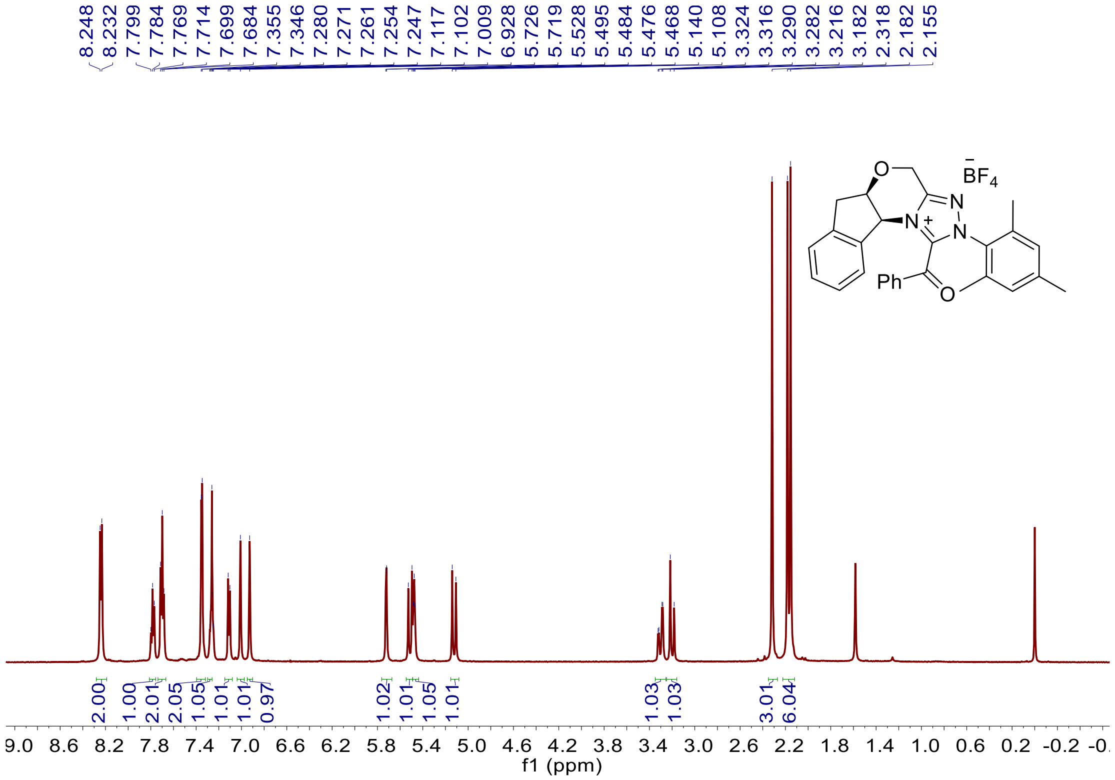

^{13}C NMR (151 MHz, $CDCl_3$) spectrum for **44**.

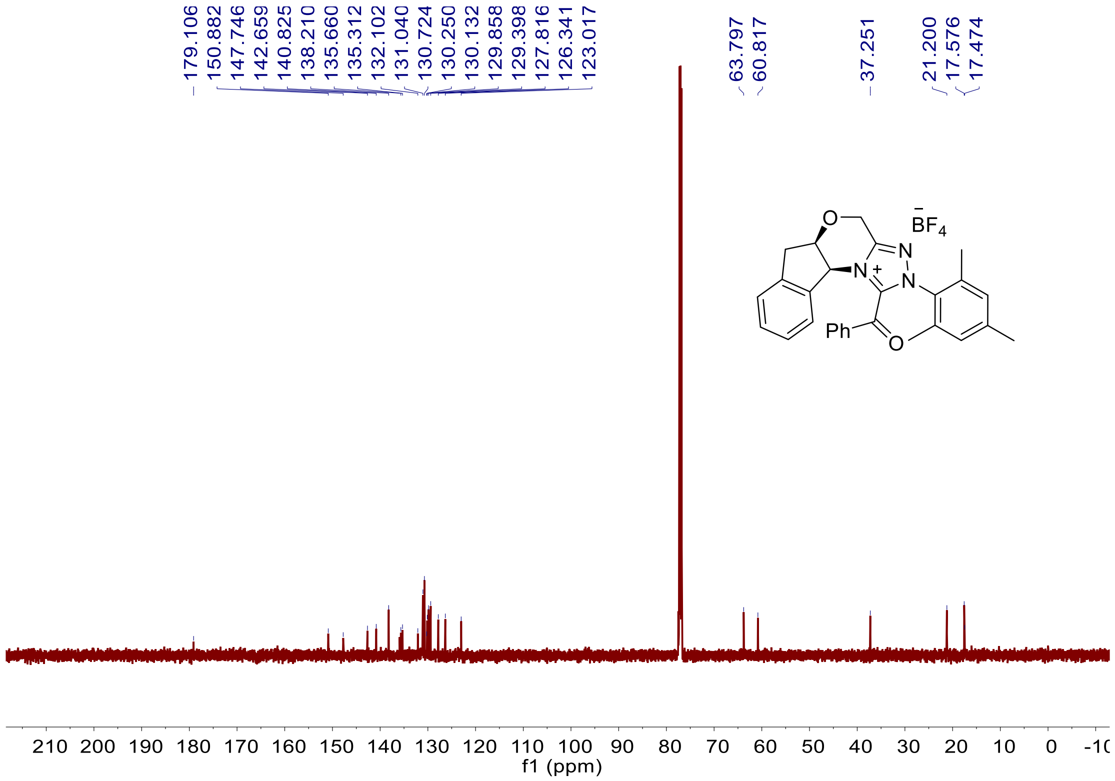

^{19}F NMR (565 MHz, $CDCl_3$) spectrum for **44**.

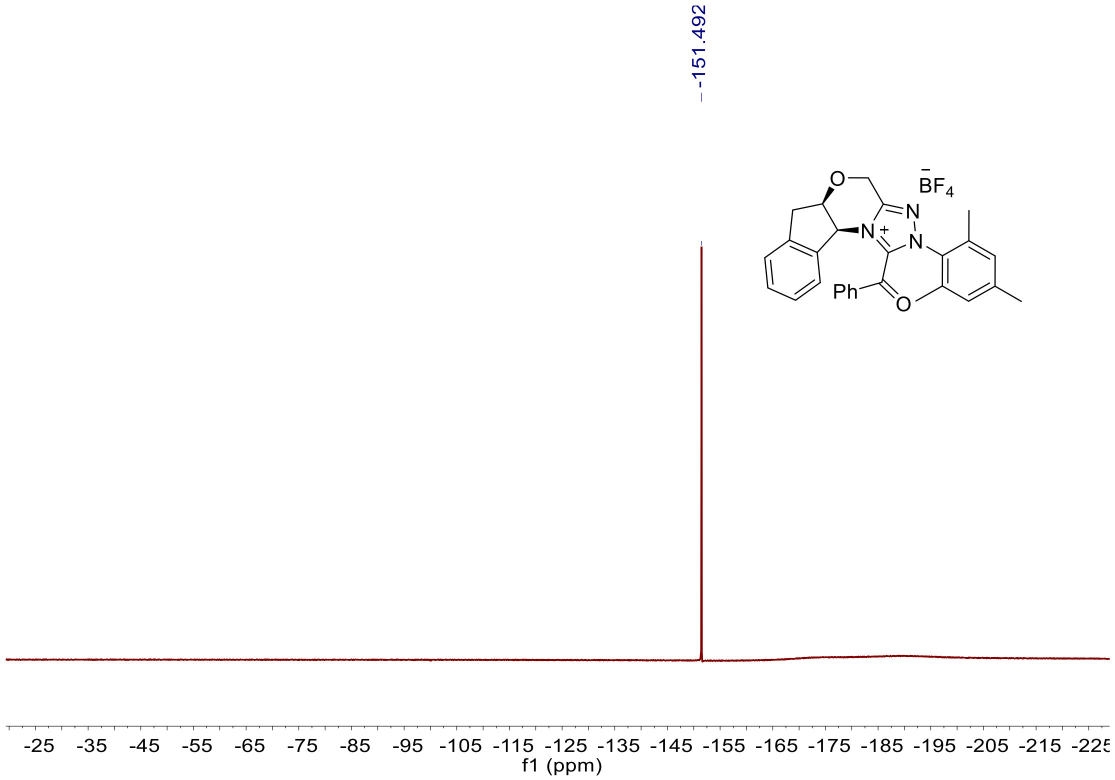

Supplement: Supplementary file 1 [file molecules-29-00790-s001.zip › molecules-2828426-supplementary.pdf]
